# Progressive failure mechanism and stability assessment of high-steep dangerous rock mass: A case study of Heicao dangerous rock mass

**DOI:** 10.1371/journal.pone.0336115

**Published:** 2025-11-10

**Authors:** Wu Yi, Yihua Dong, Xiaohu Huang, Tianzuo Wang, Yonghuang Deng, Zhengyu Wang

**Affiliations:** 1 Key Laboratory of Geological Hazards on Three Gorges Reservoir Area (China Three Gorges University), Ministry of Education, Yichang, Hubei, China; 2 College of Civil Engineering and Architecture, China Three Gorges University, Yichang, Hubei, China; 3 Key Laboratory of Rock Mechanics and Geohazards of Zhejiang Province, Shaoxing, Zhejiang, China; 4 Hubei Provincial Geological Environment Monitoring Station, Wuhan, China; 5 Xingshan County Land Consolidation Center, Yichang, Hubei, China; Guizhou University, CHINA

## Abstract

Western Hubei Province, China, lies in the transition zone between the second and third topographic steps of China’s terrain ladder system. Influenced by the deep incision of the Yangtze River and its tributaries, numerous high-steep slopes have been widely developed in this region.This study focuses on the Heicao dangerous rock mass in Xingshan County, Hubei Province. Through field investigations and Unmanned Aerial Vehicle (UAV) photogrammetry, a discrete element numerical model was developed to investigate the progressive failure mechanisms of high-steep dangerous rock masses under varying lengths of the main structural plane.This study will contribute to future prevention and control of such hazardous rock disasters.The principal conclusions are as follows: (1) The failure surface of the Heicao rock mass exhibits composite form,with the upper section displaying a polyline geometry and the lower section approximating an arc-shaped profile. (2) The main structural plane length significantly affects both the damage extent and propagation velocity in the upper rock mass. (3) Dangerous rock mass instability is jointly influenced by the basal rock mass and the overlying rock mass. Under gravity, progressive failure of the basal rock mass triggers crack development and anti-sliding force attenuation, while main structural plane propagation exacerbates damage. Ultimately, rock bridge shearing and crack coalescence lead to the formation of a progressively penetrating failure surface, resulting in global instability. (4) The results of this study have important theoretical and practical implications for future geological disaster assessment and prevention. In particular, the effective protection of the basement rock mass is the key factor to prevent the formation of dangerous rock mass disasters.

## Introduction

Western Hubei Province, China, lies in the transition zone between the second and third topographic steps of China’s terrain ladder system. Influenced by the deep incision of the Yangtze River and its tributaries, a large number of high and steep slopes are widely distributed on both sides of the region [1]. Such high-steep slopes are highly prone to rockfall hazards. Collapse events often involve rock masses exceeding 100 m^3^, occurring on slopes steeper than 60°, with sources located over 100 m above ground. For example, in October 2017, about 200 m^3^ of rock collapsed on the Heicao Slope, destroying a village road and depositing debris at the slope toe. Investigations revealed that a tensile crack approximately 30m in length had developed at the rear of the rock mass near the slope crest.Additionally, a potentially dangerous rock mass of approximately 2500 m^3^ remains on the high-steep slope. Under gravitational influence, this dangerous rock mass exhibits a tendency to move toward the free surface.Therefore,the failure mechanism of high-steep dangerous rock masses is an essential and unavoidable issue in geohazard studies. Scholars have classified the failure types of dangerous rock masses based on different perspectives, including movement patterns [2], stability-controlling factors [3], and geomorphological characteristics [1,4]. In addition, the failure mechanisms of dangerous rock masses remain a central research focus. Numerous studies have shown that slope structural type, lithological combination, structural plane characteristics, and human activities are the main factors affecting slope stability. Among them, the characteristics of structural planes and the rock-bridge effect play a key role in triggering the failure of dangerous rock masses [5–8]. In the field of slope stability analysis and risk assessment, extensive research has been carried out worldwide. Theoretically, catastrophe theory has been introduced to establish stability criteria [9], and multi-factor mathematical evaluation models have also been developed [10]. In terms of numerical analysis, advanced techniques such as three-dimensional discrete element methods [11,12] and the three-dimensional numerical manifold method [13] have been widely applied. In particular, by coupling with conventional strength criteria, these methods have enabled refined simulations of the progressive failure process of jointed slopes [14].

Research methodologies play a crucial role in the study of high-steep dangerous rock masses. The progressive failure of dangerous rock masses is a macroscopic manifestation of the gradual penetration of structural planes within the rock mass. Therefore, the precise acquisition of slope structural plane parameters is key to constructing reliable models of dangerous rock masses. In recent years, various advanced technologies have been widely applied in this field. Three-dimensional laser scanning has been used to obtain slope boundary point cloud data [15], analyze structural plane characteristics [16,17], and study the distribution of landslide deposits [18,19]. Unmanned aerial vehicle (UAV) photogrammetry, due to its high efficiency, has achieved significant progress in measuring joint roughness on steep slopes [20], automatic fracture identification [21], and three-dimensional modeling of dangerous rock masses [22]. Close-range photogrammetry is particularly suitable for the detailed characterization of rock surface features [23]. In comparison, 3D laser scanning and close-range photogrammetry have limitations in measuring high-steep slopes due to their lower accessibility, whereas UAV-based photogrammetry has been widely applied for acquiring structural plane parameters of rock masses. Therefore, in this study, UAV oblique photogrammetry was employed to obtain surface structural parameters of the Heicao dangerous rock mass, and the results were compared and validated against manual measurements.

In addition, in the simulation of dangerous rock masses, existing approaches can be broadly divided into physical model tests and numerical simulations. Physical model tests construct scaled laboratory models and apply loading methods such as geotechnical centrifuges or shaking tables to simulate slope failure under gravity or seismic conditions. These tests provide intuitive evidence for understanding the deformation and failure mechanisms of rock slopes [24–27]. Numerical simulations include techniques such as the finite element strength reduction method [28] and the discrete element method [29,30], which can effectively capture the progressive failure process of fractured rock masses. In particular, three-dimensional discrete element methods show unique advantages in representing meso-scale damage and fracture evolution in rock masses [31–33]. Compared with other approaches, the 3D discrete element method offers high modeling flexibility and convenient monitoring, making it especially valuable in slope stability studies. Based on this method, the present study investigates the failure mechanisms of dangerous rock masses to obtain more reliable simulation results.

Decades ago, scholars such as Casagrande, Terzaghi, and Bishop conducted extensive research on the progressive failure of slopes. Zhou et al. [34] proposed that all rock mass failures, except for rockbursts, can be classified as progressive failures. Under self-weight and other influences, local rock mass cracking occurs first, reaching the strength limit. Subsequently, stress decreases in the failure zone, leading to a reduction in load-bearing capacity and the expansion of the failure region, ultimately resulting in through-going rupture surfaces. However, at present, research on the progressive failure of high-steep dangerous rock masses remains incomplete both domestically and internationally. Meanwhile, numerous cases of dangerous rock mass hazards have occurred in recent years, resulting in a large number of casualties. These incidents highlight the urgent need to investigate the progressive failure mechanisms of high-steep rock masses. The Heicao dangerous rock mass is located on a high-steep slope and is characterized by a well-defined main structural plane. Local rock masses are segmented into multiple independent rock blocks due to joint and crack cutting. Under external influences, the rock mass undergoes progressive failure along the controlling structural plane, leading to a gradual decrease in stability and potentially resulting in hazardous events. This underscores the typical nature of the Heicao dangerous rock mass and the significance of its study. Therefore, this study takes the Heicao dangerous rock mass in Xingshan County, Hubei Province, as a case study. By combining field investigations and UAV aerial photography, establish an accurate 3DEC numerical model of the Heicao rock mass using the discrete element method. The research focuses on the progressive failure processes of high-steep dangerous rock masses under varying lengths of main structural planes, providing critical theoretical support for understanding their instability mechanisms. The findings will contribute significantly to future prevention and control measures for such dangerous rock mass disasters.

## Overview of the study area

### Study area

**Fig 1:**
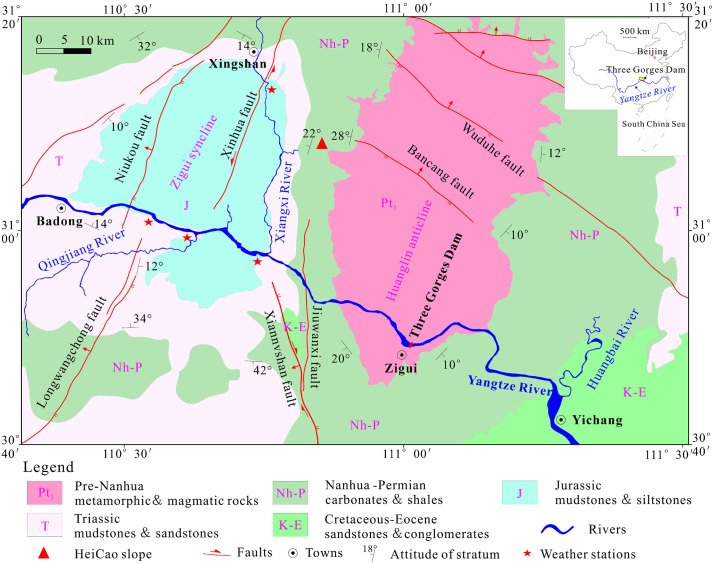
Regional setting and location of the study area.

The Heicao slope is located on the left bank of the Gaolan River in Xiakou Town, Xingshan County, Yichang City, Hubei Province, with geographic coordinates of 110°51′24″E and 31°08′10″N (Fig 1). The study area features elevation differences from 200 m to 1800 m, with slope angles between 30° and 70°. It is mainly composed of mid- to low-altitude mountains shaped by tectonic denudation and river incision. Several NW-SE trending faults exist within the region, among which the Bancang Fault is the closest to the study area. The overall regional stability is relatively high, classifying it as a relatively stable zone. The study area is situated in a subtropical continental monsoon climate zone, with current climatic conditions featuring an average daily temperature range of 3.8 ~ 35.3°C and an annual precipitation of 900 ~ 1400 mm, indicating abundant rainfall. The exposed stratigraphy mainly comprises Quaternary colluvial deposits and the Upper Sinian Doushantuo Formation (Z_2_d), which consists of medium- to thick-bedded dolomite and limestone. The bedding occurrence is dip direction 140–160°, and the dip is 5–35°.

### Situation of rock mass

**Fig 2:**
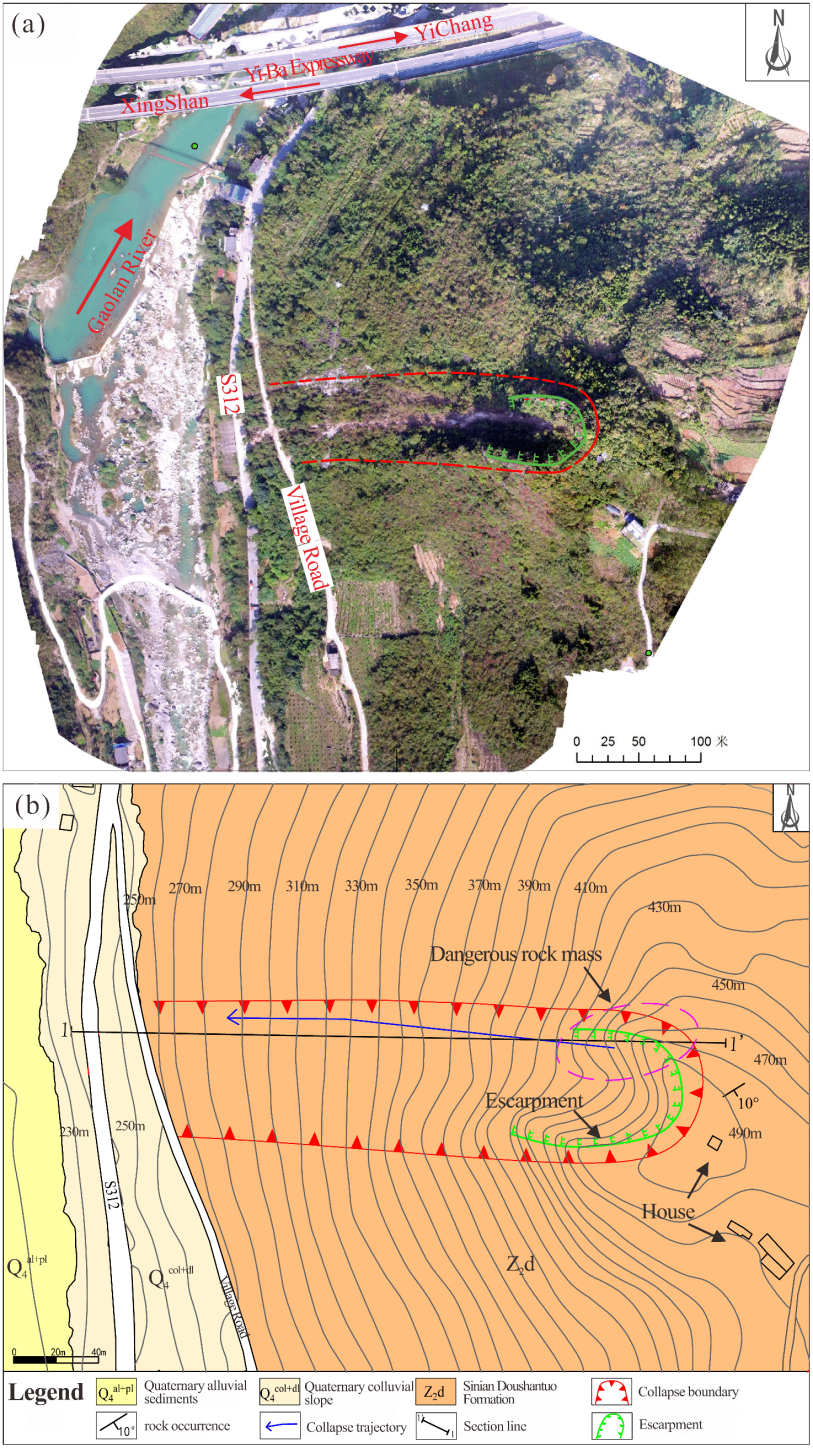
(a) Panoramic view of the Heicao slope. (b) Engineering geological plan.

**Fig 3:**
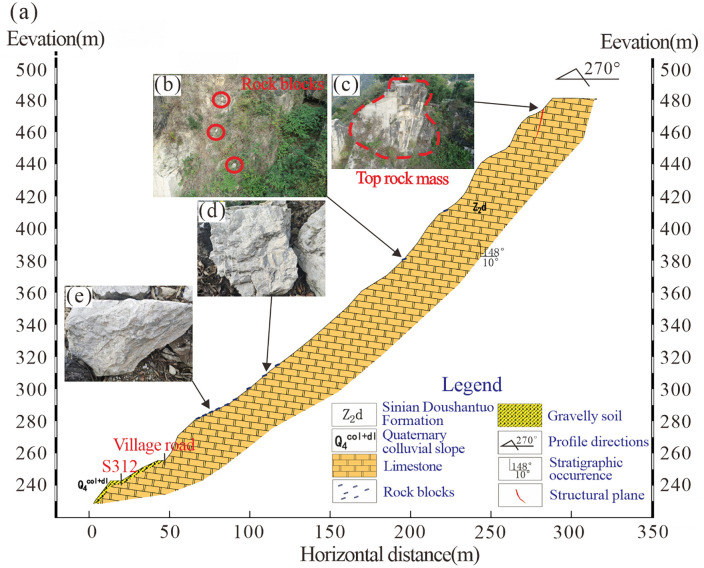
Cross-sectional view of the Heicao slope (Slope orientation 270°, gradient 30°-70°).

The Heicao slope is located on the left bank of the Gaolan River in Xiakou Town, Xingshan County, Yichang City. The area has well-developed transportation infrastructure, with the Yi-Ba Expressway, Provincial Highway 312, and rural roads in close proximity, as shown in Fig 2. The Heicao slope exhibits an oblique counter-tilted structure, characterized by a steep upper section and a gentler lower portion, with a slope orientation of 270° (Fig 3a). As a super-high slope (elevation>300 m), it features well-developed joint fissures within the rock mass. Two main structural planes are observed, with occurrence of 305–355° (dip direction) and 60–80° (dip), as well as 60–75° (dip direction) and 50–70° (dip). These two fracture surfaces, along with the bedding plane, constitute the primary structural controls of the slope. The fractures, exhibiting good connectivity, define the deformation boundary, while the bedding plane governs the sliding surface.Scattered rock blocks, approximately 1.5 m^3^ in volume, are present in the middle-to-lower part of the slope (Fig 3b, 3d, and 3e). At the top of the slope, a high and steep dangerous rock mass, known as the Heicao dangerous rock mass, is present (Fig 3c).This dangerous rock mass is located at an elevation ranging from 420 m to 480 m, with a height of 50 m, a width of 10 m, and an average thickness of 5 m, resulting in a total volume of 2500 m^3^. The rock mass primarily consists of limestone from the Doushantuo Formation.

**Fig 4:**
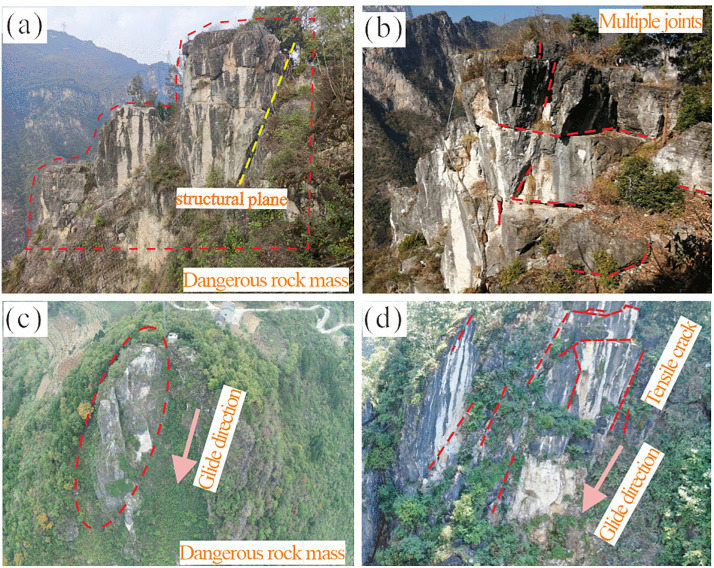
Geological survey map of the dangerous rock mass: (a-b) Manual survey images. (c-d) UAV survey images.

Due to the steep terrain of the slope hosting the dangerous rock mass, manual measurements of lithology and structural attitudes were conducted on proximal rock masses. Additionally, UAV-based photogrammetry was employed to capture detailed images of the Heicao slope and specific sections of the dangerous rock mass, as shown in Fig 4. According to the manual measurement, the occurrence of the slope rock stratum is dip direction 148° and the dip is 10°, classifying it as a gently dipping anti-dip slope. Field survey images reveal that the Heicao dangerous rock mass (Fig 4a) exhibits well-developed surface cracks and an overall blocky distribution. The base cracks are relatively well-developed, forming a structurally weak layer that gives the rock mass a “top-heavy” characteristic. The morphology of the dangerous rock mass and its primary controlling structural planes are clearly identifiable, with the main controlling structural plane measuring approximately 30 m in length and the occurrence is dip direction 310°, and the dip is 67°. The aperture of the structural plane gradually decreases from top to bottom, with crack apertures ranging between 10 mm and 80 mm. At the rear edge of the dangerous rock mass (Fig 4b), multiple rock blocks formed by joints cutting are visible. The bedding plane exhibits highly developed rock mass cracks accompanied by relative displacement differences. Measurements indicate that the maximum width of the tensile crack at the rear edge reaches approximately 1 m.Using UAV photogrammetry, a comprehensive survey of crack development across the entire Heicao dangerous rock mass and its localized rock surfaces was conducted. As shown in Fig 4c, a three-sided free-face geometry at the rock mass location, with exposed bedrock and clear joint distributions, and erosion gullies formed by falling rock blocks are present. Fig 4d presents a close-up view of the dangerous rock mass, showing significant weathering, well-developed cracks, and a predominance of tensile cracks. These cracks range from approximately 2 ~ 3 m in length and are primarily open-type, with a spacing of approximately 0.15 ~ 1.0 m, exhibiting strong connectivity. Additionally, extensive vegetation within the rock cracks further accelerates crack development.

### Historical deformation feature

**Fig 5:**
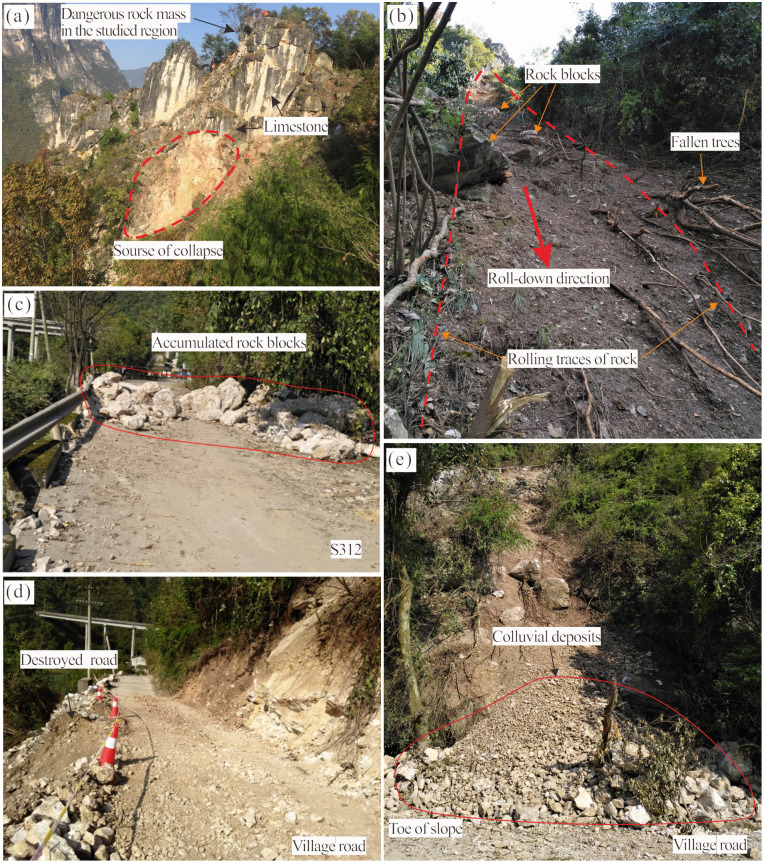
Detailed characteristics of the 2017 collapse event: (a) Location of the collapse source. (b) Rockfall trace. (c) Accumulation of rock blocks on the provincial highway (Maximum stone block 5.5 m^3^). (d) Damaged village road. (e) Colluvial deposit at the slope toe.

At 8:50 AM on October 15, 2017, a limestone rockfall occurred at the lower right section of the Heicao dangerous rock mass (Fig 5a), causing approximately 200 m^3^ of rock to collapse and accumulate near an elevation of 230 m. During the rockfall process, vegetation on the slope was destroyed, leaving a clearly visible rockfall traces. Additionally, several rock blocks of varying sizes were scattered across the slope, as shown in Fig 5b. Another portion of the rock blocks tumbled down and accumulated on Provincial Highway 312, with the largest fallen block measuring approximately 5.5 m^3^, while the remaining blocks ranged in volume from 0.01 to 1.50 m^3^ (Fig 5c). A significant amount of colluvium was deposited at the slope toe, which also resulted in damage to the village road (Fig 5d-e).

**Fig 6:**
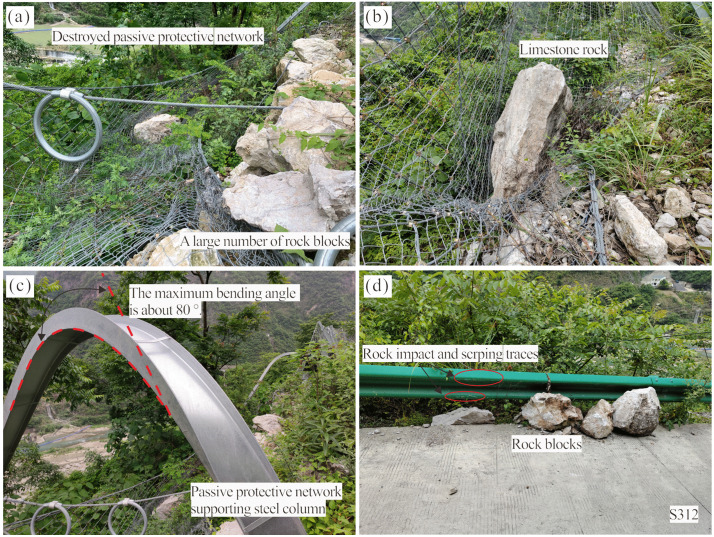
Detailed characteristics of the 2022 collapse event: (a-c) Specific conditions of the destroyed passive protective net (steel column bending 80°). (d) Rock blocks fallen onto the provincial highway (0.01-1.00 m^3^).

At approximately 1:00 PM on April 17, 2022, a rockfall event occurred on the northwest-facing cliff within the study area, involving a collapsed rock mass of approximately 50 m^3^. The majority of the rock blocks were intercepted by the passive protective net; however, about 40 meters of the net was destroyed, with its supporting steel columns exhibiting significant bending deformation, reaching a maximum bending angle of approximately 80°, as shown in Fig 6a-c. A minor portion of the rock blocks (volume ranging from approximately 0.01 ~ 1.00 m^3^) breached the passive protective net and scattered along the roadside of Provincial Highway 312. The highway guardrail exhibited distinct impact and scraping traces (Fig 6d).

## Method

### Setting of model

**Fig 7:**
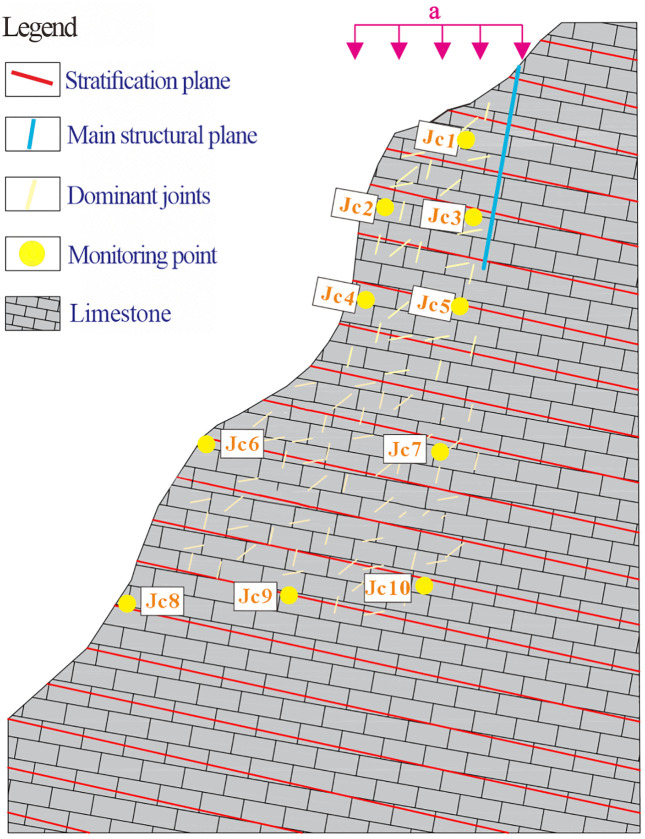
Schematic diagram of the numerical model for the Heicao rock mass (Dimension: width 60 m × height 90 m; number of blocks: 2879).

3DEC (3-Dimensional Distinct Element Code) is numerical modeling software based on the Discrete Element Method (DEM). It is mainly used to analyze the mechanical behavior of discontinuous media, such as jointed rock masses, under static or dynamic loads. In this study, the three-dimensional block discrete element method in 3DEC was applied to simulate the progressive failure of dangerous rock masses. The method was chosen because it can accurately capture the dynamic process from local damage to overall instability and is particularly suitable for slope collapse analysis. The 3DEC numerical model was constructed by extracting the relevant elevation section from the Heicao slope (Fig 3). This setup allowed cracks beneath the main structural plane to develop progressively into a through-going failure surface. The numerical model is depicted in Fig 7. The model spans 60 m in width and 90 m in height. The lengths of the main structural planes and the configuration of bedding surfaces were determined based on field investigation data. Within the dangerous rock mass zone, numerous randomly distributed dominant joints were incorporated, while the remaining regions were designated as bedrock matrix (S1 File).

Ten monitoring points were positioned within the numerical model to monitor key parameters such as rock displacement and velocity. The model comprised 2879 block elements. The bottom and right of model boundaries were fixed to simulate deep rock constraints, while vertical displacement was permitted to reduce artificial restrictions. The slope surface was set as a free boundary to represent actual topographic conditions. Failure of the Heicao dangerous rock mass was induced by applying an elevated gravitational acceleration of a = 40 m/s^2^. The model was considered failed if either of the following conditions was met: (1) the maximum unbalanced force dropped below 1 × 10⁻⁵; or (2) the slip surface was fully penetrated, and the maximum displacement exceeded the set threshold. All simulation data were systematically recorded throughout the failure process. The criterion of maximum unbalanced force below 1 × 10⁻⁵ is based on the static equilibrium requirement of the DEM. This threshold ensures sufficient convergence of contact forces while avoiding unnecessary computational costs. Similar criteria have been widely adopted in DEM-based slope stability analyses [35,36].

### Distribution of structural planes

**Fig 8:**
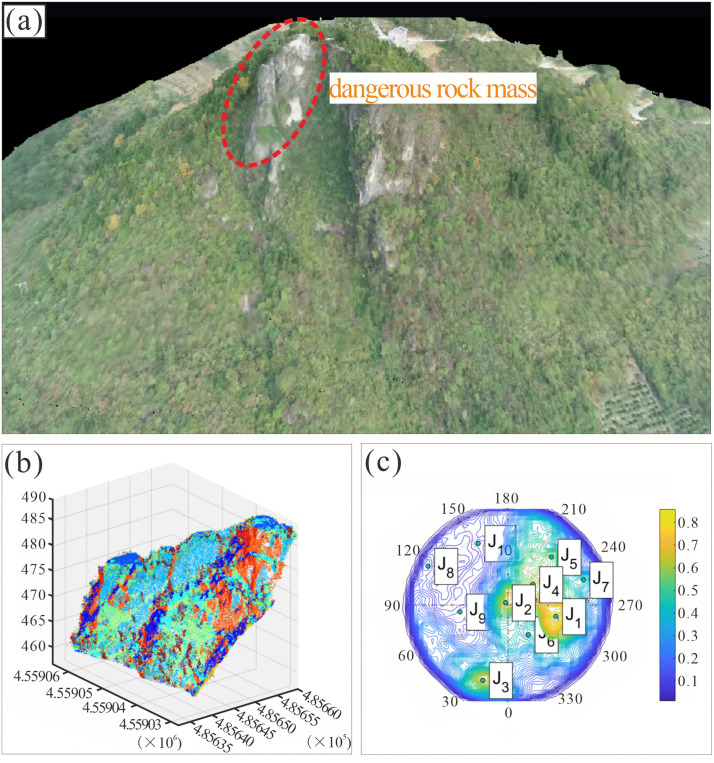
(a) Point cloud diagram of the Heicao slope. (b) Joint map of the dangerous rock mass. (c) Contoured pole density plot.

A 3D point cloud model of the Heicao slope was constructed using UAV oblique photogrammetry. The crest-area data were then processed using the K-means clustering algorithm [37,38] to extract structural planes (Fig 8).

DSE [39] a structural planes extraction software developed on the Matlab platform, was employed to process point cloud data from the target region (Fig 8a). Following parameter recommendations from prior studies [40], the local cluster number K was set to 30. The point cloud visualization and algorithmic identification results for the target region are shown in Fig 6b. In region (Fig 8b), the colors of some rock masses appear mixed, with multiple hues observed in the surrounding area. This is due to the long-term weathering in the dangerous rock zone has caused uneven fragmentation, while surface boulders from past rockfalls were sometimes misidentified as small structural planes during extraction. The extracted structural planes were cataloged and projected onto a Schmidt net, represented as a contoured pole density plot (Fig 8c). As illustrated in Fig 6c, three dominant joint sets were identified on the surface of the Heicao dangerous rock mass: J1 (dip direction 283.3 °,dip 54.36°), J2 (dip direction 200.44°,dip 83.29 °), and J3 (dip direction 222.14°,dip 67.95°). These dominant joints were subsequently randomly distributed within the numerical model (Fig 7).

### Mechanical parameters of structural planes

The numerical model consists of two parts: a constitutive model for rock blocks and another for structural planes. Rock blocks (i.e., the rock mass) were modeled using a Mohr-Coulomb formulation, while structural planes (e.g., bedding and joints) followed the Coulomb slip model. The mechanical parameters of the rock mass were calibrated based on laboratory tests of limestone specimens sampled from the Heicao dangerous rock mass, combined with empirical values for carbonate rocks provided in the Standard for Engineering Classification of Rock Masses (GB/T 50218–2014). The mechanical parameters of structural planes were determined from field geological surveys, laboratory tests on representative rock samples, and reference to the parameter ranges reported in previous studies on similar rock types and structural conditions [41–43]. The normal stiffness (Kn) values were calculated using the empirical relation Kn = 10 × E/d, where E is the elastic modulus of the rock mass and d is the joint spacing, while the tangential stiffness (Ks) can be taken as 1/3 of Kn. The parameters adopted in the numerical model are listed in Table 1.

**Table 1:** Mechanical parameters of the structural plane.

Structural Plane	Normal Stiffness	Tangential Stiffness	Tensile Strength	Cohesion	Friction Angle
	GPa/m	GPa/m	MPa	MPa	°
Bedding Plane	9.20	6.89	0.01	8.00	34.00
Joint Plane	4.60	3.40	0.01	0.10	20.00

## Progressive failure mechanisms

### Analysis of progressive failure characteristics

**Fig 9:**
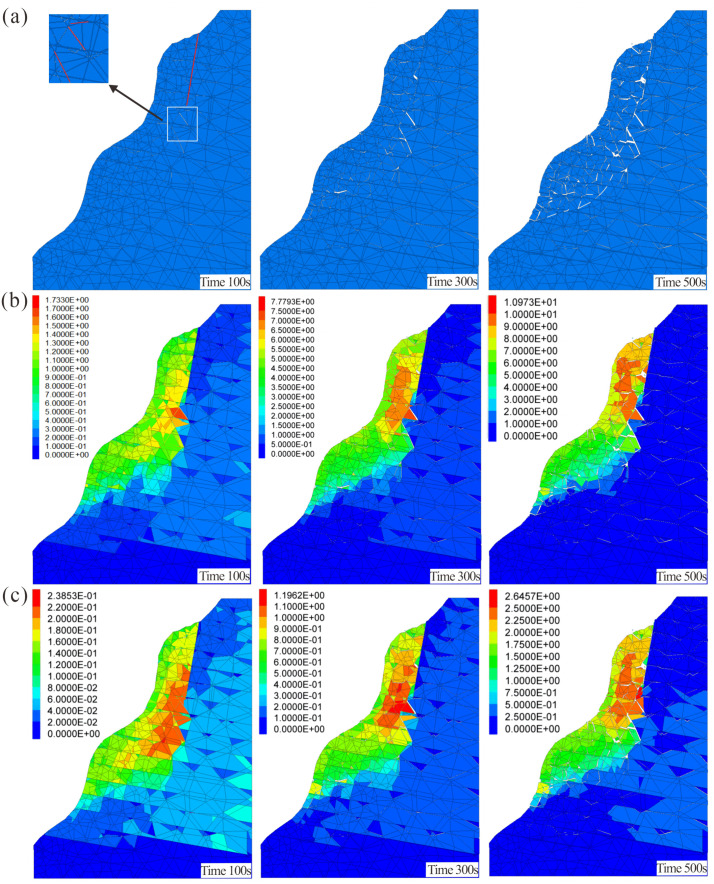
Cloud diagrams of the progressive failure process in the dangerous rock mass (the length of mainstructural plane is 30 m): (a) Crack development process. (b) Displacement field (maximum velocity 9.5 × 10^−3^ m/s). (c) Velocity field (maximum displacement 2.34 m).

As illustrated in the crack propagation cloud diagram (Fig 9a), the Heicao dangerous rock mass exhibited relatively limited crack development at 100 s, with only sporadic cracks emerging at the tips of the predefined main structural plane. This phenomenon resulted from stress concentration at the structural planes following the application of gravitational acceleration. By 300 s, interconnected cracks had formed within the influence zone of the main structural plane, segmenting the upper portion of the rock mass into discrete blocks. At this stage, the shear opening in the lower rock mass had not yet formed, although intermittent internal cracks were already developing due to stress buildup. At 500 s, the failure surface of the dangerous rock mass has already developed, with cracks predominantly propagating along the structural planes and extending downward. Concurrently, numerous cracks initiated in the compressive zone at the rock mass base, the resulting non-uniform deformation propagates upward. Polyline geometry cracks in the upper region gradually interconnected with randomly distributed cracks in the lower zone, forming a penetrating failure surface. Ultimately, sliding collapse occurred, with the failure surface in the lower rock mass exhibiting an arc-shaped profile. Carbonate rocks typically contain dissolution pores, fracture networks, and bedding planes, resulting in heterogeneous strength distribution. A brittle-to-ductile transition may occur in the lower rock mass. During progressive failure, stress initially concentrates in weakened zones, forming potential curved slip surfaces. Under external loads, tensile stress localizes near fracture tips while shear stress dominates in the lower rock mass. Ultimately, downward-propagating tensile cracks interconnect with developing shear slip zones, forming a steep-to-gentle arc-shaped failure surface.

The velocity cloud diagram of the dangerous rock mass (Fig 9b) reveals that during the initial simulation stage, no significant velocity variations were observed within the rock mass, with the maximum velocity localized near the main structural plane. From 300 s to 500 s, as time progressed, velocity gradients shifted from the interior to the slope surface. The upper portion of the dangerous rock mass gradually fragmented into discrete rock blocks, accompanied by a progressive increase in velocity, consistent with displacement trends. Progressive fragmentation at the shear opening caused rock blocks to detach from the bedrock. The resulting velocities exceeded those observed in the adjacent cracked regions. Subsequently, heterogeneous velocity distributions between the upper and lower portions of the rock mass induced a downward sliding tendency, marking the transition to incipient catastrophic failure.

According to the displacement cloud diagram of the dangerous rock mass (Fig 9c), at 100 s, the location of maximum displacement coincided with crack initiation trends, occurring at the tip of the main structural plane. cracks propagated from the tip of the structural plane toward the slope surface. By 300 s, displacement magnitudes within the rock mass continued to increase, with displacement at the structural plane tip transmitting downward. Simultaneously, relative displacements emerged in the compressive zone at the base of the dangerous rock mass, indicating that the lower rock mass was subjected to combined compression and shear. At 500 s, the tip of the structural plane still showed the greatest displacement. A through-going crack surface had formed there, resulting in a separated rock block. In the lower rock mass, the maximum displacement occurs at the shear opening, where rock fragmentation has resulted in the formation of falling blocks. At this stage, noticeable relative displacement has already appeared in the previously unbroken segment of the lower rock mass, with visible crack connections indicating failure of the rock bridges within the basal anti-sliding section.

#### Analysis of velocity variations.

The monitoring points in the numerical model were grouped and statistically analyzed, with monitoring points Jc1 ~ Jc5 located in the upper portion of the dangerous rock mass and Jc6 ~ Jc10 positioned in the lower portion.

**Fig 10:**
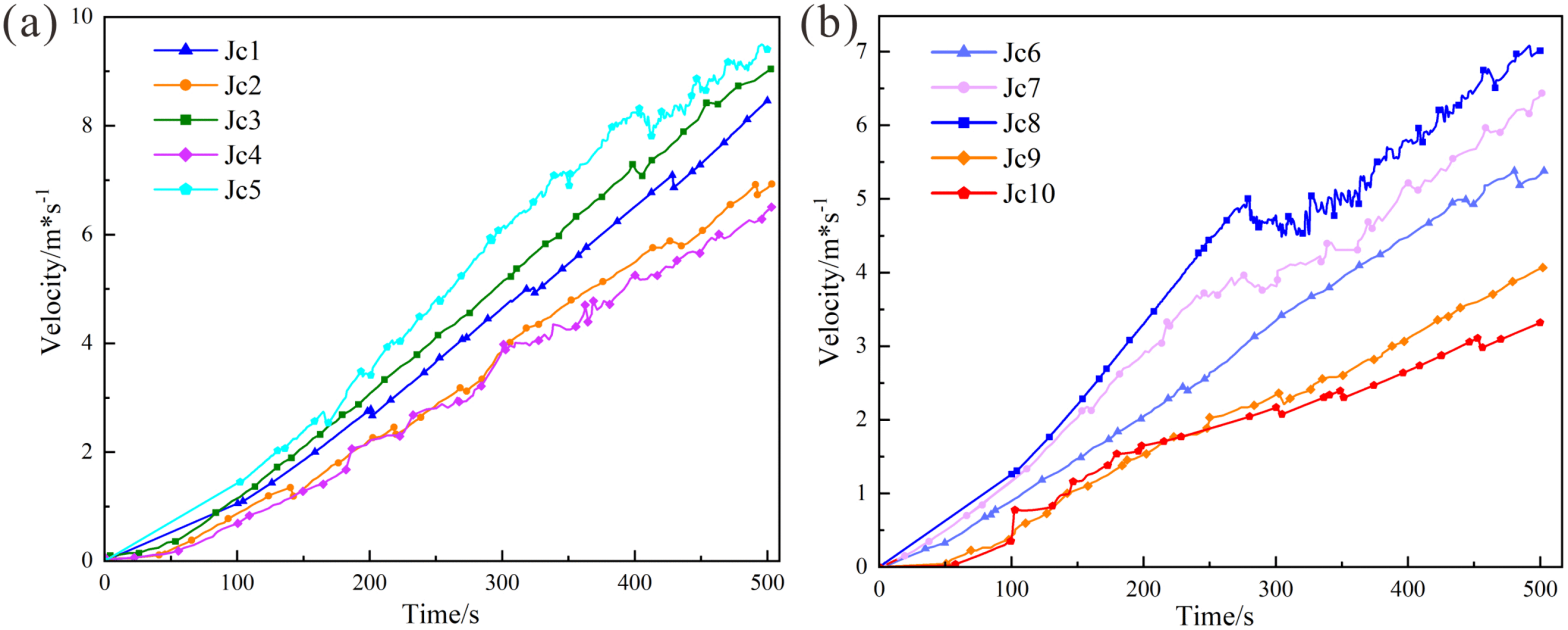
Velocity variation curves: (a) Velocity in the upper rock mass (Jc1-Jc5, maximum velocity 9.5 × 10^−3^ m/s). (b) Velocity in the lower rock mass (Jc6-Jc10, maximum velocity 7.3 × 10⁻^3^ m/s).

As shown in Fig 10a, the peak velocity in the upper rock mass occurred at monitoring point Jc5, with a magnitude of 9.5 × 10^−3^ m/s.The velocities at all upper monitoring points (Jc1 ~ Jc5) monotonically increased with time, indicating a coherent sliding motion of the upper rock mass with consistent kinematic behavior.

Fig 10b reveals that the maximum velocity in the lower rock mass was located at monitoring point Jc8, with a value of 7.3 × 10⁻^3^ m/s. Between 250 s and 500 s, the velocity curves for Jc7 and Jc8 showed an oscillatory upward trend, indicating that the rock blocks inside the slope were broken and collided with each other during this time period, and the rock mass was broken and fell off at the shear opening. The velocity trends for Jc9 and Jc10, located adjacent to the intact bedrock, there is almost no initial velocity. As cracks on the slope surface and in the upper rock mass gradually propagate and form a penetrating sliding surface, the velocity at Jc9 and Jc10 begins to increase slowly, although it remains the lowest among all monitoring points.

Comprehensive analysis indicates that with increasing time, the velocities at all monitoring points exhibit a general upward trend, although the rates of increase vary. Certain points displayed rapid acceleration, while others exhibited a more gradual increase. During the progressive failure process, multiple velocity peaks were observed, indicating that internal rock blocks have become detached from each other and that the contact surfaces between rock masses are cracked. Concurrently, collisions and friction between rock blocks during this process result in “oscillatory” fluctuations in the velocity curves.

#### Analysis of displacement variations.

**Fig 11:**
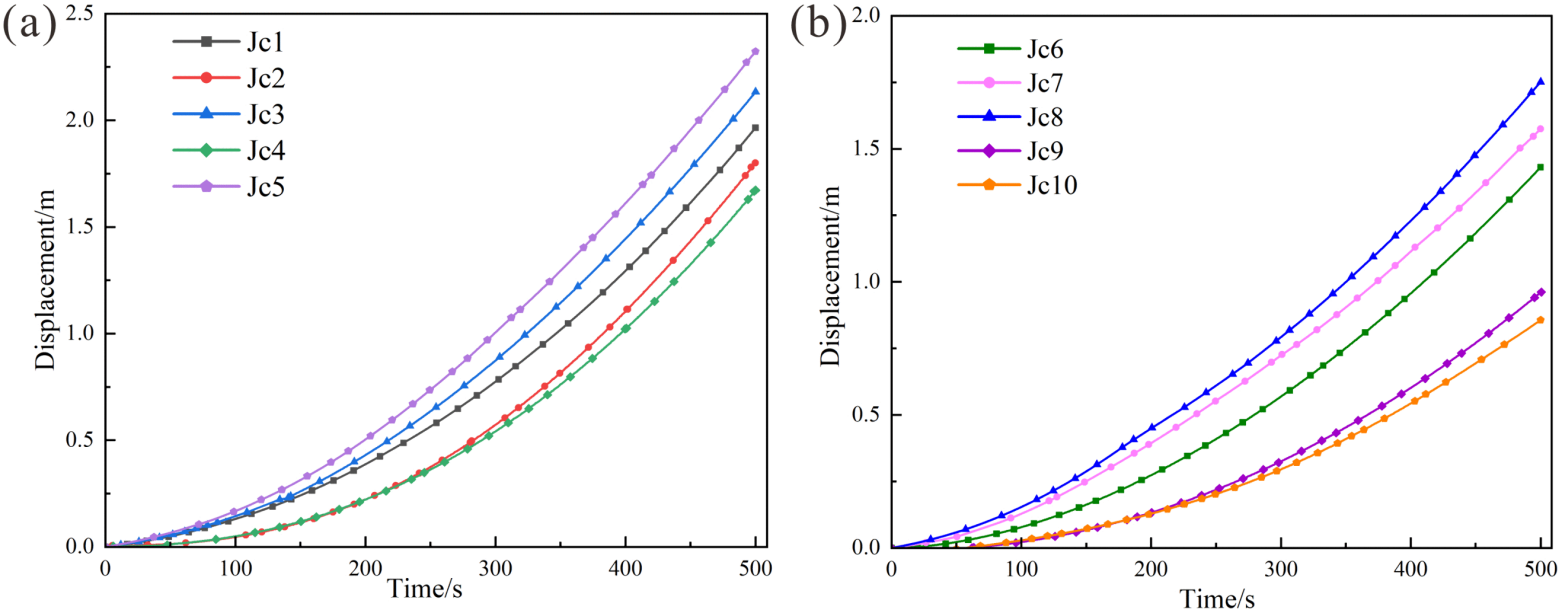
Displacement variation curves: (a) Displacement in the upper rock mass (Jc1-Jc5, maximum displacement 2.34 m). (b) Displacement in the lower rock mass (Jc6-Jc10, maximum displacement 1.77 m).

As shown in Fig 11a, the displacement magnitudes at the upper monitoring points of the Heicao dangerous rock mass increased progressively with time. When the model reached the predetermined failure threshold, the maximum displacement (2.34 m) was observed at monitoring point Jc5, located at the tip of the main structural plane. The maximum displacement and growth trend of monitoring point Jc5 are close to those of monitoring points Jc1 and Jc3, indicating that initial displacements originated around the main structural plane, with cracks propagating outward. Displacement trends at Jc2 and Jc4 exhibited similar growth patterns and comparable magnitudes, attributed to minor displacements in the slope surface rock mass. After 300 s, cracks generated from the main structural plane sequentially propagated to Jc2 and Jc4. Analysis of displacement trends at Jc1 ~ Jc5 confirms that the upper rock mass underwent whole sliding during failure.

Fig 11b demonstrates that displacement trends in the lower portion of the dangerous rock mass mirrored those in the upper region, with the maximum displacement (1.77 m) recorded at monitoring point Jc8, located at the shear opening in the lower portion of the dangerous rock mass. Cross-referencing with the displacement contour map, this peak displacement primarily resulted from the detachment of fragmented rock mass at the shear opening. Displacement trends at Jc6, Jc7, Jc9, and Jc10 were analogous, though magnitudes at Jc6 and Jc7 exceeded those at Jc9 and Jc10. This disparity reflects whole sliding and localized debris detachment in the lower rock mass, while Jc9 and Jc10, situated closer to the bedrock, exhibited similar displacements with bedrock. Comparative data at 500 s show significant displacement heterogeneity across the rock mass, with the lower portion exhibiting smaller displacements than the upper part. This suggests that the upper portion failured first under tensile stresses, while the lower region remained subjected to combined compressive-shear stresses until final failure.

#### Comparative analysis of numerical simulation results.

(1) Comparison of Failure Modes and Morphologies

Comparison of Failure Modes and Morphologies Numerical simulation results reveal that the failure surface of the Heicao rock mass exhibits a typical composite morphology: the upper section features zigzag tensile failure controlled by the main structural plane, while the following section displays an arc-shaped shear failure zone formed by compressive shear action (Fig 9a). This finding aligns closely with field investigations. UAV imagery (Fig 4a, b) clearly reveals a main tensile crack approximately 30 m long at the rear edge of the rock mass, with a dip direction of 310° and dip of 67°. Its opening decreases from 80 mm at the top to 10 mm at the bottom, consistent with the development characteristics of the upper fissure in the simulation. Concurrently, field investigations revealed highly developed base-level fractures at the rock mass foundation (Fig 4a), forming a relatively weak zone. This provides geological evidence for the formation of the lower compression-shear failure zone, explaining the origin of the lower arc-shaped failure surface observed in the simulation.

(2) Comparison of Movement Processes and Phenomena

Simulations revealed the sequential progression of progressive failure in dangerous rock masses: cracks spontaneously nucleate at the tips of controlling planes, propagate downward, and connect with lower random fissures, ultimately triggering rock mass sliding and collapse. This process aligns with historical disaster records. Both the 2017 and 2022 collapse events (Figs 5, 6) exhibited phenomena where, following upper rock mass instability, rock blocks rolled down the slope, collided, and accumulated at the toe and along the roadway. The “oscillatory” feature in the simulated velocity contour diagram (Fig 9b) and the sharp fluctuations at monitoring points Jc7 and Jc8 in the velocity-time curve (Fig 10) vividly reproduce the dynamic behavior of rock blocks colliding and fracturing during the collapse process.

(3) Quantitative Data Comparison

Quantitative data from monitoring points provide critical evidence for model validation. Simulation results indicate that maximum displacements occurred at the tip of the upper main structure plane (monitoring point Jc5, 2.34 m) and the lower shear opening (monitoring point Jc8, 1.77 m). Field measurements indicate that the rock mass at Jc5 exhibits greater fragmentation than that at Jc8, and both Jc5 and Jc8 show significantly higher fragmentation levels than other rock mass sections. This aligns with the locations of peak displacements identified in the numerical simulation. The maximum rear-edge tensile crack width at Jc8 was approximately 1 m. This result is quantitatively consistent with the numerical simulation value (2.34 m), with the discrepancy stemming from the field measurement being crack width while the simulation output represents displacement. Furthermore, the simulation results indicate that both displacement and velocity in the upper rock mass exceed those in the lower rock mass (Jc5 velocity: 9.5 × 10⁻^3^ m/s > Jc8 velocity: 7.3 × 10⁻^3^ m/s). This quantitatively reveals the instability mechanism of the dangerous rock mass, characterized by its “top-heavy” nature and uneven deformation, which is consistent with field observations.

### Failure analysis of varied structural planes

When the main structural plane propagates downward to a critical position, it reaches the failure path of the unconnected segment, ultimately forming a progressively penetrating failure surface. Accordingly, numerical models of the Heicao dangerous rock mass with varying lengths of main structural planes (10 m, 20 m, 30 m, and 40 m) were established. The 30 m scenario represents the actual geological conditions of Heicao dangerous rock mass, and its simulation results have been comprehensively detailed in the preceding section. Based on the characteristic monitoring point data from the actual Heicao dangerous rock mass conditions, four representative monitoring points were selected for quantitative analysis of the progressive failure process: Jc1 (slope crest), Jc5 (tip of the main structural plane/ mid-section of the rock mass), Jc8 (shear opening), and Jc10 (remote from the tip of the main structural plane).

**Fig 12:**
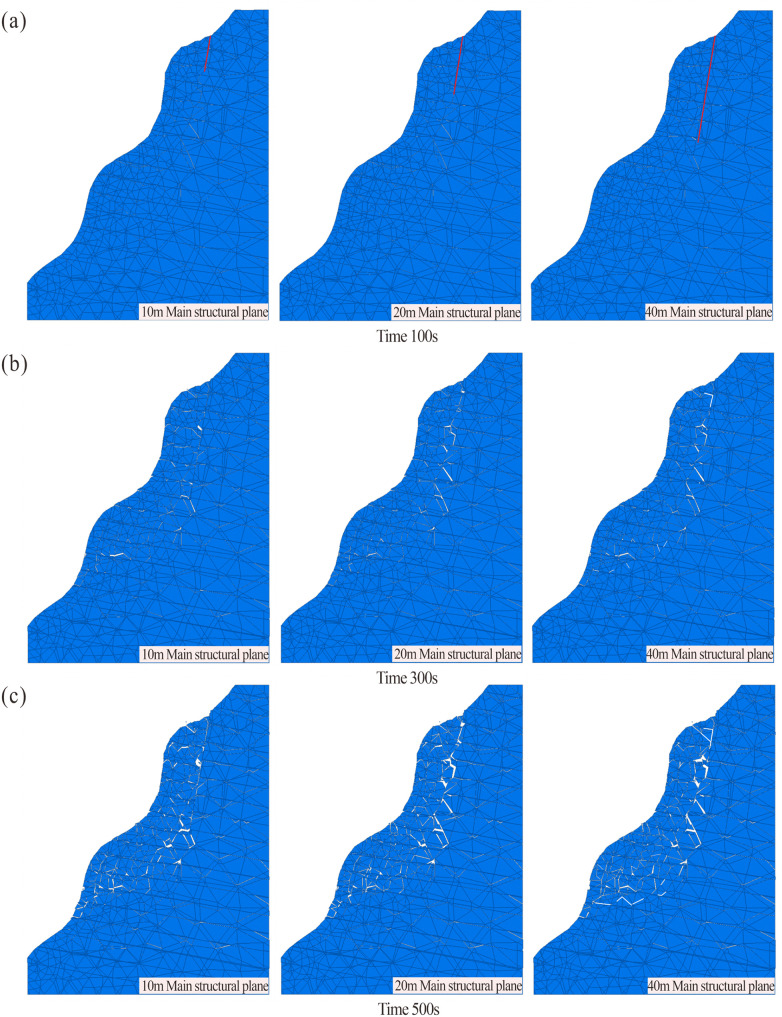
Crack Development of the dangerous rock mass with different structural plane lengths (10m, 20m, 40m structural plane length): (a) Development of 100s. (b) Development of 300s. (c) Development of 500s.

As illustrated in the crack propagation cloud diagram (Fig 12), at 100 s of simulation runtime, no significant crack development was observed in any numerical model under all working conditions (Fig 12a). During this initial stage, the rock mass primarily underwent compaction along structural planes under gravitational loading, with minimal or no new crack initiation. By 300 s (Fig 12b), varying numbers of cracks emerged across the all working conditions. As the length of the structural plane increased, the degree of rock fragmentation at the shear opening intensified, accelerating the formation of the shear failure surface. At 500 s (Fig 12c), the dangerous rock mass was segmented into multiple blocks by progressively developing cracks, leading to a sharp decline in overall stability. A progressively penetrating failure surface formed in all working conditions by this stage. The main structural planes with different lengths affect the overall development process of the dangerous rock mass. With increasing structural plane length, stress concentration at the tip facilitated rapid crack propagation toward the shear opening. Under identical timeframes, longer structural planes promoted crack initiation in the unconnected lower portions of the rock mass, thereby accelerating the instability of the dangerous rock mass.

**Fig 13:**
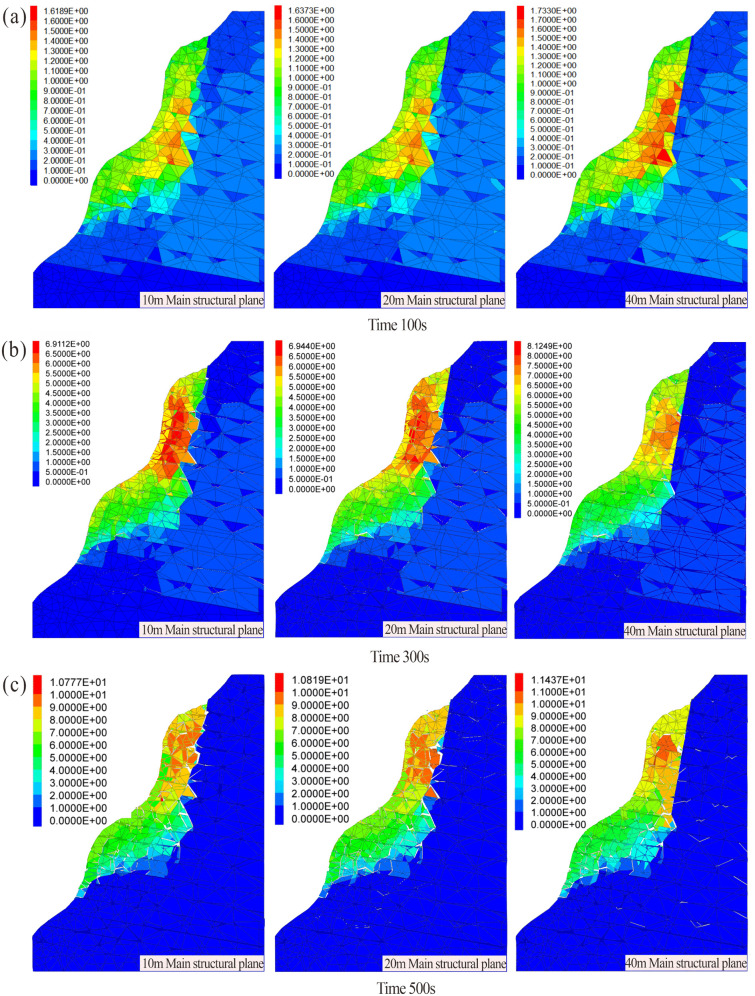
Velocity field of the dangerous rock mass with different structural plane lengths (10m, 20m, 40m structural plane length): (a) Development of 100s. (b) Development of 300s. (c) Development of 500s.

As shown in the velocity evolution field diagram (Fig 13), during the initial stage at 100 s (Fig 13a), the maximum velocities across all working conditions were relatively similar, indicating comparable overall rock mass strength and initial conditions. The velocity distribution aligned with crack propagation trends. Between 300 s and 500 s (Fig 13b,c), internal velocities gradually migrated toward the slope surface, with peak velocities localized in the upper portion of the dangerous rock mass. Under continuous development of the main structural plane, a progressively penetrating failure surface formed.For the 10 m main structural plane model, the maximum velocity increased from 6.91 × 10^−3^ m/s to 10.78 × 10^−3^ m/s, yielding a velocity increment of 3.87 × 10^−3^ m/s. Similarly, the 20 m model exhibited an increase from 6.94 × 10^−3^ m/s to 10.82 × 10^−3^ m/s, with an increment of 3.88 × 10^−3^ m/s. In contrast, the 40 m model showed a smaller velocity increment (3.34 × 10^−3^ m/s), rising from 8.12 × 10^−3^ m/s to 11.44 × 10^−3^ m/s. This reduced increment in the 40 m model is attributed to the internal fragmentation of the rock mass into blocks within the longer structural plane. The collisions among these blocks influence the overall velocity increment. Comparative analysis of velocity trends during failure revealed that the 40 m model consistently exhibited the highest peak velocities, demonstrating a positive correlation between structural plane length and rock mass failure velocity.

**Fig 14:**
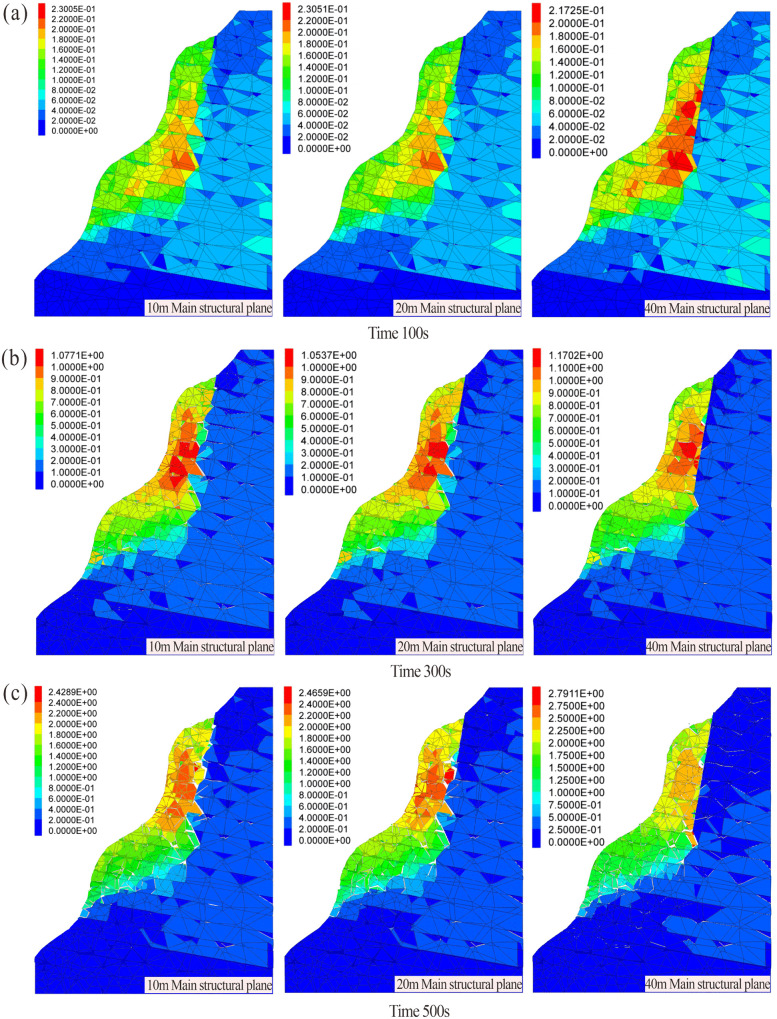
Displacement field of the dangerous rock mass with different structural plane lengths (10m, 20m, 40m structural plane length): (a) Development of 100s. (b) Development of 300s. (c) Development of 500s.

As shown in the displacement field distribution diagram (Fig 14), during the initial stage at 100 s (Fig 14a), the maximum displacements under each working conditions were 0.230 m, 0.231 m, 0.239 m, and 0.217 m, respectively, indicating comparable deformation ranges across all numerical models of the dangerous rock mass. This observation aligns with the characteristics of initial crack and displacement development. Between 300 s and 500 s (Fig 14b,c), significant deformation and failure occurred in the rock mass. The maximum displacement in the 10 m main structural plane model increased from 1.077 m to 2.429 m, yielding an increment of 1.352 m. Similarly, the 20 m model exhibited an increase from 1.054 m to 2.466 m (increment: 1.412 m), while the 40 m model showed a rise from 1.170 m to 2.791 m (increment: 1.621 m). The maximum displacement values and failure extent escalated with increasing length of the main structural plane, demonstrating a direct correlation between structural plane length and failure severity.

#### Analysis of velocity variations at monitoring points.

**Fig 15:**
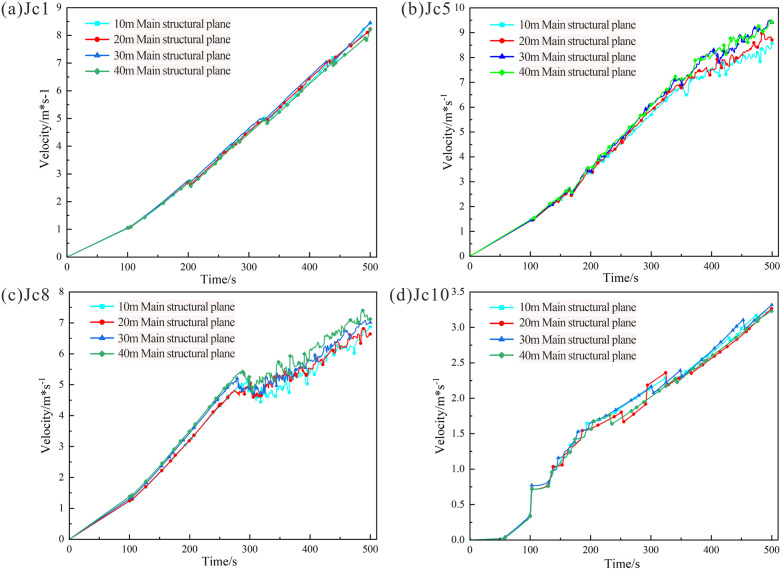
Velocity variation curves for monitoring points in rock masses with different structural planes: (a) Velocity at monitoring point Jc1 (slope crest). (b) Velocity at monitoring point Jc5 (mid-section of the rock mass). (c) Velocity at monitoring point Jc8 (shear opening). (d) Velocity at monitoring point Jc10 (remote from the tip of the main structural plane).

Fig 15a illustrates the velocity variation trend of the rock mass at the slope crest. Over the 0 ~ 500 s period, the velocity of the slope crest rock mass exhibited an overall linear increasing trend under all four working conditions, with consistent velocity variations across cases. The velocity at rock mass failure reached 8.5 × 10^−3^ m/s.

Fig 15b illustrates the velocity variation trends of the mid-section rock mass. During the initial phase (0 ~ 200 s), as evidenced by the cloud diagram, the velocity trends under all four working conditions were consistent. Between 200 s and 500 s, the rock mass entered an accelerated failure phase, during which the length of the structural plane significantly influenced failure velocity. The 30 m and 40 m main structural planes exhibited the most pronounced and similar velocity variations, while the 10 m structural plane showed the smallest changes. This disparity arises because the 10 m structural plane is farthest from the mid-section of the rock mass, resulting in the longest crack propagation path. The maximum velocity at rock mass failure reached 9.5 × 10^−3^ m/s.

Fig 15c illustrates the velocity variation trends of the rock mass at the shear opening. From the simulation start to 290 s, the velocity curves exhibited near-linear behavior. During this phase, the shear opening rock mass was primarily influenced by the cutting action of dominant joints and the gravitation from the upper rock mass, but no significant fragmented blocks had formed. From 290 s to 500 s, cracks gradually developed and interconnected within the shear opening rock mass across all working conditions, reducing rock mass strength and generating fragmented rock masses. This resulted in a sustained oscillatory phase in the velocity curves. Comparative analysis of the four working conditions revealed that while the 40 m scenario exhibited higher velocities than others, the shear opening rock mass was minimally influenced by the length of the main structural plane. The maximum velocity at rock mass failure reached 7.3 × 10^−3^ m/s.

Fig 15d displays the velocity variation trends of the rock mass remote from the tip of the main structural plane (Jc10). As Jc10 is located far from the structural plane tip, its velocity trends closely resembled those of the rock mass at the interface between the failure surface and bedrock. These trends were less affected by structural plane parameters, remaining consistent across all working conditions. The maximum velocity at rock mass failure reached 3.4 × 10^−3^ m/s.

Comprehensive analysis indicates that the peak velocities at the four monitoring points ranked as follows: Jc5 > Jc1 > Jc8 > Jc10. Velocities in the upper rock mass (Jc1 and Jc5) consistently exceeded those in the lower regions (Jc8 and Jc10), aligning with observations from the cloud diagrams. This confirms that crack propagation were more pronounced in the upper rock mass.

#### Analysis of displacement variations at monitoring points.

**Fig 16:**
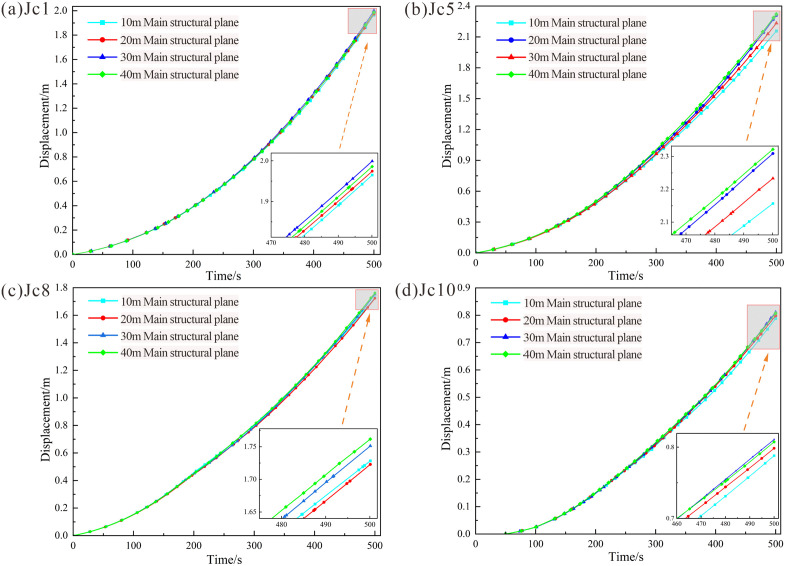
Displacement variation curves for monitoring points in rock masses with different structural planes: (a) Displacement at monitoring point Jc1 (slope crest). (b) Displacement at monitoring point Jc5 (mid-section of the rock mass). (c) Displacement at monitoring point Jc8 (shear opening). (d) Displacement at monitoring point Jc10 (remote from the tip of the main structural plane).

Analysis of displacement curves under the four working conditions (Fig 16) reveals that the displacement trends at monitoring point Jc1 (slope crest) were nearly identical across all cases, with negligible differences in magnitudes. The length of the main structural plane exhibited similar effects on displacements at the slope crest, where displacements reached 2.0 m at failure. Similarly, displacement trends at Jc5 (mid-section of the rock mass) were consistent. For the 10, 20, and 30 m conditions, failure displacement increased with the length of the main structural surface, reaching 2.32 m. The values for the 30 m and 40 m conditions were nearly identical. At Jc8 (shear opening) and Jc10 (remote region of the structural plane), displacement trends also aligned closely, as the structural plane length exerted minimal influence on these regions. Displacements at Jc8 and Jc10 during failure reached 1.77 m and 0.85 m, respectively, with minor variations across working conditions. Overall, the maximum displacements across the four monitoring points ranked as follows: Jc5 > Jc1 > Jc8 > Jc10, consistent with velocity curve trends. The velocities in the upper rock mass (Jc1 and Jc5) consistently exceeded those in the lower regions (Jc8 and Jc10), aligning with cloud diagram observations.

Combining the displacement cloud diagram and comparing the displacement and velocity curves shows that peak velocities and failure severity are positively correlated with the length of the main structural plane. The upper rock mass, more significantly influenced by structural plane length, exhibited higher velocities and displacements compared to the lower rock mass. The greater failure severity in the upper region stems from dominant stress redistribution and crack propagation, while the lower rock mass is primarily influenced by the cutting action of dominant joints and gravitational loading from the upper rock mass. However, downward crack propagation is hindered by rock bridges within the lower rock mass. Consequently, the main structure plane has less influence on the lower rock mass.

## Stability assessment

**Fig 17:**
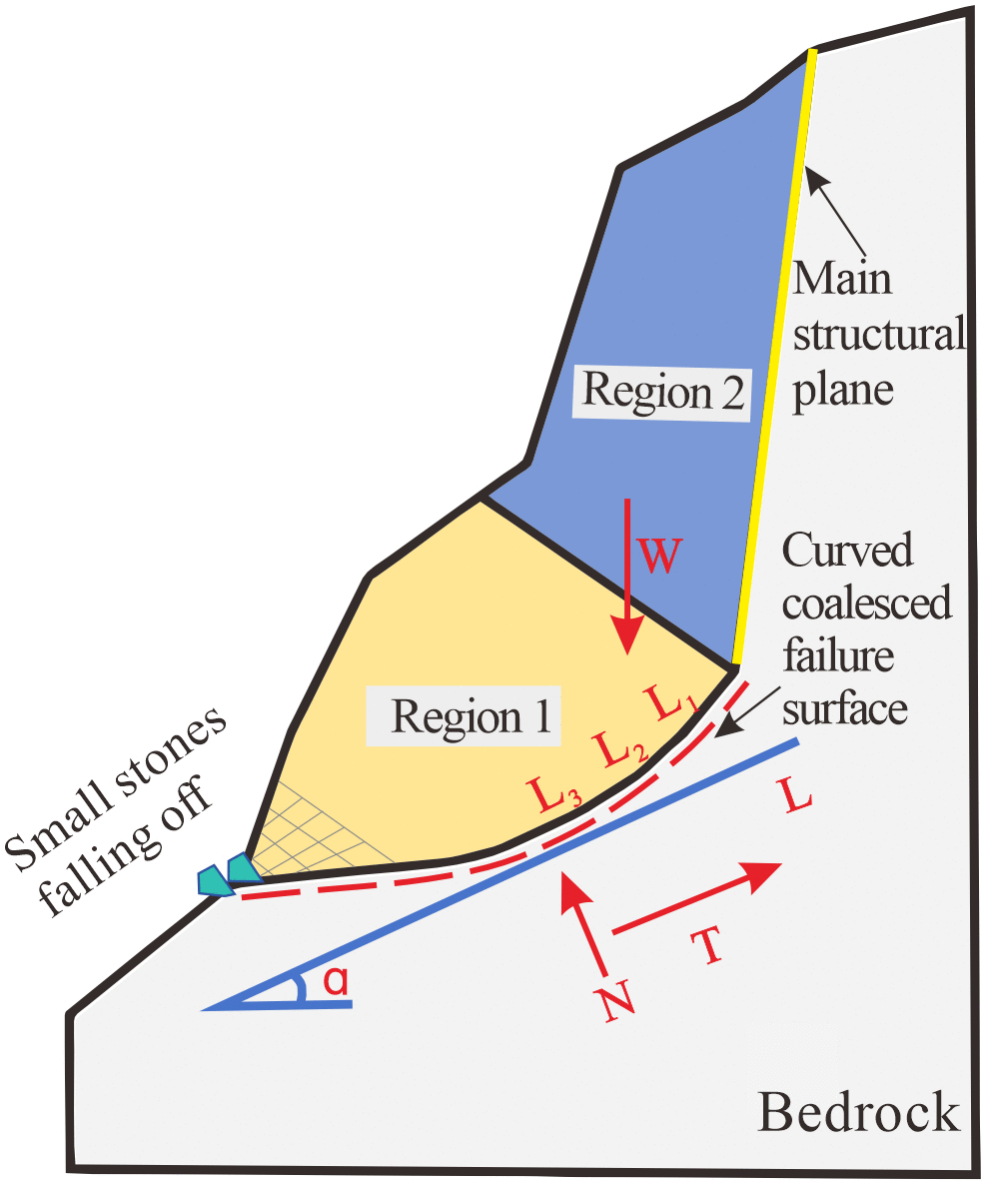
Mechanical model of dangerous rock mass instability in Heicao.

Simulation results show that the failure surface penetrates progressively due to the coalescence of the main structural plane and internal cracks under gravitational loading. The failure of the Heicao dangerous rock mass proceeds in two distinct stages. **Stage 1:** Under gravitational forces, cracks primarily propagate downward from the tip of the main structural plane. This causes the upper rock mass to move toward the free slope surface. The fractured rock blocks still exhibit overall coherent displacement. **Stage 2:** Once the main structural plane extends beyond a critical length, shallow cracks begin to form in the lower slope area due to gravity, weathering, and root wedging. These cracks weaken the compressive zone of the lower rock mass. This degradation reduces key mechanical parameters such as compressive strength, elastic modulus, and shear strength in the lower rock mass. This eventually causes arc-shaped penetration of the rock bridge part of the lower mass and a large amount of fragmentation of the rock mass at the shear opening and a small amount of rockfall formation. The mechanical model of this instability mechanism is illustrated in Fig 17.

(1) Region 1

Region 1 refers to the unconnected part of the dangerous rock mass. Two failure modes may occur here during the development of the main structural plane: compression-induced tensile cracking and shear failure.

When compression-induced tensile cracking occurs:Under unconfined or low-confined stress states, the crack surfaces generated by axial compression are generally aligned with or nearly parallel to the principal stress direction, indicating tensile failure of the rock mass. This process initiates when the maximum tensile strain reaches the ultimate tensile strain of the rock mass, resulting in tensile crack formation [44]. The following formula considers only the stress-strain relationship within the elastic deformation range of a continuous medium, adhering to Hooke’s Law. Assuming tensile failure initiates from the direction of the minimum principal stress (*σ*_*3*_) under uniaxial compression, let *ε*_*3*_ denote the strain in the *σ*_*3*_ -direction and ε_t_ represent the ultimate tensile strain. When compression-induced tensile cracking occurs, the failure criterion is expressed as:


$${E\varepsilon }_{\text{3}}=[\sigma_{\text{3}}-\mu (\sigma_{\text{1}}+\sigma_{\text{2}})]={E\varepsilon }_{t}$$ (1)

*E* denotes the elastic modulus; *σ*_*1*_, *σ*_*2*_, and *σ*_*3*_ represent the principal stresses; and *μ* is Poisson’s ratio.

Under uniaxial compression stress paths:


$$\sigma_{\text{3}}=\sigma_{\text{2}}=\text{0},\sigma_{\text{1}}=\sigma_{c}$$ (2)

*σ*_*c*_ is the compressive stress.

Substitute into formula (1) to get:


$$\varepsilon_{t}=-\frac{\mu }{E}\sigma_{c}$$ (3)

Rewritten to get:


$$[\sigma_{\text{3}}-\mu (\sigma_{\text{1}}+\sigma_{\text{2}})]={-\mu \sigma }_{c}$$ (4)

When σ_*2* _= σ_*3*_:


$$\sigma_{\text{1}}=\frac{\text{1}-\mu }{\mu }\sigma_{\text{1}}-\sigma_{\text{3}}$$ (5)

When shear failure occurs: The shear failure of the rock mass in Region 1 can be determined using the Mohr-Coulomb failure criterion, expressed as:


$$\sigma_{\text{1}}\geq \frac{(\text{1}+sin\varphi )+\text{2}C\cdot cos\varphi }{\text{1}-sin\varphi }$$ (6)

*C* denotes the cohesion of the rock, and *φ* represents the internal friction angle.

In summary, if the stress state in Region 1 meets Equation (5) or (6), the rock will fail accordingly, forming a failure surface in the unconnected zone.

(2) Region 2

Region 2 lies in the upper part of the rock mass, controlled by the main structural plane and supported from below by Region 1. Assuming zero supporting force from Region 1, the progressive failure surface comprises multiple discontinuous planes *L*_*(n)*_ that satisfy the Coulomb yield criterion, with dip angle α and horizontal projection lengths *L₁, L₂, …, L*ₙ*.*The total gravitational force is *W*. The stability coefficient *F*_*s*_ of the dangerous rock mass can then be expressed as:


$$F_{s}=\frac{Wcos\alpha tan\varphi +C(L_{\text{1}}+L_{\text{2}}+\cdot \cdot \cdot +L_{n})}{Wsin\alpha }$$ (7)

By expressing the length of the unconnected cracks in Equation (7) in terms of the projection length *L*, obtain:


$$F_{S}=\frac{Wcos\alpha tan\varphi +CL}{Wsin\alpha }$$ (8)

When the stability coefficient *F*_*s*_ < 1, a failure surface will form in the unconnected segment of the dangerous rock mass, leading to collapse hazards.

To quantitatively evaluate the stability of the Heicao dangerous rock mass, the stability coefficient *Fs* under various lengths of the main structural plane was calculated based on Equations (4–7) and (4–8), along with the physico-mechanical parameters provided in Table 1. The inclination angle of the failure surface *α* was set to 38° according to numerical simulation results. The projected length *L* of the penetrated segment under the 30 m main controlling structural plane was taken as 25 m, while other *L* values decreased as the length of the main structural plane increased. The calculation results are presented in Table 2.

**Table 2:** Stability coefficient at different lengths of the main structure plane.

Lengths of the Main Structure Plane (m)	10	20	30	40
Stability Coefficient (*Fs*)	1.38	1.20	1.06	0.92
State of stability	Stable	Generally stable	Less stable	Unstable

The results indicate that *Fs* exhibits a continuous decreasing trend with increasing length of the main controlling structural plane. When the structural plane length reaches 30 m or more, the stability coefficient falls below the critical value of 1.0, indicating that the rock mass becomes unstable. This finding is consistent with the numerical simulation results, demonstrating that longer structural planes accelerate the downward penetration of fractures and reduce the anti-sliding capacity of the base rock mass, ultimately leading to overall collapse.

In summary, the instability of the dangerous rock mass is jointly influenced by the basal rock mass in Region 1 and the overlying rock mass in Region 2. When the stress conditions satisfy: $\sigma_{\text{1}}=\frac{\text{1}-\mu }{\mu }\sigma_{\text{1}}+\sigma_{\text{3}}$ or $\sigma_{\text{1}}\geq \frac{(\text{1}+sin\varphi )+\text{2}C\cdot cos\varphi }{\text{1}-sin\varphi }$, a progressively penetrating failure surface will develop in the unconnected segment (Region 1), leading to compression-induced tensile cracking or shear failure. Additionally, when the stability coefficient $F_{S}=\frac{Wcos\alpha tan\varphi +CL}{Wsin\alpha }$ is less than 1, a progressively penetrating failure surface will also develop in the unconnected segment (Region 1), leading to collapse hazards. The mechanism of collapse can be summarized as follows: under gravitational loading, gradual degradation of the rock mass in Region 1 generates cracks, progressively weakening the anti-sliding force provided to Region 2. Concurrently, the main structural plane extends further, which exacerbates the destruction of the rock mass in Region 1, and the cycle repeats itself. Ultimately, crack coalescence and rock bridge shearing in Region 1 lead to the formation of a penetrating failure surface, triggering overall instability. Therefore, the basal rock mass in Region 1 is essential for maintaining the overall stability of the slope. Implementing effective protective measures for this foundation is crucial to preventing the development of dangerous rock mass hazards.

## Discussion

### Research methods

This study integrates UAV oblique photogrammetry with 3DEC numerical modeling, overcoming the limitations of traditional survey methods in steep terrain. Compared with laser scanning [15–19] and close-range photogrammetry [24], UAVs greatly improve efficiency in vegetated and high-slope areas, though fracture identification accuracy remains limited. Future work may incorporate deep learning to enhance image interpretation. The 3DEC model reproduced the progressive failure process, but did not consider long-term weathering effects. Future research should focus on multi-field coupled models to achieve more realistic simulations.

### Mechanical mechanism of progressive failure

Field observations and numerical simulations together clarify the progressive failure mechanism of the Heicao rock mass. Results show that gravity connects structural planes and internal fractures, forming a continuous failure surface, consistent with previous studies on the roles of structural planes and rock bridges [5–8]. The failure process occurs in two stages. First, cracks concentrate at the tip of the main structural plane, causing tensile failure and outward movement of the upper rock mass with small displacement differences. Second, shallow fractures in the lower slope develop under gravity, weathering, and root wedging, reducing the basal rock’s strength and forming an arcuate failure surface with fragmentation at the shear outlet. This evolution indicates that upper tensile failure and lower compressive-shear degradation jointly control the process. Moreover, the length of the main structural plane strongly influences failure intensity. A longer plane accelerates crack propagation and increases peak velocity in the upper slope by reducing effective rock bridge length and concentrating stress. In contrast, its influence on the lower slope is weaker, likely due to intact rock bridges that hinder downward crack growth.

### Limitations and future directions

Although this study clarified the progressive failure mechanism of the Heicao dangerous rock mass, certain limitations remain. The model did not consider long-term environmental effects such as rainfall and freeze–thaw cycles, and the analysis would benefit from integrating in situ tests with numerical simulations. Future work should therefore focus on: (1) developing multi-field coupled models that account for hydro–mechanical–chemical interactions; (2) combining field monitoring methods (e.g., microseismic and acoustic emission) with simulations; and (3) applying artificial intelligence to stability prediction. These efforts will improve the accuracy of early-warning systems and strengthen the theoretical basis for hazard prevention.

## Conclusions

A large number of structural planes have developed within the Heicao dangerous rock mass, where the main structural plane governs the initial crack propagation direction. Meanwhile, the randomly distributed dominant joints also influence the failure process of the rock mass This investigation into the failure process of the Heicao dangerous rock mass yields the following conclusions.

First, the failure surface of the Heicao dangerous rock mass formed through progressive coalescence, exhibiting a polyline geometry in the upper section and an arc-shaped profile in the lower section,which is a characteristic composite form.There is a general trend of increasing velocities and displacements at the various monitoring points over time. Inter-block collisions and friction caused oscillatory fluctuations in the velocity-time curves during the failure process. Significant displacement heterogeneity is observed between the upper and lower rock masses, tensile failure dominates the upper region due to crack initiation at the tip of the main structural plane, whereas the lower region undergoes sustained compressive-shear failure.

Analysis of the failure process in rock mass models with varying structural plane lengths reveals that the peak velocity and failure severity during rock mass failure exhibit a positive correlation with the length of the main structural plane. Specifically, the effect of structural plane length is stronger in the upper rock mass, while the lower part is less affected. Furthermore, the upper rock mass generally outperforms the lower rock mass in terms of velocity and displacement, and the upper rock mass is more severely damaged.

Finally, the instability of the dangerous rock mass is jointly influenced by the basal rock mass in Region 1 and the overlying rock mass in Region 2. When the stress conditions satisfy: $\sigma_{\text{1}}=\frac{\text{1}-\mu }{\mu }\sigma_{\text{1}}+\sigma_{\text{3}}$ or $\sigma_{\text{1}}\geq \frac{(\text{1}+sin\varphi )+\text{2}C\cdot cos\varphi }{\text{1}-sin\varphi }$, a progressively penetrating failure surface will develop in the unconnected segment (Region 1), leading to compression-induced tensile cracking or shear failure. When the stability coefficient $F_{S}=\frac{Wcos\alpha tan\varphi +CL}{Wsin\alpha }$ is less than 1,a progressively penetrating failure surface will also develop in the unconnected segment (Region 1), leading to collapse hazards. From this, the collapse mechanism can be summarized as follows: under gravitational loading, progressive degradation of the rock mass in Region 1 generates cracks, gradually weakening the anti-sliding force provided to Region 2. Under gravitational loading, progressive degradation of the rock mass in Region 1 generates cracks, gradually weakening the anti-sliding force provided to Region 2. Concurrently, the main structural plane extends further,which exacerbates the destruction of the rock mass in Region 1, and the cycle repeats itself. Ultimately, crack coalescence and rock bridge shearing in Region 1 lead to the formation of a penetrating failure surface, triggering overall instability.

As the above shows, this study provides important theoretical and practical insights for geological hazard assessment and prevention. The quantitative link between structural plane length and failure velocity improves risk evaluation, while the two-stage “tensile–shear” mechanism supports the development of more accurate early-warning models. For mitigation, protecting the basal rock mass is crucial, and combining UAV monitoring with numerical simulation offers a standardized approach for slope monitoring. Future work should extend this framework to multi-factor conditions, such as earthquakes and rainfall, to provide a more comprehensive basis for hazard prevention.

## Supporting information

S1 File Discrete element code of different working conditions. (DOCX)
