# Supplementary material for: Progressive failure mechanism and stability assessment of high-steep dangerous rock mass: A case study of Heicao dangerous rock mass
Source: PLoS One. 2025 Nov 10;20(11):e0336115. doi: 10.1371/journal.pone.0336115 (PMC12599965; doi:10.1371/journal.pone.0336115)
Supplement: S1 File — (DOCX) [file pone.0336115.s001.docx]

**Discrete element code of different working conditions**

**Crack propagation cloud diagram condition**

res model1.sav

hide range plane dip 0 dd 90 origin 822,733,421 below ; 411-40m 421-30m 431-20m,441-10m

jset dip 100 dd 90 origin 822,733,421

seek

hide range plane dip 120 dd 90 origin 796,732,391 above

jset dip -18 dd 90 origin 796,732,390

seek

hide range plane dip 100 dd 90 origin 822,733,421 above

hide range plane dip -20 dd 90 origin 796,732,391 below

group BLOCK bt range Z -10 500

seek

jset dip 10 dd 90 spac 5 num 27 ori 827,733,430 id 3

;join on

;gen edge 3

save model2dsav

**Displacement cloud diagram condition**

res model2dsav

fix range z 362 374

bound yvel 0 range z 0 500

bound yvel 0 zvel 0 range y 731.153

bound yvel 0 zvel 0 range y 742.958

bound xvel 0 yvel 0 zvel 0 range x 839 839.5

change con 2

change jcon 1

change mat 2 range group bt

change jmat=2 range dip 100 dd 90

change jmat=3 range dip -20 dd 90

change jmat=4 range dip 10 dd 90

prop mat=1 dens=25000 k=59.250e99 g=49.277e99

prop mat=2 dens=87500 k=45.250e9 g=35.277e9

prop jmat=1 jkn=1e12 jks=1e12 jfric=20

prop jmat=2 jkn=2e1 jks=2e1 jfric=30

prop jmat=3 jkn=2e9 jks=2e1 jfric=30

prop jmat=4 jkn=2e1 jks=2e1 jfric=30

;bound stress -9e6,-9e6,0 0,0,0 range group bt

;bound stress -9e6,0,0 0,0,0 range group bt

bound yvel 0 range z 0 500

grav 0,0,-40

hist unbal

step 1000

reset disp time hist

;solve fos

;bound stress -9e19,0,-9e10 0,0,0 range group bt

plot cut add plane origin (800,736,374) name Plane normal (0,1,0)

plot contour displacement plane onplane wireframe off

history id 55 vel (811,736,434)

history id 66 disp (811,736,434)

his dump 55 file er1.txt

his dump 66 file er2.txt

hist id 1 vel (811,736,434)

hist id 2 displacement (811,736,434)

hist id 3 vel (821,736,427)

hist id 4 displacement (821,736,427)

hist id 5 vel (811,736,420)

hist id 6 displacement (811,736,420)

hist id 7 vel (821,736,420)

hist id 8 displacement (821,736,420)

hist id 9 vel (793,736,405)

hist id 10 displacement (793,736,405)

hist id 11 vel (800,736,405)

hist id 12 displacement (800,736,405)

hist id 13 vel (815,736,405)

hist id 14 displacement (815,736,405)

hist id 15 vel (821,736,434)

hist id 16 displacement (811,736,434)

hist id 17 vel (793,736,405)

hist id 18 displacement (793,736,390)

hist id 19 vel (800,736,405)

hist id 20 displacement (800,736,390)

hist id 21 vel (815,736,405)

hist id 22 displacement (815,736,390)

hist id 23 vel (811,736,427)

hist id 24 displacement (811,736,427)

his dump 1 3 5 7 9 11 13 15 17 19 21 23 skip 10 begin 1 end 6500 file jcsudu.txt

his dump 2 4 6 8 10 12 14 16 18 20 22 24 skip 10 begin 1 end 6500 file jcweiyi.txt

his dump 20 file er.txt

his dump 24 file er.txt

set hist_rep 1

;plot create

;plot clear

;plot add contour displacement wireframe on width 1 color black

;plot add contour vel wireframe on width 1 color black

;plot cut add plane origin (800,736,374) name Plane normal (0,1,0)

;plot contour displacement plane onplane wireframe off

;;plot set projection perspective magnification 1 center (156.637,9.80187,112.5) eye (-156.637,-517.655,112.5) vertical (90,180,360)

;plot set movieactive on

;plot set movieextension png

;plot set movieinterval 500

;plot set movieprefix new_

;plot set moviesize 1660 1080

;reset disp time hist

fix range group 10001

bound yvel 0 range z 0 500

bound yvel 0 zvel 0 range y 731.153

bound yvel 0 zvel 0 range y 742.958

bound xvel 0 yvel 0 zvel 0 range x 839 839.5

change con 2

change jcon 1

change mat 2 range group bt

change jmat=2 range dip 100 dd 90

change jmat=3 range dip -20 dd 90

prop mat=1 dens=25000 k=59.250e99 g=49.277e99

prop mat=2 dens=87500 k=45.250e9 g=35.277e9

prop jmat=1 jkn=1e12 jks=1e12 jfric=20

prop jmat=2 jkn=2 jks=2 jfric=0

prop jmat=3 jkn=2 jks=2 jfric=0

;

;

;bound stress -9e6,-9e6,0 0,0,0 range group bt

;bound stress -9e6,0,0 0,0,0 range group bt

;

bound yvel 0 range z 0 500

grav -10,0,-40

;bound stress -9e19,0,-9e10 0,0,0 range group bt

;hist vel (811,736,434)

;hist displacement (811,736,434)

;

;hist vel (821,736,434)

;hist displacement (821,736,434)

;

;hist vel (811,736,427)

;hist displacement (811,736,427)

;

;hist vel (821,736,427)

;hist displacement (821,736,427)

;

;hist vel (811,736,420)

;hist displacement (811,736,420)

;

;hist vel (821,736,420)

;hist displacement (821,736,420)

;

;hist vel (793,736,405)

;hist displacement (793,736,405)

;

;hist vel (800,736,405)

;hist displacement (800,736,405)

;

;hist vel (815,736,405)

;hist displacement (815,736,405)

;

;Hist vel (793,736,405)

;hist displacement (793,736,390)

;

;hist vel (800,736,405)

;hist displacement (800,736,390)

;

;hist vel (815,736,405)

;hist displacement (815,736,390)

;

;history id 811 vel (811,736,434)

;history id 8112 disp (811,736,434)

;his dump 811 file 811.txt

;his dump 8112 file 8112.txt

;

;

;

;;plot create

;;plot clear

;;;plot add contour displacement wireframe on width 1 color black

;;;plot add contour vel wireframe on width 1 color black

;;plot cut add plane origin (800,736,374) name Plane normal (0,1,0)

;;plot contour displacement plane onplane wireframe off

;;;plot set projection perspective magnification 1 center (156.637,9.80187,112.5) eye (-156.637,-517.655,112.5) vertical (90,180,360)

;;plot set movieactive on

;;plot set movieextension png

;;plot set movieinterval 500

;;plot set movieprefix new_

;;plot set moviesize 1660 1080

**Velocity cloud diagram condition**

new

set atol 3.67010332978382e-02

set echo suppress

; block 1

poly group "10001" mat 1 con 1 &

face id 2 &

8.29251774542374e+02 7.34873958371039e+02 4.43856971841759e+02 &

8.28516744472485e+02 7.39206897844698e+02 4.40026719289599e+02 &

8.27910339647761e+02 7.39308812863053e+02 4.44974237053554e+02 &

face id 2 &

8.28516744472485e+02 7.39206897844698e+02 4.40026719289599e+02 &

8.29251774542374e+02 7.34873958371039e+02 4.43856971841759e+02 &

8.31846429871947e+02 7.39228897154382e+02 4.43461849917398e+02 &

face id 2 &

8.27910339647761e+02 7.39308812863053e+02 4.44974237053554e+02 &

8.28516744472485e+02 7.39206897844698e+02 4.40026719289599e+02 &

8.31846429871947e+02 7.39228897154382e+02 4.43461849917398e+02 &

face id 2 &

8.29251774542374e+02 7.34873958371039e+02 4.43856971841759e+02 &

8.27910339647761e+02 7.39308812863053e+02 4.44974237053554e+02 &

8.31846429871947e+02 7.39228897154382e+02 4.43461849917398e+02

; block 2

poly group "10001" mat 1 con 1 &

face id 2 &

8.35759803702102e+02 7.42957763671875e+02 4.49303153342892e+02 &

8.35849131295671e+02 7.40452496287849e+02 4.52089202880859e+02 &

8.39084289550781e+02 7.42957763671875e+02 4.52089202880859e+02 &

face id 2 &

8.35849131295671e+02 7.40452496287849e+02 4.52089202880859e+02 &

8.35759803702102e+02 7.42957763671875e+02 4.49303153342892e+02 &

8.34390460238686e+02 7.42957763671875e+02 4.52089202880859e+02 &

face id 1 &

8.39084289550781e+02 7.42957763671875e+02 4.52089202880859e+02 &

8.35849131295671e+02 7.40452496287849e+02 4.52089202880859e+02 &

8.34390460238686e+02 7.42957763671875e+02 4.52089202880859e+02 &

face id 1 &

8.35759803702102e+02 7.42957763671875e+02 4.49303153342892e+02 &

8.39084289550781e+02 7.42957763671875e+02 4.52089202880859e+02 &

8.34390460238686e+02 7.42957763671875e+02 4.52089202880859e+02

; block 3

poly group "10001" mat 1 con 1 &

face id 2 &

8.28706483747381e+02 7.39357681581388e+02 4.34620473496206e+02 &

8.23877178682653e+02 7.39381023327622e+02 4.36180865465919e+02 &

8.26210126105588e+02 7.35272944122540e+02 4.33492780179440e+02 &

face id 2 &

8.23877178682653e+02 7.39381023327622e+02 4.36180865465919e+02 &

8.28706483747381e+02 7.39357681581388e+02 4.34620473496206e+02 &

8.26011586687483e+02 7.37674958461435e+02 4.37842021451426e+02 &

face id 2 &

8.26210126105588e+02 7.35272944122540e+02 4.33492780179440e+02 &

8.23877178682653e+02 7.39381023327622e+02 4.36180865465919e+02 &

8.26011586687483e+02 7.37674958461435e+02 4.37842021451426e+02 &

face id 2 &

8.28706483747381e+02 7.39357681581388e+02 4.34620473496206e+02 &

8.26210126105588e+02 7.35272944122540e+02 4.33492780179440e+02 &

8.26011586687483e+02 7.37674958461435e+02 4.37842021451426e+02

; block 4

poly group "10001" mat 1 con 1 &

face id 2 &

8.24231497582248e+02 7.39555545391830e+02 4.40655328606252e+02 &

8.28516744472485e+02 7.39206897844698e+02 4.40026719289599e+02 &

8.26114508878027e+02 7.34878405664679e+02 4.40774789501596e+02 &

face id 2 &

8.28516744472485e+02 7.39206897844698e+02 4.40026719289599e+02 &

8.24231497582248e+02 7.39555545391830e+02 4.40655328606252e+02 &

8.26011586687483e+02 7.37674958461435e+02 4.37842021451426e+02 &

face id 2 &

8.26114508878027e+02 7.34878405664679e+02 4.40774789501596e+02 &

8.28516744472485e+02 7.39206897844698e+02 4.40026719289599e+02 &

8.26011586687483e+02 7.37674958461435e+02 4.37842021451426e+02 &

face id 2 &

8.24231497582248e+02 7.39555545391830e+02 4.40655328606252e+02 &

8.26114508878027e+02 7.34878405664679e+02 4.40774789501596e+02 &

8.26011586687483e+02 7.37674958461435e+02 4.37842021451426e+02

; block 5

poly group "10001" mat 1 con 1 &

face id 2 &

8.28516744472485e+02 7.39206897844698e+02 4.40026719289599e+02 &

8.30150338633184e+02 7.35459866734055e+02 4.38544963342445e+02 &

8.26114508878027e+02 7.34878405664679e+02 4.40774789501596e+02 &

face id 2 &

8.30150338633184e+02 7.35459866734055e+02 4.38544963342445e+02 &

8.28516744472485e+02 7.39206897844698e+02 4.40026719289599e+02 &

8.26011586687483e+02 7.37674958461435e+02 4.37842021451426e+02 &

face id 2 &

8.26114508878027e+02 7.34878405664679e+02 4.40774789501596e+02 &

8.30150338633184e+02 7.35459866734055e+02 4.38544963342445e+02 &

8.26011586687483e+02 7.37674958461435e+02 4.37842021451426e+02 &

face id 2 &

8.28516744472485e+02 7.39206897844698e+02 4.40026719289599e+02 &

8.26114508878027e+02 7.34878405664679e+02 4.40774789501596e+02 &

8.26011586687483e+02 7.37674958461435e+02 4.37842021451426e+02

; block 6

poly group "10001" mat 1 con 1 &

face id 1 &

7.93403327531912e+02 7.31153198242188e+02 3.64848876953125e+02 &

7.96608477764343e+02 7.37040518562079e+02 3.64848876953125e+02 &

7.90958430310048e+02 7.37028537924020e+02 3.64848876953125e+02 &

face id 2 &

7.96608477764343e+02 7.37040518562079e+02 3.64848876953125e+02 &

7.93403327531912e+02 7.31153198242188e+02 3.64848876953125e+02 &

7.93658752091436e+02 7.34748621680107e+02 3.69706280758364e+02 &

face id 2 &

7.90958430310048e+02 7.37028537924020e+02 3.64848876953125e+02 &

7.96608477764343e+02 7.37040518562079e+02 3.64848876953125e+02 &

7.93658752091436e+02 7.34748621680107e+02 3.69706280758364e+02 &

face id 2 &

7.93403327531912e+02 7.31153198242188e+02 3.64848876953125e+02 &

7.90958430310048e+02 7.37028537924020e+02 3.64848876953125e+02 &

7.93658752091436e+02 7.34748621680107e+02 3.69706280758364e+02

; block 7

poly group "10001" mat 1 con 1 &

face id 2 &

7.89220069374438e+02 7.39019972726502e+02 3.69753861949652e+02 &

7.93658752091436e+02 7.34748621680107e+02 3.69706280758364e+02 &

7.90958430310048e+02 7.37028537924020e+02 3.64848876953125e+02 &

face id 2 &

7.93658752091436e+02 7.34748621680107e+02 3.69706280758364e+02 &

7.89220069374438e+02 7.39019972726502e+02 3.69753861949652e+02 &

7.94048121019450e+02 7.39434762152529e+02 3.69924562422854e+02 &

face id 2 &

7.90958430310048e+02 7.37028537924020e+02 3.64848876953125e+02 &

7.93658752091436e+02 7.34748621680107e+02 3.69706280758364e+02 &

7.94048121019450e+02 7.39434762152529e+02 3.69924562422854e+02 &

face id 2 &

7.89220069374438e+02 7.39019972726502e+02 3.69753861949652e+02 &

7.90958430310048e+02 7.37028537924020e+02 3.64848876953125e+02 &

7.94048121019450e+02 7.39434762152529e+02 3.69924562422854e+02

; block 8

poly group "10001" mat 1 con 1 &

face id 2 &

8.28706483747381e+02 7.39357681581388e+02 4.34620473496206e+02 &

8.30150338633184e+02 7.35459866734055e+02 4.38544963342445e+02 &

8.28516744472485e+02 7.39206897844698e+02 4.40026719289599e+02 &

face id 2 &

8.30150338633184e+02 7.35459866734055e+02 4.38544963342445e+02 &

8.28706483747381e+02 7.39357681581388e+02 4.34620473496206e+02 &

8.26011586687483e+02 7.37674958461435e+02 4.37842021451426e+02 &

face id 2 &

8.28516744472485e+02 7.39206897844698e+02 4.40026719289599e+02 &

8.30150338633184e+02 7.35459866734055e+02 4.38544963342445e+02 &

8.26011586687483e+02 7.37674958461435e+02 4.37842021451426e+02 &

face id 2 &

8.28706483747381e+02 7.39357681581388e+02 4.34620473496206e+02 &

8.28516744472485e+02 7.39206897844698e+02 4.40026719289599e+02 &

8.26011586687483e+02 7.37674958461435e+02 4.37842021451426e+02

; block 9

poly group "10001" mat 1 con 1 &

face id 2 &

7.87897434460258e+02 7.35374415526222e+02 3.76760851304169e+02 &

7.84542004849083e+02 7.34101642090691e+02 3.72287161444778e+02 &

7.83487660963961e+02 7.35663106170653e+02 3.76533043538780e+02 &

face id 2 &

7.84542004849083e+02 7.34101642090691e+02 3.72287161444778e+02 &

7.87897434460258e+02 7.35374415526222e+02 3.76760851304169e+02 &

7.85027598845248e+02 7.38476661150185e+02 3.72345633894984e+02 &

face id 2 &

7.83487660963961e+02 7.35663106170653e+02 3.76533043538780e+02 &

7.84542004849083e+02 7.34101642090691e+02 3.72287161444778e+02 &

7.85027598845248e+02 7.38476661150185e+02 3.72345633894984e+02 &

face id 2 &

7.87897434460258e+02 7.35374415526222e+02 3.76760851304169e+02 &

7.83487660963961e+02 7.35663106170653e+02 3.76533043538780e+02 &

7.85027598845248e+02 7.38476661150185e+02 3.72345633894984e+02

; block 10

poly group "10001" mat 1 con 1 &

face id 2 &

7.71135925292969e+02 7.37570331301490e+02 3.67890739425259e+02 &

7.71135925292969e+02 7.34852222738633e+02 3.64848876953125e+02 &

7.73853484607426e+02 7.38926977297481e+02 3.64848876953125e+02 &

face id 2 &

7.71135925292969e+02 7.34852222738633e+02 3.64848876953125e+02 &

7.71135925292969e+02 7.37570331301490e+02 3.67890739425259e+02 &

7.74873482850570e+02 7.34379916376657e+02 3.64848876953125e+02 &

face id 1 &

7.73853484607426e+02 7.38926977297481e+02 3.64848876953125e+02 &

7.71135925292969e+02 7.34852222738633e+02 3.64848876953125e+02 &

7.74873482850570e+02 7.34379916376657e+02 3.64848876953125e+02 &

face id 2 &

7.71135925292969e+02 7.37570331301490e+02 3.67890739425259e+02 &

7.73853484607426e+02 7.38926977297481e+02 3.64848876953125e+02 &

7.74873482850570e+02 7.34379916376657e+02 3.64848876953125e+02

; block 11

poly group "10001" mat 1 con 1 &

face id 1 &

7.71135925292969e+02 7.34852222738633e+02 3.64848876953125e+02 &

7.71135925292969e+02 7.37570331301490e+02 3.67890739425259e+02 &

7.71135925292969e+02 7.34076365039034e+02 3.68803624990762e+02 &

face id 2 &

7.71135925292969e+02 7.37570331301490e+02 3.67890739425259e+02 &

7.71135925292969e+02 7.34852222738633e+02 3.64848876953125e+02 &

7.74873482850570e+02 7.34379916376657e+02 3.64848876953125e+02 &

face id 2 &

7.71135925292969e+02 7.34076365039034e+02 3.68803624990762e+02 &

7.71135925292969e+02 7.37570331301490e+02 3.67890739425259e+02 &

7.74873482850570e+02 7.34379916376657e+02 3.64848876953125e+02 &

face id 2 &

7.71135925292969e+02 7.34852222738633e+02 3.64848876953125e+02 &

7.71135925292969e+02 7.34076365039034e+02 3.68803624990762e+02 &

7.74873482850570e+02 7.34379916376657e+02 3.64848876953125e+02

; block 12

poly group "10001" mat 1 con 1 &

face id 1 &

8.18827984754551e+02 7.42957763671875e+02 4.21773857031163e+02 &

8.17389222175387e+02 7.42957763671875e+02 4.15634944560790e+02 &

8.22722716594357e+02 7.42957763671875e+02 4.17247040728472e+02 &

face id 2 &

8.17389222175387e+02 7.42957763671875e+02 4.15634944560790e+02 &

8.18827984754551e+02 7.42957763671875e+02 4.21773857031163e+02 &

8.16786864436763e+02 7.38348293032234e+02 4.19074465024219e+02 &

face id 2 &

8.22722716594357e+02 7.42957763671875e+02 4.17247040728472e+02 &

8.17389222175387e+02 7.42957763671875e+02 4.15634944560790e+02 &

8.16786864436763e+02 7.38348293032234e+02 4.19074465024219e+02 &

face id 2 &

8.18827984754551e+02 7.42957763671875e+02 4.21773857031163e+02 &

8.22722716594357e+02 7.42957763671875e+02 4.17247040728472e+02 &

8.16786864436763e+02 7.38348293032234e+02 4.19074465024219e+02

; block 13

poly group "10001" mat 1 con 1 &

face id 2 &

8.08261313858064e+02 7.31153198242188e+02 4.15940627976668e+02 &

8.09722830709629e+02 7.35099693077143e+02 4.16724782696196e+02 &

8.12035696916532e+02 7.31153198242188e+02 4.15510532530018e+02 &

face id 2 &

8.09722830709629e+02 7.35099693077143e+02 4.16724782696196e+02 &

8.08261313858064e+02 7.31153198242188e+02 4.15940627976668e+02 &

8.11257337378527e+02 7.34892257183169e+02 4.13167370048717e+02 &

face id 2 &

8.12035696916532e+02 7.31153198242188e+02 4.15510532530018e+02 &

8.09722830709629e+02 7.35099693077143e+02 4.16724782696196e+02 &

8.11257337378527e+02 7.34892257183169e+02 4.13167370048717e+02 &

face id 2 &

8.08261313858064e+02 7.31153198242188e+02 4.15940627976668e+02 &

8.12035696916532e+02 7.31153198242188e+02 4.15510532530018e+02 &

8.11257337378527e+02 7.34892257183169e+02 4.13167370048717e+02

; block 14

poly group "10001" mat 1 con 1 &

face id 2 &

8.07206434877838e+02 7.34828533348313e+02 4.13909643827570e+02 &

8.09722830709629e+02 7.35099693077143e+02 4.16724782696196e+02 &

8.08261313858064e+02 7.31153198242188e+02 4.15940627976668e+02 &

face id 2 &

8.09722830709629e+02 7.35099693077143e+02 4.16724782696196e+02 &

8.07206434877838e+02 7.34828533348313e+02 4.13909643827570e+02 &

8.11257337378527e+02 7.34892257183169e+02 4.13167370048717e+02 &

face id 2 &

8.08261313858064e+02 7.31153198242188e+02 4.15940627976668e+02 &

8.09722830709629e+02 7.35099693077143e+02 4.16724782696196e+02 &

8.11257337378527e+02 7.34892257183169e+02 4.13167370048717e+02 &

face id 2 &

8.07206434877838e+02 7.34828533348313e+02 4.13909643827570e+02 &

8.08261313858064e+02 7.31153198242188e+02 4.15940627976668e+02 &

8.11257337378527e+02 7.34892257183169e+02 4.13167370048717e+02

; block 15

poly group "10001" mat 1 con 1 &

face id 2 &

8.32888075996738e+02 7.31153198242188e+02 4.17223739127876e+02 &

8.26023627752663e+02 7.35851143679981e+02 4.17420806529159e+02 &

8.31592196255603e+02 7.37553570391624e+02 4.20696295870736e+02 &

face id 2 &

8.26023627752663e+02 7.35851143679981e+02 4.17420806529159e+02 &

8.32888075996738e+02 7.31153198242188e+02 4.17223739127876e+02 &

8.30534430583977e+02 7.37647109839753e+02 4.13260662990091e+02 &

face id 2 &

8.31592196255603e+02 7.37553570391624e+02 4.20696295870736e+02 &

8.26023627752663e+02 7.35851143679981e+02 4.17420806529159e+02 &

8.30534430583977e+02 7.37647109839753e+02 4.13260662990091e+02 &

face id 2 &

8.32888075996738e+02 7.31153198242188e+02 4.17223739127876e+02 &

8.31592196255603e+02 7.37553570391624e+02 4.20696295870736e+02 &

8.30534430583977e+02 7.37647109839753e+02 4.13260662990091e+02

; block 16

poly group "10001" mat 1 con 1 &

face id 2 &

8.13694588926832e+02 7.34627007610324e+02 4.24114636773096e+02 &

8.12144870341643e+02 7.31153198242188e+02 4.22594160457549e+02 &

8.10294982910156e+02 7.33683044433594e+02 4.24688598632812e+02 &

face id 2 &

8.12144870341643e+02 7.31153198242188e+02 4.22594160457549e+02 &

8.13694588926832e+02 7.34627007610324e+02 4.24114636773096e+02 &

8.12353907525031e+02 7.34871351232252e+02 4.20363434442144e+02 &

face id 2 &

8.10294982910156e+02 7.33683044433594e+02 4.24688598632812e+02 &

8.12144870341643e+02 7.31153198242188e+02 4.22594160457549e+02 &

8.12353907525031e+02 7.34871351232252e+02 4.20363434442144e+02 &

face id 2 &

8.13694588926832e+02 7.34627007610324e+02 4.24114636773096e+02 &

8.10294982910156e+02 7.33683044433594e+02 4.24688598632812e+02 &

8.12353907525031e+02 7.34871351232252e+02 4.20363434442144e+02

; block 17

poly group "10001" mat 1 con 1 &

face id 2 &

8.20992676355960e+02 7.42957763671875e+02 4.09981770246072e+02 &

8.24755271421208e+02 7.36547619220907e+02 4.09070766481084e+02 &

8.27273584754918e+02 7.42957763671875e+02 4.12480220146875e+02 &

face id 2 &

8.24755271421208e+02 7.36547619220907e+02 4.09070766481084e+02 &

8.20992676355960e+02 7.42957763671875e+02 4.09981770246072e+02 &

8.20437476796949e+02 7.36584150205391e+02 4.14823811708292e+02 &

face id 2 &

8.27273584754918e+02 7.42957763671875e+02 4.12480220146875e+02 &

8.24755271421208e+02 7.36547619220907e+02 4.09070766481084e+02 &

8.20437476796949e+02 7.36584150205391e+02 4.14823811708292e+02 &

face id 2 &

8.20992676355960e+02 7.42957763671875e+02 4.09981770246072e+02 &

8.27273584754918e+02 7.42957763671875e+02 4.12480220146875e+02 &

8.20437476796949e+02 7.36584150205391e+02 4.14823811708292e+02

; block 18

poly group "10001" mat 1 con 1 &

face id 2 &

8.14249908486812e+02 7.35177858463510e+02 4.16789366059111e+02 &

8.12035696916532e+02 7.31153198242188e+02 4.15510532530018e+02 &

8.09722830709629e+02 7.35099693077143e+02 4.16724782696196e+02 &

face id 2 &

8.12035696916532e+02 7.31153198242188e+02 4.15510532530018e+02 &

8.14249908486812e+02 7.35177858463510e+02 4.16789366059111e+02 &

8.11257337378527e+02 7.34892257183169e+02 4.13167370048717e+02 &

face id 2 &

8.09722830709629e+02 7.35099693077143e+02 4.16724782696196e+02 &

8.12035696916532e+02 7.31153198242188e+02 4.15510532530018e+02 &

8.11257337378527e+02 7.34892257183169e+02 4.13167370048717e+02 &

face id 2 &

8.14249908486812e+02 7.35177858463510e+02 4.16789366059111e+02 &

8.09722830709629e+02 7.35099693077143e+02 4.16724782696196e+02 &

8.11257337378527e+02 7.34892257183169e+02 4.13167370048717e+02

; block 19

poly group "10001" mat 1 con 1 &

face id 2 &

8.03993643326886e+02 7.38727533005205e+02 3.95422628877349e+02 &

8.02809345752658e+02 7.35127916281908e+02 3.91487446480074e+02 &

8.06109708892715e+02 7.37858790834568e+02 3.89837284033218e+02 &

face id 2 &

8.02809345752658e+02 7.35127916281908e+02 3.91487446480074e+02 &

8.03993643326886e+02 7.38727533005205e+02 3.95422628877349e+02 &

8.06533727168147e+02 7.34687160194815e+02 3.94660458496592e+02 &

face id 2 &

8.06109708892715e+02 7.37858790834568e+02 3.89837284033218e+02 &

8.02809345752658e+02 7.35127916281908e+02 3.91487446480074e+02 &

8.06533727168147e+02 7.34687160194815e+02 3.94660458496592e+02 &

face id 2 &

8.03993643326886e+02 7.38727533005205e+02 3.95422628877349e+02 &

8.06109708892715e+02 7.37858790834568e+02 3.89837284033218e+02 &

8.06533727168147e+02 7.34687160194815e+02 3.94660458496592e+02

; block 20

poly group "10001" mat 1 con 1 &

face id 2 &

8.10641773231557e+02 7.37725836349123e+02 3.93566404294409e+02 &

8.03993643326886e+02 7.38727533005205e+02 3.95422628877349e+02 &

8.06109708892715e+02 7.37858790834568e+02 3.89837284033218e+02 &

face id 2 &

8.03993643326886e+02 7.38727533005205e+02 3.95422628877349e+02 &

8.10641773231557e+02 7.37725836349123e+02 3.93566404294409e+02 &

8.06533727168147e+02 7.34687160194815e+02 3.94660458496592e+02 &

face id 2 &

8.06109708892715e+02 7.37858790834568e+02 3.89837284033218e+02 &

8.03993643326886e+02 7.38727533005205e+02 3.95422628877349e+02 &

8.06533727168147e+02 7.34687160194815e+02 3.94660458496592e+02 &

face id 2 &

8.10641773231557e+02 7.37725836349123e+02 3.93566404294409e+02 &

8.06109708892715e+02 7.37858790834568e+02 3.89837284033218e+02 &

8.06533727168147e+02 7.34687160194815e+02 3.94660458496592e+02

; block 21

poly group "10001" mat 1 con 1 &

face id 2 &

8.08008378521269e+02 7.38730493882952e+02 3.99001486076239e+02 &

8.03993643326886e+02 7.38727533005205e+02 3.95422628877349e+02 &

8.10641773231557e+02 7.37725836349123e+02 3.93566404294409e+02 &

face id 2 &

8.03993643326886e+02 7.38727533005205e+02 3.95422628877349e+02 &

8.08008378521269e+02 7.38730493882952e+02 3.99001486076239e+02 &

8.06533727168147e+02 7.34687160194815e+02 3.94660458496592e+02 &

face id 2 &

8.10641773231557e+02 7.37725836349123e+02 3.93566404294409e+02 &

8.03993643326886e+02 7.38727533005205e+02 3.95422628877349e+02 &

8.06533727168147e+02 7.34687160194815e+02 3.94660458496592e+02 &

face id 2 &

8.08008378521269e+02 7.38730493882952e+02 3.99001486076239e+02 &

8.10641773231557e+02 7.37725836349123e+02 3.93566404294409e+02 &

8.06533727168147e+02 7.34687160194815e+02 3.94660458496592e+02

; block 22

poly group "10001" mat 1 con 1 &

face id 2 &

8.03993643326886e+02 7.38727533005205e+02 3.95422628877349e+02 &

8.08008378521269e+02 7.38730493882952e+02 3.99001486076239e+02 &

8.05722853410866e+02 7.35087646434574e+02 3.98900933743773e+02 &

face id 2 &

8.08008378521269e+02 7.38730493882952e+02 3.99001486076239e+02 &

8.03993643326886e+02 7.38727533005205e+02 3.95422628877349e+02 &

8.06533727168147e+02 7.34687160194815e+02 3.94660458496592e+02 &

face id 2 &

8.05722853410866e+02 7.35087646434574e+02 3.98900933743773e+02 &

8.08008378521269e+02 7.38730493882952e+02 3.99001486076239e+02 &

8.06533727168147e+02 7.34687160194815e+02 3.94660458496592e+02 &

face id 2 &

8.03993643326886e+02 7.38727533005205e+02 3.95422628877349e+02 &

8.05722853410866e+02 7.35087646434574e+02 3.98900933743773e+02 &

8.06533727168147e+02 7.34687160194815e+02 3.94660458496592e+02

; block 23

poly group "10001" mat 1 con 1 &

face id 1 &

7.75682933598744e+02 7.40739985908110e+02 3.77714938437535e+02 &

7.74307374524590e+02 7.38276177052764e+02 3.77641478314468e+02 &

7.73026424837277e+02 7.40248588391272e+02 3.75361224909564e+02 &

face id 2 &

7.74307374524590e+02 7.38276177052764e+02 3.77641478314468e+02 &

7.75682933598744e+02 7.40739985908110e+02 3.77714938437535e+02 &

7.76938768959141e+02 7.39984334820939e+02 3.75213120298485e+02 &

face id 2 &

7.73026424837277e+02 7.40248588391272e+02 3.75361224909564e+02 &

7.74307374524590e+02 7.38276177052764e+02 3.77641478314468e+02 &

7.76938768959141e+02 7.39984334820939e+02 3.75213120298485e+02 &

face id 2 &

7.75682933598744e+02 7.40739985908110e+02 3.77714938437535e+02 &

7.73026424837277e+02 7.40248588391272e+02 3.75361224909564e+02 &

7.76938768959141e+02 7.39984334820939e+02 3.75213120298485e+02

; block 24

poly group "10001" mat 1 con 1 &

face id 2 &

7.74275596880056e+02 7.39458613389495e+02 3.71359700373594e+02 &

7.76268518704517e+02 7.42957763671875e+02 3.72447920290831e+02 &

7.73026424837277e+02 7.40248588391272e+02 3.75361224909564e+02 &

face id 2 &

7.76268518704517e+02 7.42957763671875e+02 3.72447920290831e+02 &

7.74275596880056e+02 7.39458613389495e+02 3.71359700373594e+02 &

7.76938768959141e+02 7.39984334820939e+02 3.75213120298485e+02 &

face id 2 &

7.73026424837277e+02 7.40248588391272e+02 3.75361224909564e+02 &

7.76268518704517e+02 7.42957763671875e+02 3.72447920290831e+02 &

7.76938768959141e+02 7.39984334820939e+02 3.75213120298485e+02 &

face id 2 &

7.74275596880056e+02 7.39458613389495e+02 3.71359700373594e+02 &

7.73026424837277e+02 7.40248588391272e+02 3.75361224909564e+02 &

7.76938768959141e+02 7.39984334820939e+02 3.75213120298485e+02

; block 25

poly group "10001" mat 1 con 1 &

face id 2 &

8.28903935786781e+02 7.42957763671875e+02 3.71492197084357e+02 &

8.39084289550781e+02 7.42957763671875e+02 3.64848876953125e+02 &

8.39084289550781e+02 7.37056602324881e+02 3.71762524831428e+02 &

face id 2 &

8.39084289550781e+02 7.42957763671875e+02 3.64848876953125e+02 &

8.28903935786781e+02 7.42957763671875e+02 3.71492197084357e+02 &

8.32654324314816e+02 7.37038115406820e+02 3.64848876953125e+02 &

face id 2 &

8.39084289550781e+02 7.37056602324881e+02 3.71762524831428e+02 &

8.39084289550781e+02 7.42957763671875e+02 3.64848876953125e+02 &

8.32654324314816e+02 7.37038115406820e+02 3.64848876953125e+02 &

face id 2 &

8.28903935786781e+02 7.42957763671875e+02 3.71492197084357e+02 &

8.39084289550781e+02 7.37056602324881e+02 3.71762524831428e+02 &

8.32654324314816e+02 7.37038115406820e+02 3.64848876953125e+02

; block 26

poly group "10001" mat 1 con 1 &

face id 1 &

8.11336709716380e+02 7.35514424983111e+02 4.37293766236316e+02 &

8.13871858615772e+02 7.34630144757922e+02 4.39260921721436e+02 &

8.12824694618945e+02 7.33817339552651e+02 4.36694878313933e+02 &

face id 2 &

8.13871858615772e+02 7.34630144757922e+02 4.39260921721436e+02 &

8.11336709716380e+02 7.35514424983111e+02 4.37293766236316e+02 &

8.14183182034257e+02 7.37171551191613e+02 4.36783264905590e+02 &

face id 2 &

8.12824694618945e+02 7.33817339552651e+02 4.36694878313933e+02 &

8.13871858615772e+02 7.34630144757922e+02 4.39260921721436e+02 &

8.14183182034257e+02 7.37171551191613e+02 4.36783264905590e+02 &

face id 2 &

8.11336709716380e+02 7.35514424983111e+02 4.37293766236316e+02 &

8.12824694618945e+02 7.33817339552651e+02 4.36694878313933e+02 &

8.14183182034257e+02 7.37171551191613e+02 4.36783264905590e+02

; block 27

poly group "10001" mat 1 con 1 &

face id 1 &

8.39084289550781e+02 7.37056602324881e+02 3.71762524831428e+02 &

8.39084289550781e+02 7.42957763671875e+02 3.64848876953125e+02 &

8.39084289550781e+02 7.31153198242188e+02 3.64848876953125e+02 &

face id 2 &

8.39084289550781e+02 7.42957763671875e+02 3.64848876953125e+02 &

8.39084289550781e+02 7.37056602324881e+02 3.71762524831428e+02 &

8.32654324314816e+02 7.37038115406820e+02 3.64848876953125e+02 &

face id 1 &

8.39084289550781e+02 7.31153198242188e+02 3.64848876953125e+02 &

8.39084289550781e+02 7.42957763671875e+02 3.64848876953125e+02 &

8.32654324314816e+02 7.37038115406820e+02 3.64848876953125e+02 &

face id 2 &

8.39084289550781e+02 7.37056602324881e+02 3.71762524831428e+02 &

8.39084289550781e+02 7.31153198242188e+02 3.64848876953125e+02 &

8.32654324314816e+02 7.37038115406820e+02 3.64848876953125e+02

; block 28

poly group "10001" mat 1 con 1 &

face id 2 &

8.09490919631596e+02 7.36167724749901e+02 4.19676489619966e+02 &

8.10593672000049e+02 7.39659613160195e+02 4.21874911950142e+02 &

8.14352799333017e+02 7.39139456449856e+02 4.22176948605109e+02 &

face id 2 &

8.10593672000049e+02 7.39659613160195e+02 4.21874911950142e+02 &

8.09490919631596e+02 7.36167724749901e+02 4.19676489619966e+02 &

8.12647078267193e+02 7.38844593444619e+02 4.17279149585504e+02 &

face id 2 &

8.14352799333017e+02 7.39139456449856e+02 4.22176948605109e+02 &

8.10593672000049e+02 7.39659613160195e+02 4.21874911950142e+02 &

8.12647078267193e+02 7.38844593444619e+02 4.17279149585504e+02 &

face id 2 &

8.09490919631596e+02 7.36167724749901e+02 4.19676489619966e+02 &

8.14352799333017e+02 7.39139456449856e+02 4.22176948605109e+02 &

8.12647078267193e+02 7.38844593444619e+02 4.17279149585504e+02

; block 29

poly group "10001" mat 1 con 1 &

face id 2 &

7.97797335860310e+02 7.31153198242188e+02 4.07175625594507e+02 &

7.97326795645079e+02 7.33484291964610e+02 4.05886842936342e+02 &

7.95502299357658e+02 7.31153198242188e+02 4.06252065709902e+02 &

face id 2 &

7.97326795645079e+02 7.33484291964610e+02 4.05886842936342e+02 &

7.97797335860310e+02 7.31153198242188e+02 4.07175625594507e+02 &

7.95446009423280e+02 7.33370401603083e+02 4.07686153733035e+02 &

face id 2 &

7.95502299357658e+02 7.31153198242188e+02 4.06252065709902e+02 &

7.97326795645079e+02 7.33484291964610e+02 4.05886842936342e+02 &

7.95446009423280e+02 7.33370401603083e+02 4.07686153733035e+02 &

face id 2 &

7.97797335860310e+02 7.31153198242188e+02 4.07175625594507e+02 &

7.95502299357658e+02 7.31153198242188e+02 4.06252065709902e+02 &

7.95446009423280e+02 7.33370401603083e+02 4.07686153733035e+02

; block 30

poly group "10001" mat 1 con 1 &

face id 2 &

7.89197276621344e+02 7.34576411507420e+02 3.90327463427344e+02 &

7.90851304530732e+02 7.36812816901129e+02 3.93877274718733e+02 &

7.92640570936094e+02 7.34389419631712e+02 3.91408058836435e+02 &

face id 2 &

7.90851304530732e+02 7.36812816901129e+02 3.93877274718733e+02 &

7.89197276621344e+02 7.34576411507420e+02 3.90327463427344e+02 &

7.93075306390299e+02 7.37259553714495e+02 3.89912466417267e+02 &

face id 2 &

7.92640570936094e+02 7.34389419631712e+02 3.91408058836435e+02 &

7.90851304530732e+02 7.36812816901129e+02 3.93877274718733e+02 &

7.93075306390299e+02 7.37259553714495e+02 3.89912466417267e+02 &

face id 2 &

7.89197276621344e+02 7.34576411507420e+02 3.90327463427344e+02 &

7.92640570936094e+02 7.34389419631712e+02 3.91408058836435e+02 &

7.93075306390299e+02 7.37259553714495e+02 3.89912466417267e+02

; block 31

poly group "10001" mat 1 con 1 &

face id 2 &

8.13871858615772e+02 7.34630144757922e+02 4.39260921721436e+02 &

8.15793233645232e+02 7.34747728264843e+02 4.35883786023879e+02 &

8.12824694618945e+02 7.33817339552651e+02 4.36694878313933e+02 &

face id 2 &

8.15793233645232e+02 7.34747728264843e+02 4.35883786023879e+02 &

8.13871858615772e+02 7.34630144757922e+02 4.39260921721436e+02 &

8.14183182034257e+02 7.37171551191613e+02 4.36783264905590e+02 &

face id 2 &

8.12824694618945e+02 7.33817339552651e+02 4.36694878313933e+02 &

8.15793233645232e+02 7.34747728264843e+02 4.35883786023879e+02 &

8.14183182034257e+02 7.37171551191613e+02 4.36783264905590e+02 &

face id 2 &

8.13871858615772e+02 7.34630144757922e+02 4.39260921721436e+02 &

8.12824694618945e+02 7.33817339552651e+02 4.36694878313933e+02 &

8.14183182034257e+02 7.37171551191613e+02 4.36783264905590e+02

; block 32

poly group "10001" mat 1 con 1 &

face id 2 &

7.99664551669819e+02 7.38727869564032e+02 3.98609473831640e+02 &

7.99432805907969e+02 7.34735523444890e+02 4.01271303355683e+02 &

7.99224647204598e+02 7.39423268495429e+02 4.03544318874035e+02 &

face id 2 &

7.99432805907969e+02 7.34735523444890e+02 4.01271303355683e+02 &

7.99664551669819e+02 7.38727869564032e+02 3.98609473831640e+02 &

7.96463284819671e+02 7.36777948701819e+02 4.01765944068918e+02 &

face id 2 &

7.99224647204598e+02 7.39423268495429e+02 4.03544318874035e+02 &

7.99432805907969e+02 7.34735523444890e+02 4.01271303355683e+02 &

7.96463284819671e+02 7.36777948701819e+02 4.01765944068918e+02 &

face id 2 &

7.99664551669819e+02 7.38727869564032e+02 3.98609473831640e+02 &

7.99224647204598e+02 7.39423268495429e+02 4.03544318874035e+02 &

7.96463284819671e+02 7.36777948701819e+02 4.01765944068918e+02

; block 33

poly group "10001" mat 1 con 1 &

face id 2 &

7.71135925292969e+02 7.34076365039034e+02 3.68803624990762e+02 &

7.75366396130398e+02 7.34213436458492e+02 3.69324910452187e+02 &

7.74432015880905e+02 7.31153198242188e+02 3.72068947519307e+02 &

face id 2 &

7.75366396130398e+02 7.34213436458492e+02 3.69324910452187e+02 &

7.71135925292969e+02 7.34076365039034e+02 3.68803624990762e+02 &

7.73503107340380e+02 7.31153198242188e+02 3.68146209117521e+02 &

face id 2 &

7.74432015880905e+02 7.31153198242188e+02 3.72068947519307e+02 &

7.75366396130398e+02 7.34213436458492e+02 3.69324910452187e+02 &

7.73503107340380e+02 7.31153198242188e+02 3.68146209117521e+02 &

face id 2 &

7.71135925292969e+02 7.34076365039034e+02 3.68803624990762e+02 &

7.74432015880905e+02 7.31153198242188e+02 3.72068947519307e+02 &

7.73503107340380e+02 7.31153198242188e+02 3.68146209117521e+02

; block 34

poly group "10001" mat 1 con 1 &

face id 1 &

8.34585057442727e+02 7.42957763671875e+02 4.45469991642717e+02 &

8.34694045880472e+02 7.42957763671875e+02 4.40556675161879e+02 &

8.39084289550781e+02 7.42957763671875e+02 4.43160901718362e+02 &

face id 2 &

8.34694045880472e+02 7.42957763671875e+02 4.40556675161879e+02 &

8.34585057442727e+02 7.42957763671875e+02 4.45469991642717e+02 &

8.34729885421035e+02 7.36525928807045e+02 4.41869552698341e+02 &

face id 2 &

8.39084289550781e+02 7.42957763671875e+02 4.43160901718362e+02 &

8.34694045880472e+02 7.42957763671875e+02 4.40556675161879e+02 &

8.34729885421035e+02 7.36525928807045e+02 4.41869552698341e+02 &

face id 2 &

8.34585057442727e+02 7.42957763671875e+02 4.45469991642717e+02 &

8.39084289550781e+02 7.42957763671875e+02 4.43160901718362e+02 &

8.34729885421035e+02 7.36525928807045e+02 4.41869552698341e+02

; block 35

poly group "10001" mat 1 con 1 &

face id 1 &

8.39084289550781e+02 7.37053357299508e+02 4.39638740088288e+02 &

8.39084289550781e+02 7.36818187365417e+02 4.44196269944235e+02 &

8.39084289550781e+02 7.42957763671875e+02 4.43160901718362e+02 &

face id 2 &

8.39084289550781e+02 7.36818187365417e+02 4.44196269944235e+02 &

8.39084289550781e+02 7.37053357299508e+02 4.39638740088288e+02 &

8.34729885421035e+02 7.36525928807045e+02 4.41869552698341e+02 &

face id 2 &

8.39084289550781e+02 7.42957763671875e+02 4.43160901718362e+02 &

8.39084289550781e+02 7.36818187365417e+02 4.44196269944235e+02 &

8.34729885421035e+02 7.36525928807045e+02 4.41869552698341e+02 &

face id 2 &

8.39084289550781e+02 7.37053357299508e+02 4.39638740088288e+02 &

8.39084289550781e+02 7.42957763671875e+02 4.43160901718362e+02 &

8.34729885421035e+02 7.36525928807045e+02 4.41869552698341e+02

; block 36

poly group "10001" mat 1 con 1 &

face id 2 &

8.04234314501538e+02 7.34635014532937e+02 4.05451928779116e+02 &

8.08631556812620e+02 7.34644604063582e+02 4.06314744213599e+02 &

8.08005581782927e+02 7.38153886431079e+02 4.07831518617536e+02 &

face id 2 &

8.08631556812620e+02 7.34644604063582e+02 4.06314744213599e+02 &

8.04234314501538e+02 7.34635014532937e+02 4.05451928779116e+02 &

8.04748042289060e+02 7.34593085936688e+02 4.08934836753061e+02 &

face id 2 &

8.08005581782927e+02 7.38153886431079e+02 4.07831518617536e+02 &

8.08631556812620e+02 7.34644604063582e+02 4.06314744213599e+02 &

8.04748042289060e+02 7.34593085936688e+02 4.08934836753061e+02 &

face id 2 &

8.04234314501538e+02 7.34635014532937e+02 4.05451928779116e+02 &

8.08005581782927e+02 7.38153886431079e+02 4.07831518617536e+02 &

8.04748042289060e+02 7.34593085936688e+02 4.08934836753061e+02

; block 37

poly group "10001" mat 1 con 1 &

face id 1 &

8.08062608321876e+02 7.31153198242188e+02 3.81109923197277e+02 &

8.10009578774608e+02 7.31153198242188e+02 3.86599906858927e+02 &

8.15100275115170e+02 7.31153198242188e+02 3.82923343064734e+02 &

face id 2 &

8.10009578774608e+02 7.31153198242188e+02 3.86599906858927e+02 &

8.08062608321876e+02 7.31153198242188e+02 3.81109923197277e+02 &

8.07581738970324e+02 7.36655126437911e+02 3.84328905480290e+02 &

face id 2 &

8.15100275115170e+02 7.31153198242188e+02 3.82923343064734e+02 &

8.10009578774608e+02 7.31153198242188e+02 3.86599906858927e+02 &

8.07581738970324e+02 7.36655126437911e+02 3.84328905480290e+02 &

face id 2 &

8.08062608321876e+02 7.31153198242188e+02 3.81109923197277e+02 &

8.15100275115170e+02 7.31153198242188e+02 3.82923343064734e+02 &

8.07581738970324e+02 7.36655126437911e+02 3.84328905480290e+02

; block 38

poly group "10001" mat 1 con 1 &

face id 2 &

8.24925363977043e+02 7.31153198242188e+02 3.71989177722232e+02 &

8.24640127572402e+02 7.37081027998942e+02 3.77991472841031e+02 &

8.28903935786781e+02 7.42957763671875e+02 3.71492197084357e+02 &

face id 2 &

8.24640127572402e+02 7.37081027998942e+02 3.77991472841031e+02 &

8.24925363977043e+02 7.31153198242188e+02 3.71989177722232e+02 &

8.20842798872635e+02 7.37189646407413e+02 3.71833994279653e+02 &

face id 2 &

8.28903935786781e+02 7.42957763671875e+02 3.71492197084357e+02 &

8.24640127572402e+02 7.37081027998942e+02 3.77991472841031e+02 &

8.20842798872635e+02 7.37189646407413e+02 3.71833994279653e+02 &

face id 2 &

8.24925363977043e+02 7.31153198242188e+02 3.71989177722232e+02 &

8.28903935786781e+02 7.42957763671875e+02 3.71492197084357e+02 &

8.20842798872635e+02 7.37189646407413e+02 3.71833994279653e+02

; block 39

poly group "10001" mat 1 con 1 &

face id 2 &

8.00965458695504e+02 7.31153198242188e+02 3.91758115901608e+02 &

8.02809345752658e+02 7.35127916281908e+02 3.91487446480074e+02 &

8.00423884947867e+02 7.31153198242188e+02 3.87599039819564e+02 &

face id 2 &

8.02809345752658e+02 7.35127916281908e+02 3.91487446480074e+02 &

8.00965458695504e+02 7.31153198242188e+02 3.91758115901608e+02 &

7.99028431323575e+02 7.35618187258196e+02 3.90154438356032e+02 &

face id 2 &

8.00423884947867e+02 7.31153198242188e+02 3.87599039819564e+02 &

8.02809345752658e+02 7.35127916281908e+02 3.91487446480074e+02 &

7.99028431323575e+02 7.35618187258196e+02 3.90154438356032e+02 &

face id 2 &

8.00965458695504e+02 7.31153198242188e+02 3.91758115901608e+02 &

8.00423884947867e+02 7.31153198242188e+02 3.87599039819564e+02 &

7.99028431323575e+02 7.35618187258196e+02 3.90154438356032e+02

; block 40

poly group "10001" mat 1 con 1 &

face id 1 &

7.94211527624996e+02 7.31153198242188e+02 3.96061394416997e+02 &

7.97607558832901e+02 7.31153198242188e+02 3.95040058596455e+02 &

7.94113331391958e+02 7.31153198242188e+02 3.92954018796712e+02 &

face id 2 &

7.97607558832901e+02 7.31153198242188e+02 3.95040058596455e+02 &

7.94211527624996e+02 7.31153198242188e+02 3.96061394416997e+02 &

7.96478775035514e+02 7.34528043696007e+02 3.93001842890862e+02 &

face id 2 &

7.94113331391958e+02 7.31153198242188e+02 3.92954018796712e+02 &

7.97607558832901e+02 7.31153198242188e+02 3.95040058596455e+02 &

7.96478775035514e+02 7.34528043696007e+02 3.93001842890862e+02 &

face id 2 &

7.94211527624996e+02 7.31153198242188e+02 3.96061394416997e+02 &

7.94113331391958e+02 7.31153198242188e+02 3.92954018796712e+02 &

7.96478775035514e+02 7.34528043696007e+02 3.93001842890862e+02

; block 41

poly group "10001" mat 1 con 1 &

face id 1 &

8.05097224411228e+02 7.42957763671875e+02 3.93075069571725e+02 &

8.00873343269525e+02 7.42957763671875e+02 3.96358347833586e+02 &

8.00896600562332e+02 7.42957763671875e+02 3.91322005726277e+02 &

face id 2 &

8.00873343269525e+02 7.42957763671875e+02 3.96358347833586e+02 &

8.05097224411228e+02 7.42957763671875e+02 3.93075069571725e+02 &

7.99389806333365e+02 7.38801050700467e+02 3.93943509497245e+02 &

face id 2 &

8.00896600562332e+02 7.42957763671875e+02 3.91322005726277e+02 &

8.00873343269525e+02 7.42957763671875e+02 3.96358347833586e+02 &

7.99389806333365e+02 7.38801050700467e+02 3.93943509497245e+02 &

face id 2 &

8.05097224411228e+02 7.42957763671875e+02 3.93075069571725e+02 &

8.00896600562332e+02 7.42957763671875e+02 3.91322005726277e+02 &

7.99389806333365e+02 7.38801050700467e+02 3.93943509497245e+02

; block 42

poly group "10001" mat 1 con 1 &

face id 1 &

8.07751909840409e+02 7.42957763671875e+02 3.82260514378822e+02 &

8.14017271454085e+02 7.42957763671875e+02 3.83339423702604e+02 &

8.09805525478267e+02 7.42957763671875e+02 3.89445971649157e+02 &

face id 2 &

8.14017271454085e+02 7.42957763671875e+02 3.83339423702604e+02 &

8.07751909840409e+02 7.42957763671875e+02 3.82260514378822e+02 &

8.07581738970324e+02 7.36655126437911e+02 3.84328905480290e+02 &

face id 2 &

8.09805525478267e+02 7.42957763671875e+02 3.89445971649157e+02 &

8.14017271454085e+02 7.42957763671875e+02 3.83339423702604e+02 &

8.07581738970324e+02 7.36655126437911e+02 3.84328905480290e+02 &

face id 2 &

8.07751909840409e+02 7.42957763671875e+02 3.82260514378822e+02 &

8.09805525478267e+02 7.42957763671875e+02 3.89445971649157e+02 &

8.07581738970324e+02 7.36655126437911e+02 3.84328905480290e+02

; block 43

poly group "10001" mat 1 con 1 &

face id 2 &

8.14249908486812e+02 7.35177858463510e+02 4.16789366059111e+02 &

8.12647078267193e+02 7.38844593444619e+02 4.17279149585504e+02 &

8.09722830709629e+02 7.35099693077143e+02 4.16724782696196e+02 &

face id 2 &

8.12647078267193e+02 7.38844593444619e+02 4.17279149585504e+02 &

8.14249908486812e+02 7.35177858463510e+02 4.16789366059111e+02 &

8.12353907525031e+02 7.34871351232252e+02 4.20363434442144e+02 &

face id 2 &

8.09722830709629e+02 7.35099693077143e+02 4.16724782696196e+02 &

8.12647078267193e+02 7.38844593444619e+02 4.17279149585504e+02 &

8.12353907525031e+02 7.34871351232252e+02 4.20363434442144e+02 &

face id 2 &

8.14249908486812e+02 7.35177858463510e+02 4.16789366059111e+02 &

8.09722830709629e+02 7.35099693077143e+02 4.16724782696196e+02 &

8.12353907525031e+02 7.34871351232252e+02 4.20363434442144e+02

; block 44

poly group "10001" mat 1 con 1 &

face id 1 &

8.27273584754918e+02 7.42957763671875e+02 4.12480220146875e+02 &

8.33324474406425e+02 7.42957763671875e+02 4.15623786886125e+02 &

8.28202156483526e+02 7.42957763671875e+02 4.19788988709625e+02 &

face id 2 &

8.33324474406425e+02 7.42957763671875e+02 4.15623786886125e+02 &

8.27273584754918e+02 7.42957763671875e+02 4.12480220146875e+02 &

8.30534430583977e+02 7.37647109839753e+02 4.13260662990091e+02 &

face id 2 &

8.28202156483526e+02 7.42957763671875e+02 4.19788988709625e+02 &

8.33324474406425e+02 7.42957763671875e+02 4.15623786886125e+02 &

8.30534430583977e+02 7.37647109839753e+02 4.13260662990091e+02 &

face id 2 &

8.27273584754918e+02 7.42957763671875e+02 4.12480220146875e+02 &

8.28202156483526e+02 7.42957763671875e+02 4.19788988709625e+02 &

8.30534430583977e+02 7.37647109839753e+02 4.13260662990091e+02

; block 45

poly group "10001" mat 1 con 1 &

face id 2 &

8.05267509908869e+02 7.37189947377813e+02 4.17093434190900e+02 &

8.07206434877838e+02 7.34828533348313e+02 4.13909643827570e+02 &

8.03140082525883e+02 7.36388256837763e+02 4.14903214031251e+02 &

face id 2 &

8.07206434877838e+02 7.34828533348313e+02 4.13909643827570e+02 &

8.05267509908869e+02 7.37189947377813e+02 4.17093434190900e+02 &

8.06053783905492e+02 7.39353100736547e+02 4.13045444506629e+02 &

face id 2 &

8.03140082525883e+02 7.36388256837763e+02 4.14903214031251e+02 &

8.07206434877838e+02 7.34828533348313e+02 4.13909643827570e+02 &

8.06053783905492e+02 7.39353100736547e+02 4.13045444506629e+02 &

face id 2 &

8.05267509908869e+02 7.37189947377813e+02 4.17093434190900e+02 &

8.03140082525883e+02 7.36388256837763e+02 4.14903214031251e+02 &

8.06053783905492e+02 7.39353100736547e+02 4.13045444506629e+02

; block 46

poly group "10001" mat 1 con 1 &

face id 1 &

8.09809660917787e+02 7.42957763671875e+02 4.19037083632094e+02 &

8.12445679494332e+02 7.42957763671875e+02 4.14750818125651e+02 &

8.14143386380350e+02 7.42957763671875e+02 4.19814593480705e+02 &

face id 2 &

8.12445679494332e+02 7.42957763671875e+02 4.14750818125651e+02 &

8.09809660917787e+02 7.42957763671875e+02 4.19037083632094e+02 &

8.12647078267193e+02 7.38844593444619e+02 4.17279149585504e+02 &

face id 2 &

8.14143386380350e+02 7.42957763671875e+02 4.19814593480705e+02 &

8.12445679494332e+02 7.42957763671875e+02 4.14750818125651e+02 &

8.12647078267193e+02 7.38844593444619e+02 4.17279149585504e+02 &

face id 2 &

8.09809660917787e+02 7.42957763671875e+02 4.19037083632094e+02 &

8.14143386380350e+02 7.42957763671875e+02 4.19814593480705e+02 &

8.12647078267193e+02 7.38844593444619e+02 4.17279149585504e+02

; block 47

poly group "10001" mat 1 con 1 &

face id 1 &

8.18827984754551e+02 7.42957763671875e+02 4.21773857031163e+02 &

8.23718108440382e+02 7.42957763671875e+02 4.24060560484302e+02 &

8.19213703414815e+02 7.42957763671875e+02 4.27495334690443e+02 &

face id 2 &

8.23718108440382e+02 7.42957763671875e+02 4.24060560484302e+02 &

8.18827984754551e+02 7.42957763671875e+02 4.21773857031163e+02 &

8.18126133140210e+02 7.39128284797895e+02 4.24521969903783e+02 &

face id 2 &

8.19213703414815e+02 7.42957763671875e+02 4.27495334690443e+02 &

8.23718108440382e+02 7.42957763671875e+02 4.24060560484302e+02 &

8.18126133140210e+02 7.39128284797895e+02 4.24521969903783e+02 &

face id 2 &

8.18827984754551e+02 7.42957763671875e+02 4.21773857031163e+02 &

8.19213703414815e+02 7.42957763671875e+02 4.27495334690443e+02 &

8.18126133140210e+02 7.39128284797895e+02 4.24521969903783e+02

; block 48

poly group "10001" mat 1 con 1 &

face id 1 &

8.18069174339446e+02 7.42957763671875e+02 4.01829283575629e+02 &

8.11726863481470e+02 7.42957763671875e+02 4.05255074248258e+02 &

8.11893258522981e+02 7.42957763671875e+02 4.00369720464388e+02 &

face id 2 &

8.11726863481470e+02 7.42957763671875e+02 4.05255074248258e+02 &

8.18069174339446e+02 7.42957763671875e+02 4.01829283575629e+02 &

8.14194519614525e+02 7.36222240308663e+02 4.03223835774641e+02 &

face id 2 &

8.11893258522981e+02 7.42957763671875e+02 4.00369720464388e+02 &

8.11726863481470e+02 7.42957763671875e+02 4.05255074248258e+02 &

8.14194519614525e+02 7.36222240308663e+02 4.03223835774641e+02 &

face id 2 &

8.18069174339446e+02 7.42957763671875e+02 4.01829283575629e+02 &

8.11893258522981e+02 7.42957763671875e+02 4.00369720464388e+02 &

8.14194519614525e+02 7.36222240308663e+02 4.03223835774641e+02

; block 49

poly group "10001" mat 1 con 1 &

face id 1 &

8.22198614973049e+02 7.31153198242188e+02 4.17110132334116e+02 &

8.18663640401986e+02 7.31153198242188e+02 4.20323564658305e+02 &

8.22847947705952e+02 7.31153198242188e+02 4.22600298353562e+02 &

face id 2 &

8.18663640401986e+02 7.31153198242188e+02 4.20323564658305e+02 &

8.22198614973049e+02 7.31153198242188e+02 4.17110132334116e+02 &

8.22003939543759e+02 7.37603465338854e+02 4.20982666462121e+02 &

face id 2 &

8.22847947705952e+02 7.31153198242188e+02 4.22600298353562e+02 &

8.18663640401986e+02 7.31153198242188e+02 4.20323564658305e+02 &

8.22003939543759e+02 7.37603465338854e+02 4.20982666462121e+02 &

face id 2 &

8.22198614973049e+02 7.31153198242188e+02 4.17110132334116e+02 &

8.22847947705952e+02 7.31153198242188e+02 4.22600298353562e+02 &

8.22003939543759e+02 7.37603465338854e+02 4.20982666462121e+02

; block 50

poly group "10001" mat 1 con 1 &

face id 1 &

8.11683577279594e+02 7.42957763671875e+02 4.26990978647697e+02 &

8.15105453724136e+02 7.42957763671875e+02 4.24551856435235e+02 &

8.14891237294761e+02 7.42957763671875e+02 4.28254850378271e+02 &

face id 2 &

8.15105453724136e+02 7.42957763671875e+02 4.24551856435235e+02 &

8.11683577279594e+02 7.42957763671875e+02 4.26990978647697e+02 &

8.13756389123963e+02 7.39589893800934e+02 4.25916239240713e+02 &

face id 2 &

8.14891237294761e+02 7.42957763671875e+02 4.28254850378271e+02 &

8.15105453724136e+02 7.42957763671875e+02 4.24551856435235e+02 &

8.13756389123963e+02 7.39589893800934e+02 4.25916239240713e+02 &

face id 2 &

8.11683577279594e+02 7.42957763671875e+02 4.26990978647697e+02 &

8.14891237294761e+02 7.42957763671875e+02 4.28254850378271e+02 &

8.13756389123963e+02 7.39589893800934e+02 4.25916239240713e+02

; block 51

poly group "10001" mat 1 con 1 &

face id 2 &

7.99389806333365e+02 7.38801050700467e+02 3.93943509497245e+02 &

8.05097224411228e+02 7.42957763671875e+02 3.93075069571725e+02 &

8.00873343269525e+02 7.42957763671875e+02 3.96358347833586e+02 &

face id 2 &

8.05097224411228e+02 7.42957763671875e+02 3.93075069571725e+02 &

7.99389806333365e+02 7.38801050700467e+02 3.93943509497245e+02 &

8.03993643326886e+02 7.38727533005205e+02 3.95422628877349e+02 &

face id 2 &

8.00873343269525e+02 7.42957763671875e+02 3.96358347833586e+02 &

8.05097224411228e+02 7.42957763671875e+02 3.93075069571725e+02 &

8.03993643326886e+02 7.38727533005205e+02 3.95422628877349e+02 &

face id 2 &

7.99389806333365e+02 7.38801050700467e+02 3.93943509497245e+02 &

8.00873343269525e+02 7.42957763671875e+02 3.96358347833586e+02 &

8.03993643326886e+02 7.38727533005205e+02 3.95422628877349e+02

; block 52

poly group "10001" mat 1 con 1 &

face id 2 &

8.03140082525883e+02 7.36388256837763e+02 4.14903214031251e+02 &

8.07206434877838e+02 7.34828533348313e+02 4.13909643827570e+02 &

8.03509241519356e+02 7.36685442706800e+02 4.11505637635243e+02 &

face id 2 &

8.07206434877838e+02 7.34828533348313e+02 4.13909643827570e+02 &

8.03140082525883e+02 7.36388256837763e+02 4.14903214031251e+02 &

8.06053783905492e+02 7.39353100736547e+02 4.13045444506629e+02 &

face id 2 &

8.03509241519356e+02 7.36685442706800e+02 4.11505637635243e+02 &

8.07206434877838e+02 7.34828533348313e+02 4.13909643827570e+02 &

8.06053783905492e+02 7.39353100736547e+02 4.13045444506629e+02 &

face id 2 &

8.03140082525883e+02 7.36388256837763e+02 4.14903214031251e+02 &

8.03509241519356e+02 7.36685442706800e+02 4.11505637635243e+02 &

8.06053783905492e+02 7.39353100736547e+02 4.13045444506629e+02

; block 53

poly group "10001" mat 1 con 1 &

face id 2 &

8.09864479417309e+02 7.34571602650726e+02 4.00066379407306e+02 &

8.10641773231557e+02 7.37725836349123e+02 3.93566404294409e+02 &

8.11593521502643e+02 7.31153198242188e+02 3.97113810821721e+02 &

face id 2 &

8.10641773231557e+02 7.37725836349123e+02 3.93566404294409e+02 &

8.09864479417309e+02 7.34571602650726e+02 4.00066379407306e+02 &

8.06533727168147e+02 7.34687160194815e+02 3.94660458496592e+02 &

face id 2 &

8.11593521502643e+02 7.31153198242188e+02 3.97113810821721e+02 &

8.10641773231557e+02 7.37725836349123e+02 3.93566404294409e+02 &

8.06533727168147e+02 7.34687160194815e+02 3.94660458496592e+02 &

face id 2 &

8.09864479417309e+02 7.34571602650726e+02 4.00066379407306e+02 &

8.11593521502643e+02 7.31153198242188e+02 3.97113810821721e+02 &

8.06533727168147e+02 7.34687160194815e+02 3.94660458496592e+02

; block 54

poly group "10001" mat 1 con 1 &

face id 1 &

7.78553435143339e+02 7.37372081584988e+02 3.81246946594663e+02 &

7.75612143490425e+02 7.36202827550051e+02 3.79657937703356e+02 &

7.76623604261048e+02 7.38637497875149e+02 3.79313058357629e+02 &

face id 2 &

7.75612143490425e+02 7.36202827550051e+02 3.79657937703356e+02 &

7.78553435143339e+02 7.37372081584988e+02 3.81246946594663e+02 &

7.77051870553251e+02 7.37085809011748e+02 3.76695713437529e+02 &

face id 2 &

7.76623604261048e+02 7.38637497875149e+02 3.79313058357629e+02 &

7.75612143490425e+02 7.36202827550051e+02 3.79657937703356e+02 &

7.77051870553251e+02 7.37085809011748e+02 3.76695713437529e+02 &

face id 2 &

7.78553435143339e+02 7.37372081584988e+02 3.81246946594663e+02 &

7.76623604261048e+02 7.38637497875149e+02 3.79313058357629e+02 &

7.77051870553251e+02 7.37085809011748e+02 3.76695713437529e+02

; block 55

poly group "10001" mat 1 con 1 &

face id 2 &

8.00446633691467e+02 7.34890585746234e+02 3.94456651409666e+02 &

8.02809345752658e+02 7.35127916281908e+02 3.91487446480074e+02 &

8.00965458695504e+02 7.31153198242188e+02 3.91758115901608e+02 &

face id 2 &

8.02809345752658e+02 7.35127916281908e+02 3.91487446480074e+02 &

8.00446633691467e+02 7.34890585746234e+02 3.94456651409666e+02 &

7.99028431323575e+02 7.35618187258196e+02 3.90154438356032e+02 &

face id 2 &

8.00965458695504e+02 7.31153198242188e+02 3.91758115901608e+02 &

8.02809345752658e+02 7.35127916281908e+02 3.91487446480074e+02 &

7.99028431323575e+02 7.35618187258196e+02 3.90154438356032e+02 &

face id 2 &

8.00446633691467e+02 7.34890585746234e+02 3.94456651409666e+02 &

8.00965458695504e+02 7.31153198242188e+02 3.91758115901608e+02 &

7.99028431323575e+02 7.35618187258196e+02 3.90154438356032e+02

; block 56

poly group "10001" mat 1 con 1 &

face id 1 &

7.76690916896458e+02 7.33984982361572e+02 3.81358736446098e+02 &

7.75612143490425e+02 7.36202827550051e+02 3.79657937703356e+02 &

7.78553435143339e+02 7.37372081584988e+02 3.81246946594663e+02 &

face id 2 &

7.75612143490425e+02 7.36202827550051e+02 3.79657937703356e+02 &

7.76690916896458e+02 7.33984982361572e+02 3.81358736446098e+02 &

7.79511950549799e+02 7.34457282336568e+02 3.78709527306644e+02 &

face id 2 &

7.78553435143339e+02 7.37372081584988e+02 3.81246946594663e+02 &

7.75612143490425e+02 7.36202827550051e+02 3.79657937703356e+02 &

7.79511950549799e+02 7.34457282336568e+02 3.78709527306644e+02 &

face id 2 &

7.76690916896458e+02 7.33984982361572e+02 3.81358736446098e+02 &

7.78553435143339e+02 7.37372081584988e+02 3.81246946594663e+02 &

7.79511950549799e+02 7.34457282336568e+02 3.78709527306644e+02

; block 57

poly group "10001" mat 1 con 1 &

face id 1 &

8.05043104633530e+02 7.42957763671875e+02 4.11063531291342e+02 &

8.09796767008882e+02 7.42957763671875e+02 4.10290671888704e+02 &

8.07792915601657e+02 7.42957763671875e+02 4.14815876761776e+02 &

face id 2 &

8.09796767008882e+02 7.42957763671875e+02 4.10290671888704e+02 &

8.05043104633530e+02 7.42957763671875e+02 4.11063531291342e+02 &

8.06053783905492e+02 7.39353100736547e+02 4.13045444506629e+02 &

face id 2 &

8.07792915601657e+02 7.42957763671875e+02 4.14815876761776e+02 &

8.09796767008882e+02 7.42957763671875e+02 4.10290671888704e+02 &

8.06053783905492e+02 7.39353100736547e+02 4.13045444506629e+02 &

face id 2 &

8.05043104633530e+02 7.42957763671875e+02 4.11063531291342e+02 &

8.07792915601657e+02 7.42957763671875e+02 4.14815876761776e+02 &

8.06053783905492e+02 7.39353100736547e+02 4.13045444506629e+02

; block 58

poly group "10001" mat 1 con 1 &

face id 1 &

8.13870124921521e+02 7.42957763671875e+02 3.95224545195568e+02 &

8.11893258522981e+02 7.42957763671875e+02 4.00369720464388e+02 &

8.08851677477977e+02 7.42957763671875e+02 3.96344698776517e+02 &

face id 2 &

8.11893258522981e+02 7.42957763671875e+02 4.00369720464388e+02 &

8.13870124921521e+02 7.42957763671875e+02 3.95224545195568e+02 &

8.13648731082524e+02 7.37198409103348e+02 3.98003234961222e+02 &

face id 2 &

8.08851677477977e+02 7.42957763671875e+02 3.96344698776517e+02 &

8.11893258522981e+02 7.42957763671875e+02 4.00369720464388e+02 &

8.13648731082524e+02 7.37198409103348e+02 3.98003234961222e+02 &

face id 2 &

8.13870124921521e+02 7.42957763671875e+02 3.95224545195568e+02 &

8.08851677477977e+02 7.42957763671875e+02 3.96344698776517e+02 &

8.13648731082524e+02 7.37198409103348e+02 3.98003234961222e+02

; block 59

poly group "10001" mat 1 con 1 &

face id 1 &

8.04364753763366e+02 7.42957763671875e+02 3.98815314415627e+02 &

8.05097224411228e+02 7.42957763671875e+02 3.93075069571725e+02 &

8.08851677477977e+02 7.42957763671875e+02 3.96344698776517e+02 &

face id 2 &

8.05097224411228e+02 7.42957763671875e+02 3.93075069571725e+02 &

8.04364753763366e+02 7.42957763671875e+02 3.98815314415627e+02 &

8.03993643326886e+02 7.38727533005205e+02 3.95422628877349e+02 &

face id 2 &

8.08851677477977e+02 7.42957763671875e+02 3.96344698776517e+02 &

8.05097224411228e+02 7.42957763671875e+02 3.93075069571725e+02 &

8.03993643326886e+02 7.38727533005205e+02 3.95422628877349e+02 &

face id 2 &

8.04364753763366e+02 7.42957763671875e+02 3.98815314415627e+02 &

8.08851677477977e+02 7.42957763671875e+02 3.96344698776517e+02 &

8.03993643326886e+02 7.38727533005205e+02 3.95422628877349e+02

; block 60

poly group "10001" mat 1 con 1 &

face id 1 &

7.75032978427684e+02 7.42957763671875e+02 3.76072155832965e+02 &

7.76268518704517e+02 7.42957763671875e+02 3.72447920290831e+02 &

7.79220406464566e+02 7.42957763671875e+02 3.75205861997925e+02 &

face id 2 &

7.76268518704517e+02 7.42957763671875e+02 3.72447920290831e+02 &

7.75032978427684e+02 7.42957763671875e+02 3.76072155832965e+02 &

7.76938768959141e+02 7.39984334820939e+02 3.75213120298485e+02 &

face id 2 &

7.79220406464566e+02 7.42957763671875e+02 3.75205861997925e+02 &

7.76268518704517e+02 7.42957763671875e+02 3.72447920290831e+02 &

7.76938768959141e+02 7.39984334820939e+02 3.75213120298485e+02 &

face id 2 &

7.75032978427684e+02 7.42957763671875e+02 3.76072155832965e+02 &

7.79220406464566e+02 7.42957763671875e+02 3.75205861997925e+02 &

7.76938768959141e+02 7.39984334820939e+02 3.75213120298485e+02

; block 61

poly group "10001" mat 1 con 1 &

face id 1 &

7.76623604261048e+02 7.38637497875149e+02 3.79313058357629e+02 &

7.78505842137311e+02 7.40529325582286e+02 3.79457485086928e+02 &

7.78553435143339e+02 7.37372081584988e+02 3.81246946594663e+02 &

face id 2 &

7.78505842137311e+02 7.40529325582286e+02 3.79457485086928e+02 &

7.76623604261048e+02 7.38637497875149e+02 3.79313058357629e+02 &

7.80799502317009e+02 7.38595032412240e+02 3.78079266885418e+02 &

face id 2 &

7.78553435143339e+02 7.37372081584988e+02 3.81246946594663e+02 &

7.78505842137311e+02 7.40529325582286e+02 3.79457485086928e+02 &

7.80799502317009e+02 7.38595032412240e+02 3.78079266885418e+02 &

face id 2 &

7.76623604261048e+02 7.38637497875149e+02 3.79313058357629e+02 &

7.78553435143339e+02 7.37372081584988e+02 3.81246946594663e+02 &

7.80799502317009e+02 7.38595032412240e+02 3.78079266885418e+02

; block 62

poly group "10001" mat 1 con 1 &

face id 1 &

7.86150062467024e+02 7.42957763671875e+02 3.78920105584715e+02 &

7.82723936045558e+02 7.42957763671875e+02 3.77023845732179e+02 &

7.85724375088134e+02 7.42957763671875e+02 3.75809207520845e+02 &

face id 2 &

7.82723936045558e+02 7.42957763671875e+02 3.77023845732179e+02 &

7.86150062467024e+02 7.42957763671875e+02 3.78920105584715e+02 &

7.84345668476437e+02 7.40135313357215e+02 3.78526374317212e+02 &

face id 2 &

7.85724375088134e+02 7.42957763671875e+02 3.75809207520845e+02 &

7.82723936045558e+02 7.42957763671875e+02 3.77023845732179e+02 &

7.84345668476437e+02 7.40135313357215e+02 3.78526374317212e+02 &

face id 2 &

7.86150062467024e+02 7.42957763671875e+02 3.78920105584715e+02 &

7.85724375088134e+02 7.42957763671875e+02 3.75809207520845e+02 &

7.84345668476437e+02 7.40135313357215e+02 3.78526374317212e+02

; block 63

poly group "10001" mat 1 con 1 &

face id 1 &

7.82723936045558e+02 7.42957763671875e+02 3.77023845732179e+02 &

7.83243069165867e+02 7.42957763671875e+02 3.72786606032438e+02 &

7.85724375088134e+02 7.42957763671875e+02 3.75809207520845e+02 &

face id 2 &

7.83243069165867e+02 7.42957763671875e+02 3.72786606032438e+02 &

7.82723936045558e+02 7.42957763671875e+02 3.77023845732179e+02 &

7.83813396428186e+02 7.39314645759285e+02 3.75273836784356e+02 &

face id 2 &

7.85724375088134e+02 7.42957763671875e+02 3.75809207520845e+02 &

7.83243069165867e+02 7.42957763671875e+02 3.72786606032438e+02 &

7.83813396428186e+02 7.39314645759285e+02 3.75273836784356e+02 &

face id 2 &

7.82723936045558e+02 7.42957763671875e+02 3.77023845732179e+02 &

7.85724375088134e+02 7.42957763671875e+02 3.75809207520845e+02 &

7.83813396428186e+02 7.39314645759285e+02 3.75273836784356e+02

; block 64

poly group "10001" mat 1 con 1 &

face id 2 &

8.33190865039410e+02 7.35305529052256e+02 4.34142847499269e+02 &

8.33864722379183e+02 7.39652653709875e+02 4.32623313919057e+02 &

8.39084289550781e+02 7.37015214063697e+02 4.30185524731766e+02 &

face id 2 &

8.33864722379183e+02 7.39652653709875e+02 4.32623313919057e+02 &

8.33190865039410e+02 7.35305529052256e+02 4.34142847499269e+02 &

8.33405021547574e+02 7.37173781106677e+02 4.27268238018421e+02 &

face id 2 &

8.39084289550781e+02 7.37015214063697e+02 4.30185524731766e+02 &

8.33864722379183e+02 7.39652653709875e+02 4.32623313919057e+02 &

8.33405021547574e+02 7.37173781106677e+02 4.27268238018421e+02 &

face id 2 &

8.33190865039410e+02 7.35305529052256e+02 4.34142847499269e+02 &

8.39084289550781e+02 7.37015214063697e+02 4.30185524731766e+02 &

8.33405021547574e+02 7.37173781106677e+02 4.27268238018421e+02

; block 65

poly group "10001" mat 1 con 1 &

face id 1 &

7.96802081798252e+02 7.42957763671875e+02 3.69534985512364e+02 &

7.93898632039194e+02 7.42957763671875e+02 3.64848876953125e+02 &

7.99831206538623e+02 7.42957763671875e+02 3.64848876953125e+02 &

face id 2 &

7.93898632039194e+02 7.42957763671875e+02 3.64848876953125e+02 &

7.96802081798252e+02 7.42957763671875e+02 3.69534985512364e+02 &

7.98072850148110e+02 7.38921841899001e+02 3.69042179430648e+02 &

face id 2 &

7.99831206538623e+02 7.42957763671875e+02 3.64848876953125e+02 &

7.93898632039194e+02 7.42957763671875e+02 3.64848876953125e+02 &

7.98072850148110e+02 7.38921841899001e+02 3.69042179430648e+02 &

face id 2 &

7.96802081798252e+02 7.42957763671875e+02 3.69534985512364e+02 &

7.99831206538623e+02 7.42957763671875e+02 3.64848876953125e+02 &

7.98072850148110e+02 7.38921841899001e+02 3.69042179430648e+02

; block 66

poly group "10001" mat 1 con 1 &

face id 2 &

8.11726863481470e+02 7.42957763671875e+02 4.05255074248258e+02 &

8.10063396272645e+02 7.38264255256752e+02 4.03157482752262e+02 &

8.11893258522981e+02 7.42957763671875e+02 4.00369720464388e+02 &

face id 2 &

8.10063396272645e+02 7.38264255256752e+02 4.03157482752262e+02 &

8.11726863481470e+02 7.42957763671875e+02 4.05255074248258e+02 &

8.14194519614525e+02 7.36222240308663e+02 4.03223835774641e+02 &

face id 2 &

8.11893258522981e+02 7.42957763671875e+02 4.00369720464388e+02 &

8.10063396272645e+02 7.38264255256752e+02 4.03157482752262e+02 &

8.14194519614525e+02 7.36222240308663e+02 4.03223835774641e+02 &

face id 2 &

8.11726863481470e+02 7.42957763671875e+02 4.05255074248258e+02 &

8.11893258522981e+02 7.42957763671875e+02 4.00369720464388e+02 &

8.14194519614525e+02 7.36222240308663e+02 4.03223835774641e+02

; block 67

poly group "10001" mat 1 con 1 &

face id 2 &

8.24755271421208e+02 7.36547619220907e+02 4.09070766481084e+02 &

8.26023627752663e+02 7.35851143679981e+02 4.17420806529159e+02 &

8.27273584754918e+02 7.42957763671875e+02 4.12480220146875e+02 &

face id 2 &

8.26023627752663e+02 7.35851143679981e+02 4.17420806529159e+02 &

8.24755271421208e+02 7.36547619220907e+02 4.09070766481084e+02 &

8.20437476796949e+02 7.36584150205391e+02 4.14823811708292e+02 &

face id 2 &

8.27273584754918e+02 7.42957763671875e+02 4.12480220146875e+02 &

8.26023627752663e+02 7.35851143679981e+02 4.17420806529159e+02 &

8.20437476796949e+02 7.36584150205391e+02 4.14823811708292e+02 &

face id 2 &

8.24755271421208e+02 7.36547619220907e+02 4.09070766481084e+02 &

8.27273584754918e+02 7.42957763671875e+02 4.12480220146875e+02 &

8.20437476796949e+02 7.36584150205391e+02 4.14823811708292e+02

; block 68

poly group "10001" mat 1 con 1 &

face id 1 &

7.95824267105237e+02 7.42957763671875e+02 3.85406053453334e+02 &

7.92696333260128e+02 7.42957763671875e+02 3.88796385777957e+02 &

7.91463798947070e+02 7.42957763671875e+02 3.84768523563060e+02 &

face id 2 &

7.92696333260128e+02 7.42957763671875e+02 3.88796385777957e+02 &

7.95824267105237e+02 7.42957763671875e+02 3.85406053453334e+02 &

7.93126102700700e+02 7.39147352988408e+02 3.85920040094875e+02 &

face id 2 &

7.91463798947070e+02 7.42957763671875e+02 3.84768523563060e+02 &

7.92696333260128e+02 7.42957763671875e+02 3.88796385777957e+02 &

7.93126102700700e+02 7.39147352988408e+02 3.85920040094875e+02 &

face id 2 &

7.95824267105237e+02 7.42957763671875e+02 3.85406053453334e+02 &

7.91463798947070e+02 7.42957763671875e+02 3.84768523563060e+02 &

7.93126102700700e+02 7.39147352988408e+02 3.85920040094875e+02

; block 69

poly group "10001" mat 1 con 1 &

face id 1 &

7.99724447295145e+02 7.42957763671875e+02 3.87022301448822e+02 &

7.96671058174266e+02 7.42957763671875e+02 3.90227925573037e+02 &

7.95824267105237e+02 7.42957763671875e+02 3.85406053453334e+02 &

face id 2 &

7.96671058174266e+02 7.42957763671875e+02 3.90227925573037e+02 &

7.99724447295145e+02 7.42957763671875e+02 3.87022301448822e+02 &

7.97168838062648e+02 7.39187632994892e+02 3.86818051061183e+02 &

face id 2 &

7.95824267105237e+02 7.42957763671875e+02 3.85406053453334e+02 &

7.96671058174266e+02 7.42957763671875e+02 3.90227925573037e+02 &

7.97168838062648e+02 7.39187632994892e+02 3.86818051061183e+02 &

face id 2 &

7.99724447295145e+02 7.42957763671875e+02 3.87022301448822e+02 &

7.95824267105237e+02 7.42957763671875e+02 3.85406053453334e+02 &

7.97168838062648e+02 7.39187632994892e+02 3.86818051061183e+02

; block 70

poly group "10001" mat 1 con 1 &

face id 2 &

8.01323359896620e+02 7.34865317685774e+02 4.13114368486747e+02 &

8.03509241519356e+02 7.36685442706800e+02 4.11505637635243e+02 &

8.03140082525883e+02 7.36388256837763e+02 4.14903214031251e+02 &

face id 2 &

8.03509241519356e+02 7.36685442706800e+02 4.11505637635243e+02 &

8.01323359896620e+02 7.34865317685774e+02 4.13114368486747e+02 &

8.04465957264557e+02 7.33969583985651e+02 4.12069612436356e+02 &

face id 2 &

8.03140082525883e+02 7.36388256837763e+02 4.14903214031251e+02 &

8.03509241519356e+02 7.36685442706800e+02 4.11505637635243e+02 &

8.04465957264557e+02 7.33969583985651e+02 4.12069612436356e+02 &

face id 2 &

8.01323359896620e+02 7.34865317685774e+02 4.13114368486747e+02 &

8.03140082525883e+02 7.36388256837763e+02 4.14903214031251e+02 &

8.04465957264557e+02 7.33969583985651e+02 4.12069612436356e+02

; block 71

poly group "10001" mat 1 con 1 &

face id 1 &

8.15234808544163e+02 7.42957763671875e+02 4.09308534149983e+02 &

8.11726863481470e+02 7.42957763671875e+02 4.05255074248258e+02 &

8.18069174339446e+02 7.42957763671875e+02 4.01829283575629e+02 &

face id 2 &

8.11726863481470e+02 7.42957763671875e+02 4.05255074248258e+02 &

8.15234808544163e+02 7.42957763671875e+02 4.09308534149983e+02 &

8.18044212102941e+02 7.37294444794664e+02 4.06947605911675e+02 &

face id 2 &

8.18069174339446e+02 7.42957763671875e+02 4.01829283575629e+02 &

8.11726863481470e+02 7.42957763671875e+02 4.05255074248258e+02 &

8.18044212102941e+02 7.37294444794664e+02 4.06947605911675e+02 &

face id 2 &

8.15234808544163e+02 7.42957763671875e+02 4.09308534149983e+02 &

8.18069174339446e+02 7.42957763671875e+02 4.01829283575629e+02 &

8.18044212102941e+02 7.37294444794664e+02 4.06947605911675e+02

; block 72

poly group "10001" mat 1 con 1 &

face id 2 &

8.09796767008882e+02 7.42957763671875e+02 4.10290671888704e+02 &

8.09911809905185e+02 7.38789513583694e+02 4.13229940854814e+02 &

8.07792915601657e+02 7.42957763671875e+02 4.14815876761776e+02 &

face id 2 &

8.09911809905185e+02 7.38789513583694e+02 4.13229940854814e+02 &

8.09796767008882e+02 7.42957763671875e+02 4.10290671888704e+02 &

8.06053783905492e+02 7.39353100736547e+02 4.13045444506629e+02 &

face id 2 &

8.07792915601657e+02 7.42957763671875e+02 4.14815876761776e+02 &

8.09911809905185e+02 7.38789513583694e+02 4.13229940854814e+02 &

8.06053783905492e+02 7.39353100736547e+02 4.13045444506629e+02 &

face id 2 &

8.09796767008882e+02 7.42957763671875e+02 4.10290671888704e+02 &

8.07792915601657e+02 7.42957763671875e+02 4.14815876761776e+02 &

8.06053783905492e+02 7.39353100736547e+02 4.13045444506629e+02

; block 73

poly group "10001" mat 1 con 1 &

face id 1 &

8.02303389777493e+02 7.42957763671875e+02 4.08048950041985e+02 &

8.01098614236767e+02 7.42957763671875e+02 4.11833138231907e+02 &

7.98543325683381e+02 7.42957763671875e+02 4.09357360801871e+02 &

face id 2 &

8.01098614236767e+02 7.42957763671875e+02 4.11833138231907e+02 &

8.02303389777493e+02 7.42957763671875e+02 4.08048950041985e+02 &

7.99326131256449e+02 7.40590042422744e+02 4.07820655966596e+02 &

face id 2 &

7.98543325683381e+02 7.42957763671875e+02 4.09357360801871e+02 &

8.01098614236767e+02 7.42957763671875e+02 4.11833138231907e+02 &

7.99326131256449e+02 7.40590042422744e+02 4.07820655966596e+02 &

face id 2 &

8.02303389777493e+02 7.42957763671875e+02 4.08048950041985e+02 &

7.98543325683381e+02 7.42957763671875e+02 4.09357360801871e+02 &

7.99326131256449e+02 7.40590042422744e+02 4.07820655966596e+02

; block 74

poly group "10001" mat 1 con 1 &

face id 2 &

7.97326795645079e+02 7.33484291964610e+02 4.05886842936342e+02 &

7.97797335860310e+02 7.31153198242188e+02 4.07175625594507e+02 &

7.97589752627985e+02 7.34292202745755e+02 4.08481026952103e+02 &

face id 2 &

7.97797335860310e+02 7.31153198242188e+02 4.07175625594507e+02 &

7.97326795645079e+02 7.33484291964610e+02 4.05886842936342e+02 &

7.95446009423280e+02 7.33370401603083e+02 4.07686153733035e+02 &

face id 2 &

7.97589752627985e+02 7.34292202745755e+02 4.08481026952103e+02 &

7.97797335860310e+02 7.31153198242188e+02 4.07175625594507e+02 &

7.95446009423280e+02 7.33370401603083e+02 4.07686153733035e+02 &

face id 2 &

7.97326795645079e+02 7.33484291964610e+02 4.05886842936342e+02 &

7.97589752627985e+02 7.34292202745755e+02 4.08481026952103e+02 &

7.95446009423280e+02 7.33370401603083e+02 4.07686153733035e+02

; block 75

poly group "10001" mat 1 con 1 &

face id 2 &

8.18073172819596e+02 7.39472123297221e+02 4.29992682558518e+02 &

8.15983052506966e+02 7.36922527517859e+02 4.33218426937274e+02 &

8.14314473444842e+02 7.39174793419805e+02 4.30562106333613e+02 &

face id 2 &

8.15983052506966e+02 7.36922527517859e+02 4.33218426937274e+02 &

8.18073172819596e+02 7.39472123297221e+02 4.29992682558518e+02 &

8.16363471593303e+02 7.36867764055710e+02 4.27997707946941e+02 &

face id 2 &

8.14314473444842e+02 7.39174793419805e+02 4.30562106333613e+02 &

8.15983052506966e+02 7.36922527517859e+02 4.33218426937274e+02 &

8.16363471593303e+02 7.36867764055710e+02 4.27997707946941e+02 &

face id 2 &

8.18073172819596e+02 7.39472123297221e+02 4.29992682558518e+02 &

8.14314473444842e+02 7.39174793419805e+02 4.30562106333613e+02 &

8.16363471593303e+02 7.36867764055710e+02 4.27997707946941e+02

; block 76

poly group "10001" mat 1 con 1 &

face id 2 &

8.22822252675870e+02 7.36637749364954e+02 4.41131123199765e+02 &

8.24231497582248e+02 7.39555545391830e+02 4.40655328606252e+02 &

8.20813556445955e+02 7.40175884004747e+02 4.38506718959274e+02 &

face id 2 &

8.24231497582248e+02 7.39555545391830e+02 4.40655328606252e+02 &

8.22822252675870e+02 7.36637749364954e+02 4.41131123199765e+02 &

8.21348267997198e+02 7.39634491253400e+02 4.42846308765791e+02 &

face id 2 &

8.20813556445955e+02 7.40175884004747e+02 4.38506718959274e+02 &

8.24231497582248e+02 7.39555545391830e+02 4.40655328606252e+02 &

8.21348267997198e+02 7.39634491253400e+02 4.42846308765791e+02 &

face id 2 &

8.22822252675870e+02 7.36637749364954e+02 4.41131123199765e+02 &

8.20813556445955e+02 7.40175884004747e+02 4.38506718959274e+02 &

8.21348267997198e+02 7.39634491253400e+02 4.42846308765791e+02

; block 77

poly group "10001" mat 1 con 1 &

face id 1 &

8.08068987260185e+02 7.42957763671875e+02 4.26046463140405e+02 &

8.08320277092379e+02 7.40548022708132e+02 4.28306475041886e+02 &

8.07996067368936e+02 7.40284697371919e+02 4.24709539056186e+02 &

face id 2 &

8.08320277092379e+02 7.40548022708132e+02 4.28306475041886e+02 &

8.08068987260185e+02 7.42957763671875e+02 4.26046463140405e+02 &

8.10984080669042e+02 7.39211793104898e+02 4.26585347528298e+02 &

face id 2 &

8.07996067368936e+02 7.40284697371919e+02 4.24709539056186e+02 &

8.08320277092379e+02 7.40548022708132e+02 4.28306475041886e+02 &

8.10984080669042e+02 7.39211793104898e+02 4.26585347528298e+02 &

face id 2 &

8.08068987260185e+02 7.42957763671875e+02 4.26046463140405e+02 &

8.07996067368936e+02 7.40284697371919e+02 4.24709539056186e+02 &

8.10984080669042e+02 7.39211793104898e+02 4.26585347528298e+02

; block 78

poly group "10001" mat 1 con 1 &

face id 2 &

8.18663640401986e+02 7.31153198242188e+02 4.20323564658305e+02 &

8.16747344865554e+02 7.34789113348238e+02 4.20781967480880e+02 &

8.15880858022893e+02 7.31153198242188e+02 4.23505248338825e+02 &

face id 2 &

8.16747344865554e+02 7.34789113348238e+02 4.20781967480880e+02 &

8.18663640401986e+02 7.31153198242188e+02 4.20323564658305e+02 &

8.18144726682255e+02 7.34408968805460e+02 4.25196769141094e+02 &

face id 2 &

8.15880858022893e+02 7.31153198242188e+02 4.23505248338825e+02 &

8.16747344865554e+02 7.34789113348238e+02 4.20781967480880e+02 &

8.18144726682255e+02 7.34408968805460e+02 4.25196769141094e+02 &

face id 2 &

8.18663640401986e+02 7.31153198242188e+02 4.20323564658305e+02 &

8.15880858022893e+02 7.31153198242188e+02 4.23505248338825e+02 &

8.18144726682255e+02 7.34408968805460e+02 4.25196769141094e+02

; block 79

poly group "10001" mat 1 con 1 &

face id 1 &

8.20017509411527e+02 7.31153198242188e+02 4.28097071064497e+02 &

8.17436891461985e+02 7.31153198242188e+02 4.30682311023628e+02 &

8.21847538316180e+02 7.31153198242188e+02 4.30907399722412e+02 &

face id 2 &

8.17436891461985e+02 7.31153198242188e+02 4.30682311023628e+02 &

8.20017509411527e+02 7.31153198242188e+02 4.28097071064497e+02 &

8.19506582324828e+02 7.34677513556790e+02 4.29945141932819e+02 &

face id 2 &

8.21847538316180e+02 7.31153198242188e+02 4.30907399722412e+02 &

8.17436891461985e+02 7.31153198242188e+02 4.30682311023628e+02 &

8.19506582324828e+02 7.34677513556790e+02 4.29945141932819e+02 &

face id 2 &

8.20017509411527e+02 7.31153198242188e+02 4.28097071064497e+02 &

8.21847538316180e+02 7.31153198242188e+02 4.30907399722412e+02 &

8.19506582324828e+02 7.34677513556790e+02 4.29945141932819e+02

; block 80

poly group "10001" mat 1 con 1 &

face id 2 &

8.27667286520472e+02 7.38125365214952e+02 4.29857973751925e+02 &

8.23079169574055e+02 7.38841631466596e+02 4.32490747965191e+02 &

8.24453593292951e+02 7.42957763671875e+02 4.30499495465792e+02 &

face id 2 &

8.23079169574055e+02 7.38841631466596e+02 4.32490747965191e+02 &

8.27667286520472e+02 7.38125365214952e+02 4.29857973751925e+02 &

8.22048825758541e+02 7.38705365341897e+02 4.27519172119006e+02 &

face id 2 &

8.24453593292951e+02 7.42957763671875e+02 4.30499495465792e+02 &

8.23079169574055e+02 7.38841631466596e+02 4.32490747965191e+02 &

8.22048825758541e+02 7.38705365341897e+02 4.27519172119006e+02 &

face id 2 &

8.27667286520472e+02 7.38125365214952e+02 4.29857973751925e+02 &

8.24453593292951e+02 7.42957763671875e+02 4.30499495465792e+02 &

8.22048825758541e+02 7.38705365341897e+02 4.27519172119006e+02

; block 81

poly group "10001" mat 1 con 1 &

face id 2 &

8.08320277092379e+02 7.40548022708132e+02 4.28306475041886e+02 &

8.08068987260185e+02 7.42957763671875e+02 4.26046463140405e+02 &

8.11683577279594e+02 7.42957763671875e+02 4.26990978647697e+02 &

face id 2 &

8.08068987260185e+02 7.42957763671875e+02 4.26046463140405e+02 &

8.08320277092379e+02 7.40548022708132e+02 4.28306475041886e+02 &

8.10984080669042e+02 7.39211793104898e+02 4.26585347528298e+02 &

face id 2 &

8.11683577279594e+02 7.42957763671875e+02 4.26990978647697e+02 &

8.08068987260185e+02 7.42957763671875e+02 4.26046463140405e+02 &

8.10984080669042e+02 7.39211793104898e+02 4.26585347528298e+02 &

face id 2 &

8.08320277092379e+02 7.40548022708132e+02 4.28306475041886e+02 &

8.11683577279594e+02 7.42957763671875e+02 4.26990978647697e+02 &

8.10984080669042e+02 7.39211793104898e+02 4.26585347528298e+02

; block 82

poly group "10001" mat 1 con 1 &

face id 1 &

8.16574401308042e+02 7.31153198242188e+02 4.15370838721868e+02 &

8.18663640401986e+02 7.31153198242188e+02 4.20323564658305e+02 &

8.22198614973049e+02 7.31153198242188e+02 4.17110132334116e+02 &

face id 2 &

8.18663640401986e+02 7.31153198242188e+02 4.20323564658305e+02 &

8.16574401308042e+02 7.31153198242188e+02 4.15370838721868e+02 &

8.20437476796949e+02 7.36584150205391e+02 4.14823811708292e+02 &

face id 2 &

8.22198614973049e+02 7.31153198242188e+02 4.17110132334116e+02 &

8.18663640401986e+02 7.31153198242188e+02 4.20323564658305e+02 &

8.20437476796949e+02 7.36584150205391e+02 4.14823811708292e+02 &

face id 2 &

8.16574401308042e+02 7.31153198242188e+02 4.15370838721868e+02 &

8.22198614973049e+02 7.31153198242188e+02 4.17110132334116e+02 &

8.20437476796949e+02 7.36584150205391e+02 4.14823811708292e+02

; block 83

poly group "10001" mat 1 con 1 &

face id 1 &

7.96671058174266e+02 7.42957763671875e+02 3.90227925573037e+02 &

7.99724447295145e+02 7.42957763671875e+02 3.87022301448822e+02 &

8.00896600562332e+02 7.42957763671875e+02 3.91322005726277e+02 &

face id 2 &

7.99724447295145e+02 7.42957763671875e+02 3.87022301448822e+02 &

7.96671058174266e+02 7.42957763671875e+02 3.90227925573037e+02 &

8.01076203387915e+02 7.39174910359122e+02 3.89319688925391e+02 &

face id 2 &

8.00896600562332e+02 7.42957763671875e+02 3.91322005726277e+02 &

7.99724447295145e+02 7.42957763671875e+02 3.87022301448822e+02 &

8.01076203387915e+02 7.39174910359122e+02 3.89319688925391e+02 &

face id 2 &

7.96671058174266e+02 7.42957763671875e+02 3.90227925573037e+02 &

8.00896600562332e+02 7.42957763671875e+02 3.91322005726277e+02 &

8.01076203387915e+02 7.39174910359122e+02 3.89319688925391e+02

; block 84

poly group "10001" mat 1 con 1 &

face id 1 &

7.95760540850189e+02 7.31153198242187e+02 3.98481504684038e+02 &

7.97607558832901e+02 7.31153198242188e+02 3.95040058596455e+02 &

7.94211527624996e+02 7.31153198242188e+02 3.96061394416997e+02 &

face id 2 &

7.97607558832901e+02 7.31153198242188e+02 3.95040058596455e+02 &

7.95760540850189e+02 7.31153198242187e+02 3.98481504684038e+02 &

7.97516497216007e+02 7.34563943186781e+02 3.97383221855559e+02 &

face id 2 &

7.94211527624996e+02 7.31153198242188e+02 3.96061394416997e+02 &

7.97607558832901e+02 7.31153198242188e+02 3.95040058596455e+02 &

7.97516497216007e+02 7.34563943186781e+02 3.97383221855559e+02 &

face id 2 &

7.95760540850189e+02 7.31153198242187e+02 3.98481504684038e+02 &

7.94211527624996e+02 7.31153198242188e+02 3.96061394416997e+02 &

7.97516497216007e+02 7.34563943186781e+02 3.97383221855559e+02

; block 85

poly group "10001" mat 1 con 1 &

face id 2 &

8.24925363977043e+02 7.31153198242188e+02 3.71989177722232e+02 &

8.25214620888485e+02 7.37064024906030e+02 3.64848876953125e+02 &

8.28903935786781e+02 7.42957763671875e+02 3.71492197084357e+02 &

face id 2 &

8.25214620888485e+02 7.37064024906030e+02 3.64848876953125e+02 &

8.24925363977043e+02 7.31153198242188e+02 3.71989177722232e+02 &

8.32654324314816e+02 7.37038115406820e+02 3.64848876953125e+02 &

face id 2 &

8.28903935786781e+02 7.42957763671875e+02 3.71492197084357e+02 &

8.25214620888485e+02 7.37064024906030e+02 3.64848876953125e+02 &

8.32654324314816e+02 7.37038115406820e+02 3.64848876953125e+02 &

face id 2 &

8.24925363977043e+02 7.31153198242188e+02 3.71989177722232e+02 &

8.28903935786781e+02 7.42957763671875e+02 3.71492197084357e+02 &

8.32654324314816e+02 7.37038115406820e+02 3.64848876953125e+02

; block 86

poly group "10001" mat 1 con 1 &

face id 2 &

7.94113331391958e+02 7.31153198242188e+02 3.92954018796712e+02 &

7.93143690467033e+02 7.34153895053626e+02 3.94736213202014e+02 &

7.94211527624996e+02 7.31153198242188e+02 3.96061394416997e+02 &

face id 2 &

7.93143690467033e+02 7.34153895053626e+02 3.94736213202014e+02 &

7.94113331391958e+02 7.31153198242188e+02 3.92954018796712e+02 &

7.96478775035514e+02 7.34528043696007e+02 3.93001842890862e+02 &

face id 2 &

7.94211527624996e+02 7.31153198242188e+02 3.96061394416997e+02 &

7.93143690467033e+02 7.34153895053626e+02 3.94736213202014e+02 &

7.96478775035514e+02 7.34528043696007e+02 3.93001842890862e+02 &

face id 2 &

7.94113331391958e+02 7.31153198242188e+02 3.92954018796712e+02 &

7.94211527624996e+02 7.31153198242188e+02 3.96061394416997e+02 &

7.96478775035514e+02 7.34528043696007e+02 3.93001842890862e+02

; block 87

poly group "10001" mat 1 con 1 &

face id 1 &

8.39084289550781e+02 7.42957763671875e+02 3.94516974331130e+02 &

8.32060029686998e+02 7.42957763671875e+02 3.99153584793838e+02 &

8.30768763830069e+02 7.42957763671875e+02 3.89468079453802e+02 &

face id 2 &

8.32060029686998e+02 7.42957763671875e+02 3.99153584793838e+02 &

8.39084289550781e+02 7.42957763671875e+02 3.94516974331130e+02 &

8.30209617746932e+02 7.36595444598988e+02 3.95547397157632e+02 &

face id 2 &

8.30768763830069e+02 7.42957763671875e+02 3.89468079453802e+02 &

8.32060029686998e+02 7.42957763671875e+02 3.99153584793838e+02 &

8.30209617746932e+02 7.36595444598988e+02 3.95547397157632e+02 &

face id 2 &

8.39084289550781e+02 7.42957763671875e+02 3.94516974331130e+02 &

8.30768763830069e+02 7.42957763671875e+02 3.89468079453802e+02 &

8.30209617746932e+02 7.36595444598988e+02 3.95547397157632e+02

; block 88

poly group "10001" mat 1 con 1 &

face id 2 &

8.39084289550781e+02 7.42957763671875e+02 4.47836106283018e+02 &

8.35759803702102e+02 7.42957763671875e+02 4.49303153342892e+02 &

8.39084289550781e+02 7.39301375828281e+02 4.52089202880859e+02 &

face id 1 &

8.35759803702102e+02 7.42957763671875e+02 4.49303153342892e+02 &

8.39084289550781e+02 7.42957763671875e+02 4.47836106283018e+02 &

8.39084289550781e+02 7.42957763671875e+02 4.52089202880859e+02 &

face id 2 &

8.39084289550781e+02 7.39301375828281e+02 4.52089202880859e+02 &

8.35759803702102e+02 7.42957763671875e+02 4.49303153342892e+02 &

8.39084289550781e+02 7.42957763671875e+02 4.52089202880859e+02 &

face id 1 &

8.39084289550781e+02 7.42957763671875e+02 4.47836106283018e+02 &

8.39084289550781e+02 7.39301375828281e+02 4.52089202880859e+02 &

8.39084289550781e+02 7.42957763671875e+02 4.52089202880859e+02

; block 89

poly group "10001" mat 1 con 1 &

face id 1 &

8.19171832620069e+02 7.42957763671875e+02 3.93190604054630e+02 &

8.25134549450002e+02 7.42957763671875e+02 3.96485013648556e+02 &

8.18069174339446e+02 7.42957763671875e+02 4.01829283575629e+02 &

face id 2 &

8.25134549450002e+02 7.42957763671875e+02 3.96485013648556e+02 &

8.19171832620069e+02 7.42957763671875e+02 3.93190604054630e+02 &

8.20600098315628e+02 7.37493952138853e+02 3.97440050588930e+02 &

face id 2 &

8.18069174339446e+02 7.42957763671875e+02 4.01829283575629e+02 &

8.25134549450002e+02 7.42957763671875e+02 3.96485013648556e+02 &

8.20600098315628e+02 7.37493952138853e+02 3.97440050588930e+02 &

face id 2 &

8.19171832620069e+02 7.42957763671875e+02 3.93190604054630e+02 &

8.18069174339446e+02 7.42957763671875e+02 4.01829283575629e+02 &

8.20600098315628e+02 7.37493952138853e+02 3.97440050588930e+02

; block 90

poly group "10001" mat 1 con 1 &

face id 1 &

8.25018161800981e+02 7.31153198242188e+02 4.06227920166475e+02 &

8.30305606368492e+02 7.31153198242188e+02 3.99328987610237e+02 &

8.22786902955485e+02 7.31153198242188e+02 4.00598299907967e+02 &

face id 2 &

8.30305606368492e+02 7.31153198242188e+02 3.99328987610237e+02 &

8.25018161800981e+02 7.31153198242188e+02 4.06227920166475e+02 &

8.25062590342976e+02 7.37119532782199e+02 4.02466299226852e+02 &

face id 2 &

8.22786902955485e+02 7.31153198242188e+02 4.00598299907967e+02 &

8.30305606368492e+02 7.31153198242188e+02 3.99328987610237e+02 &

8.25062590342976e+02 7.37119532782199e+02 4.02466299226852e+02 &

face id 2 &

8.25018161800981e+02 7.31153198242188e+02 4.06227920166475e+02 &

8.22786902955485e+02 7.31153198242188e+02 4.00598299907967e+02 &

8.25062590342976e+02 7.37119532782199e+02 4.02466299226852e+02

; block 91

poly group "10001" mat 1 con 1 &

face id 2 &

8.04748042289060e+02 7.34593085936688e+02 4.08934836753061e+02 &

8.03287461742746e+02 7.37038107997743e+02 4.07568950280680e+02 &

8.08005581782927e+02 7.38153886431079e+02 4.07831518617536e+02 &

face id 2 &

8.03287461742746e+02 7.37038107997743e+02 4.07568950280680e+02 &

8.04748042289060e+02 7.34593085936688e+02 4.08934836753061e+02 &

8.04234314501538e+02 7.34635014532937e+02 4.05451928779116e+02 &

face id 2 &

8.08005581782927e+02 7.38153886431079e+02 4.07831518617536e+02 &

8.03287461742746e+02 7.37038107997743e+02 4.07568950280680e+02 &

8.04234314501538e+02 7.34635014532937e+02 4.05451928779116e+02 &

face id 2 &

8.04748042289060e+02 7.34593085936688e+02 4.08934836753061e+02 &

8.08005581782927e+02 7.38153886431079e+02 4.07831518617536e+02 &

8.04234314501538e+02 7.34635014532937e+02 4.05451928779116e+02

; block 92

poly group "10001" mat 1 con 1 &

face id 2 &

8.18920370898699e+02 7.37329641873752e+02 3.85333219479087e+02 &

8.18483672968706e+02 7.37593060216477e+02 3.91417863514613e+02 &

8.22991116415336e+02 7.31153198242188e+02 3.85627454268575e+02 &

face id 2 &

8.18483672968706e+02 7.37593060216477e+02 3.91417863514613e+02 &

8.18920370898699e+02 7.37329641873752e+02 3.85333219479087e+02 &

8.25353446890711e+02 7.37840937237503e+02 3.89874328693950e+02 &

face id 2 &

8.22991116415336e+02 7.31153198242188e+02 3.85627454268575e+02 &

8.18483672968706e+02 7.37593060216477e+02 3.91417863514613e+02 &

8.25353446890711e+02 7.37840937237503e+02 3.89874328693950e+02 &

face id 2 &

8.18920370898699e+02 7.37329641873752e+02 3.85333219479087e+02 &

8.22991116415336e+02 7.31153198242188e+02 3.85627454268575e+02 &

8.25353446890711e+02 7.37840937237503e+02 3.89874328693950e+02

; block 93

poly group "10001" mat 1 con 1 &

face id 2 &

7.93126102700700e+02 7.39147352988408e+02 3.85920040094875e+02 &

7.91723693011833e+02 7.34565359260124e+02 3.87421651270315e+02 &

7.94608063469192e+02 7.35413562350436e+02 3.84880272566777e+02 &

face id 2 &

7.91723693011833e+02 7.34565359260124e+02 3.87421651270315e+02 &

7.93126102700700e+02 7.39147352988408e+02 3.85920040094875e+02 &

7.93075306390299e+02 7.37259553714495e+02 3.89912466417267e+02 &

face id 2 &

7.94608063469192e+02 7.35413562350436e+02 3.84880272566777e+02 &

7.91723693011833e+02 7.34565359260124e+02 3.87421651270315e+02 &

7.93075306390299e+02 7.37259553714495e+02 3.89912466417267e+02 &

face id 2 &

7.93126102700700e+02 7.39147352988408e+02 3.85920040094875e+02 &

7.94608063469192e+02 7.35413562350436e+02 3.84880272566777e+02 &

7.93075306390299e+02 7.37259553714495e+02 3.89912466417267e+02

; block 94

poly group "10001" mat 1 con 1 &

face id 2 &

8.21348267997198e+02 7.39634491253400e+02 4.42846308765791e+02 &

8.24231497582248e+02 7.39555545391830e+02 4.40655328606252e+02 &

8.22571342321449e+02 7.42957763671875e+02 4.41938917297102e+02 &

face id 2 &

8.24231497582248e+02 7.39555545391830e+02 4.40655328606252e+02 &

8.21348267997198e+02 7.39634491253400e+02 4.42846308765791e+02 &

8.24345516769850e+02 7.40101480767813e+02 4.44800655912060e+02 &

face id 2 &

8.22571342321449e+02 7.42957763671875e+02 4.41938917297102e+02 &

8.24231497582248e+02 7.39555545391830e+02 4.40655328606252e+02 &

8.24345516769850e+02 7.40101480767813e+02 4.44800655912060e+02 &

face id 2 &

8.21348267997198e+02 7.39634491253400e+02 4.42846308765791e+02 &

8.22571342321449e+02 7.42957763671875e+02 4.41938917297102e+02 &

8.24345516769850e+02 7.40101480767813e+02 4.44800655912060e+02

; block 95

poly group "10001" mat 1 con 1 &

face id 2 &

7.99664551669819e+02 7.38727869564032e+02 3.98609473831640e+02 &

7.95632045078570e+02 7.39829233879320e+02 4.01197287506287e+02 &

7.96021030652622e+02 7.39527701437256e+02 3.96689394205240e+02 &

face id 2 &

7.95632045078570e+02 7.39829233879320e+02 4.01197287506287e+02 &

7.99664551669819e+02 7.38727869564032e+02 3.98609473831640e+02 &

7.94943182185095e+02 7.36813761564512e+02 3.98851536379301e+02 &

face id 2 &

7.96021030652622e+02 7.39527701437256e+02 3.96689394205240e+02 &

7.95632045078570e+02 7.39829233879320e+02 4.01197287506287e+02 &

7.94943182185095e+02 7.36813761564512e+02 3.98851536379301e+02 &

face id 2 &

7.99664551669819e+02 7.38727869564032e+02 3.98609473831640e+02 &

7.96021030652622e+02 7.39527701437256e+02 3.96689394205240e+02 &

7.94943182185095e+02 7.36813761564512e+02 3.98851536379301e+02

; block 96

poly group "10001" mat 1 con 1 &

face id 2 &

8.39084289550781e+02 7.42957763671875e+02 4.43160901718362e+02 &

8.34694045880472e+02 7.42957763671875e+02 4.40556675161879e+02 &

8.39084289550781e+02 7.37053357299508e+02 4.39638740088288e+02 &

face id 2 &

8.34694045880472e+02 7.42957763671875e+02 4.40556675161879e+02 &

8.39084289550781e+02 7.42957763671875e+02 4.43160901718362e+02 &

8.34729885421035e+02 7.36525928807045e+02 4.41869552698341e+02 &

face id 2 &

8.39084289550781e+02 7.37053357299508e+02 4.39638740088288e+02 &

8.34694045880472e+02 7.42957763671875e+02 4.40556675161879e+02 &

8.34729885421035e+02 7.36525928807045e+02 4.41869552698341e+02 &

face id 2 &

8.39084289550781e+02 7.42957763671875e+02 4.43160901718362e+02 &

8.39084289550781e+02 7.37053357299508e+02 4.39638740088288e+02 &

8.34729885421035e+02 7.36525928807045e+02 4.41869552698341e+02

; block 97

poly group "10001" mat 1 con 1 &

face id 2 &

8.29691648616029e+02 7.42957763671875e+02 4.32536259090026e+02 &

8.27667286520472e+02 7.38125365214952e+02 4.29857973751925e+02 &

8.33935290810690e+02 7.42957763671875e+02 4.29387199865961e+02 &

face id 2 &

8.27667286520472e+02 7.38125365214952e+02 4.29857973751925e+02 &

8.29691648616029e+02 7.42957763671875e+02 4.32536259090026e+02 &

8.33864722379183e+02 7.39652653709875e+02 4.32623313919057e+02 &

face id 2 &

8.33935290810690e+02 7.42957763671875e+02 4.29387199865961e+02 &

8.27667286520472e+02 7.38125365214952e+02 4.29857973751925e+02 &

8.33864722379183e+02 7.39652653709875e+02 4.32623313919057e+02 &

face id 2 &

8.29691648616029e+02 7.42957763671875e+02 4.32536259090026e+02 &

8.33935290810690e+02 7.42957763671875e+02 4.29387199865961e+02 &

8.33864722379183e+02 7.39652653709875e+02 4.32623313919057e+02

; block 98

poly group "10001" mat 1 con 1 &

face id 2 &

7.97516497216007e+02 7.34563943186781e+02 3.97383221855559e+02 &

7.99664551669819e+02 7.38727869564032e+02 3.98609473831640e+02 &

7.96021030652622e+02 7.39527701437256e+02 3.96689394205240e+02 &

face id 2 &

7.99664551669819e+02 7.38727869564032e+02 3.98609473831640e+02 &

7.97516497216007e+02 7.34563943186781e+02 3.97383221855559e+02 &

7.94943182185095e+02 7.36813761564512e+02 3.98851536379301e+02 &

face id 2 &

7.96021030652622e+02 7.39527701437256e+02 3.96689394205240e+02 &

7.99664551669819e+02 7.38727869564032e+02 3.98609473831640e+02 &

7.94943182185095e+02 7.36813761564512e+02 3.98851536379301e+02 &

face id 2 &

7.97516497216007e+02 7.34563943186781e+02 3.97383221855559e+02 &

7.96021030652622e+02 7.39527701437256e+02 3.96689394205240e+02 &

7.94943182185095e+02 7.36813761564512e+02 3.98851536379301e+02

; block 99

poly group "10001" mat 1 con 1 &

face id 2 &

7.94608063469192e+02 7.35413562350436e+02 3.84880272566777e+02 &

7.91723693011833e+02 7.34565359260124e+02 3.87421651270315e+02 &

7.96012403601172e+02 7.34733593543699e+02 3.88834189030604e+02 &

face id 2 &

7.91723693011833e+02 7.34565359260124e+02 3.87421651270315e+02 &

7.94608063469192e+02 7.35413562350436e+02 3.84880272566777e+02 &

7.93075306390299e+02 7.37259553714495e+02 3.89912466417267e+02 &

face id 2 &

7.96012403601172e+02 7.34733593543699e+02 3.88834189030604e+02 &

7.91723693011833e+02 7.34565359260124e+02 3.87421651270315e+02 &

7.93075306390299e+02 7.37259553714495e+02 3.89912466417267e+02 &

face id 2 &

7.94608063469192e+02 7.35413562350436e+02 3.84880272566777e+02 &

7.96012403601172e+02 7.34733593543699e+02 3.88834189030604e+02 &

7.93075306390299e+02 7.37259553714495e+02 3.89912466417267e+02

; block 100

poly group "10001" mat 1 con 1 &

face id 2 &

8.18483672968706e+02 7.37593060216477e+02 3.91417863514613e+02 &

8.18920370898699e+02 7.37329641873752e+02 3.85333219479087e+02 &

8.19289966892218e+02 7.42957763671875e+02 3.85948308253048e+02 &

face id 2 &

8.18920370898699e+02 7.37329641873752e+02 3.85333219479087e+02 &

8.18483672968706e+02 7.37593060216477e+02 3.91417863514613e+02 &

8.25353446890711e+02 7.37840937237503e+02 3.89874328693950e+02 &

face id 2 &

8.19289966892218e+02 7.42957763671875e+02 3.85948308253048e+02 &

8.18920370898699e+02 7.37329641873752e+02 3.85333219479087e+02 &

8.25353446890711e+02 7.37840937237503e+02 3.89874328693950e+02 &

face id 2 &

8.18483672968706e+02 7.37593060216477e+02 3.91417863514613e+02 &

8.19289966892218e+02 7.42957763671875e+02 3.85948308253048e+02 &

8.25353446890711e+02 7.37840937237503e+02 3.89874328693950e+02

; block 101

poly group "10001" mat 1 con 1 &

face id 2 &

8.25353446890711e+02 7.37840937237503e+02 3.89874328693950e+02 &

8.23770891999958e+02 7.42957763671875e+02 3.89750043288969e+02 &

8.19289966892218e+02 7.42957763671875e+02 3.85948308253048e+02 &

face id 2 &

8.23770891999958e+02 7.42957763671875e+02 3.89750043288969e+02 &

8.25353446890711e+02 7.37840937237503e+02 3.89874328693950e+02 &

8.18483672968706e+02 7.37593060216477e+02 3.91417863514613e+02 &

face id 2 &

8.19289966892218e+02 7.42957763671875e+02 3.85948308253048e+02 &

8.23770891999958e+02 7.42957763671875e+02 3.89750043288969e+02 &

8.18483672968706e+02 7.37593060216477e+02 3.91417863514613e+02 &

face id 2 &

8.25353446890711e+02 7.37840937237503e+02 3.89874328693950e+02 &

8.19289966892218e+02 7.42957763671875e+02 3.85948308253048e+02 &

8.18483672968706e+02 7.37593060216477e+02 3.91417863514613e+02

; block 102

poly group "10001" mat 1 con 1 &

face id 2 &

8.20686199095838e+02 7.34536120483993e+02 4.38575068556780e+02 &

8.18750826609427e+02 7.36171568127837e+02 4.40122380486379e+02 &

8.22822252675870e+02 7.36637749364954e+02 4.41131123199765e+02 &

face id 2 &

8.18750826609427e+02 7.36171568127837e+02 4.40122380486379e+02 &

8.20686199095838e+02 7.34536120483993e+02 4.38575068556780e+02 &

8.21832695989208e+02 7.37054930723214e+02 4.37100055976185e+02 &

face id 2 &

8.22822252675870e+02 7.36637749364954e+02 4.41131123199765e+02 &

8.18750826609427e+02 7.36171568127837e+02 4.40122380486379e+02 &

8.21832695989208e+02 7.37054930723214e+02 4.37100055976185e+02 &

face id 2 &

8.20686199095838e+02 7.34536120483993e+02 4.38575068556780e+02 &

8.22822252675870e+02 7.36637749364954e+02 4.41131123199765e+02 &

8.21832695989208e+02 7.37054930723214e+02 4.37100055976185e+02

; block 103

poly group "10001" mat 1 con 1 &

face id 2 &

8.25973252814539e+02 7.42957763671875e+02 4.43890670479208e+02 &

8.27910339647761e+02 7.39308812863053e+02 4.44974237053554e+02 &

8.25193073523086e+02 7.42957763671875e+02 4.47593921454763e+02 &

face id 2 &

8.27910339647761e+02 7.39308812863053e+02 4.44974237053554e+02 &

8.25973252814539e+02 7.42957763671875e+02 4.43890670479208e+02 &

8.24345516769850e+02 7.40101480767813e+02 4.44800655912060e+02 &

face id 2 &

8.25193073523086e+02 7.42957763671875e+02 4.47593921454763e+02 &

8.27910339647761e+02 7.39308812863053e+02 4.44974237053554e+02 &

8.24345516769850e+02 7.40101480767813e+02 4.44800655912060e+02 &

face id 2 &

8.25973252814539e+02 7.42957763671875e+02 4.43890670479208e+02 &

8.25193073523086e+02 7.42957763671875e+02 4.47593921454763e+02 &

8.24345516769850e+02 7.40101480767813e+02 4.44800655912060e+02

; block 104

poly group "10001" mat 1 con 1 &

face id 2 &

8.15793233645232e+02 7.34747728264843e+02 4.35883786023879e+02 &

8.18363373352647e+02 7.37289189498031e+02 4.37173452288585e+02 &

8.19270705387996e+02 7.33695343369261e+02 4.35937073693200e+02 &

face id 2 &

8.18363373352647e+02 7.37289189498031e+02 4.37173452288585e+02 &

8.15793233645232e+02 7.34747728264843e+02 4.35883786023879e+02 &

8.18859811694220e+02 7.36907823701414e+02 4.33778095113987e+02 &

face id 2 &

8.19270705387996e+02 7.33695343369261e+02 4.35937073693200e+02 &

8.18363373352647e+02 7.37289189498031e+02 4.37173452288585e+02 &

8.18859811694220e+02 7.36907823701414e+02 4.33778095113987e+02 &

face id 2 &

8.15793233645232e+02 7.34747728264843e+02 4.35883786023879e+02 &

8.19270705387996e+02 7.33695343369261e+02 4.35937073693200e+02 &

8.18859811694220e+02 7.36907823701414e+02 4.33778095113987e+02

; block 105

poly group "10001" mat 1 con 1 &

face id 1 &

8.23502394316352e+02 7.41141241547762e+02 4.46889342786744e+02 &

8.26644054329157e+02 7.40940525295196e+02 4.49188869423419e+02 &

8.25450073242188e+02 7.38673095703125e+02 4.48417846679688e+02 &

face id 2 &

8.26644054329157e+02 7.40940525295196e+02 4.49188869423419e+02 &

8.23502394316352e+02 7.41141241547762e+02 4.46889342786744e+02 &

8.24345516769850e+02 7.40101480767813e+02 4.44800655912060e+02 &

face id 2 &

8.25450073242188e+02 7.38673095703125e+02 4.48417846679688e+02 &

8.26644054329157e+02 7.40940525295196e+02 4.49188869423419e+02 &

8.24345516769850e+02 7.40101480767813e+02 4.44800655912060e+02 &

face id 2 &

8.23502394316352e+02 7.41141241547762e+02 4.46889342786744e+02 &

8.25450073242188e+02 7.38673095703125e+02 4.48417846679688e+02 &

8.24345516769850e+02 7.40101480767813e+02 4.44800655912060e+02

; block 106

poly group "10001" mat 1 con 1 &

face id 2 &

8.26644054329157e+02 7.40940525295196e+02 4.49188869423419e+02 &

8.27910339647761e+02 7.39308812863053e+02 4.44974237053554e+02 &

8.25450073242188e+02 7.38673095703125e+02 4.48417846679688e+02 &

face id 2 &

8.27910339647761e+02 7.39308812863053e+02 4.44974237053554e+02 &

8.26644054329157e+02 7.40940525295196e+02 4.49188869423419e+02 &

8.24345516769850e+02 7.40101480767813e+02 4.44800655912060e+02 &

face id 2 &

8.25450073242188e+02 7.38673095703125e+02 4.48417846679688e+02 &

8.27910339647761e+02 7.39308812863053e+02 4.44974237053554e+02 &

8.24345516769850e+02 7.40101480767813e+02 4.44800655912060e+02 &

face id 2 &

8.26644054329157e+02 7.40940525295196e+02 4.49188869423419e+02 &

8.25450073242188e+02 7.38673095703125e+02 4.48417846679688e+02 &

8.24345516769850e+02 7.40101480767813e+02 4.44800655912060e+02

; block 107

poly group "10001" mat 1 con 1 &

face id 1 &

8.26644054329157e+02 7.40940525295196e+02 4.49188869423419e+02 &

8.23502394316352e+02 7.41141241547762e+02 4.46889342786744e+02 &

8.25193073523086e+02 7.42957763671875e+02 4.47593921454763e+02 &

face id 2 &

8.23502394316352e+02 7.41141241547762e+02 4.46889342786744e+02 &

8.26644054329157e+02 7.40940525295196e+02 4.49188869423419e+02 &

8.24345516769850e+02 7.40101480767813e+02 4.44800655912060e+02 &

face id 2 &

8.25193073523086e+02 7.42957763671875e+02 4.47593921454763e+02 &

8.23502394316352e+02 7.41141241547762e+02 4.46889342786744e+02 &

8.24345516769850e+02 7.40101480767813e+02 4.44800655912060e+02 &

face id 2 &

8.26644054329157e+02 7.40940525295196e+02 4.49188869423419e+02 &

8.25193073523086e+02 7.42957763671875e+02 4.47593921454763e+02 &

8.24345516769850e+02 7.40101480767813e+02 4.44800655912060e+02

; block 108

poly group "10001" mat 1 con 1 &

face id 2 &

8.12647078267193e+02 7.38844593444619e+02 4.17279149585504e+02 &

8.10593672000049e+02 7.39659613160195e+02 4.21874911950142e+02 &

8.08606526611431e+02 7.39255690450706e+02 4.17166663969857e+02 &

face id 2 &

8.10593672000049e+02 7.39659613160195e+02 4.21874911950142e+02 &

8.12647078267193e+02 7.38844593444619e+02 4.17279149585504e+02 &

8.09490919631596e+02 7.36167724749901e+02 4.19676489619966e+02 &

face id 2 &

8.08606526611431e+02 7.39255690450706e+02 4.17166663969857e+02 &

8.10593672000049e+02 7.39659613160195e+02 4.21874911950142e+02 &

8.09490919631596e+02 7.36167724749901e+02 4.19676489619966e+02 &

face id 2 &

8.12647078267193e+02 7.38844593444619e+02 4.17279149585504e+02 &

8.08606526611431e+02 7.39255690450706e+02 4.17166663969857e+02 &

8.09490919631596e+02 7.36167724749901e+02 4.19676489619966e+02

; block 109

poly group "10001" mat 1 con 1 &

face id 2 &

8.18363373352647e+02 7.37289189498031e+02 4.37173452288585e+02 &

8.18750826609427e+02 7.36171568127837e+02 4.40122380486379e+02 &

8.20686199095838e+02 7.34536120483993e+02 4.38575068556780e+02 &

face id 2 &

8.18750826609427e+02 7.36171568127837e+02 4.40122380486379e+02 &

8.18363373352647e+02 7.37289189498031e+02 4.37173452288585e+02 &

8.21832695989208e+02 7.37054930723214e+02 4.37100055976185e+02 &

face id 2 &

8.20686199095838e+02 7.34536120483993e+02 4.38575068556780e+02 &

8.18750826609427e+02 7.36171568127837e+02 4.40122380486379e+02 &

8.21832695989208e+02 7.37054930723214e+02 4.37100055976185e+02 &

face id 2 &

8.18363373352647e+02 7.37289189498031e+02 4.37173452288585e+02 &

8.20686199095838e+02 7.34536120483993e+02 4.38575068556780e+02 &

8.21832695989208e+02 7.37054930723214e+02 4.37100055976185e+02

; block 110

poly group "10001" mat 1 con 1 &

face id 2 &

8.08037597656250e+02 7.37380371093750e+02 4.23707122802734e+02 &

8.11328747716264e+02 7.37004144157769e+02 4.23930341163385e+02 &

8.08622778697454e+02 7.33969474930182e+02 4.21836602431366e+02 &

face id 2 &

8.11328747716264e+02 7.37004144157769e+02 4.23930341163385e+02 &

8.08037597656250e+02 7.37380371093750e+02 4.23707122802734e+02 &

8.09490919631596e+02 7.36167724749901e+02 4.19676489619966e+02 &

face id 2 &

8.08622778697454e+02 7.33969474930182e+02 4.21836602431366e+02 &

8.11328747716264e+02 7.37004144157769e+02 4.23930341163385e+02 &

8.09490919631596e+02 7.36167724749901e+02 4.19676489619966e+02 &

face id 2 &

8.08037597656250e+02 7.37380371093750e+02 4.23707122802734e+02 &

8.08622778697454e+02 7.33969474930182e+02 4.21836602431366e+02 &

8.09490919631596e+02 7.36167724749901e+02 4.19676489619966e+02

; block 111

poly group "10001" mat 1 con 1 &

face id 2 &

8.11726863481470e+02 7.42957763671875e+02 4.05255074248258e+02 &

8.14194519614525e+02 7.36222240308663e+02 4.03223835774641e+02 &

8.18069174339446e+02 7.42957763671875e+02 4.01829283575629e+02 &

face id 2 &

8.14194519614525e+02 7.36222240308663e+02 4.03223835774641e+02 &

8.11726863481470e+02 7.42957763671875e+02 4.05255074248258e+02 &

8.18044212102941e+02 7.37294444794664e+02 4.06947605911675e+02 &

face id 2 &

8.18069174339446e+02 7.42957763671875e+02 4.01829283575629e+02 &

8.14194519614525e+02 7.36222240308663e+02 4.03223835774641e+02 &

8.18044212102941e+02 7.37294444794664e+02 4.06947605911675e+02 &

face id 2 &

8.11726863481470e+02 7.42957763671875e+02 4.05255074248258e+02 &

8.18069174339446e+02 7.42957763671875e+02 4.01829283575629e+02 &

8.18044212102941e+02 7.37294444794664e+02 4.06947605911675e+02

; block 112

poly group "10001" mat 1 con 1 &

face id 2 &

7.77400463245723e+02 7.38786178236515e+02 3.64848876953125e+02 &

7.80313712190835e+02 7.35222135995747e+02 3.64848876953125e+02 &

7.79101118006749e+02 7.34898937065474e+02 3.69554192649387e+02 &

face id 1 &

7.80313712190835e+02 7.35222135995747e+02 3.64848876953125e+02 &

7.77400463245723e+02 7.38786178236515e+02 3.64848876953125e+02 &

7.74873482850570e+02 7.34379916376657e+02 3.64848876953125e+02 &

face id 2 &

7.79101118006749e+02 7.34898937065474e+02 3.69554192649387e+02 &

7.80313712190835e+02 7.35222135995747e+02 3.64848876953125e+02 &

7.74873482850570e+02 7.34379916376657e+02 3.64848876953125e+02 &

face id 2 &

7.77400463245723e+02 7.38786178236515e+02 3.64848876953125e+02 &

7.79101118006749e+02 7.34898937065474e+02 3.69554192649387e+02 &

7.74873482850570e+02 7.34379916376657e+02 3.64848876953125e+02

; block 113

poly group "10001" mat 1 con 1 &

face id 2 &

8.10593672000049e+02 7.39659613160195e+02 4.21874911950142e+02 &

8.11328747716264e+02 7.37004144157769e+02 4.23930341163385e+02 &

8.08037597656250e+02 7.37380371093750e+02 4.23707122802734e+02 &

face id 2 &

8.11328747716264e+02 7.37004144157769e+02 4.23930341163385e+02 &

8.10593672000049e+02 7.39659613160195e+02 4.21874911950142e+02 &

8.09490919631596e+02 7.36167724749901e+02 4.19676489619966e+02 &

face id 2 &

8.08037597656250e+02 7.37380371093750e+02 4.23707122802734e+02 &

8.11328747716264e+02 7.37004144157769e+02 4.23930341163385e+02 &

8.09490919631596e+02 7.36167724749901e+02 4.19676489619966e+02 &

face id 2 &

8.10593672000049e+02 7.39659613160195e+02 4.21874911950142e+02 &

8.08037597656250e+02 7.37380371093750e+02 4.23707122802734e+02 &

8.09490919631596e+02 7.36167724749901e+02 4.19676489619966e+02

; block 114

poly group "10001" mat 1 con 1 &

face id 2 &

8.06053783905492e+02 7.39353100736547e+02 4.13045444506629e+02 &

8.09911809905185e+02 7.38789513583694e+02 4.13229940854814e+02 &

8.07792915601657e+02 7.42957763671875e+02 4.14815876761776e+02 &

face id 2 &

8.09911809905185e+02 7.38789513583694e+02 4.13229940854814e+02 &

8.06053783905492e+02 7.39353100736547e+02 4.13045444506629e+02 &

8.08606526611431e+02 7.39255690450706e+02 4.17166663969857e+02 &

face id 2 &

8.07792915601657e+02 7.42957763671875e+02 4.14815876761776e+02 &

8.09911809905185e+02 7.38789513583694e+02 4.13229940854814e+02 &

8.08606526611431e+02 7.39255690450706e+02 4.17166663969857e+02 &

face id 2 &

8.06053783905492e+02 7.39353100736547e+02 4.13045444506629e+02 &

8.07792915601657e+02 7.42957763671875e+02 4.14815876761776e+02 &

8.08606526611431e+02 7.39255690450706e+02 4.17166663969857e+02

; block 115

poly group "10001" mat 1 con 1 &

face id 2 &

8.14838076915517e+02 7.39071150525117e+02 4.39175800078397e+02 &

8.16269421594957e+02 7.36054575659189e+02 4.39716662360012e+02 &

8.16834488631438e+02 7.39654348453849e+02 4.36643480628616e+02 &

face id 2 &

8.16269421594957e+02 7.36054575659189e+02 4.39716662360012e+02 &

8.14838076915517e+02 7.39071150525117e+02 4.39175800078397e+02 &

8.16975670140343e+02 7.39892790962574e+02 4.39960247842898e+02 &

face id 2 &

8.16834488631438e+02 7.39654348453849e+02 4.36643480628616e+02 &

8.16269421594957e+02 7.36054575659189e+02 4.39716662360012e+02 &

8.16975670140343e+02 7.39892790962574e+02 4.39960247842898e+02 &

face id 2 &

8.14838076915517e+02 7.39071150525117e+02 4.39175800078397e+02 &

8.16834488631438e+02 7.39654348453849e+02 4.36643480628616e+02 &

8.16975670140343e+02 7.39892790962574e+02 4.39960247842898e+02

; block 116

poly group "10001" mat 1 con 1 &

face id 2 &

8.21044537549873e+02 7.31153198242188e+02 4.36848956154312e+02 &

8.23363932724725e+02 7.33802420140527e+02 4.37276399935772e+02 &

8.24135792527692e+02 7.31153198242188e+02 4.34857938641253e+02 &

face id 2 &

8.23363932724725e+02 7.33802420140527e+02 4.37276399935772e+02 &

8.21044537549873e+02 7.31153198242188e+02 4.36848956154312e+02 &

8.22071412209386e+02 7.34221053581135e+02 4.34081587769800e+02 &

face id 2 &

8.24135792527692e+02 7.31153198242188e+02 4.34857938641253e+02 &

8.23363932724725e+02 7.33802420140527e+02 4.37276399935772e+02 &

8.22071412209386e+02 7.34221053581135e+02 4.34081587769800e+02 &

face id 2 &

8.21044537549873e+02 7.31153198242188e+02 4.36848956154312e+02 &

8.24135792527692e+02 7.31153198242188e+02 4.34857938641253e+02 &

8.22071412209386e+02 7.34221053581135e+02 4.34081587769800e+02

; block 117

poly group "10001" mat 1 con 1 &

face id 2 &

8.16269421594957e+02 7.36054575659189e+02 4.39716662360012e+02 &

8.15793233645232e+02 7.34747728264843e+02 4.35883786023879e+02 &

8.14183182034257e+02 7.37171551191613e+02 4.36783264905590e+02 &

face id 2 &

8.15793233645232e+02 7.34747728264843e+02 4.35883786023879e+02 &

8.16269421594957e+02 7.36054575659189e+02 4.39716662360012e+02 &

8.18363373352647e+02 7.37289189498031e+02 4.37173452288585e+02 &

face id 2 &

8.14183182034257e+02 7.37171551191613e+02 4.36783264905590e+02 &

8.15793233645232e+02 7.34747728264843e+02 4.35883786023879e+02 &

8.18363373352647e+02 7.37289189498031e+02 4.37173452288585e+02 &

face id 2 &

8.16269421594957e+02 7.36054575659189e+02 4.39716662360012e+02 &

8.14183182034257e+02 7.37171551191613e+02 4.36783264905590e+02 &

8.18363373352647e+02 7.37289189498031e+02 4.37173452288585e+02

; block 118

poly group "10001" mat 1 con 1 &

face id 1 &

8.17436891461985e+02 7.31153198242188e+02 4.30682311023628e+02 &

8.14712710670199e+02 7.31153198242188e+02 4.31987832312421e+02 &

8.16297973632812e+02 7.31153198242188e+02 4.34297637939453e+02 &

face id 2 &

8.14712710670199e+02 7.31153198242188e+02 4.31987832312421e+02 &

8.17436891461985e+02 7.31153198242188e+02 4.30682311023628e+02 &

8.17450431002428e+02 7.33768641315643e+02 4.33222124331104e+02 &

face id 2 &

8.16297973632812e+02 7.31153198242188e+02 4.34297637939453e+02 &

8.14712710670199e+02 7.31153198242188e+02 4.31987832312421e+02 &

8.17450431002428e+02 7.33768641315643e+02 4.33222124331104e+02 &

face id 2 &

8.17436891461985e+02 7.31153198242188e+02 4.30682311023628e+02 &

8.16297973632812e+02 7.31153198242188e+02 4.34297637939453e+02 &

8.17450431002428e+02 7.33768641315643e+02 4.33222124331104e+02

; block 119

poly group "10001" mat 1 con 1 &

face id 1 &

7.99156871754180e+02 7.42957763671875e+02 3.74726900763980e+02 &

8.05448465309216e+02 7.42957763671875e+02 3.76149659904077e+02 &

8.00154378440319e+02 7.42957763671875e+02 3.81345085869093e+02 &

face id 2 &

8.05448465309216e+02 7.42957763671875e+02 3.76149659904077e+02 &

7.99156871754180e+02 7.42957763671875e+02 3.74726900763980e+02 &

8.04135590675662e+02 7.37036903814702e+02 3.79060658468265e+02 &

face id 2 &

8.00154378440319e+02 7.42957763671875e+02 3.81345085869093e+02 &

8.05448465309216e+02 7.42957763671875e+02 3.76149659904077e+02 &

8.04135590675662e+02 7.37036903814702e+02 3.79060658468265e+02 &

face id 2 &

7.99156871754180e+02 7.42957763671875e+02 3.74726900763980e+02 &

8.00154378440319e+02 7.42957763671875e+02 3.81345085869093e+02 &

8.04135590675662e+02 7.37036903814702e+02 3.79060658468265e+02

; block 120

poly group "10001" mat 1 con 1 &

face id 2 &

8.15090273562936e+02 7.34284678180158e+02 4.30425431650755e+02 &

8.14712710670199e+02 7.31153198242188e+02 4.31987832312421e+02 &

8.17436891461985e+02 7.31153198242188e+02 4.30682311023628e+02 &

face id 2 &

8.14712710670199e+02 7.31153198242188e+02 4.31987832312421e+02 &

8.15090273562936e+02 7.34284678180158e+02 4.30425431650755e+02 &

8.17450431002428e+02 7.33768641315643e+02 4.33222124331104e+02 &

face id 2 &

8.17436891461985e+02 7.31153198242188e+02 4.30682311023628e+02 &

8.14712710670199e+02 7.31153198242188e+02 4.31987832312421e+02 &

8.17450431002428e+02 7.33768641315643e+02 4.33222124331104e+02 &

face id 2 &

8.15090273562936e+02 7.34284678180158e+02 4.30425431650755e+02 &

8.17436891461985e+02 7.31153198242188e+02 4.30682311023628e+02 &

8.17450431002428e+02 7.33768641315643e+02 4.33222124331104e+02

; block 121

poly group "10001" mat 1 con 1 &

face id 1 &

8.00423884947867e+02 7.31153198242188e+02 3.87599039819564e+02 &

8.00965458695504e+02 7.31153198242188e+02 3.91758115901608e+02 &

8.05202148637845e+02 7.31153198242188e+02 3.89338513210927e+02 &

face id 2 &

8.00965458695504e+02 7.31153198242188e+02 3.91758115901608e+02 &

8.00423884947867e+02 7.31153198242188e+02 3.87599039819564e+02 &

8.02809345752658e+02 7.35127916281908e+02 3.91487446480074e+02 &

face id 2 &

8.05202148637845e+02 7.31153198242188e+02 3.89338513210927e+02 &

8.00965458695504e+02 7.31153198242188e+02 3.91758115901608e+02 &

8.02809345752658e+02 7.35127916281908e+02 3.91487446480074e+02 &

face id 2 &

8.00423884947867e+02 7.31153198242188e+02 3.87599039819564e+02 &

8.05202148637845e+02 7.31153198242188e+02 3.89338513210927e+02 &

8.02809345752658e+02 7.35127916281908e+02 3.91487446480074e+02

; block 122

poly group "10001" mat 1 con 1 &

face id 2 &

8.15105453724136e+02 7.42957763671875e+02 4.24551856435235e+02 &

8.18126133140210e+02 7.39128284797895e+02 4.24521969903783e+02 &

8.14891237294761e+02 7.42957763671875e+02 4.28254850378271e+02 &

face id 2 &

8.18126133140210e+02 7.39128284797895e+02 4.24521969903783e+02 &

8.15105453724136e+02 7.42957763671875e+02 4.24551856435235e+02 &

8.13756389123963e+02 7.39589893800934e+02 4.25916239240713e+02 &

face id 2 &

8.14891237294761e+02 7.42957763671875e+02 4.28254850378271e+02 &

8.18126133140210e+02 7.39128284797895e+02 4.24521969903783e+02 &

8.13756389123963e+02 7.39589893800934e+02 4.25916239240713e+02 &

face id 2 &

8.15105453724136e+02 7.42957763671875e+02 4.24551856435235e+02 &

8.14891237294761e+02 7.42957763671875e+02 4.28254850378271e+02 &

8.13756389123963e+02 7.39589893800934e+02 4.25916239240713e+02

; block 123

poly group "10001" mat 1 con 1 &

face id 1 &

8.15105453724136e+02 7.42957763671875e+02 4.24551856435235e+02 &

8.11683577279594e+02 7.42957763671875e+02 4.26990978647697e+02 &

8.10989743959868e+02 7.42957763671875e+02 4.23463792596016e+02 &

face id 2 &

8.11683577279594e+02 7.42957763671875e+02 4.26990978647697e+02 &

8.15105453724136e+02 7.42957763671875e+02 4.24551856435235e+02 &

8.13756389123963e+02 7.39589893800934e+02 4.25916239240713e+02 &

face id 2 &

8.10989743959868e+02 7.42957763671875e+02 4.23463792596016e+02 &

8.11683577279594e+02 7.42957763671875e+02 4.26990978647697e+02 &

8.13756389123963e+02 7.39589893800934e+02 4.25916239240713e+02 &

face id 2 &

8.15105453724136e+02 7.42957763671875e+02 4.24551856435235e+02 &

8.10989743959868e+02 7.42957763671875e+02 4.23463792596016e+02 &

8.13756389123963e+02 7.39589893800934e+02 4.25916239240713e+02

; block 124

poly group "10001" mat 1 con 1 &

face id 1 &

8.00965458695504e+02 7.31153198242188e+02 3.91758115901608e+02 &

8.01878943722119e+02 7.31153198242188e+02 3.95977524084350e+02 &

8.04638018864358e+02 7.31153198242188e+02 3.93249980696247e+02 &

face id 2 &

8.01878943722119e+02 7.31153198242188e+02 3.95977524084350e+02 &

8.00965458695504e+02 7.31153198242188e+02 3.91758115901608e+02 &

8.00446633691467e+02 7.34890585746234e+02 3.94456651409666e+02 &

face id 2 &

8.04638018864358e+02 7.31153198242188e+02 3.93249980696247e+02 &

8.01878943722119e+02 7.31153198242188e+02 3.95977524084350e+02 &

8.00446633691467e+02 7.34890585746234e+02 3.94456651409666e+02 &

face id 2 &

8.00965458695504e+02 7.31153198242188e+02 3.91758115901608e+02 &

8.04638018864358e+02 7.31153198242188e+02 3.93249980696247e+02 &

8.00446633691467e+02 7.34890585746234e+02 3.94456651409666e+02

; block 125

poly group "10001" mat 1 con 1 &

face id 1 &

7.88920547739239e+02 7.37839599356893e+02 3.99365105432018e+02 &

7.87969075995420e+02 7.36014520892453e+02 3.97897194333784e+02 &

7.88326407845862e+02 7.38079130393022e+02 3.96095971328112e+02 &

face id 2 &

7.87969075995420e+02 7.36014520892453e+02 3.97897194333784e+02 &

7.88920547739239e+02 7.37839599356893e+02 3.99365105432018e+02 &

7.91408350694546e+02 7.37046149302478e+02 3.97065808643254e+02 &

face id 2 &

7.88326407845862e+02 7.38079130393022e+02 3.96095971328112e+02 &

7.87969075995420e+02 7.36014520892453e+02 3.97897194333784e+02 &

7.91408350694546e+02 7.37046149302478e+02 3.97065808643254e+02 &

face id 2 &

7.88920547739239e+02 7.37839599356893e+02 3.99365105432018e+02 &

7.88326407845862e+02 7.38079130393022e+02 3.96095971328112e+02 &

7.91408350694546e+02 7.37046149302478e+02 3.97065808643254e+02

; block 126

poly group "10001" mat 1 con 1 &

face id 2 &

7.80785664505661e+02 7.39236281877246e+02 3.73124042480188e+02 &

7.83243069165867e+02 7.42957763671875e+02 3.72786606032438e+02 &

7.82723936045558e+02 7.42957763671875e+02 3.77023845732179e+02 &

face id 2 &

7.83243069165867e+02 7.42957763671875e+02 3.72786606032438e+02 &

7.80785664505661e+02 7.39236281877246e+02 3.73124042480188e+02 &

7.83813396428186e+02 7.39314645759285e+02 3.75273836784356e+02 &

face id 2 &

7.82723936045558e+02 7.42957763671875e+02 3.77023845732179e+02 &

7.83243069165867e+02 7.42957763671875e+02 3.72786606032438e+02 &

7.83813396428186e+02 7.39314645759285e+02 3.75273836784356e+02 &

face id 2 &

7.80785664505661e+02 7.39236281877246e+02 3.73124042480188e+02 &

7.82723936045558e+02 7.42957763671875e+02 3.77023845732179e+02 &

7.83813396428186e+02 7.39314645759285e+02 3.75273836784356e+02

; block 127

poly group "10001" mat 1 con 1 &

face id 2 &

7.95525613451117e+02 7.34053194520976e+02 4.00352038635573e+02 &

7.92440189486717e+02 7.33520249849107e+02 4.00528860955446e+02 &

7.92491426636271e+02 7.36717278666031e+02 4.00346859894982e+02 &

face id 2 &

7.92440189486717e+02 7.33520249849107e+02 4.00528860955446e+02 &

7.95525613451117e+02 7.34053194520976e+02 4.00352038635573e+02 &

7.93571382806349e+02 7.34474319806959e+02 3.97972750447882e+02 &

face id 2 &

7.92491426636271e+02 7.36717278666031e+02 4.00346859894982e+02 &

7.92440189486717e+02 7.33520249849107e+02 4.00528860955446e+02 &

7.93571382806349e+02 7.34474319806959e+02 3.97972750447882e+02 &

face id 2 &

7.95525613451117e+02 7.34053194520976e+02 4.00352038635573e+02 &

7.92491426636271e+02 7.36717278666031e+02 4.00346859894982e+02 &

7.93571382806349e+02 7.34474319806959e+02 3.97972750447882e+02

; block 128

poly group "10001" mat 1 con 1 &

face id 1 &

7.88322365919560e+02 7.31153198242188e+02 3.94575346324754e+02 &

7.90665610154540e+02 7.31153198242188e+02 3.92006573131752e+02 &

7.87324272338391e+02 7.31153198242188e+02 3.91428677099727e+02 &

face id 2 &

7.90665610154540e+02 7.31153198242188e+02 3.92006573131752e+02 &

7.88322365919560e+02 7.31153198242188e+02 3.94575346324754e+02 &

7.90048611720629e+02 7.34003260059407e+02 3.93926493742337e+02 &

face id 2 &

7.87324272338391e+02 7.31153198242188e+02 3.91428677099727e+02 &

7.90665610154540e+02 7.31153198242188e+02 3.92006573131752e+02 &

7.90048611720629e+02 7.34003260059407e+02 3.93926493742337e+02 &

face id 2 &

7.88322365919560e+02 7.31153198242188e+02 3.94575346324754e+02 &

7.87324272338391e+02 7.31153198242188e+02 3.91428677099727e+02 &

7.90048611720629e+02 7.34003260059407e+02 3.93926493742337e+02

; block 129

poly group "10001" mat 1 con 1 &

face id 2 &

7.86042239988681e+02 7.34167137613057e+02 3.92935837866163e+02 &

7.88322365919560e+02 7.31153198242188e+02 3.94575346324754e+02 &

7.87324272338391e+02 7.31153198242188e+02 3.91428677099727e+02 &

face id 2 &

7.88322365919560e+02 7.31153198242188e+02 3.94575346324754e+02 &

7.86042239988681e+02 7.34167137613057e+02 3.92935837866163e+02 &

7.90048611720629e+02 7.34003260059407e+02 3.93926493742337e+02 &

face id 2 &

7.87324272338391e+02 7.31153198242188e+02 3.91428677099727e+02 &

7.88322365919560e+02 7.31153198242188e+02 3.94575346324754e+02 &

7.90048611720629e+02 7.34003260059407e+02 3.93926493742337e+02 &

face id 2 &

7.86042239988681e+02 7.34167137613057e+02 3.92935837866163e+02 &

7.87324272338391e+02 7.31153198242188e+02 3.91428677099727e+02 &

7.90048611720629e+02 7.34003260059407e+02 3.93926493742337e+02

; block 130

poly group "10001" mat 1 con 1 &

face id 1 &

8.33732177221493e+02 7.42957763671875e+02 4.22767791864061e+02 &

8.33324474406425e+02 7.42957763671875e+02 4.15623786886125e+02 &

8.39084289550781e+02 7.42957763671875e+02 4.19336260947644e+02 &

face id 2 &

8.33324474406425e+02 7.42957763671875e+02 4.15623786886125e+02 &

8.33732177221493e+02 7.42957763671875e+02 4.22767791864061e+02 &

8.31592196255603e+02 7.37553570391624e+02 4.20696295870736e+02 &

face id 2 &

8.39084289550781e+02 7.42957763671875e+02 4.19336260947644e+02 &

8.33324474406425e+02 7.42957763671875e+02 4.15623786886125e+02 &

8.31592196255603e+02 7.37553570391624e+02 4.20696295870736e+02 &

face id 2 &

8.33732177221493e+02 7.42957763671875e+02 4.22767791864061e+02 &

8.39084289550781e+02 7.42957763671875e+02 4.19336260947644e+02 &

8.31592196255603e+02 7.37553570391624e+02 4.20696295870736e+02

; block 131

poly group "10001" mat 1 con 1 &

face id 2 &

7.79552550043064e+02 7.34262768372563e+02 3.83255082743573e+02 &

7.81100846896327e+02 7.31153198242188e+02 3.81735958194611e+02 &

7.78389795522681e+02 7.31153198242188e+02 3.79403540956464e+02 &

face id 2 &

7.81100846896327e+02 7.31153198242188e+02 3.81735958194611e+02 &

7.79552550043064e+02 7.34262768372563e+02 3.83255082743573e+02 &

7.79511950549799e+02 7.34457282336568e+02 3.78709527306644e+02 &

face id 2 &

7.78389795522681e+02 7.31153198242188e+02 3.79403540956464e+02 &

7.81100846896327e+02 7.31153198242188e+02 3.81735958194611e+02 &

7.79511950549799e+02 7.34457282336568e+02 3.78709527306644e+02 &

face id 2 &

7.79552550043064e+02 7.34262768372563e+02 3.83255082743573e+02 &

7.78389795522681e+02 7.31153198242188e+02 3.79403540956464e+02 &

7.79511950549799e+02 7.34457282336568e+02 3.78709527306644e+02

; block 132

poly group "10001" mat 1 con 1 &

face id 2 &

8.19582997168244e+02 7.40188540403784e+02 4.34477675949180e+02 &

8.15566677879630e+02 7.39926095892137e+02 4.33708679063442e+02 &

8.18073172819596e+02 7.39472123297221e+02 4.29992682558518e+02 &

face id 2 &

8.15566677879630e+02 7.39926095892137e+02 4.33708679063442e+02 &

8.19582997168244e+02 7.40188540403784e+02 4.34477675949180e+02 &

8.18859811694220e+02 7.36907823701414e+02 4.33778095113987e+02 &

face id 2 &

8.18073172819596e+02 7.39472123297221e+02 4.29992682558518e+02 &

8.15566677879630e+02 7.39926095892137e+02 4.33708679063442e+02 &

8.18859811694220e+02 7.36907823701414e+02 4.33778095113987e+02 &

face id 2 &

8.19582997168244e+02 7.40188540403784e+02 4.34477675949180e+02 &

8.18073172819596e+02 7.39472123297221e+02 4.29992682558518e+02 &

8.18859811694220e+02 7.36907823701414e+02 4.33778095113987e+02

; block 133

poly group "10001" mat 1 con 1 &

face id 2 &

8.22071412209386e+02 7.34221053581135e+02 4.34081587769800e+02 &

8.18859811694220e+02 7.36907823701414e+02 4.33778095113987e+02 &

8.19270705387996e+02 7.33695343369261e+02 4.35937073693200e+02 &

face id 2 &

8.18859811694220e+02 7.36907823701414e+02 4.33778095113987e+02 &

8.22071412209386e+02 7.34221053581135e+02 4.34081587769800e+02 &

8.21832695989208e+02 7.37054930723214e+02 4.37100055976185e+02 &

face id 2 &

8.19270705387996e+02 7.33695343369261e+02 4.35937073693200e+02 &

8.18859811694220e+02 7.36907823701414e+02 4.33778095113987e+02 &

8.21832695989208e+02 7.37054930723214e+02 4.37100055976185e+02 &

face id 2 &

8.22071412209386e+02 7.34221053581135e+02 4.34081587769800e+02 &

8.19270705387996e+02 7.33695343369261e+02 4.35937073693200e+02 &

8.21832695989208e+02 7.37054930723214e+02 4.37100055976185e+02

; block 134

poly group "10001" mat 1 con 1 &

face id 1 &

8.33324474406425e+02 7.42957763671875e+02 4.15623786886125e+02 &

8.33732177221493e+02 7.42957763671875e+02 4.22767791864061e+02 &

8.28202156483526e+02 7.42957763671875e+02 4.19788988709625e+02 &

face id 2 &

8.33732177221493e+02 7.42957763671875e+02 4.22767791864061e+02 &

8.33324474406425e+02 7.42957763671875e+02 4.15623786886125e+02 &

8.31592196255603e+02 7.37553570391624e+02 4.20696295870736e+02 &

face id 2 &

8.28202156483526e+02 7.42957763671875e+02 4.19788988709625e+02 &

8.33732177221493e+02 7.42957763671875e+02 4.22767791864061e+02 &

8.31592196255603e+02 7.37553570391624e+02 4.20696295870736e+02 &

face id 2 &

8.33324474406425e+02 7.42957763671875e+02 4.15623786886125e+02 &

8.28202156483526e+02 7.42957763671875e+02 4.19788988709625e+02 &

8.31592196255603e+02 7.37553570391624e+02 4.20696295870736e+02

; block 135

poly group "10001" mat 1 con 1 &

face id 1 &

8.18663640401986e+02 7.31153198242188e+02 4.20323564658305e+02 &

8.19981081416289e+02 7.31153198242188e+02 4.24737298340724e+02 &

8.22847947705952e+02 7.31153198242188e+02 4.22600298353562e+02 &

face id 2 &

8.19981081416289e+02 7.31153198242188e+02 4.24737298340724e+02 &

8.18663640401986e+02 7.31153198242188e+02 4.20323564658305e+02 &

8.21662862454680e+02 7.34770629444877e+02 4.24506602520138e+02 &

face id 2 &

8.22847947705952e+02 7.31153198242188e+02 4.22600298353562e+02 &

8.19981081416289e+02 7.31153198242188e+02 4.24737298340724e+02 &

8.21662862454680e+02 7.34770629444877e+02 4.24506602520138e+02 &

face id 2 &

8.18663640401986e+02 7.31153198242188e+02 4.20323564658305e+02 &

8.22847947705952e+02 7.31153198242188e+02 4.22600298353562e+02 &

8.21662862454680e+02 7.34770629444877e+02 4.24506602520138e+02

; block 136

poly group "10001" mat 1 con 1 &

face id 1 &

8.02959053430883e+02 7.42957763671875e+02 3.70313836990870e+02 &

7.99831206538623e+02 7.42957763671875e+02 3.64848876953125e+02 &

8.06329060990043e+02 7.42957763671875e+02 3.64848876953125e+02 &

face id 2 &

7.99831206538623e+02 7.42957763671875e+02 3.64848876953125e+02 &

8.02959053430883e+02 7.42957763671875e+02 3.70313836990870e+02 &

8.02814519768868e+02 7.37055118158099e+02 3.64848876953125e+02 &

face id 1 &

8.06329060990043e+02 7.42957763671875e+02 3.64848876953125e+02 &

7.99831206538623e+02 7.42957763671875e+02 3.64848876953125e+02 &

8.02814519768868e+02 7.37055118158099e+02 3.64848876953125e+02 &

face id 2 &

8.02959053430883e+02 7.42957763671875e+02 3.70313836990870e+02 &

8.06329060990043e+02 7.42957763671875e+02 3.64848876953125e+02 &

8.02814519768868e+02 7.37055118158099e+02 3.64848876953125e+02

; block 137

poly group "10001" mat 1 con 1 &

face id 2 &

8.02420357858640e+02 7.31153198242188e+02 3.70297218122081e+02 &

8.02814519768868e+02 7.37055118158099e+02 3.64848876953125e+02 &

7.99300744947642e+02 7.31153198242188e+02 3.64848876953125e+02 &

face id 2 &

8.02814519768868e+02 7.37055118158099e+02 3.64848876953125e+02 &

8.02420357858640e+02 7.31153198242188e+02 3.70297218122081e+02 &

7.99572291217004e+02 7.34946679237868e+02 3.70014757234137e+02 &

face id 2 &

7.99300744947642e+02 7.31153198242188e+02 3.64848876953125e+02 &

8.02814519768868e+02 7.37055118158099e+02 3.64848876953125e+02 &

7.99572291217004e+02 7.34946679237868e+02 3.70014757234137e+02 &

face id 2 &

8.02420357858640e+02 7.31153198242188e+02 3.70297218122081e+02 &

7.99300744947642e+02 7.31153198242188e+02 3.64848876953125e+02 &

7.99572291217004e+02 7.34946679237868e+02 3.70014757234137e+02

; block 138

poly group "10001" mat 1 con 1 &

face id 1 &

7.99300744947642e+02 7.31153198242188e+02 3.64848876953125e+02 &

8.02814519768868e+02 7.37055118158099e+02 3.64848876953125e+02 &

7.96608477764343e+02 7.37040518562079e+02 3.64848876953125e+02 &

face id 2 &

8.02814519768868e+02 7.37055118158099e+02 3.64848876953125e+02 &

7.99300744947642e+02 7.31153198242188e+02 3.64848876953125e+02 &

7.99572291217004e+02 7.34946679237868e+02 3.70014757234137e+02 &

face id 2 &

7.96608477764343e+02 7.37040518562079e+02 3.64848876953125e+02 &

8.02814519768868e+02 7.37055118158099e+02 3.64848876953125e+02 &

7.99572291217004e+02 7.34946679237868e+02 3.70014757234137e+02 &

face id 2 &

7.99300744947642e+02 7.31153198242188e+02 3.64848876953125e+02 &

7.96608477764343e+02 7.37040518562079e+02 3.64848876953125e+02 &

7.99572291217004e+02 7.34946679237868e+02 3.70014757234137e+02

; block 139

poly group "10001" mat 1 con 1 &

face id 1 &

7.90039169839192e+02 7.42957763671875e+02 3.77197613275622e+02 &

7.93786566901830e+02 7.42957763671875e+02 3.81227106778512e+02 &

7.89216666082699e+02 7.42957763671875e+02 3.81321491506794e+02 &

face id 2 &

7.93786566901830e+02 7.42957763671875e+02 3.81227106778512e+02 &

7.90039169839192e+02 7.42957763671875e+02 3.77197613275622e+02 &

7.91306819168008e+02 7.39396483628046e+02 3.82381074655449e+02 &

face id 2 &

7.89216666082699e+02 7.42957763671875e+02 3.81321491506794e+02 &

7.93786566901830e+02 7.42957763671875e+02 3.81227106778512e+02 &

7.91306819168008e+02 7.39396483628046e+02 3.82381074655449e+02 &

face id 2 &

7.90039169839192e+02 7.42957763671875e+02 3.77197613275622e+02 &

7.89216666082699e+02 7.42957763671875e+02 3.81321491506794e+02 &

7.91306819168008e+02 7.39396483628046e+02 3.82381074655449e+02

; block 140

poly group "10001" mat 1 con 1 &

face id 2 &

7.74873482850570e+02 7.34379916376657e+02 3.64848876953125e+02 &

7.80313712190835e+02 7.35222135995747e+02 3.64848876953125e+02 &

7.79101118006749e+02 7.34898937065474e+02 3.69554192649387e+02 &

face id 1 &

7.80313712190835e+02 7.35222135995747e+02 3.64848876953125e+02 &

7.74873482850570e+02 7.34379916376657e+02 3.64848876953125e+02 &

7.78777345184916e+02 7.31153198242188e+02 3.64848876953125e+02 &

face id 2 &

7.79101118006749e+02 7.34898937065474e+02 3.69554192649387e+02 &

7.80313712190835e+02 7.35222135995747e+02 3.64848876953125e+02 &

7.78777345184916e+02 7.31153198242188e+02 3.64848876953125e+02 &

face id 2 &

7.74873482850570e+02 7.34379916376657e+02 3.64848876953125e+02 &

7.79101118006749e+02 7.34898937065474e+02 3.69554192649387e+02 &

7.78777345184916e+02 7.31153198242188e+02 3.64848876953125e+02

; block 141

poly group "10001" mat 1 con 1 &

face id 2 &

8.34882226700407e+02 7.31153198242188e+02 4.49087548912733e+02 &

8.35026428979855e+02 7.33989068884031e+02 4.52089202880859e+02 &

8.34966014154959e+02 7.31153198242188e+02 4.52089202880859e+02 &

face id 2 &

8.35026428979855e+02 7.33989068884031e+02 4.52089202880859e+02 &

8.34882226700407e+02 7.31153198242188e+02 4.49087548912733e+02 &

8.39084289550781e+02 7.31153198242188e+02 4.52089202880859e+02 &

face id 1 &

8.34966014154959e+02 7.31153198242188e+02 4.52089202880859e+02 &

8.35026428979855e+02 7.33989068884031e+02 4.52089202880859e+02 &

8.39084289550781e+02 7.31153198242188e+02 4.52089202880859e+02 &

face id 1 &

8.34882226700407e+02 7.31153198242188e+02 4.49087548912733e+02 &

8.34966014154959e+02 7.31153198242188e+02 4.52089202880859e+02 &

8.39084289550781e+02 7.31153198242188e+02 4.52089202880859e+02

; block 142

poly group "10001" mat 1 con 1 &

face id 2 &

8.34882226700407e+02 7.31153198242188e+02 4.49087548912733e+02 &

8.39084289550781e+02 7.31153198242188e+02 4.47022665071879e+02 &

8.39084289550781e+02 7.35382362355743e+02 4.48634004175127e+02 &

face id 1 &

8.39084289550781e+02 7.31153198242188e+02 4.47022665071879e+02 &

8.34882226700407e+02 7.31153198242188e+02 4.49087548912733e+02 &

8.39084289550781e+02 7.31153198242188e+02 4.52089202880859e+02 &

face id 1 &

8.39084289550781e+02 7.35382362355743e+02 4.48634004175127e+02 &

8.39084289550781e+02 7.31153198242188e+02 4.47022665071879e+02 &

8.39084289550781e+02 7.31153198242188e+02 4.52089202880859e+02 &

face id 2 &

8.34882226700407e+02 7.31153198242188e+02 4.49087548912733e+02 &

8.39084289550781e+02 7.35382362355743e+02 4.48634004175127e+02 &

8.39084289550781e+02 7.31153198242188e+02 4.52089202880859e+02

; block 143

poly group "10001" mat 1 con 1 &

face id 1 &

7.71135925292969e+02 7.37856289064324e+02 3.74220236918397e+02 &

7.72425976066511e+02 7.37573492425862e+02 3.76138787610486e+02 &

7.71135925292969e+02 7.35708841438567e+02 3.75363883785495e+02 &

face id 2 &

7.72425976066511e+02 7.37573492425862e+02 3.76138787610486e+02 &

7.71135925292969e+02 7.37856289064324e+02 3.74220236918397e+02 &

7.73931732739116e+02 7.36394807165182e+02 3.74019289682300e+02 &

face id 2 &

7.71135925292969e+02 7.35708841438567e+02 3.75363883785495e+02 &

7.72425976066511e+02 7.37573492425862e+02 3.76138787610486e+02 &

7.73931732739116e+02 7.36394807165182e+02 3.74019289682300e+02 &

face id 2 &

7.71135925292969e+02 7.37856289064324e+02 3.74220236918397e+02 &

7.71135925292969e+02 7.35708841438567e+02 3.75363883785495e+02 &

7.73931732739116e+02 7.36394807165182e+02 3.74019289682300e+02

; block 144

poly group "10001" mat 1 con 1 &

face id 2 &

8.07730281172094e+02 7.35055793723661e+02 4.10604566802921e+02 &

8.05166630720558e+02 7.39260084743635e+02 4.09062297464921e+02 &

8.06053783905492e+02 7.39353100736547e+02 4.13045444506629e+02 &

face id 2 &

8.05166630720558e+02 7.39260084743635e+02 4.09062297464921e+02 &

8.07730281172094e+02 7.35055793723661e+02 4.10604566802921e+02 &

8.08005581782927e+02 7.38153886431079e+02 4.07831518617536e+02 &

face id 2 &

8.06053783905492e+02 7.39353100736547e+02 4.13045444506629e+02 &

8.05166630720558e+02 7.39260084743635e+02 4.09062297464921e+02 &

8.08005581782927e+02 7.38153886431079e+02 4.07831518617536e+02 &

face id 2 &

8.07730281172094e+02 7.35055793723661e+02 4.10604566802921e+02 &

8.06053783905492e+02 7.39353100736547e+02 4.13045444506629e+02 &

8.08005581782927e+02 7.38153886431079e+02 4.07831518617536e+02

; block 145

poly group "10001" mat 1 con 1 &

face id 2 &

8.09911809905185e+02 7.38789513583694e+02 4.13229940854814e+02 &

8.07730281172094e+02 7.35055793723661e+02 4.10604566802921e+02 &

8.06053783905492e+02 7.39353100736547e+02 4.13045444506629e+02 &

face id 2 &

8.07730281172094e+02 7.35055793723661e+02 4.10604566802921e+02 &

8.09911809905185e+02 7.38789513583694e+02 4.13229940854814e+02 &

8.08005581782927e+02 7.38153886431079e+02 4.07831518617536e+02 &

face id 2 &

8.06053783905492e+02 7.39353100736547e+02 4.13045444506629e+02 &

8.07730281172094e+02 7.35055793723661e+02 4.10604566802921e+02 &

8.08005581782927e+02 7.38153886431079e+02 4.07831518617536e+02 &

face id 2 &

8.09911809905185e+02 7.38789513583694e+02 4.13229940854814e+02 &

8.06053783905492e+02 7.39353100736547e+02 4.13045444506629e+02 &

8.08005581782927e+02 7.38153886431079e+02 4.07831518617536e+02

; block 146

poly group "10001" mat 1 con 1 &

face id 2 &

8.05741411139629e+02 7.38531717048476e+02 4.04360921641735e+02 &

8.08631556812620e+02 7.34644604063582e+02 4.06314744213599e+02 &

8.10063396272645e+02 7.38264255256752e+02 4.03157482752262e+02 &

face id 2 &

8.08631556812620e+02 7.34644604063582e+02 4.06314744213599e+02 &

8.05741411139629e+02 7.38531717048476e+02 4.04360921641735e+02 &

8.08005581782927e+02 7.38153886431079e+02 4.07831518617536e+02 &

face id 2 &

8.10063396272645e+02 7.38264255256752e+02 4.03157482752262e+02 &

8.08631556812620e+02 7.34644604063582e+02 4.06314744213599e+02 &

8.08005581782927e+02 7.38153886431079e+02 4.07831518617536e+02 &

face id 2 &

8.05741411139629e+02 7.38531717048476e+02 4.04360921641735e+02 &

8.10063396272645e+02 7.38264255256752e+02 4.03157482752262e+02 &

8.08005581782927e+02 7.38153886431079e+02 4.07831518617536e+02

; block 147

poly group "10001" mat 1 con 1 &

face id 2 &

7.97168838062648e+02 7.39187632994892e+02 3.86818051061183e+02 &

7.92696333260128e+02 7.42957763671875e+02 3.88796385777957e+02 &

7.95824267105237e+02 7.42957763671875e+02 3.85406053453334e+02 &

face id 2 &

7.92696333260128e+02 7.42957763671875e+02 3.88796385777957e+02 &

7.97168838062648e+02 7.39187632994892e+02 3.86818051061183e+02 &

7.93126102700700e+02 7.39147352988408e+02 3.85920040094875e+02 &

face id 2 &

7.95824267105237e+02 7.42957763671875e+02 3.85406053453334e+02 &

7.92696333260128e+02 7.42957763671875e+02 3.88796385777957e+02 &

7.93126102700700e+02 7.39147352988408e+02 3.85920040094875e+02 &

face id 2 &

7.97168838062648e+02 7.39187632994892e+02 3.86818051061183e+02 &

7.95824267105237e+02 7.42957763671875e+02 3.85406053453334e+02 &

7.93126102700700e+02 7.39147352988408e+02 3.85920040094875e+02

; block 148

poly group "10001" mat 1 con 1 &

face id 1 &

7.90039169839192e+02 7.42957763671875e+02 3.77197613275622e+02 &

7.86150062467024e+02 7.42957763671875e+02 3.78920105584715e+02 &

7.85724375088134e+02 7.42957763671875e+02 3.75809207520845e+02 &

face id 2 &

7.86150062467024e+02 7.42957763671875e+02 3.78920105584715e+02 &

7.90039169839192e+02 7.42957763671875e+02 3.77197613275622e+02 &

7.87873418313903e+02 7.38883924129284e+02 3.75421137459169e+02 &

face id 2 &

7.85724375088134e+02 7.42957763671875e+02 3.75809207520845e+02 &

7.86150062467024e+02 7.42957763671875e+02 3.78920105584715e+02 &

7.87873418313903e+02 7.38883924129284e+02 3.75421137459169e+02 &

face id 2 &

7.90039169839192e+02 7.42957763671875e+02 3.77197613275622e+02 &

7.85724375088134e+02 7.42957763671875e+02 3.75809207520845e+02 &

7.87873418313903e+02 7.38883924129284e+02 3.75421137459169e+02

; block 149

poly group "10001" mat 1 con 1 &

face id 1 &

8.12198316771942e+02 7.36419651568513e+02 4.39675799850027e+02 &

8.13033386230469e+02 7.37296997070312e+02 4.41984466552734e+02 &

8.14714510506809e+02 7.35830011232890e+02 4.41727635030682e+02 &

face id 2 &

8.13033386230469e+02 7.37296997070312e+02 4.41984466552734e+02 &

8.12198316771942e+02 7.36419651568513e+02 4.39675799850027e+02 &

8.14838076915517e+02 7.39071150525117e+02 4.39175800078397e+02 &

face id 2 &

8.14714510506809e+02 7.35830011232890e+02 4.41727635030682e+02 &

8.13033386230469e+02 7.37296997070312e+02 4.41984466552734e+02 &

8.14838076915517e+02 7.39071150525117e+02 4.39175800078397e+02 &

face id 2 &

8.12198316771942e+02 7.36419651568513e+02 4.39675799850027e+02 &

8.14714510506809e+02 7.35830011232890e+02 4.41727635030682e+02 &

8.14838076915517e+02 7.39071150525117e+02 4.39175800078397e+02

; block 150

poly group "10001" mat 1 con 1 &

face id 2 &

7.80799502317009e+02 7.38595032412240e+02 3.78079266885418e+02 &

7.84345668476437e+02 7.40135313357215e+02 3.78526374317212e+02 &

7.83487660963961e+02 7.35663106170653e+02 3.76533043538780e+02 &

face id 2 &

7.84345668476437e+02 7.40135313357215e+02 3.78526374317212e+02 &

7.80799502317009e+02 7.38595032412240e+02 3.78079266885418e+02 &

7.83813396428186e+02 7.39314645759285e+02 3.75273836784356e+02 &

face id 2 &

7.83487660963961e+02 7.35663106170653e+02 3.76533043538780e+02 &

7.84345668476437e+02 7.40135313357215e+02 3.78526374317212e+02 &

7.83813396428186e+02 7.39314645759285e+02 3.75273836784356e+02 &

face id 2 &

7.80799502317009e+02 7.38595032412240e+02 3.78079266885418e+02 &

7.83487660963961e+02 7.35663106170653e+02 3.76533043538780e+02 &

7.83813396428186e+02 7.39314645759285e+02 3.75273836784356e+02

; block 151

poly group "10001" mat 1 con 1 &

face id 1 &

8.04424438044407e+02 7.42957763671875e+02 3.86522059563540e+02 &

7.99724447295145e+02 7.42957763671875e+02 3.87022301448822e+02 &

8.00154378440319e+02 7.42957763671875e+02 3.81345085869093e+02 &

face id 2 &

7.99724447295145e+02 7.42957763671875e+02 3.87022301448822e+02 &

8.04424438044407e+02 7.42957763671875e+02 3.86522059563540e+02 &

8.01551593806463e+02 7.37121430531446e+02 3.83226223248936e+02 &

face id 2 &

8.00154378440319e+02 7.42957763671875e+02 3.81345085869093e+02 &

7.99724447295145e+02 7.42957763671875e+02 3.87022301448822e+02 &

8.01551593806463e+02 7.37121430531446e+02 3.83226223248936e+02 &

face id 2 &

8.04424438044407e+02 7.42957763671875e+02 3.86522059563540e+02 &

8.00154378440319e+02 7.42957763671875e+02 3.81345085869093e+02 &

8.01551593806463e+02 7.37121430531446e+02 3.83226223248936e+02

; block 152

poly group "10001" mat 1 con 1 &

face id 2 &

8.08631556812620e+02 7.34644604063582e+02 4.06314744213599e+02 &

8.12071975737130e+02 7.36741810211853e+02 4.08693605631730e+02 &

8.10063396272645e+02 7.38264255256752e+02 4.03157482752262e+02 &

face id 2 &

8.12071975737130e+02 7.36741810211853e+02 4.08693605631730e+02 &

8.08631556812620e+02 7.34644604063582e+02 4.06314744213599e+02 &

8.08005581782927e+02 7.38153886431079e+02 4.07831518617536e+02 &

face id 2 &

8.10063396272645e+02 7.38264255256752e+02 4.03157482752262e+02 &

8.12071975737130e+02 7.36741810211853e+02 4.08693605631730e+02 &

8.08005581782927e+02 7.38153886431079e+02 4.07831518617536e+02 &

face id 2 &

8.08631556812620e+02 7.34644604063582e+02 4.06314744213599e+02 &

8.10063396272645e+02 7.38264255256752e+02 4.03157482752262e+02 &

8.08005581782927e+02 7.38153886431079e+02 4.07831518617536e+02

; block 153

poly group "10001" mat 1 con 1 &

face id 2 &

8.09796767008882e+02 7.42957763671875e+02 4.10290671888704e+02 &

8.12071975737130e+02 7.36741810211853e+02 4.08693605631730e+02 &

8.09911809905185e+02 7.38789513583694e+02 4.13229940854814e+02 &

face id 2 &

8.12071975737130e+02 7.36741810211853e+02 4.08693605631730e+02 &

8.09796767008882e+02 7.42957763671875e+02 4.10290671888704e+02 &

8.08005581782927e+02 7.38153886431079e+02 4.07831518617536e+02 &

face id 2 &

8.09911809905185e+02 7.38789513583694e+02 4.13229940854814e+02 &

8.12071975737130e+02 7.36741810211853e+02 4.08693605631730e+02 &

8.08005581782927e+02 7.38153886431079e+02 4.07831518617536e+02 &

face id 2 &

8.09796767008882e+02 7.42957763671875e+02 4.10290671888704e+02 &

8.09911809905185e+02 7.38789513583694e+02 4.13229940854814e+02 &

8.08005581782927e+02 7.38153886431079e+02 4.07831518617536e+02

; block 154

poly group "10001" mat 1 con 1 &

face id 2 &

8.12071975737130e+02 7.36741810211853e+02 4.08693605631730e+02 &

8.07730281172094e+02 7.35055793723661e+02 4.10604566802921e+02 &

8.09911809905185e+02 7.38789513583694e+02 4.13229940854814e+02 &

face id 2 &

8.07730281172094e+02 7.35055793723661e+02 4.10604566802921e+02 &

8.12071975737130e+02 7.36741810211853e+02 4.08693605631730e+02 &

8.08005581782927e+02 7.38153886431079e+02 4.07831518617536e+02 &

face id 2 &

8.09911809905185e+02 7.38789513583694e+02 4.13229940854814e+02 &

8.07730281172094e+02 7.35055793723661e+02 4.10604566802921e+02 &

8.08005581782927e+02 7.38153886431079e+02 4.07831518617536e+02 &

face id 2 &

8.12071975737130e+02 7.36741810211853e+02 4.08693605631730e+02 &

8.09911809905185e+02 7.38789513583694e+02 4.13229940854814e+02 &

8.08005581782927e+02 7.38153886431079e+02 4.07831518617536e+02

; block 155

poly group "10001" mat 1 con 1 &

face id 2 &

8.07730281172094e+02 7.35055793723661e+02 4.10604566802921e+02 &

8.12071975737130e+02 7.36741810211853e+02 4.08693605631730e+02 &

8.08631556812620e+02 7.34644604063582e+02 4.06314744213599e+02 &

face id 2 &

8.12071975737130e+02 7.36741810211853e+02 4.08693605631730e+02 &

8.07730281172094e+02 7.35055793723661e+02 4.10604566802921e+02 &

8.08005581782927e+02 7.38153886431079e+02 4.07831518617536e+02 &

face id 2 &

8.08631556812620e+02 7.34644604063582e+02 4.06314744213599e+02 &

8.12071975737130e+02 7.36741810211853e+02 4.08693605631730e+02 &

8.08005581782927e+02 7.38153886431079e+02 4.07831518617536e+02 &

face id 2 &

8.07730281172094e+02 7.35055793723661e+02 4.10604566802921e+02 &

8.08631556812620e+02 7.34644604063582e+02 4.06314744213599e+02 &

8.08005581782927e+02 7.38153886431079e+02 4.07831518617536e+02

; block 156

poly group "10001" mat 1 con 1 &

face id 2 &

7.77051870553251e+02 7.37085809011748e+02 3.76695713437529e+02 &

7.73931732739116e+02 7.36394807165182e+02 3.74019289682300e+02 &

7.76938768959141e+02 7.39984334820939e+02 3.75213120298485e+02 &

face id 2 &

7.73931732739116e+02 7.36394807165182e+02 3.74019289682300e+02 &

7.77051870553251e+02 7.37085809011748e+02 3.76695713437529e+02 &

7.77182913303155e+02 7.38045780756561e+02 3.71850481726892e+02 &

face id 2 &

7.76938768959141e+02 7.39984334820939e+02 3.75213120298485e+02 &

7.73931732739116e+02 7.36394807165182e+02 3.74019289682300e+02 &

7.77182913303155e+02 7.38045780756561e+02 3.71850481726892e+02 &

face id 2 &

7.77051870553251e+02 7.37085809011748e+02 3.76695713437529e+02 &

7.76938768959141e+02 7.39984334820939e+02 3.75213120298485e+02 &

7.77182913303155e+02 7.38045780756561e+02 3.71850481726892e+02

; block 157

poly group "10001" mat 1 con 1 &

face id 2 &

7.80279121453295e+02 7.34974032328978e+02 3.74087850984229e+02 &

7.77182913303155e+02 7.38045780756561e+02 3.71850481726892e+02 &

7.79101118006749e+02 7.34898937065474e+02 3.69554192649387e+02 &

face id 2 &

7.77182913303155e+02 7.38045780756561e+02 3.71850481726892e+02 &

7.80279121453295e+02 7.34974032328978e+02 3.74087850984229e+02 &

7.76873874052688e+02 7.34965282604117e+02 3.73088491535245e+02 &

face id 2 &

7.79101118006749e+02 7.34898937065474e+02 3.69554192649387e+02 &

7.77182913303155e+02 7.38045780756561e+02 3.71850481726892e+02 &

7.76873874052688e+02 7.34965282604117e+02 3.73088491535245e+02 &

face id 2 &

7.80279121453295e+02 7.34974032328978e+02 3.74087850984229e+02 &

7.79101118006749e+02 7.34898937065474e+02 3.69554192649387e+02 &

7.76873874052688e+02 7.34965282604117e+02 3.73088491535245e+02

; block 158

poly group "10001" mat 1 con 1 &

face id 1 &

7.81100846896327e+02 7.31153198242188e+02 3.81735958194611e+02 &

7.81919545568227e+02 7.31153198242188e+02 3.77708803974256e+02 &

7.78389795522681e+02 7.31153198242188e+02 3.79403540956464e+02 &

face id 2 &

7.81919545568227e+02 7.31153198242188e+02 3.77708803974256e+02 &

7.81100846896327e+02 7.31153198242188e+02 3.81735958194611e+02 &

7.79511950549799e+02 7.34457282336568e+02 3.78709527306644e+02 &

face id 2 &

7.78389795522681e+02 7.31153198242188e+02 3.79403540956464e+02 &

7.81919545568227e+02 7.31153198242188e+02 3.77708803974256e+02 &

7.79511950549799e+02 7.34457282336568e+02 3.78709527306644e+02 &

face id 2 &

7.81100846896327e+02 7.31153198242188e+02 3.81735958194611e+02 &

7.78389795522681e+02 7.31153198242188e+02 3.79403540956464e+02 &

7.79511950549799e+02 7.34457282336568e+02 3.78709527306644e+02

; block 159

poly group "10001" mat 1 con 1 &

face id 2 &

7.76629831384941e+02 7.34030537730047e+02 3.76463041133043e+02 &

7.77051870553251e+02 7.37085809011748e+02 3.76695713437529e+02 &

7.80279121453295e+02 7.34974032328978e+02 3.74087850984229e+02 &

face id 2 &

7.77051870553251e+02 7.37085809011748e+02 3.76695713437529e+02 &

7.76629831384941e+02 7.34030537730047e+02 3.76463041133043e+02 &

7.76873874052688e+02 7.34965282604117e+02 3.73088491535245e+02 &

face id 2 &

7.80279121453295e+02 7.34974032328978e+02 3.74087850984229e+02 &

7.77051870553251e+02 7.37085809011748e+02 3.76695713437529e+02 &

7.76873874052688e+02 7.34965282604117e+02 3.73088491535245e+02 &

face id 2 &

7.76629831384941e+02 7.34030537730047e+02 3.76463041133043e+02 &

7.80279121453295e+02 7.34974032328978e+02 3.74087850984229e+02 &

7.76873874052688e+02 7.34965282604117e+02 3.73088491535245e+02

; block 160

poly group "10001" mat 1 con 1 &

face id 2 &

7.77051870553251e+02 7.37085809011748e+02 3.76695713437529e+02 &

7.77182913303155e+02 7.38045780756561e+02 3.71850481726892e+02 &

7.80279121453295e+02 7.34974032328978e+02 3.74087850984229e+02 &

face id 2 &

7.77182913303155e+02 7.38045780756561e+02 3.71850481726892e+02 &

7.77051870553251e+02 7.37085809011748e+02 3.76695713437529e+02 &

7.76873874052688e+02 7.34965282604117e+02 3.73088491535245e+02 &

face id 2 &

7.80279121453295e+02 7.34974032328978e+02 3.74087850984229e+02 &

7.77182913303155e+02 7.38045780756561e+02 3.71850481726892e+02 &

7.76873874052688e+02 7.34965282604117e+02 3.73088491535245e+02 &

face id 2 &

7.77051870553251e+02 7.37085809011748e+02 3.76695713437529e+02 &

7.80279121453295e+02 7.34974032328978e+02 3.74087850984229e+02 &

7.76873874052688e+02 7.34965282604117e+02 3.73088491535245e+02

; block 161

poly group "10001" mat 1 con 1 &

face id 2 &

7.73853484607426e+02 7.38926977297481e+02 3.64848876953125e+02 &

7.74603396653911e+02 7.37387876588618e+02 3.68598092656050e+02 &

7.77400463245723e+02 7.38786178236515e+02 3.64848876953125e+02 &

face id 2 &

7.74603396653911e+02 7.37387876588618e+02 3.68598092656050e+02 &

7.73853484607426e+02 7.38926977297481e+02 3.64848876953125e+02 &

7.74035965173978e+02 7.40428059095133e+02 3.67725881874126e+02 &

face id 2 &

7.77400463245723e+02 7.38786178236515e+02 3.64848876953125e+02 &

7.74603396653911e+02 7.37387876588618e+02 3.68598092656050e+02 &

7.74035965173978e+02 7.40428059095133e+02 3.67725881874126e+02 &

face id 2 &

7.73853484607426e+02 7.38926977297481e+02 3.64848876953125e+02 &

7.77400463245723e+02 7.38786178236515e+02 3.64848876953125e+02 &

7.74035965173978e+02 7.40428059095133e+02 3.67725881874126e+02

; block 162

poly group "10001" mat 1 con 1 &

face id 2 &

7.76910067951921e+02 7.42957763671875e+02 3.68343163376403e+02 &

7.77400463245723e+02 7.38786178236515e+02 3.64848876953125e+02 &

7.74899575681289e+02 7.42957763671875e+02 3.64848876953125e+02 &

face id 2 &

7.77400463245723e+02 7.38786178236515e+02 3.64848876953125e+02 &

7.76910067951921e+02 7.42957763671875e+02 3.68343163376403e+02 &

7.79021842216292e+02 7.42957763671875e+02 3.64848876953125e+02 &

face id 1 &

7.74899575681289e+02 7.42957763671875e+02 3.64848876953125e+02 &

7.77400463245723e+02 7.38786178236515e+02 3.64848876953125e+02 &

7.79021842216292e+02 7.42957763671875e+02 3.64848876953125e+02 &

face id 1 &

7.76910067951921e+02 7.42957763671875e+02 3.68343163376403e+02 &

7.74899575681289e+02 7.42957763671875e+02 3.64848876953125e+02 &

7.79021842216292e+02 7.42957763671875e+02 3.64848876953125e+02

; block 163

poly group "10001" mat 1 con 1 &

face id 1 &

7.76268518704517e+02 7.42957763671875e+02 3.72447920290831e+02 &

7.76910067951921e+02 7.42957763671875e+02 3.68343163376403e+02 &

7.79423947278674e+02 7.42957763671875e+02 3.71496632848602e+02 &

face id 2 &

7.76910067951921e+02 7.42957763671875e+02 3.68343163376403e+02 &

7.76268518704517e+02 7.42957763671875e+02 3.72447920290831e+02 &

7.79020877778572e+02 7.40242502036992e+02 3.68708253145675e+02 &

face id 2 &

7.79423947278674e+02 7.42957763671875e+02 3.71496632848602e+02 &

7.76910067951921e+02 7.42957763671875e+02 3.68343163376403e+02 &

7.79020877778572e+02 7.40242502036992e+02 3.68708253145675e+02 &

face id 2 &

7.76268518704517e+02 7.42957763671875e+02 3.72447920290831e+02 &

7.79423947278674e+02 7.42957763671875e+02 3.71496632848602e+02 &

7.79020877778572e+02 7.40242502036992e+02 3.68708253145675e+02

; block 164

poly group "10001" mat 1 con 1 &

face id 2 &

8.04234314501538e+02 7.34635014532937e+02 4.05451928779116e+02 &

8.08631556812620e+02 7.34644604063582e+02 4.06314744213599e+02 &

8.05741411139629e+02 7.38531717048476e+02 4.04360921641735e+02 &

face id 2 &

8.08631556812620e+02 7.34644604063582e+02 4.06314744213599e+02 &

8.04234314501538e+02 7.34635014532937e+02 4.05451928779116e+02 &

8.08005581782927e+02 7.38153886431079e+02 4.07831518617536e+02 &

face id 2 &

8.05741411139629e+02 7.38531717048476e+02 4.04360921641735e+02 &

8.08631556812620e+02 7.34644604063582e+02 4.06314744213599e+02 &

8.08005581782927e+02 7.38153886431079e+02 4.07831518617536e+02 &

face id 2 &

8.04234314501538e+02 7.34635014532937e+02 4.05451928779116e+02 &

8.05741411139629e+02 7.38531717048476e+02 4.04360921641735e+02 &

8.08005581782927e+02 7.38153886431079e+02 4.07831518617536e+02

; block 165

poly group "10001" mat 1 con 1 &

face id 2 &

7.73931732739116e+02 7.36394807165182e+02 3.74019289682300e+02 &

7.77051870553251e+02 7.37085809011748e+02 3.76695713437529e+02 &

7.76629831384941e+02 7.34030537730047e+02 3.76463041133043e+02 &

face id 2 &

7.77051870553251e+02 7.37085809011748e+02 3.76695713437529e+02 &

7.73931732739116e+02 7.36394807165182e+02 3.74019289682300e+02 &

7.76873874052688e+02 7.34965282604117e+02 3.73088491535245e+02 &

face id 2 &

7.76629831384941e+02 7.34030537730047e+02 3.76463041133043e+02 &

7.77051870553251e+02 7.37085809011748e+02 3.76695713437529e+02 &

7.76873874052688e+02 7.34965282604117e+02 3.73088491535245e+02 &

face id 2 &

7.73931732739116e+02 7.36394807165182e+02 3.74019289682300e+02 &

7.76629831384941e+02 7.34030537730047e+02 3.76463041133043e+02 &

7.76873874052688e+02 7.34965282604117e+02 3.73088491535245e+02

; block 166

poly group "10001" mat 1 con 1 &

face id 2 &

8.26114508878027e+02 7.34878405664679e+02 4.40774789501596e+02 &

8.29251774542374e+02 7.34873958371039e+02 4.43856971841759e+02 &

8.30150338633184e+02 7.35459866734055e+02 4.38544963342445e+02 &

face id 2 &

8.29251774542374e+02 7.34873958371039e+02 4.43856971841759e+02 &

8.26114508878027e+02 7.34878405664679e+02 4.40774789501596e+02 &

8.28516744472485e+02 7.39206897844698e+02 4.40026719289599e+02 &

face id 2 &

8.30150338633184e+02 7.35459866734055e+02 4.38544963342445e+02 &

8.29251774542374e+02 7.34873958371039e+02 4.43856971841759e+02 &

8.28516744472485e+02 7.39206897844698e+02 4.40026719289599e+02 &

face id 2 &

8.26114508878027e+02 7.34878405664679e+02 4.40774789501596e+02 &

8.30150338633184e+02 7.35459866734055e+02 4.38544963342445e+02 &

8.28516744472485e+02 7.39206897844698e+02 4.40026719289599e+02

; block 167

poly group "10001" mat 1 con 1 &

face id 1 &

8.26215102552087e+02 7.42957763671875e+02 4.40024896299389e+02 &

8.30336570924782e+02 7.42957763671875e+02 4.37949645698195e+02 &

8.30279431984152e+02 7.42957763671875e+02 4.42330783680423e+02 &

face id 2 &

8.30336570924782e+02 7.42957763671875e+02 4.37949645698195e+02 &

8.26215102552087e+02 7.42957763671875e+02 4.40024896299389e+02 &

8.28516744472485e+02 7.39206897844698e+02 4.40026719289599e+02 &

face id 2 &

8.30279431984152e+02 7.42957763671875e+02 4.42330783680423e+02 &

8.30336570924782e+02 7.42957763671875e+02 4.37949645698195e+02 &

8.28516744472485e+02 7.39206897844698e+02 4.40026719289599e+02 &

face id 2 &

8.26215102552087e+02 7.42957763671875e+02 4.40024896299389e+02 &

8.30279431984152e+02 7.42957763671875e+02 4.42330783680423e+02 &

8.28516744472485e+02 7.39206897844698e+02 4.40026719289599e+02

; block 168

poly group "10001" mat 1 con 1 &

face id 2 &

8.05741411139629e+02 7.38531717048476e+02 4.04360921641735e+02 &

8.03128066302503e+02 7.38633199556869e+02 4.01150420510148e+02 &

8.00896162902359e+02 7.34934397043815e+02 4.04514414125901e+02 &

face id 2 &

8.03128066302503e+02 7.38633199556869e+02 4.01150420510148e+02 &

8.05741411139629e+02 7.38531717048476e+02 4.04360921641735e+02 &

8.01835956103130e+02 7.39112516643646e+02 4.06005204500498e+02 &

face id 2 &

8.00896162902359e+02 7.34934397043815e+02 4.04514414125901e+02 &

8.03128066302503e+02 7.38633199556869e+02 4.01150420510148e+02 &

8.01835956103130e+02 7.39112516643646e+02 4.06005204500498e+02 &

face id 2 &

8.05741411139629e+02 7.38531717048476e+02 4.04360921641735e+02 &

8.00896162902359e+02 7.34934397043815e+02 4.04514414125901e+02 &

8.01835956103130e+02 7.39112516643646e+02 4.06005204500498e+02

; block 169

poly group "10001" mat 1 con 1 &

face id 2 &

8.15793233645232e+02 7.34747728264843e+02 4.35883786023879e+02 &

8.17450431002428e+02 7.33768641315643e+02 4.33222124331104e+02 &

8.14181028467167e+02 7.32132772428309e+02 4.34887560622655e+02 &

face id 2 &

8.17450431002428e+02 7.33768641315643e+02 4.33222124331104e+02 &

8.15793233645232e+02 7.34747728264843e+02 4.35883786023879e+02 &

8.13616534804187e+02 7.34858466472432e+02 4.33854840236454e+02 &

face id 2 &

8.14181028467167e+02 7.32132772428309e+02 4.34887560622655e+02 &

8.17450431002428e+02 7.33768641315643e+02 4.33222124331104e+02 &

8.13616534804187e+02 7.34858466472432e+02 4.33854840236454e+02 &

face id 2 &

8.15793233645232e+02 7.34747728264843e+02 4.35883786023879e+02 &

8.14181028467167e+02 7.32132772428309e+02 4.34887560622655e+02 &

8.13616534804187e+02 7.34858466472432e+02 4.33854840236454e+02

; block 170

poly group "10001" mat 1 con 1 &

face id 2 &

8.10038104121493e+02 7.39022257823943e+02 4.35900094865737e+02 &

8.12285854665448e+02 7.37661675314558e+02 4.34186351604388e+02 &

8.10253018028565e+02 7.40840161728360e+02 4.35461772473196e+02 &

face id 2 &

8.12285854665448e+02 7.37661675314558e+02 4.34186351604388e+02 &

8.10038104121493e+02 7.39022257823943e+02 4.35900094865737e+02 &

8.13221790344536e+02 7.40692716560294e+02 4.36922508985554e+02 &

face id 2 &

8.10253018028565e+02 7.40840161728360e+02 4.35461772473196e+02 &

8.12285854665448e+02 7.37661675314558e+02 4.34186351604388e+02 &

8.13221790344536e+02 7.40692716560294e+02 4.36922508985554e+02 &

face id 2 &

8.10038104121493e+02 7.39022257823943e+02 4.35900094865737e+02 &

8.10253018028565e+02 7.40840161728360e+02 4.35461772473196e+02 &

8.13221790344536e+02 7.40692716560294e+02 4.36922508985554e+02

; block 171

poly group "10001" mat 1 con 1 &

face id 1 &

8.19791625976562e+02 7.37224121093750e+02 4.44772796630859e+02 &

8.21872915596893e+02 7.35455555334649e+02 4.44418293051948e+02 &

8.19953904426496e+02 7.34738927486142e+02 4.42268893279197e+02 &

face id 2 &

8.21872915596893e+02 7.35455555334649e+02 4.44418293051948e+02 &

8.19791625976562e+02 7.37224121093750e+02 4.44772796630859e+02 &

8.19470217536442e+02 7.38262925844703e+02 4.41377617023149e+02 &

face id 2 &

8.19953904426496e+02 7.34738927486142e+02 4.42268893279197e+02 &

8.21872915596893e+02 7.35455555334649e+02 4.44418293051948e+02 &

8.19470217536442e+02 7.38262925844703e+02 4.41377617023149e+02 &

face id 2 &

8.19791625976562e+02 7.37224121093750e+02 4.44772796630859e+02 &

8.19953904426496e+02 7.34738927486142e+02 4.42268893279197e+02 &

8.19470217536442e+02 7.38262925844703e+02 4.41377617023149e+02

; block 172

poly group "10001" mat 1 con 1 &

face id 1 &

8.21351515263609e+02 7.31979012400083e+02 4.38941281795801e+02 &

8.23611633300781e+02 7.31153198242188e+02 4.38513122558594e+02 &

8.21044537549873e+02 7.31153198242188e+02 4.36848956154312e+02 &

face id 2 &

8.23611633300781e+02 7.31153198242188e+02 4.38513122558594e+02 &

8.21351515263609e+02 7.31979012400083e+02 4.38941281795801e+02 &

8.23363932724725e+02 7.33802420140527e+02 4.37276399935772e+02 &

face id 2 &

8.21044537549873e+02 7.31153198242188e+02 4.36848956154312e+02 &

8.23611633300781e+02 7.31153198242188e+02 4.38513122558594e+02 &

8.23363932724725e+02 7.33802420140527e+02 4.37276399935772e+02 &

face id 2 &

8.21351515263609e+02 7.31979012400083e+02 4.38941281795801e+02 &

8.21044537549873e+02 7.31153198242188e+02 4.36848956154312e+02 &

8.23363932724725e+02 7.33802420140527e+02 4.37276399935772e+02

; block 173

poly group "10001" mat 1 con 1 &

face id 1 &

8.20009998744681e+02 7.42957763671875e+02 4.32354041528251e+02 &

8.16320558032978e+02 7.42957763671875e+02 4.31298908485349e+02 &

8.19213703414815e+02 7.42957763671875e+02 4.27495334690443e+02 &

face id 2 &

8.16320558032978e+02 7.42957763671875e+02 4.31298908485349e+02 &

8.20009998744681e+02 7.42957763671875e+02 4.32354041528251e+02 &

8.18073172819596e+02 7.39472123297221e+02 4.29992682558518e+02 &

face id 2 &

8.19213703414815e+02 7.42957763671875e+02 4.27495334690443e+02 &

8.16320558032978e+02 7.42957763671875e+02 4.31298908485349e+02 &

8.18073172819596e+02 7.39472123297221e+02 4.29992682558518e+02 &

face id 2 &

8.20009998744681e+02 7.42957763671875e+02 4.32354041528251e+02 &

8.19213703414815e+02 7.42957763671875e+02 4.27495334690443e+02 &

8.18073172819596e+02 7.39472123297221e+02 4.29992682558518e+02

; block 174

poly group "10001" mat 1 con 1 &

face id 2 &

7.87643654364816e+02 7.34661112155498e+02 3.86945147752232e+02 &

7.91723693011833e+02 7.34565359260124e+02 3.87421651270315e+02 &

7.89863937233331e+02 7.38539747715945e+02 3.87566254647709e+02 &

face id 2 &

7.91723693011833e+02 7.34565359260124e+02 3.87421651270315e+02 &

7.87643654364816e+02 7.34661112155498e+02 3.86945147752232e+02 &

7.89197276621344e+02 7.34576411507420e+02 3.90327463427344e+02 &

face id 2 &

7.89863937233331e+02 7.38539747715945e+02 3.87566254647709e+02 &

7.91723693011833e+02 7.34565359260124e+02 3.87421651270315e+02 &

7.89197276621344e+02 7.34576411507420e+02 3.90327463427344e+02 &

face id 2 &

7.87643654364816e+02 7.34661112155498e+02 3.86945147752232e+02 &

7.89863937233331e+02 7.38539747715945e+02 3.87566254647709e+02 &

7.89197276621344e+02 7.34576411507420e+02 3.90327463427344e+02

; block 175

poly group "10001" mat 1 con 1 &

face id 1 &

8.09301722486162e+02 7.31153198242187e+02 4.08675358829636e+02 &

8.05732608301790e+02 7.31153198242188e+02 4.10478336468474e+02 &

8.08925078135902e+02 7.31153198242188e+02 4.12433565810271e+02 &

face id 2 &

8.05732608301790e+02 7.31153198242188e+02 4.10478336468474e+02 &

8.09301722486162e+02 7.31153198242187e+02 4.08675358829636e+02 &

8.07730281172094e+02 7.35055793723661e+02 4.10604566802921e+02 &

face id 2 &

8.08925078135902e+02 7.31153198242188e+02 4.12433565810271e+02 &

8.05732608301790e+02 7.31153198242188e+02 4.10478336468474e+02 &

8.07730281172094e+02 7.35055793723661e+02 4.10604566802921e+02 &

face id 2 &

8.09301722486162e+02 7.31153198242187e+02 4.08675358829636e+02 &

8.08925078135902e+02 7.31153198242188e+02 4.12433565810271e+02 &

8.07730281172094e+02 7.35055793723661e+02 4.10604566802921e+02

; block 176

poly group "10001" mat 1 con 1 &

face id 2 &

8.05732608301790e+02 7.31153198242188e+02 4.10478336468474e+02 &

8.07206434877838e+02 7.34828533348313e+02 4.13909643827570e+02 &

8.08925078135902e+02 7.31153198242188e+02 4.12433565810271e+02 &

face id 2 &

8.07206434877838e+02 7.34828533348313e+02 4.13909643827570e+02 &

8.05732608301790e+02 7.31153198242188e+02 4.10478336468474e+02 &

8.07730281172094e+02 7.35055793723661e+02 4.10604566802921e+02 &

face id 2 &

8.08925078135902e+02 7.31153198242188e+02 4.12433565810271e+02 &

8.07206434877838e+02 7.34828533348313e+02 4.13909643827570e+02 &

8.07730281172094e+02 7.35055793723661e+02 4.10604566802921e+02 &

face id 2 &

8.05732608301790e+02 7.31153198242188e+02 4.10478336468474e+02 &

8.08925078135902e+02 7.31153198242188e+02 4.12433565810271e+02 &

8.07730281172094e+02 7.35055793723661e+02 4.10604566802921e+02

; block 177

poly group "10001" mat 1 con 1 &

face id 2 &

8.09301722486162e+02 7.31153198242187e+02 4.08675358829636e+02 &

8.08631556812620e+02 7.34644604063582e+02 4.06314744213599e+02 &

8.05732608301790e+02 7.31153198242188e+02 4.10478336468474e+02 &

face id 2 &

8.08631556812620e+02 7.34644604063582e+02 4.06314744213599e+02 &

8.09301722486162e+02 7.31153198242187e+02 4.08675358829636e+02 &

8.07730281172094e+02 7.35055793723661e+02 4.10604566802921e+02 &

face id 2 &

8.05732608301790e+02 7.31153198242188e+02 4.10478336468474e+02 &

8.08631556812620e+02 7.34644604063582e+02 4.06314744213599e+02 &

8.07730281172094e+02 7.35055793723661e+02 4.10604566802921e+02 &

face id 2 &

8.09301722486162e+02 7.31153198242187e+02 4.08675358829636e+02 &

8.05732608301790e+02 7.31153198242188e+02 4.10478336468474e+02 &

8.07730281172094e+02 7.35055793723661e+02 4.10604566802921e+02

; block 178

poly group "10001" mat 1 con 1 &

face id 2 &

8.14194519614525e+02 7.36222240308663e+02 4.03223835774641e+02 &

8.12071975737130e+02 7.36741810211853e+02 4.08693605631730e+02 &

8.13668525515790e+02 7.31153198242188e+02 4.06737925704045e+02 &

face id 2 &

8.12071975737130e+02 7.36741810211853e+02 4.08693605631730e+02 &

8.14194519614525e+02 7.36222240308663e+02 4.03223835774641e+02 &

8.18044212102941e+02 7.37294444794664e+02 4.06947605911675e+02 &

face id 2 &

8.13668525515790e+02 7.31153198242188e+02 4.06737925704045e+02 &

8.12071975737130e+02 7.36741810211853e+02 4.08693605631730e+02 &

8.18044212102941e+02 7.37294444794664e+02 4.06947605911675e+02 &

face id 2 &

8.14194519614525e+02 7.36222240308663e+02 4.03223835774641e+02 &

8.13668525515790e+02 7.31153198242188e+02 4.06737925704045e+02 &

8.18044212102941e+02 7.37294444794664e+02 4.06947605911675e+02

; block 179

poly group "10001" mat 1 con 1 &

face id 2 &

7.77182913303155e+02 7.38045780756561e+02 3.71850481726892e+02 &

7.77051870553251e+02 7.37085809011748e+02 3.76695713437529e+02 &

7.73931732739116e+02 7.36394807165182e+02 3.74019289682300e+02 &

face id 2 &

7.77051870553251e+02 7.37085809011748e+02 3.76695713437529e+02 &

7.77182913303155e+02 7.38045780756561e+02 3.71850481726892e+02 &

7.76873874052688e+02 7.34965282604117e+02 3.73088491535245e+02 &

face id 2 &

7.73931732739116e+02 7.36394807165182e+02 3.74019289682300e+02 &

7.77051870553251e+02 7.37085809011748e+02 3.76695713437529e+02 &

7.76873874052688e+02 7.34965282604117e+02 3.73088491535245e+02 &

face id 2 &

7.77182913303155e+02 7.38045780756561e+02 3.71850481726892e+02 &

7.73931732739116e+02 7.36394807165182e+02 3.74019289682300e+02 &

7.76873874052688e+02 7.34965282604117e+02 3.73088491535245e+02

; block 180

poly group "10001" mat 1 con 1 &

face id 2 &

7.79220406464566e+02 7.42957763671875e+02 3.75205861997925e+02 &

7.78505842137311e+02 7.40529325582286e+02 3.79457485086928e+02 &

7.80165506077248e+02 7.42957763671875e+02 3.79040446190487e+02 &

face id 2 &

7.78505842137311e+02 7.40529325582286e+02 3.79457485086928e+02 &

7.79220406464566e+02 7.42957763671875e+02 3.75205861997925e+02 &

7.77530581111218e+02 7.42957763671875e+02 3.77862977864846e+02 &

face id 1 &

7.80165506077248e+02 7.42957763671875e+02 3.79040446190487e+02 &

7.78505842137311e+02 7.40529325582286e+02 3.79457485086928e+02 &

7.77530581111218e+02 7.42957763671875e+02 3.77862977864846e+02 &

face id 1 &

7.79220406464566e+02 7.42957763671875e+02 3.75205861997925e+02 &

7.80165506077248e+02 7.42957763671875e+02 3.79040446190487e+02 &

7.77530581111218e+02 7.42957763671875e+02 3.77862977864846e+02

; block 181

poly group "10001" mat 1 con 1 &

face id 2 &

7.83487660963961e+02 7.35663106170653e+02 3.76533043538780e+02 &

7.80785664505661e+02 7.39236281877246e+02 3.73124042480188e+02 &

7.80799502317009e+02 7.38595032412240e+02 3.78079266885418e+02 &

face id 2 &

7.80785664505661e+02 7.39236281877246e+02 3.73124042480188e+02 &

7.83487660963961e+02 7.35663106170653e+02 3.76533043538780e+02 &

7.83813396428186e+02 7.39314645759285e+02 3.75273836784356e+02 &

face id 2 &

7.80799502317009e+02 7.38595032412240e+02 3.78079266885418e+02 &

7.80785664505661e+02 7.39236281877246e+02 3.73124042480188e+02 &

7.83813396428186e+02 7.39314645759285e+02 3.75273836784356e+02 &

face id 2 &

7.83487660963961e+02 7.35663106170653e+02 3.76533043538780e+02 &

7.80799502317009e+02 7.38595032412240e+02 3.78079266885418e+02 &

7.83813396428186e+02 7.39314645759285e+02 3.75273836784356e+02

; block 182

poly group "10001" mat 1 con 1 &

face id 2 &

8.19270705387996e+02 7.33695343369261e+02 4.35937073693200e+02 &

8.18363373352647e+02 7.37289189498031e+02 4.37173452288585e+02 &

8.20686199095838e+02 7.34536120483993e+02 4.38575068556780e+02 &

face id 2 &

8.18363373352647e+02 7.37289189498031e+02 4.37173452288585e+02 &

8.19270705387996e+02 7.33695343369261e+02 4.35937073693200e+02 &

8.21832695989208e+02 7.37054930723214e+02 4.37100055976185e+02 &

face id 2 &

8.20686199095838e+02 7.34536120483993e+02 4.38575068556780e+02 &

8.18363373352647e+02 7.37289189498031e+02 4.37173452288585e+02 &

8.21832695989208e+02 7.37054930723214e+02 4.37100055976185e+02 &

face id 2 &

8.19270705387996e+02 7.33695343369261e+02 4.35937073693200e+02 &

8.20686199095838e+02 7.34536120483993e+02 4.38575068556780e+02 &

8.21832695989208e+02 7.37054930723214e+02 4.37100055976185e+02

; block 183

poly group "10001" mat 1 con 1 &

face id 2 &

7.73620174311311e+02 7.31153198242188e+02 3.75562823635389e+02 &

7.76629831384941e+02 7.34030537730047e+02 3.76463041133043e+02 &

7.76277723244486e+02 7.31153198242188e+02 3.74603674908883e+02 &

face id 2 &

7.76629831384941e+02 7.34030537730047e+02 3.76463041133043e+02 &

7.73620174311311e+02 7.31153198242188e+02 3.75562823635389e+02 &

7.74716057886906e+02 7.34040302468977e+02 3.74164033232456e+02 &

face id 2 &

7.76277723244486e+02 7.31153198242188e+02 3.74603674908883e+02 &

7.76629831384941e+02 7.34030537730047e+02 3.76463041133043e+02 &

7.74716057886906e+02 7.34040302468977e+02 3.74164033232456e+02 &

face id 2 &

7.73620174311311e+02 7.31153198242188e+02 3.75562823635389e+02 &

7.76277723244486e+02 7.31153198242188e+02 3.74603674908883e+02 &

7.74716057886906e+02 7.34040302468977e+02 3.74164033232456e+02

; block 184

poly group "10001" mat 1 con 1 &

face id 2 &

8.18859811694220e+02 7.36907823701414e+02 4.33778095113987e+02 &

8.18363373352647e+02 7.37289189498031e+02 4.37173452288585e+02 &

8.19270705387996e+02 7.33695343369261e+02 4.35937073693200e+02 &

face id 2 &

8.18363373352647e+02 7.37289189498031e+02 4.37173452288585e+02 &

8.18859811694220e+02 7.36907823701414e+02 4.33778095113987e+02 &

8.21832695989208e+02 7.37054930723214e+02 4.37100055976185e+02 &

face id 2 &

8.19270705387996e+02 7.33695343369261e+02 4.35937073693200e+02 &

8.18363373352647e+02 7.37289189498031e+02 4.37173452288585e+02 &

8.21832695989208e+02 7.37054930723214e+02 4.37100055976185e+02 &

face id 2 &

8.18859811694220e+02 7.36907823701414e+02 4.33778095113987e+02 &

8.19270705387996e+02 7.33695343369261e+02 4.35937073693200e+02 &

8.21832695989208e+02 7.37054930723214e+02 4.37100055976185e+02

; block 185

poly group "10001" mat 1 con 1 &

face id 1 &

7.75612143490425e+02 7.36202827550051e+02 3.79657937703356e+02 &

7.74307374524590e+02 7.38276177052764e+02 3.77641478314468e+02 &

7.76623604261048e+02 7.38637497875149e+02 3.79313058357629e+02 &

face id 2 &

7.74307374524590e+02 7.38276177052764e+02 3.77641478314468e+02 &

7.75612143490425e+02 7.36202827550051e+02 3.79657937703356e+02 &

7.77051870553251e+02 7.37085809011748e+02 3.76695713437529e+02 &

face id 2 &

7.76623604261048e+02 7.38637497875149e+02 3.79313058357629e+02 &

7.74307374524590e+02 7.38276177052764e+02 3.77641478314468e+02 &

7.77051870553251e+02 7.37085809011748e+02 3.76695713437529e+02 &

face id 2 &

7.75612143490425e+02 7.36202827550051e+02 3.79657937703356e+02 &

7.76623604261048e+02 7.38637497875149e+02 3.79313058357629e+02 &

7.77051870553251e+02 7.37085809011748e+02 3.76695713437529e+02

; block 186

poly group "10001" mat 1 con 1 &

face id 2 &

8.33864722379183e+02 7.39652653709875e+02 4.32623313919057e+02 &

8.27667286520472e+02 7.38125365214952e+02 4.29857973751925e+02 &

8.33190865039410e+02 7.35305529052256e+02 4.34142847499269e+02 &

face id 2 &

8.27667286520472e+02 7.38125365214952e+02 4.29857973751925e+02 &

8.33864722379183e+02 7.39652653709875e+02 4.32623313919057e+02 &

8.28706483747381e+02 7.39357681581388e+02 4.34620473496206e+02 &

face id 2 &

8.33190865039410e+02 7.35305529052256e+02 4.34142847499269e+02 &

8.27667286520472e+02 7.38125365214952e+02 4.29857973751925e+02 &

8.28706483747381e+02 7.39357681581388e+02 4.34620473496206e+02 &

face id 2 &

8.33864722379183e+02 7.39652653709875e+02 4.32623313919057e+02 &

8.33190865039410e+02 7.35305529052256e+02 4.34142847499269e+02 &

8.28706483747381e+02 7.39357681581388e+02 4.34620473496206e+02

; block 187

poly group "10001" mat 1 con 1 &

face id 1 &

8.39084289550781e+02 7.37087837102949e+02 4.16245800357892e+02 &

8.39084289550781e+02 7.42957763671875e+02 4.19336260947644e+02 &

8.39084289550781e+02 7.42957763671875e+02 4.11832527052480e+02 &

face id 2 &

8.39084289550781e+02 7.42957763671875e+02 4.19336260947644e+02 &

8.39084289550781e+02 7.37087837102949e+02 4.16245800357892e+02 &

8.33324474406425e+02 7.42957763671875e+02 4.15623786886125e+02 &

face id 1 &

8.39084289550781e+02 7.42957763671875e+02 4.11832527052480e+02 &

8.39084289550781e+02 7.42957763671875e+02 4.19336260947644e+02 &

8.33324474406425e+02 7.42957763671875e+02 4.15623786886125e+02 &

face id 2 &

8.39084289550781e+02 7.37087837102949e+02 4.16245800357892e+02 &

8.39084289550781e+02 7.42957763671875e+02 4.11832527052480e+02 &

8.33324474406425e+02 7.42957763671875e+02 4.15623786886125e+02

; block 188

poly group "10001" mat 1 con 1 &

face id 2 &

8.28202156483526e+02 7.42957763671875e+02 4.19788988709625e+02 &

8.26023627752663e+02 7.35851143679981e+02 4.17420806529159e+02 &

8.27273584754918e+02 7.42957763671875e+02 4.12480220146875e+02 &

face id 2 &

8.26023627752663e+02 7.35851143679981e+02 4.17420806529159e+02 &

8.28202156483526e+02 7.42957763671875e+02 4.19788988709625e+02 &

8.30534430583977e+02 7.37647109839753e+02 4.13260662990091e+02 &

face id 2 &

8.27273584754918e+02 7.42957763671875e+02 4.12480220146875e+02 &

8.26023627752663e+02 7.35851143679981e+02 4.17420806529159e+02 &

8.30534430583977e+02 7.37647109839753e+02 4.13260662990091e+02 &

face id 2 &

8.28202156483526e+02 7.42957763671875e+02 4.19788988709625e+02 &

8.27273584754918e+02 7.42957763671875e+02 4.12480220146875e+02 &

8.30534430583977e+02 7.37647109839753e+02 4.13260662990091e+02

; block 189

poly group "10001" mat 1 con 1 &

face id 2 &

8.33324474406425e+02 7.42957763671875e+02 4.15623786886125e+02 &

8.31592196255603e+02 7.37553570391624e+02 4.20696295870736e+02 &

8.28202156483526e+02 7.42957763671875e+02 4.19788988709625e+02 &

face id 2 &

8.31592196255603e+02 7.37553570391624e+02 4.20696295870736e+02 &

8.33324474406425e+02 7.42957763671875e+02 4.15623786886125e+02 &

8.30534430583977e+02 7.37647109839753e+02 4.13260662990091e+02 &

face id 2 &

8.28202156483526e+02 7.42957763671875e+02 4.19788988709625e+02 &

8.31592196255603e+02 7.37553570391624e+02 4.20696295870736e+02 &

8.30534430583977e+02 7.37647109839753e+02 4.13260662990091e+02 &

face id 2 &

8.33324474406425e+02 7.42957763671875e+02 4.15623786886125e+02 &

8.28202156483526e+02 7.42957763671875e+02 4.19788988709625e+02 &

8.30534430583977e+02 7.37647109839753e+02 4.13260662990091e+02

; block 190

poly group "10001" mat 1 con 1 &

face id 1 &

7.90621013779993e+02 7.42957763671875e+02 3.98748321284644e+02 &

7.93739440525348e+02 7.42957763671875e+02 3.96852948504114e+02 &

7.94070870845246e+02 7.42957763671875e+02 4.00551068462923e+02 &

face id 2 &

7.93739440525348e+02 7.42957763671875e+02 3.96852948504114e+02 &

7.90621013779993e+02 7.42957763671875e+02 3.98748321284644e+02 &

7.92609359140741e+02 7.39887378961859e+02 3.99568383832409e+02 &

face id 2 &

7.94070870845246e+02 7.42957763671875e+02 4.00551068462923e+02 &

7.93739440525348e+02 7.42957763671875e+02 3.96852948504114e+02 &

7.92609359140741e+02 7.39887378961859e+02 3.99568383832409e+02 &

face id 2 &

7.90621013779993e+02 7.42957763671875e+02 3.98748321284644e+02 &

7.94070870845246e+02 7.42957763671875e+02 4.00551068462923e+02 &

7.92609359140741e+02 7.39887378961859e+02 3.99568383832409e+02

; block 191

poly group "10001" mat 1 con 1 &

face id 2 &

7.91408350694546e+02 7.37046149302478e+02 3.97065808643254e+02 &

7.90851304530732e+02 7.36812816901129e+02 3.93877274718733e+02 &

7.92078395752426e+02 7.40216819168812e+02 3.95906231480863e+02 &

face id 2 &

7.90851304530732e+02 7.36812816901129e+02 3.93877274718733e+02 &

7.91408350694546e+02 7.37046149302478e+02 3.97065808643254e+02 &

7.94862612915045e+02 7.36904823128870e+02 3.94552802867836e+02 &

face id 2 &

7.92078395752426e+02 7.40216819168812e+02 3.95906231480863e+02 &

7.90851304530732e+02 7.36812816901129e+02 3.93877274718733e+02 &

7.94862612915045e+02 7.36904823128870e+02 3.94552802867836e+02 &

face id 2 &

7.91408350694546e+02 7.37046149302478e+02 3.97065808643254e+02 &

7.92078395752426e+02 7.40216819168812e+02 3.95906231480863e+02 &

7.94862612915045e+02 7.36904823128870e+02 3.94552802867836e+02

; block 192

poly group "10001" mat 1 con 1 &

face id 2 &

7.90851304530732e+02 7.36812816901129e+02 3.93877274718733e+02 &

7.91408350694546e+02 7.37046149302478e+02 3.97065808643254e+02 &

7.93143690467033e+02 7.34153895053626e+02 3.94736213202014e+02 &

face id 2 &

7.91408350694546e+02 7.37046149302478e+02 3.97065808643254e+02 &

7.90851304530732e+02 7.36812816901129e+02 3.93877274718733e+02 &

7.94862612915045e+02 7.36904823128870e+02 3.94552802867836e+02 &

face id 2 &

7.93143690467033e+02 7.34153895053626e+02 3.94736213202014e+02 &

7.91408350694546e+02 7.37046149302478e+02 3.97065808643254e+02 &

7.94862612915045e+02 7.36904823128870e+02 3.94552802867836e+02 &

face id 2 &

7.90851304530732e+02 7.36812816901129e+02 3.93877274718733e+02 &

7.93143690467033e+02 7.34153895053626e+02 3.94736213202014e+02 &

7.94862612915045e+02 7.36904823128870e+02 3.94552802867836e+02

; block 193

poly group "10001" mat 1 con 1 &

face id 2 &

8.07996067368936e+02 7.40284697371919e+02 4.24709539056186e+02 &

8.10989743959868e+02 7.42957763671875e+02 4.23463792596016e+02 &

8.07173533475188e+02 7.42957763671875e+02 4.22397635676665e+02 &

face id 2 &

8.10989743959868e+02 7.42957763671875e+02 4.23463792596016e+02 &

8.07996067368936e+02 7.40284697371919e+02 4.24709539056186e+02 &

8.08068987260185e+02 7.42957763671875e+02 4.26046463140405e+02 &

face id 1 &

8.07173533475188e+02 7.42957763671875e+02 4.22397635676665e+02 &

8.10989743959868e+02 7.42957763671875e+02 4.23463792596016e+02 &

8.08068987260185e+02 7.42957763671875e+02 4.26046463140405e+02 &

face id 1 &

8.07996067368936e+02 7.40284697371919e+02 4.24709539056186e+02 &

8.07173533475188e+02 7.42957763671875e+02 4.22397635676665e+02 &

8.08068987260185e+02 7.42957763671875e+02 4.26046463140405e+02

; block 194

poly group "10001" mat 1 con 1 &

face id 2 &

8.08606526611431e+02 7.39255690450706e+02 4.17166663969857e+02 &

8.10593672000049e+02 7.39659613160195e+02 4.21874911950142e+02 &

8.06636523711380e+02 7.37057270407080e+02 4.20274993998921e+02 &

face id 2 &

8.10593672000049e+02 7.39659613160195e+02 4.21874911950142e+02 &

8.08606526611431e+02 7.39255690450706e+02 4.17166663969857e+02 &

8.09490919631596e+02 7.36167724749901e+02 4.19676489619966e+02 &

face id 2 &

8.06636523711380e+02 7.37057270407080e+02 4.20274993998921e+02 &

8.10593672000049e+02 7.39659613160195e+02 4.21874911950142e+02 &

8.09490919631596e+02 7.36167724749901e+02 4.19676489619966e+02 &

face id 2 &

8.08606526611431e+02 7.39255690450706e+02 4.17166663969857e+02 &

8.06636523711380e+02 7.37057270407080e+02 4.20274993998921e+02 &

8.09490919631596e+02 7.36167724749901e+02 4.19676489619966e+02

; block 195

poly group "10001" mat 1 con 1 &

face id 1 &

8.08037597656250e+02 7.37380371093750e+02 4.23707122802734e+02 &

8.06693366792638e+02 7.40079978609543e+02 4.21147459767035e+02 &

8.07996067368936e+02 7.40284697371919e+02 4.24709539056186e+02 &

face id 2 &

8.06693366792638e+02 7.40079978609543e+02 4.21147459767035e+02 &

8.08037597656250e+02 7.37380371093750e+02 4.23707122802734e+02 &

8.10593672000049e+02 7.39659613160195e+02 4.21874911950142e+02 &

face id 2 &

8.07996067368936e+02 7.40284697371919e+02 4.24709539056186e+02 &

8.06693366792638e+02 7.40079978609543e+02 4.21147459767035e+02 &

8.10593672000049e+02 7.39659613160195e+02 4.21874911950142e+02 &

face id 2 &

8.08037597656250e+02 7.37380371093750e+02 4.23707122802734e+02 &

8.07996067368936e+02 7.40284697371919e+02 4.24709539056186e+02 &

8.10593672000049e+02 7.39659613160195e+02 4.21874911950142e+02

; block 196

poly group "10001" mat 1 con 1 &

face id 2 &

8.18118163916663e+02 7.35686889324296e+02 4.43062112321898e+02 &

8.18750826609427e+02 7.36171568127837e+02 4.40122380486379e+02 &

8.16096137577365e+02 7.37091327203198e+02 4.42538748237349e+02 &

face id 2 &

8.18750826609427e+02 7.36171568127837e+02 4.40122380486379e+02 &

8.18118163916663e+02 7.35686889324296e+02 4.43062112321898e+02 &

8.19470217536442e+02 7.38262925844703e+02 4.41377617023149e+02 &

face id 2 &

8.16096137577365e+02 7.37091327203198e+02 4.42538748237349e+02 &

8.18750826609427e+02 7.36171568127837e+02 4.40122380486379e+02 &

8.19470217536442e+02 7.38262925844703e+02 4.41377617023149e+02 &

face id 2 &

8.18118163916663e+02 7.35686889324296e+02 4.43062112321898e+02 &

8.16096137577365e+02 7.37091327203198e+02 4.42538748237349e+02 &

8.19470217536442e+02 7.38262925844703e+02 4.41377617023149e+02

; block 197

poly group "10001" mat 1 con 1 &

face id 1 &

8.09809660917787e+02 7.42957763671875e+02 4.19037083632094e+02 &

8.10989743959868e+02 7.42957763671875e+02 4.23463792596016e+02 &

8.07173533475188e+02 7.42957763671875e+02 4.22397635676665e+02 &

face id 2 &

8.10989743959868e+02 7.42957763671875e+02 4.23463792596016e+02 &

8.09809660917787e+02 7.42957763671875e+02 4.19037083632094e+02 &

8.10593672000049e+02 7.39659613160195e+02 4.21874911950142e+02 &

face id 2 &

8.07173533475188e+02 7.42957763671875e+02 4.22397635676665e+02 &

8.10989743959868e+02 7.42957763671875e+02 4.23463792596016e+02 &

8.10593672000049e+02 7.39659613160195e+02 4.21874911950142e+02 &

face id 2 &

8.09809660917787e+02 7.42957763671875e+02 4.19037083632094e+02 &

8.07173533475188e+02 7.42957763671875e+02 4.22397635676665e+02 &

8.10593672000049e+02 7.39659613160195e+02 4.21874911950142e+02

; block 198

poly group "10001" mat 1 con 1 &

face id 2 &

8.11683577279594e+02 7.42957763671875e+02 4.26990978647697e+02 &

8.10984080669042e+02 7.39211793104898e+02 4.26585347528298e+02 &

8.10989743959868e+02 7.42957763671875e+02 4.23463792596016e+02 &

face id 2 &

8.10984080669042e+02 7.39211793104898e+02 4.26585347528298e+02 &

8.11683577279594e+02 7.42957763671875e+02 4.26990978647697e+02 &

8.13756389123963e+02 7.39589893800934e+02 4.25916239240713e+02 &

face id 2 &

8.10989743959868e+02 7.42957763671875e+02 4.23463792596016e+02 &

8.10984080669042e+02 7.39211793104898e+02 4.26585347528298e+02 &

8.13756389123963e+02 7.39589893800934e+02 4.25916239240713e+02 &

face id 2 &

8.11683577279594e+02 7.42957763671875e+02 4.26990978647697e+02 &

8.10989743959868e+02 7.42957763671875e+02 4.23463792596016e+02 &

8.13756389123963e+02 7.39589893800934e+02 4.25916239240713e+02

; block 199

poly group "10001" mat 1 con 1 &

face id 2 &

8.08320277092379e+02 7.40548022708132e+02 4.28306475041886e+02 &

8.08773900100900e+02 7.42957763671875e+02 4.29399355054270e+02 &

8.11683577279594e+02 7.42957763671875e+02 4.26990978647697e+02 &

face id 1 &

8.08773900100900e+02 7.42957763671875e+02 4.29399355054270e+02 &

8.08320277092379e+02 7.40548022708132e+02 4.28306475041886e+02 &

8.08068987260185e+02 7.42957763671875e+02 4.26046463140405e+02 &

face id 1 &

8.11683577279594e+02 7.42957763671875e+02 4.26990978647697e+02 &

8.08773900100900e+02 7.42957763671875e+02 4.29399355054270e+02 &

8.08068987260185e+02 7.42957763671875e+02 4.26046463140405e+02 &

face id 2 &

8.08320277092379e+02 7.40548022708132e+02 4.28306475041886e+02 &

8.11683577279594e+02 7.42957763671875e+02 4.26990978647697e+02 &

8.08068987260185e+02 7.42957763671875e+02 4.26046463140405e+02

; block 200

poly group "10001" mat 1 con 1 &

face id 2 &

8.22822252675870e+02 7.36637749364954e+02 4.41131123199765e+02 &

8.18750826609427e+02 7.36171568127837e+02 4.40122380486379e+02 &

8.19953904426496e+02 7.34738927486142e+02 4.42268893279197e+02 &

face id 2 &

8.18750826609427e+02 7.36171568127837e+02 4.40122380486379e+02 &

8.22822252675870e+02 7.36637749364954e+02 4.41131123199765e+02 &

8.19470217536442e+02 7.38262925844703e+02 4.41377617023149e+02 &

face id 2 &

8.19953904426496e+02 7.34738927486142e+02 4.42268893279197e+02 &

8.18750826609427e+02 7.36171568127837e+02 4.40122380486379e+02 &

8.19470217536442e+02 7.38262925844703e+02 4.41377617023149e+02 &

face id 2 &

8.22822252675870e+02 7.36637749364954e+02 4.41131123199765e+02 &

8.19953904426496e+02 7.34738927486142e+02 4.42268893279197e+02 &

8.19470217536442e+02 7.38262925844703e+02 4.41377617023149e+02

; block 201

poly group "10001" mat 1 con 1 &

face id 2 &

7.91171804299718e+02 7.42957763671875e+02 3.69060849676871e+02 &

7.94048121019450e+02 7.39434762152529e+02 3.69924562422854e+02 &

7.93502350955747e+02 7.42957763671875e+02 3.73386897874445e+02 &

face id 2 &

7.94048121019450e+02 7.39434762152529e+02 3.69924562422854e+02 &

7.91171804299718e+02 7.42957763671875e+02 3.69060849676871e+02 &

7.89220069374438e+02 7.39019972726502e+02 3.69753861949652e+02 &

face id 2 &

7.93502350955747e+02 7.42957763671875e+02 3.73386897874445e+02 &

7.94048121019450e+02 7.39434762152529e+02 3.69924562422854e+02 &

7.89220069374438e+02 7.39019972726502e+02 3.69753861949652e+02 &

face id 2 &

7.91171804299718e+02 7.42957763671875e+02 3.69060849676871e+02 &

7.93502350955747e+02 7.42957763671875e+02 3.73386897874445e+02 &

7.89220069374438e+02 7.39019972726502e+02 3.69753861949652e+02

; block 202

poly group "10001" mat 1 con 1 &

face id 2 &

8.15566677879630e+02 7.39926095892137e+02 4.33708679063442e+02 &

8.15983052506966e+02 7.36922527517859e+02 4.33218426937274e+02 &

8.18073172819596e+02 7.39472123297221e+02 4.29992682558518e+02 &

face id 2 &

8.15983052506966e+02 7.36922527517859e+02 4.33218426937274e+02 &

8.15566677879630e+02 7.39926095892137e+02 4.33708679063442e+02 &

8.18859811694220e+02 7.36907823701414e+02 4.33778095113987e+02 &

face id 2 &

8.18073172819596e+02 7.39472123297221e+02 4.29992682558518e+02 &

8.15983052506966e+02 7.36922527517859e+02 4.33218426937274e+02 &

8.18859811694220e+02 7.36907823701414e+02 4.33778095113987e+02 &

face id 2 &

8.15566677879630e+02 7.39926095892137e+02 4.33708679063442e+02 &

8.18073172819596e+02 7.39472123297221e+02 4.29992682558518e+02 &

8.18859811694220e+02 7.36907823701414e+02 4.33778095113987e+02

; block 203

poly group "10001" mat 1 con 1 &

face id 2 &

7.74716057886906e+02 7.34040302468977e+02 3.74164033232456e+02 &

7.73931732739116e+02 7.36394807165182e+02 3.74019289682300e+02 &

7.76629831384941e+02 7.34030537730047e+02 3.76463041133043e+02 &

face id 2 &

7.73931732739116e+02 7.36394807165182e+02 3.74019289682300e+02 &

7.74716057886906e+02 7.34040302468977e+02 3.74164033232456e+02 &

7.76873874052688e+02 7.34965282604117e+02 3.73088491535245e+02 &

face id 2 &

7.76629831384941e+02 7.34030537730047e+02 3.76463041133043e+02 &

7.73931732739116e+02 7.36394807165182e+02 3.74019289682300e+02 &

7.76873874052688e+02 7.34965282604117e+02 3.73088491535245e+02 &

face id 2 &

7.74716057886906e+02 7.34040302468977e+02 3.74164033232456e+02 &

7.76629831384941e+02 7.34030537730047e+02 3.76463041133043e+02 &

7.76873874052688e+02 7.34965282604117e+02 3.73088491535245e+02

; block 204

poly group "10001" mat 1 con 1 &

face id 2 &

7.73931732739116e+02 7.36394807165182e+02 3.74019289682300e+02 &

7.74716057886906e+02 7.34040302468977e+02 3.74164033232456e+02 &

7.73884979376549e+02 7.35280033613756e+02 3.71475020333047e+02 &

face id 2 &

7.74716057886906e+02 7.34040302468977e+02 3.74164033232456e+02 &

7.73931732739116e+02 7.36394807165182e+02 3.74019289682300e+02 &

7.76873874052688e+02 7.34965282604117e+02 3.73088491535245e+02 &

face id 2 &

7.73884979376549e+02 7.35280033613756e+02 3.71475020333047e+02 &

7.74716057886906e+02 7.34040302468977e+02 3.74164033232456e+02 &

7.76873874052688e+02 7.34965282604117e+02 3.73088491535245e+02 &

face id 2 &

7.73931732739116e+02 7.36394807165182e+02 3.74019289682300e+02 &

7.73884979376549e+02 7.35280033613756e+02 3.71475020333047e+02 &

7.76873874052688e+02 7.34965282604117e+02 3.73088491535245e+02

; block 205

poly group "10001" mat 1 con 1 &

face id 1 &

8.01714828031126e+02 7.31153198242188e+02 3.79707377436264e+02 &

8.05419381102940e+02 7.31153198242188e+02 3.75640406494442e+02 &

7.98996834975274e+02 7.31153198242188e+02 3.74796954397793e+02 &

face id 2 &

8.05419381102940e+02 7.31153198242188e+02 3.75640406494442e+02 &

8.01714828031126e+02 7.31153198242188e+02 3.79707377436264e+02 &

8.02547639341709e+02 7.36329323377224e+02 3.73878279205792e+02 &

face id 2 &

7.98996834975274e+02 7.31153198242188e+02 3.74796954397793e+02 &

8.05419381102940e+02 7.31153198242188e+02 3.75640406494442e+02 &

8.02547639341709e+02 7.36329323377224e+02 3.73878279205792e+02 &

face id 2 &

8.01714828031126e+02 7.31153198242188e+02 3.79707377436264e+02 &

7.98996834975274e+02 7.31153198242188e+02 3.74796954397793e+02 &

8.02547639341709e+02 7.36329323377224e+02 3.73878279205792e+02

; block 206

poly group "10001" mat 1 con 1 &

face id 2 &

7.98996834975274e+02 7.31153198242188e+02 3.74796954397793e+02 &

7.98831708908744e+02 7.36125522704012e+02 3.77926771882722e+02 &

8.01714828031126e+02 7.31153198242188e+02 3.79707377436264e+02 &

face id 2 &

7.98831708908744e+02 7.36125522704012e+02 3.77926771882722e+02 &

7.98996834975274e+02 7.31153198242188e+02 3.74796954397793e+02 &

8.02547639341709e+02 7.36329323377224e+02 3.73878279205792e+02 &

face id 2 &

8.01714828031126e+02 7.31153198242188e+02 3.79707377436264e+02 &

7.98831708908744e+02 7.36125522704012e+02 3.77926771882722e+02 &

8.02547639341709e+02 7.36329323377224e+02 3.73878279205792e+02 &

face id 2 &

7.98996834975274e+02 7.31153198242188e+02 3.74796954397793e+02 &

8.01714828031126e+02 7.31153198242188e+02 3.79707377436264e+02 &

8.02547639341709e+02 7.36329323377224e+02 3.73878279205792e+02

; block 207

poly group "10001" mat 1 con 1 &

face id 1 &

7.96234950413729e+02 7.31153198242188e+02 3.69842370472253e+02 &

8.02420357858640e+02 7.31153198242188e+02 3.70297218122081e+02 &

7.99300744947642e+02 7.31153198242188e+02 3.64848876953125e+02 &

face id 2 &

8.02420357858640e+02 7.31153198242188e+02 3.70297218122081e+02 &

7.96234950413729e+02 7.31153198242188e+02 3.69842370472253e+02 &

7.99572291217004e+02 7.34946679237868e+02 3.70014757234137e+02 &

face id 2 &

7.99300744947642e+02 7.31153198242188e+02 3.64848876953125e+02 &

8.02420357858640e+02 7.31153198242188e+02 3.70297218122081e+02 &

7.99572291217004e+02 7.34946679237868e+02 3.70014757234137e+02 &

face id 2 &

7.96234950413729e+02 7.31153198242188e+02 3.69842370472253e+02 &

7.99300744947642e+02 7.31153198242188e+02 3.64848876953125e+02 &

7.99572291217004e+02 7.34946679237868e+02 3.70014757234137e+02

; block 208

poly group "10001" mat 1 con 1 &

face id 2 &

7.73884979376549e+02 7.35280033613756e+02 3.71475020333047e+02 &

7.77182913303155e+02 7.38045780756561e+02 3.71850481726892e+02 &

7.73931732739116e+02 7.36394807165182e+02 3.74019289682300e+02 &

face id 2 &

7.77182913303155e+02 7.38045780756561e+02 3.71850481726892e+02 &

7.73884979376549e+02 7.35280033613756e+02 3.71475020333047e+02 &

7.76873874052688e+02 7.34965282604117e+02 3.73088491535245e+02 &

face id 2 &

7.73931732739116e+02 7.36394807165182e+02 3.74019289682300e+02 &

7.77182913303155e+02 7.38045780756561e+02 3.71850481726892e+02 &

7.76873874052688e+02 7.34965282604117e+02 3.73088491535245e+02 &

face id 2 &

7.73884979376549e+02 7.35280033613756e+02 3.71475020333047e+02 &

7.73931732739116e+02 7.36394807165182e+02 3.74019289682300e+02 &

7.76873874052688e+02 7.34965282604117e+02 3.73088491535245e+02

; block 209

poly group "10001" mat 1 con 1 &

face id 2 &

8.25062590342976e+02 7.37119532782199e+02 4.02466299226852e+02 &

8.30305606368492e+02 7.31153198242188e+02 3.99328987610237e+02 &

8.22786902955485e+02 7.31153198242188e+02 4.00598299907967e+02 &

face id 2 &

8.30305606368492e+02 7.31153198242188e+02 3.99328987610237e+02 &

8.25062590342976e+02 7.37119532782199e+02 4.02466299226852e+02 &

8.30209617746932e+02 7.36595444598988e+02 3.95547397157632e+02 &

face id 2 &

8.22786902955485e+02 7.31153198242188e+02 4.00598299907967e+02 &

8.30305606368492e+02 7.31153198242188e+02 3.99328987610237e+02 &

8.30209617746932e+02 7.36595444598988e+02 3.95547397157632e+02 &

face id 2 &

8.25062590342976e+02 7.37119532782199e+02 4.02466299226852e+02 &

8.22786902955485e+02 7.31153198242188e+02 4.00598299907967e+02 &

8.30209617746932e+02 7.36595444598988e+02 3.95547397157632e+02

; block 210

poly group "10001" mat 1 con 1 &

face id 2 &

8.22751929996934e+02 7.31153198242188e+02 3.94008913980799e+02 &

8.20600098315628e+02 7.37493952138853e+02 3.97440050588930e+02 &

8.22786902955485e+02 7.31153198242188e+02 4.00598299907967e+02 &

face id 2 &

8.20600098315628e+02 7.37493952138853e+02 3.97440050588930e+02 &

8.22751929996934e+02 7.31153198242188e+02 3.94008913980799e+02 &

8.30209617746932e+02 7.36595444598988e+02 3.95547397157632e+02 &

face id 2 &

8.22786902955485e+02 7.31153198242188e+02 4.00598299907967e+02 &

8.20600098315628e+02 7.37493952138853e+02 3.97440050588930e+02 &

8.30209617746932e+02 7.36595444598988e+02 3.95547397157632e+02 &

face id 2 &

8.22751929996934e+02 7.31153198242188e+02 3.94008913980799e+02 &

8.22786902955485e+02 7.31153198242188e+02 4.00598299907967e+02 &

8.30209617746932e+02 7.36595444598988e+02 3.95547397157632e+02

; block 211

poly group "10001" mat 1 con 1 &

face id 2 &

8.20600098315628e+02 7.37493952138853e+02 3.97440050588930e+02 &

8.25062590342976e+02 7.37119532782199e+02 4.02466299226852e+02 &

8.22786902955485e+02 7.31153198242188e+02 4.00598299907967e+02 &

face id 2 &

8.25062590342976e+02 7.37119532782199e+02 4.02466299226852e+02 &

8.20600098315628e+02 7.37493952138853e+02 3.97440050588930e+02 &

8.30209617746932e+02 7.36595444598988e+02 3.95547397157632e+02 &

face id 2 &

8.22786902955485e+02 7.31153198242188e+02 4.00598299907967e+02 &

8.25062590342976e+02 7.37119532782199e+02 4.02466299226852e+02 &

8.30209617746932e+02 7.36595444598988e+02 3.95547397157632e+02 &

face id 2 &

8.20600098315628e+02 7.37493952138853e+02 3.97440050588930e+02 &

8.22786902955485e+02 7.31153198242188e+02 4.00598299907967e+02 &

8.30209617746932e+02 7.36595444598988e+02 3.95547397157632e+02

; block 212

poly group "10001" mat 1 con 1 &

face id 1 &

8.11726863481470e+02 7.42957763671875e+02 4.05255074248258e+02 &

8.06699764544697e+02 7.42957763671875e+02 4.06729796288443e+02 &

8.07623931991617e+02 7.42957763671875e+02 4.01996041631982e+02 &

face id 2 &

8.06699764544697e+02 7.42957763671875e+02 4.06729796288443e+02 &

8.11726863481470e+02 7.42957763671875e+02 4.05255074248258e+02 &

8.05741411139629e+02 7.38531717048476e+02 4.04360921641735e+02 &

face id 2 &

8.07623931991617e+02 7.42957763671875e+02 4.01996041631982e+02 &

8.06699764544697e+02 7.42957763671875e+02 4.06729796288443e+02 &

8.05741411139629e+02 7.38531717048476e+02 4.04360921641735e+02 &

face id 2 &

8.11726863481470e+02 7.42957763671875e+02 4.05255074248258e+02 &

8.07623931991617e+02 7.42957763671875e+02 4.01996041631982e+02 &

8.05741411139629e+02 7.38531717048476e+02 4.04360921641735e+02

; block 213

poly group "10001" mat 1 con 1 &

face id 1 &

8.12445679494332e+02 7.42957763671875e+02 4.14750818125651e+02 &

8.07792915601657e+02 7.42957763671875e+02 4.14815876761776e+02 &

8.09796767008882e+02 7.42957763671875e+02 4.10290671888704e+02 &

face id 2 &

8.07792915601657e+02 7.42957763671875e+02 4.14815876761776e+02 &

8.12445679494332e+02 7.42957763671875e+02 4.14750818125651e+02 &

8.09911809905185e+02 7.38789513583694e+02 4.13229940854814e+02 &

face id 2 &

8.09796767008882e+02 7.42957763671875e+02 4.10290671888704e+02 &

8.07792915601657e+02 7.42957763671875e+02 4.14815876761776e+02 &

8.09911809905185e+02 7.38789513583694e+02 4.13229940854814e+02 &

face id 2 &

8.12445679494332e+02 7.42957763671875e+02 4.14750818125651e+02 &

8.09796767008882e+02 7.42957763671875e+02 4.10290671888704e+02 &

8.09911809905185e+02 7.38789513583694e+02 4.13229940854814e+02

; block 214

poly group "10001" mat 1 con 1 &

face id 2 &

8.04465957264557e+02 7.33969583985651e+02 4.12069612436356e+02 &

8.07206434877838e+02 7.34828533348313e+02 4.13909643827570e+02 &

8.05732608301790e+02 7.31153198242188e+02 4.10478336468474e+02 &

face id 2 &

8.07206434877838e+02 7.34828533348313e+02 4.13909643827570e+02 &

8.04465957264557e+02 7.33969583985651e+02 4.12069612436356e+02 &

8.07730281172094e+02 7.35055793723661e+02 4.10604566802921e+02 &

face id 2 &

8.05732608301790e+02 7.31153198242188e+02 4.10478336468474e+02 &

8.07206434877838e+02 7.34828533348313e+02 4.13909643827570e+02 &

8.07730281172094e+02 7.35055793723661e+02 4.10604566802921e+02 &

face id 2 &

8.04465957264557e+02 7.33969583985651e+02 4.12069612436356e+02 &

8.05732608301790e+02 7.31153198242188e+02 4.10478336468474e+02 &

8.07730281172094e+02 7.35055793723661e+02 4.10604566802921e+02

; block 215

poly group "10001" mat 1 con 1 &

face id 1 &

8.02264872604041e+02 7.31153198242188e+02 4.05891525265379e+02 &

7.99684650841628e+02 7.31153198242188e+02 4.05616577582522e+02 &

8.00184267386772e+02 7.31153198242188e+02 4.08134781740007e+02 &

face id 2 &

7.99684650841628e+02 7.31153198242188e+02 4.05616577582522e+02 &

8.02264872604041e+02 7.31153198242188e+02 4.05891525265379e+02 &

8.01673851444431e+02 7.33922995712247e+02 4.07708632066640e+02 &

face id 2 &

8.00184267386772e+02 7.31153198242188e+02 4.08134781740007e+02 &

7.99684650841628e+02 7.31153198242188e+02 4.05616577582522e+02 &

8.01673851444431e+02 7.33922995712247e+02 4.07708632066640e+02 &

face id 2 &

8.02264872604041e+02 7.31153198242188e+02 4.05891525265379e+02 &

8.00184267386772e+02 7.31153198242188e+02 4.08134781740007e+02 &

8.01673851444431e+02 7.33922995712247e+02 4.07708632066640e+02

; block 216

poly group "10001" mat 1 con 1 &

face id 2 &

7.97086726809706e+02 7.31153198242188e+02 4.01394382822856e+02 &

7.96518989330910e+02 7.34092206745266e+02 4.03418560921382e+02 &

7.97602694134558e+02 7.31153198242188e+02 4.04311629734455e+02 &

face id 2 &

7.96518989330910e+02 7.34092206745266e+02 4.03418560921382e+02 &

7.97086726809706e+02 7.31153198242188e+02 4.01394382822856e+02 &

7.99432805907969e+02 7.34735523444890e+02 4.01271303355683e+02 &

face id 2 &

7.97602694134558e+02 7.31153198242188e+02 4.04311629734455e+02 &

7.96518989330910e+02 7.34092206745266e+02 4.03418560921382e+02 &

7.99432805907969e+02 7.34735523444890e+02 4.01271303355683e+02 &

face id 2 &

7.97086726809706e+02 7.31153198242188e+02 4.01394382822856e+02 &

7.97602694134558e+02 7.31153198242188e+02 4.04311629734455e+02 &

7.99432805907969e+02 7.34735523444890e+02 4.01271303355683e+02

; block 217

poly group "10001" mat 1 con 1 &

face id 1 &

7.99360324142697e+02 7.31153198242188e+02 3.98847639366796e+02 &

8.00586258626220e+02 7.31153198242188e+02 4.02680939650079e+02 &

8.03279798752908e+02 7.31153198242188e+02 3.99668089001987e+02 &

face id 2 &

8.00586258626220e+02 7.31153198242188e+02 4.02680939650079e+02 &

7.99360324142697e+02 7.31153198242188e+02 3.98847639366796e+02 &

7.99432805907969e+02 7.34735523444890e+02 4.01271303355683e+02 &

face id 2 &

8.03279798752908e+02 7.31153198242188e+02 3.99668089001987e+02 &

8.00586258626220e+02 7.31153198242188e+02 4.02680939650079e+02 &

7.99432805907969e+02 7.34735523444890e+02 4.01271303355683e+02 &

face id 2 &

7.99360324142697e+02 7.31153198242188e+02 3.98847639366796e+02 &

8.03279798752908e+02 7.31153198242188e+02 3.99668089001987e+02 &

7.99432805907969e+02 7.34735523444890e+02 4.01271303355683e+02

; block 218

poly group "10001" mat 1 con 1 &

face id 1 &

8.07623931991617e+02 7.42957763671875e+02 4.01996041631982e+02 &

8.06699764544697e+02 7.42957763671875e+02 4.06729796288443e+02 &

8.03310957544364e+02 7.42957763671875e+02 4.03779323739254e+02 &

face id 2 &

8.06699764544697e+02 7.42957763671875e+02 4.06729796288443e+02 &

8.07623931991617e+02 7.42957763671875e+02 4.01996041631982e+02 &

8.05741411139629e+02 7.38531717048476e+02 4.04360921641735e+02 &

face id 2 &

8.03310957544364e+02 7.42957763671875e+02 4.03779323739254e+02 &

8.06699764544697e+02 7.42957763671875e+02 4.06729796288443e+02 &

8.05741411139629e+02 7.38531717048476e+02 4.04360921641735e+02 &

face id 2 &

8.07623931991617e+02 7.42957763671875e+02 4.01996041631982e+02 &

8.03310957544364e+02 7.42957763671875e+02 4.03779323739254e+02 &

8.05741411139629e+02 7.38531717048476e+02 4.04360921641735e+02

; block 219

poly group "10001" mat 1 con 1 &

face id 2 &

7.99300744947642e+02 7.31153198242188e+02 3.64848876953125e+02 &

7.96608477764343e+02 7.37040518562079e+02 3.64848876953125e+02 &

7.96234950413729e+02 7.31153198242188e+02 3.69842370472253e+02 &

face id 2 &

7.96608477764343e+02 7.37040518562079e+02 3.64848876953125e+02 &

7.99300744947642e+02 7.31153198242188e+02 3.64848876953125e+02 &

7.99572291217004e+02 7.34946679237868e+02 3.70014757234137e+02 &

face id 2 &

7.96234950413729e+02 7.31153198242188e+02 3.69842370472253e+02 &

7.96608477764343e+02 7.37040518562079e+02 3.64848876953125e+02 &

7.99572291217004e+02 7.34946679237868e+02 3.70014757234137e+02 &

face id 2 &

7.99300744947642e+02 7.31153198242188e+02 3.64848876953125e+02 &

7.96234950413729e+02 7.31153198242188e+02 3.69842370472253e+02 &

7.99572291217004e+02 7.34946679237868e+02 3.70014757234137e+02

; block 220

poly group "10001" mat 1 con 1 &

face id 1 &

8.15100275115170e+02 7.31153198242188e+02 3.82923343064734e+02 &

8.12598543534452e+02 7.31153198242188e+02 3.76725379784238e+02 &

8.08062608321876e+02 7.31153198242188e+02 3.81109923197277e+02 &

face id 2 &

8.12598543534452e+02 7.31153198242188e+02 3.76725379784238e+02 &

8.15100275115170e+02 7.31153198242188e+02 3.82923343064734e+02 &

8.16038173260348e+02 7.37794690779616e+02 3.79187736116119e+02 &

face id 2 &

8.08062608321876e+02 7.31153198242188e+02 3.81109923197277e+02 &

8.12598543534452e+02 7.31153198242188e+02 3.76725379784238e+02 &

8.16038173260348e+02 7.37794690779616e+02 3.79187736116119e+02 &

face id 2 &

8.15100275115170e+02 7.31153198242188e+02 3.82923343064734e+02 &

8.08062608321876e+02 7.31153198242188e+02 3.81109923197277e+02 &

8.16038173260348e+02 7.37794690779616e+02 3.79187736116119e+02

; block 221

poly group "10001" mat 1 con 1 &

face id 2 &

8.06591027208975e+02 7.31153198242188e+02 3.96486365333883e+02 &

8.09864479417309e+02 7.34571602650726e+02 4.00066379407306e+02 &

8.11593521502643e+02 7.31153198242188e+02 3.97113810821721e+02 &

face id 2 &

8.09864479417309e+02 7.34571602650726e+02 4.00066379407306e+02 &

8.06591027208975e+02 7.31153198242188e+02 3.96486365333883e+02 &

8.06533727168147e+02 7.34687160194815e+02 3.94660458496592e+02 &

face id 2 &

8.11593521502643e+02 7.31153198242188e+02 3.97113810821721e+02 &

8.09864479417309e+02 7.34571602650726e+02 4.00066379407306e+02 &

8.06533727168147e+02 7.34687160194815e+02 3.94660458496592e+02 &

face id 2 &

8.06591027208975e+02 7.31153198242188e+02 3.96486365333883e+02 &

8.11593521502643e+02 7.31153198242188e+02 3.97113810821721e+02 &

8.06533727168147e+02 7.34687160194815e+02 3.94660458496592e+02

; block 222

poly group "10001" mat 1 con 1 &

face id 1 &

7.88049600609902e+02 7.31153198242188e+02 3.64848876953125e+02 &

7.85522935933196e+02 7.31153198242188e+02 3.68977673574438e+02 &

7.90619961402637e+02 7.31153198242188e+02 3.69409004990024e+02 &

face id 2 &

7.85522935933196e+02 7.31153198242188e+02 3.68977673574438e+02 &

7.88049600609902e+02 7.31153198242188e+02 3.64848876953125e+02 &

7.88153724348244e+02 7.34661142676503e+02 3.69505537208423e+02 &

face id 2 &

7.90619961402637e+02 7.31153198242188e+02 3.69409004990024e+02 &

7.85522935933196e+02 7.31153198242188e+02 3.68977673574438e+02 &

7.88153724348244e+02 7.34661142676503e+02 3.69505537208423e+02 &

face id 2 &

7.88049600609902e+02 7.31153198242188e+02 3.64848876953125e+02 &

7.90619961402637e+02 7.31153198242188e+02 3.69409004990024e+02 &

7.88153724348244e+02 7.34661142676503e+02 3.69505537208423e+02

; block 223

poly group "10001" mat 1 con 1 &

face id 2 &

7.79511950549799e+02 7.34457282336568e+02 3.78709527306644e+02 &

7.76629831384941e+02 7.34030537730047e+02 3.76463041133043e+02 &

7.75612143490425e+02 7.36202827550051e+02 3.79657937703356e+02 &

face id 2 &

7.76629831384941e+02 7.34030537730047e+02 3.76463041133043e+02 &

7.79511950549799e+02 7.34457282336568e+02 3.78709527306644e+02 &

7.77051870553251e+02 7.37085809011748e+02 3.76695713437529e+02 &

face id 2 &

7.75612143490425e+02 7.36202827550051e+02 3.79657937703356e+02 &

7.76629831384941e+02 7.34030537730047e+02 3.76463041133043e+02 &

7.77051870553251e+02 7.37085809011748e+02 3.76695713437529e+02 &

face id 2 &

7.79511950549799e+02 7.34457282336568e+02 3.78709527306644e+02 &

7.75612143490425e+02 7.36202827550051e+02 3.79657937703356e+02 &

7.77051870553251e+02 7.37085809011748e+02 3.76695713437529e+02

; block 224

poly group "10001" mat 1 con 1 &

face id 1 &

7.71135925292969e+02 7.35708841438567e+02 3.75363883785495e+02 &

7.71135925292969e+02 7.33451152287357e+02 3.72826619886730e+02 &

7.71135925292969e+02 7.35749939163578e+02 3.72379494377601e+02 &

face id 2 &

7.71135925292969e+02 7.33451152287357e+02 3.72826619886730e+02 &

7.71135925292969e+02 7.35708841438567e+02 3.75363883785495e+02 &

7.73931732739116e+02 7.36394807165182e+02 3.74019289682300e+02 &

face id 2 &

7.71135925292969e+02 7.35749939163578e+02 3.72379494377601e+02 &

7.71135925292969e+02 7.33451152287357e+02 3.72826619886730e+02 &

7.73931732739116e+02 7.36394807165182e+02 3.74019289682300e+02 &

face id 2 &

7.71135925292969e+02 7.35708841438567e+02 3.75363883785495e+02 &

7.71135925292969e+02 7.35749939163578e+02 3.72379494377601e+02 &

7.73931732739116e+02 7.36394807165182e+02 3.74019289682300e+02

; block 225

poly group "10001" mat 1 con 1 &

face id 2 &

7.90269754468005e+02 7.34557900438457e+02 3.99262146441341e+02 &

7.87573174580018e+02 7.34334720791543e+02 3.98838709393114e+02 &

7.87436025160242e+02 7.32691984698342e+02 3.98548675430130e+02 &

face id 2 &

7.87573174580018e+02 7.34334720791543e+02 3.98838709393114e+02 &

7.90269754468005e+02 7.34557900438457e+02 3.99262146441341e+02 &

7.88322409056619e+02 7.33102641526606e+02 4.00964819164849e+02 &

face id 1 &

7.87436025160242e+02 7.32691984698342e+02 3.98548675430130e+02 &

7.87573174580018e+02 7.34334720791543e+02 3.98838709393114e+02 &

7.88322409056619e+02 7.33102641526606e+02 4.00964819164849e+02 &

face id 2 &

7.90269754468005e+02 7.34557900438457e+02 3.99262146441341e+02 &

7.87436025160242e+02 7.32691984698342e+02 3.98548675430130e+02 &

7.88322409056619e+02 7.33102641526606e+02 4.00964819164849e+02

; block 226

poly group "10001" mat 1 con 1 &

face id 2 &

7.85546529336975e+02 7.37083937814405e+02 3.64848876953125e+02 &

7.82786128969092e+02 7.37151441981042e+02 3.68867086099698e+02 &

7.85522935933196e+02 7.31153198242188e+02 3.68977673574438e+02 &

face id 2 &

7.82786128969092e+02 7.37151441981042e+02 3.68867086099698e+02 &

7.85546529336975e+02 7.37083937814405e+02 3.64848876953125e+02 &

7.88153724348244e+02 7.34661142676503e+02 3.69505537208423e+02 &

face id 2 &

7.85522935933196e+02 7.31153198242188e+02 3.68977673574438e+02 &

7.82786128969092e+02 7.37151441981042e+02 3.68867086099698e+02 &

7.88153724348244e+02 7.34661142676503e+02 3.69505537208423e+02 &

face id 2 &

7.85546529336975e+02 7.37083937814405e+02 3.64848876953125e+02 &

7.85522935933196e+02 7.31153198242188e+02 3.68977673574438e+02 &

7.88153724348244e+02 7.34661142676503e+02 3.69505537208423e+02

; block 227

poly group "10001" mat 1 con 1 &

face id 2 &

7.88217100716273e+02 7.39280839435586e+02 3.80044098564319e+02 &

7.86150062467024e+02 7.42957763671875e+02 3.78920105584715e+02 &

7.90039169839192e+02 7.42957763671875e+02 3.77197613275622e+02 &

face id 2 &

7.86150062467024e+02 7.42957763671875e+02 3.78920105584715e+02 &

7.88217100716273e+02 7.39280839435586e+02 3.80044098564319e+02 &

7.87873418313903e+02 7.38883924129284e+02 3.75421137459169e+02 &

face id 2 &

7.90039169839192e+02 7.42957763671875e+02 3.77197613275622e+02 &

7.86150062467024e+02 7.42957763671875e+02 3.78920105584715e+02 &

7.87873418313903e+02 7.38883924129284e+02 3.75421137459169e+02 &

face id 2 &

7.88217100716273e+02 7.39280839435586e+02 3.80044098564319e+02 &

7.90039169839192e+02 7.42957763671875e+02 3.77197613275622e+02 &

7.87873418313903e+02 7.38883924129284e+02 3.75421137459169e+02

; block 228

poly group "10001" mat 1 con 1 &

face id 1 &

7.88322365919560e+02 7.31153198242188e+02 3.94575346324754e+02 &

7.89278770650053e+02 7.31153198242188e+02 3.97182633830160e+02 &

7.91329608055828e+02 7.31153198242188e+02 3.95119467848931e+02 &

face id 2 &

7.89278770650053e+02 7.31153198242188e+02 3.97182633830160e+02 &

7.88322365919560e+02 7.31153198242188e+02 3.94575346324754e+02 &

7.89909651462361e+02 7.33911936376853e+02 3.96791201524795e+02 &

face id 2 &

7.91329608055828e+02 7.31153198242188e+02 3.95119467848931e+02 &

7.89278770650053e+02 7.31153198242188e+02 3.97182633830160e+02 &

7.89909651462361e+02 7.33911936376853e+02 3.96791201524795e+02 &

face id 2 &

7.88322365919560e+02 7.31153198242188e+02 3.94575346324754e+02 &

7.91329608055828e+02 7.31153198242188e+02 3.95119467848931e+02 &

7.89909651462361e+02 7.33911936376853e+02 3.96791201524795e+02

; block 229

poly group "10001" mat 1 con 1 &

face id 1 &

7.95249005168754e+02 7.42957763671875e+02 3.77395501535433e+02 &

7.93786566901830e+02 7.42957763671875e+02 3.81227106778512e+02 &

7.90039169839192e+02 7.42957763671875e+02 3.77197613275622e+02 &

face id 2 &

7.93786566901830e+02 7.42957763671875e+02 3.81227106778512e+02 &

7.95249005168754e+02 7.42957763671875e+02 3.77395501535433e+02 &

7.91306819168008e+02 7.39396483628046e+02 3.82381074655449e+02 &

face id 2 &

7.90039169839192e+02 7.42957763671875e+02 3.77197613275622e+02 &

7.93786566901830e+02 7.42957763671875e+02 3.81227106778512e+02 &

7.91306819168008e+02 7.39396483628046e+02 3.82381074655449e+02 &

face id 2 &

7.95249005168754e+02 7.42957763671875e+02 3.77395501535433e+02 &

7.90039169839192e+02 7.42957763671875e+02 3.77197613275622e+02 &

7.91306819168008e+02 7.39396483628046e+02 3.82381074655449e+02

; block 230

poly group "10001" mat 1 con 1 &

face id 2 &

8.05722853410866e+02 7.35087646434574e+02 3.98900933743773e+02 &

8.09864479417309e+02 7.34571602650726e+02 4.00066379407306e+02 &

8.06591027208975e+02 7.31153198242188e+02 3.96486365333883e+02 &

face id 2 &

8.09864479417309e+02 7.34571602650726e+02 4.00066379407306e+02 &

8.05722853410866e+02 7.35087646434574e+02 3.98900933743773e+02 &

8.06533727168147e+02 7.34687160194815e+02 3.94660458496592e+02 &

face id 2 &

8.06591027208975e+02 7.31153198242188e+02 3.96486365333883e+02 &

8.09864479417309e+02 7.34571602650726e+02 4.00066379407306e+02 &

8.06533727168147e+02 7.34687160194815e+02 3.94660458496592e+02 &

face id 2 &

8.05722853410866e+02 7.35087646434574e+02 3.98900933743773e+02 &

8.06591027208975e+02 7.31153198242188e+02 3.96486365333883e+02 &

8.06533727168147e+02 7.34687160194815e+02 3.94660458496592e+02

; block 231

poly group "10001" mat 1 con 1 &

face id 2 &

8.10641773231557e+02 7.37725836349123e+02 3.93566404294409e+02 &

8.06109708892715e+02 7.37858790834568e+02 3.89837284033218e+02 &

8.10167029065789e+02 7.31153198242188e+02 3.92199997436435e+02 &

face id 2 &

8.06109708892715e+02 7.37858790834568e+02 3.89837284033218e+02 &

8.10641773231557e+02 7.37725836349123e+02 3.93566404294409e+02 &

8.06533727168147e+02 7.34687160194815e+02 3.94660458496592e+02 &

face id 2 &

8.10167029065789e+02 7.31153198242188e+02 3.92199997436435e+02 &

8.06109708892715e+02 7.37858790834568e+02 3.89837284033218e+02 &

8.06533727168147e+02 7.34687160194815e+02 3.94660458496592e+02 &

face id 2 &

8.10641773231557e+02 7.37725836349123e+02 3.93566404294409e+02 &

8.10167029065789e+02 7.31153198242188e+02 3.92199997436435e+02 &

8.06533727168147e+02 7.34687160194815e+02 3.94660458496592e+02

; block 232

poly group "10001" mat 1 con 1 &

face id 1 &

8.02264872604041e+02 7.31153198242188e+02 4.05891525265379e+02 &

8.04590244899594e+02 7.31153198242188e+02 4.03296348788896e+02 &

8.00586258626220e+02 7.31153198242188e+02 4.02680939650079e+02 &

face id 2 &

8.04590244899594e+02 7.31153198242188e+02 4.03296348788896e+02 &

8.02264872604041e+02 7.31153198242188e+02 4.05891525265379e+02 &

8.00896162902359e+02 7.34934397043815e+02 4.04514414125901e+02 &

face id 2 &

8.00586258626220e+02 7.31153198242188e+02 4.02680939650079e+02 &

8.04590244899594e+02 7.31153198242188e+02 4.03296348788896e+02 &

8.00896162902359e+02 7.34934397043815e+02 4.04514414125901e+02 &

face id 2 &

8.02264872604041e+02 7.31153198242188e+02 4.05891525265379e+02 &

8.00586258626220e+02 7.31153198242188e+02 4.02680939650079e+02 &

8.00896162902359e+02 7.34934397043815e+02 4.04514414125901e+02

; block 233

poly group "10001" mat 1 con 1 &

face id 1 &

8.17114515529188e+02 7.41058890054921e+02 4.43315417598528e+02 &

8.19327176779321e+02 7.42957763671875e+02 4.43648038877967e+02 &

8.20291841012187e+02 7.40631471626633e+02 4.45155902563818e+02 &

face id 2 &

8.19327176779321e+02 7.42957763671875e+02 4.43648038877967e+02 &

8.17114515529188e+02 7.41058890054921e+02 4.43315417598528e+02 &

8.21348267997198e+02 7.39634491253400e+02 4.42846308765791e+02 &

face id 2 &

8.20291841012187e+02 7.40631471626633e+02 4.45155902563818e+02 &

8.19327176779321e+02 7.42957763671875e+02 4.43648038877967e+02 &

8.21348267997198e+02 7.39634491253400e+02 4.42846308765791e+02 &

face id 2 &

8.17114515529188e+02 7.41058890054921e+02 4.43315417598528e+02 &

8.20291841012187e+02 7.40631471626633e+02 4.45155902563818e+02 &

8.21348267997198e+02 7.39634491253400e+02 4.42846308765791e+02

; block 234

poly group "10001" mat 1 con 1 &

face id 2 &

7.91117097900182e+02 7.39426485780028e+02 3.91447399439320e+02 &

7.93075306390299e+02 7.37259553714495e+02 3.89912466417267e+02 &

7.95765195392309e+02 7.39396095350468e+02 3.92331740066270e+02 &

face id 2 &

7.93075306390299e+02 7.37259553714495e+02 3.89912466417267e+02 &

7.91117097900182e+02 7.39426485780028e+02 3.91447399439320e+02 &

7.94862612915045e+02 7.36904823128870e+02 3.94552802867836e+02 &

face id 2 &

7.95765195392309e+02 7.39396095350468e+02 3.92331740066270e+02 &

7.93075306390299e+02 7.37259553714495e+02 3.89912466417267e+02 &

7.94862612915045e+02 7.36904823128870e+02 3.94552802867836e+02 &

face id 2 &

7.91117097900182e+02 7.39426485780028e+02 3.91447399439320e+02 &

7.95765195392309e+02 7.39396095350468e+02 3.92331740066270e+02 &

7.94862612915045e+02 7.36904823128870e+02 3.94552802867836e+02

; block 235

poly group "10001" mat 1 con 1 &

face id 2 &

7.97607558832901e+02 7.31153198242188e+02 3.95040058596455e+02 &

7.96478775035514e+02 7.34528043696007e+02 3.93001842890862e+02 &

7.94211527624996e+02 7.31153198242188e+02 3.96061394416997e+02 &

face id 2 &

7.96478775035514e+02 7.34528043696007e+02 3.93001842890862e+02 &

7.97607558832901e+02 7.31153198242188e+02 3.95040058596455e+02 &

7.97516497216007e+02 7.34563943186781e+02 3.97383221855559e+02 &

face id 2 &

7.94211527624996e+02 7.31153198242188e+02 3.96061394416997e+02 &

7.96478775035514e+02 7.34528043696007e+02 3.93001842890862e+02 &

7.97516497216007e+02 7.34563943186781e+02 3.97383221855559e+02 &

face id 2 &

7.97607558832901e+02 7.31153198242188e+02 3.95040058596455e+02 &

7.94211527624996e+02 7.31153198242188e+02 3.96061394416997e+02 &

7.97516497216007e+02 7.34563943186781e+02 3.97383221855559e+02

; block 236

poly group "10001" mat 1 con 1 &

face id 2 &

7.94211527624996e+02 7.31153198242188e+02 3.96061394416997e+02 &

7.96478775035514e+02 7.34528043696007e+02 3.93001842890862e+02 &

7.93143690467033e+02 7.34153895053626e+02 3.94736213202014e+02 &

face id 2 &

7.96478775035514e+02 7.34528043696007e+02 3.93001842890862e+02 &

7.94211527624996e+02 7.31153198242188e+02 3.96061394416997e+02 &

7.97516497216007e+02 7.34563943186781e+02 3.97383221855559e+02 &

face id 2 &

7.93143690467033e+02 7.34153895053626e+02 3.94736213202014e+02 &

7.96478775035514e+02 7.34528043696007e+02 3.93001842890862e+02 &

7.97516497216007e+02 7.34563943186781e+02 3.97383221855559e+02 &

face id 2 &

7.94211527624996e+02 7.31153198242188e+02 3.96061394416997e+02 &

7.93143690467033e+02 7.34153895053626e+02 3.94736213202014e+02 &

7.97516497216007e+02 7.34563943186781e+02 3.97383221855559e+02

; block 237

poly group "10001" mat 1 con 1 &

face id 2 &

7.87711954462484e+02 7.42957763671875e+02 3.84301812939577e+02 &

7.91306819168008e+02 7.39396483628046e+02 3.82381074655449e+02 &

7.86847560388448e+02 7.40049255151163e+02 3.86796454113899e+02 &

face id 2 &

7.91306819168008e+02 7.39396483628046e+02 3.82381074655449e+02 &

7.87711954462484e+02 7.42957763671875e+02 3.84301812939577e+02 &

7.87710389350481e+02 7.38310198672720e+02 3.83817696746716e+02 &

face id 2 &

7.86847560388448e+02 7.40049255151163e+02 3.86796454113899e+02 &

7.91306819168008e+02 7.39396483628046e+02 3.82381074655449e+02 &

7.87710389350481e+02 7.38310198672720e+02 3.83817696746716e+02 &

face id 2 &

7.87711954462484e+02 7.42957763671875e+02 3.84301812939577e+02 &

7.86847560388448e+02 7.40049255151163e+02 3.86796454113899e+02 &

7.87710389350481e+02 7.38310198672720e+02 3.83817696746716e+02

; block 238

poly group "10001" mat 1 con 1 &

face id 2 &

7.86150062467024e+02 7.42957763671875e+02 3.78920105584715e+02 &

7.84345668476437e+02 7.40135313357215e+02 3.78526374317212e+02 &

7.85724375088134e+02 7.42957763671875e+02 3.75809207520845e+02 &

face id 2 &

7.84345668476437e+02 7.40135313357215e+02 3.78526374317212e+02 &

7.86150062467024e+02 7.42957763671875e+02 3.78920105584715e+02 &

7.87873418313903e+02 7.38883924129284e+02 3.75421137459169e+02 &

face id 2 &

7.85724375088134e+02 7.42957763671875e+02 3.75809207520845e+02 &

7.84345668476437e+02 7.40135313357215e+02 3.78526374317212e+02 &

7.87873418313903e+02 7.38883924129284e+02 3.75421137459169e+02 &

face id 2 &

7.86150062467024e+02 7.42957763671875e+02 3.78920105584715e+02 &

7.85724375088134e+02 7.42957763671875e+02 3.75809207520845e+02 &

7.87873418313903e+02 7.38883924129284e+02 3.75421137459169e+02

; block 239

poly group "10001" mat 1 con 1 &

face id 1 &

7.81919545568227e+02 7.31153198242188e+02 3.77708803974256e+02 &

7.85583521777277e+02 7.31153198242188e+02 3.77046735711661e+02 &

7.82753568127254e+02 7.31153198242188e+02 3.73220620076838e+02 &

face id 2 &

7.85583521777277e+02 7.31153198242188e+02 3.77046735711661e+02 &

7.81919545568227e+02 7.31153198242188e+02 3.77708803974256e+02 &

7.83487660963961e+02 7.35663106170653e+02 3.76533043538780e+02 &

face id 2 &

7.82753568127254e+02 7.31153198242188e+02 3.73220620076838e+02 &

7.85583521777277e+02 7.31153198242188e+02 3.77046735711661e+02 &

7.83487660963961e+02 7.35663106170653e+02 3.76533043538780e+02 &

face id 2 &

7.81919545568227e+02 7.31153198242188e+02 3.77708803974256e+02 &

7.82753568127254e+02 7.31153198242188e+02 3.73220620076838e+02 &

7.83487660963961e+02 7.35663106170653e+02 3.76533043538780e+02

; block 240

poly group "10001" mat 1 con 1 &

face id 2 &

7.82753568127254e+02 7.31153198242188e+02 3.73220620076838e+02 &

7.80279121453295e+02 7.34974032328978e+02 3.74087850984229e+02 &

7.81919545568227e+02 7.31153198242188e+02 3.77708803974256e+02 &

face id 2 &

7.80279121453295e+02 7.34974032328978e+02 3.74087850984229e+02 &

7.82753568127254e+02 7.31153198242188e+02 3.73220620076838e+02 &

7.83487660963961e+02 7.35663106170653e+02 3.76533043538780e+02 &

face id 2 &

7.81919545568227e+02 7.31153198242188e+02 3.77708803974256e+02 &

7.80279121453295e+02 7.34974032328978e+02 3.74087850984229e+02 &

7.83487660963961e+02 7.35663106170653e+02 3.76533043538780e+02 &

face id 2 &

7.82753568127254e+02 7.31153198242188e+02 3.73220620076838e+02 &

7.81919545568227e+02 7.31153198242188e+02 3.77708803974256e+02 &

7.83487660963961e+02 7.35663106170653e+02 3.76533043538780e+02

; block 241

poly group "10001" mat 1 con 1 &

face id 1 &

7.93502350955747e+02 7.42957763671875e+02 3.73386897874445e+02 &

7.91171804299718e+02 7.42957763671875e+02 3.69060849676871e+02 &

7.96802081798252e+02 7.42957763671875e+02 3.69534985512364e+02 &

face id 2 &

7.91171804299718e+02 7.42957763671875e+02 3.69060849676871e+02 &

7.93502350955747e+02 7.42957763671875e+02 3.73386897874445e+02 &

7.94048121019450e+02 7.39434762152529e+02 3.69924562422854e+02 &

face id 2 &

7.96802081798252e+02 7.42957763671875e+02 3.69534985512364e+02 &

7.91171804299718e+02 7.42957763671875e+02 3.69060849676871e+02 &

7.94048121019450e+02 7.39434762152529e+02 3.69924562422854e+02 &

face id 2 &

7.93502350955747e+02 7.42957763671875e+02 3.73386897874445e+02 &

7.96802081798252e+02 7.42957763671875e+02 3.69534985512364e+02 &

7.94048121019450e+02 7.39434762152529e+02 3.69924562422854e+02

; block 242

poly group "10001" mat 1 con 1 &

face id 2 &

7.81919545568227e+02 7.31153198242188e+02 3.77708803974256e+02 &

7.80279121453295e+02 7.34974032328978e+02 3.74087850984229e+02 &

7.79511950549799e+02 7.34457282336568e+02 3.78709527306644e+02 &

face id 2 &

7.80279121453295e+02 7.34974032328978e+02 3.74087850984229e+02 &

7.81919545568227e+02 7.31153198242188e+02 3.77708803974256e+02 &

7.83487660963961e+02 7.35663106170653e+02 3.76533043538780e+02 &

face id 2 &

7.79511950549799e+02 7.34457282336568e+02 3.78709527306644e+02 &

7.80279121453295e+02 7.34974032328978e+02 3.74087850984229e+02 &

7.83487660963961e+02 7.35663106170653e+02 3.76533043538780e+02 &

face id 2 &

7.81919545568227e+02 7.31153198242188e+02 3.77708803974256e+02 &

7.79511950549799e+02 7.34457282336568e+02 3.78709527306644e+02 &

7.83487660963961e+02 7.35663106170653e+02 3.76533043538780e+02

; block 243

poly group "10001" mat 1 con 1 &

face id 2 &

8.03993643326886e+02 7.38727533005205e+02 3.95422628877349e+02 &

8.03128066302503e+02 7.38633199556869e+02 4.01150420510148e+02 &

8.05722853410866e+02 7.35087646434574e+02 3.98900933743773e+02 &

face id 2 &

8.03128066302503e+02 7.38633199556869e+02 4.01150420510148e+02 &

8.03993643326886e+02 7.38727533005205e+02 3.95422628877349e+02 &

8.08008378521269e+02 7.38730493882952e+02 3.99001486076239e+02 &

face id 2 &

8.05722853410866e+02 7.35087646434574e+02 3.98900933743773e+02 &

8.03128066302503e+02 7.38633199556869e+02 4.01150420510148e+02 &

8.08008378521269e+02 7.38730493882952e+02 3.99001486076239e+02 &

face id 2 &

8.03993643326886e+02 7.38727533005205e+02 3.95422628877349e+02 &

8.05722853410866e+02 7.35087646434574e+02 3.98900933743773e+02 &

8.08008378521269e+02 7.38730493882952e+02 3.99001486076239e+02

; block 244

poly group "10001" mat 1 con 1 &

face id 1 &

7.97607558832901e+02 7.31153198242188e+02 3.95040058596455e+02 &

8.01878943722119e+02 7.31153198242188e+02 3.95977524084350e+02 &

8.00965458695504e+02 7.31153198242188e+02 3.91758115901608e+02 &

face id 2 &

8.01878943722119e+02 7.31153198242188e+02 3.95977524084350e+02 &

7.97607558832901e+02 7.31153198242188e+02 3.95040058596455e+02 &

8.00446633691467e+02 7.34890585746234e+02 3.94456651409666e+02 &

face id 2 &

8.00965458695504e+02 7.31153198242188e+02 3.91758115901608e+02 &

8.01878943722119e+02 7.31153198242188e+02 3.95977524084350e+02 &

8.00446633691467e+02 7.34890585746234e+02 3.94456651409666e+02 &

face id 2 &

7.97607558832901e+02 7.31153198242188e+02 3.95040058596455e+02 &

8.00965458695504e+02 7.31153198242188e+02 3.91758115901608e+02 &

8.00446633691467e+02 7.34890585746234e+02 3.94456651409666e+02

; block 245

poly group "10001" mat 1 con 1 &

face id 1 &

8.04638018864358e+02 7.31153198242188e+02 3.93249980696247e+02 &

8.06591027208975e+02 7.31153198242188e+02 3.96486365333883e+02 &

8.10167029065789e+02 7.31153198242188e+02 3.92199997436435e+02 &

face id 2 &

8.06591027208975e+02 7.31153198242188e+02 3.96486365333883e+02 &

8.04638018864358e+02 7.31153198242188e+02 3.93249980696247e+02 &

8.06533727168147e+02 7.34687160194815e+02 3.94660458496592e+02 &

face id 2 &

8.10167029065789e+02 7.31153198242188e+02 3.92199997436435e+02 &

8.06591027208975e+02 7.31153198242188e+02 3.96486365333883e+02 &

8.06533727168147e+02 7.34687160194815e+02 3.94660458496592e+02 &

face id 2 &

8.04638018864358e+02 7.31153198242188e+02 3.93249980696247e+02 &

8.10167029065789e+02 7.31153198242188e+02 3.92199997436435e+02 &

8.06533727168147e+02 7.34687160194815e+02 3.94660458496592e+02

; block 246

poly group "10001" mat 1 con 1 &

face id 1 &

8.04638018864358e+02 7.31153198242188e+02 3.93249980696247e+02 &

8.01878943722119e+02 7.31153198242188e+02 3.95977524084350e+02 &

8.06591027208975e+02 7.31153198242188e+02 3.96486365333883e+02 &

face id 2 &

8.01878943722119e+02 7.31153198242188e+02 3.95977524084350e+02 &

8.04638018864358e+02 7.31153198242188e+02 3.93249980696247e+02 &

8.06533727168147e+02 7.34687160194815e+02 3.94660458496592e+02 &

face id 2 &

8.06591027208975e+02 7.31153198242188e+02 3.96486365333883e+02 &

8.01878943722119e+02 7.31153198242188e+02 3.95977524084350e+02 &

8.06533727168147e+02 7.34687160194815e+02 3.94660458496592e+02 &

face id 2 &

8.04638018864358e+02 7.31153198242188e+02 3.93249980696247e+02 &

8.06591027208975e+02 7.31153198242188e+02 3.96486365333883e+02 &

8.06533727168147e+02 7.34687160194815e+02 3.94660458496592e+02

; block 247

poly group "10001" mat 1 con 1 &

face id 1 &

7.93739440525348e+02 7.42957763671875e+02 3.96852948504114e+02 &

7.90621013779993e+02 7.42957763671875e+02 3.98748321284644e+02 &

7.90073406559598e+02 7.42957763671875e+02 3.95264116876513e+02 &

face id 2 &

7.90621013779993e+02 7.42957763671875e+02 3.98748321284644e+02 &

7.93739440525348e+02 7.42957763671875e+02 3.96852948504114e+02 &

7.92078395752426e+02 7.40216819168812e+02 3.95906231480863e+02 &

face id 2 &

7.90073406559598e+02 7.42957763671875e+02 3.95264116876513e+02 &

7.90621013779993e+02 7.42957763671875e+02 3.98748321284644e+02 &

7.92078395752426e+02 7.40216819168812e+02 3.95906231480863e+02 &

face id 2 &

7.93739440525348e+02 7.42957763671875e+02 3.96852948504114e+02 &

7.90073406559598e+02 7.42957763671875e+02 3.95264116876513e+02 &

7.92078395752426e+02 7.40216819168812e+02 3.95906231480863e+02

; block 248

poly group "10001" mat 1 con 1 &

face id 1 &

7.90621013779993e+02 7.42957763671875e+02 3.98748321284644e+02 &

7.89373224229090e+02 7.40320850286657e+02 3.97270117705758e+02 &

7.90073406559598e+02 7.42957763671875e+02 3.95264116876513e+02 &

face id 2 &

7.89373224229090e+02 7.40320850286657e+02 3.97270117705758e+02 &

7.90621013779993e+02 7.42957763671875e+02 3.98748321284644e+02 &

7.92078395752426e+02 7.40216819168812e+02 3.95906231480863e+02 &

face id 2 &

7.90073406559598e+02 7.42957763671875e+02 3.95264116876513e+02 &

7.89373224229090e+02 7.40320850286657e+02 3.97270117705758e+02 &

7.92078395752426e+02 7.40216819168812e+02 3.95906231480863e+02 &

face id 2 &

7.90621013779993e+02 7.42957763671875e+02 3.98748321284644e+02 &

7.90073406559598e+02 7.42957763671875e+02 3.95264116876513e+02 &

7.92078395752426e+02 7.40216819168812e+02 3.95906231480863e+02

; block 249

poly group "10001" mat 1 con 1 &

face id 1 &

7.97148665066069e+02 7.42957763671875e+02 3.94421512291450e+02 &

7.97174986366449e+02 7.42957763671875e+02 3.98793721820156e+02 &

7.93739440525348e+02 7.42957763671875e+02 3.96852948504114e+02 &

face id 2 &

7.97174986366449e+02 7.42957763671875e+02 3.98793721820156e+02 &

7.97148665066069e+02 7.42957763671875e+02 3.94421512291450e+02 &

7.96021030652622e+02 7.39527701437256e+02 3.96689394205240e+02 &

face id 2 &

7.93739440525348e+02 7.42957763671875e+02 3.96852948504114e+02 &

7.97174986366449e+02 7.42957763671875e+02 3.98793721820156e+02 &

7.96021030652622e+02 7.39527701437256e+02 3.96689394205240e+02 &

face id 2 &

7.97148665066069e+02 7.42957763671875e+02 3.94421512291450e+02 &

7.93739440525348e+02 7.42957763671875e+02 3.96852948504114e+02 &

7.96021030652622e+02 7.39527701437256e+02 3.96689394205240e+02

; block 250

poly group "10001" mat 1 con 1 &

face id 2 &

7.97148665066069e+02 7.42957763671875e+02 3.94421512291450e+02 &

7.95765195392309e+02 7.39396095350468e+02 3.92331740066270e+02 &

7.99389806333365e+02 7.38801050700467e+02 3.93943509497245e+02 &

face id 2 &

7.95765195392309e+02 7.39396095350468e+02 3.92331740066270e+02 &

7.97148665066069e+02 7.42957763671875e+02 3.94421512291450e+02 &

7.96021030652622e+02 7.39527701437256e+02 3.96689394205240e+02 &

face id 2 &

7.99389806333365e+02 7.38801050700467e+02 3.93943509497245e+02 &

7.95765195392309e+02 7.39396095350468e+02 3.92331740066270e+02 &

7.96021030652622e+02 7.39527701437256e+02 3.96689394205240e+02 &

face id 2 &

7.97148665066069e+02 7.42957763671875e+02 3.94421512291450e+02 &

7.99389806333365e+02 7.38801050700467e+02 3.93943509497245e+02 &

7.96021030652622e+02 7.39527701437256e+02 3.96689394205240e+02

; block 251

poly group "10001" mat 1 con 1 &

face id 2 &

8.05097224411228e+02 7.42957763671875e+02 3.93075069571725e+02 &

8.10641773231557e+02 7.37725836349123e+02 3.93566404294409e+02 &

8.08851677477977e+02 7.42957763671875e+02 3.96344698776517e+02 &

face id 2 &

8.10641773231557e+02 7.37725836349123e+02 3.93566404294409e+02 &

8.05097224411228e+02 7.42957763671875e+02 3.93075069571725e+02 &

8.03993643326886e+02 7.38727533005205e+02 3.95422628877349e+02 &

face id 2 &

8.08851677477977e+02 7.42957763671875e+02 3.96344698776517e+02 &

8.10641773231557e+02 7.37725836349123e+02 3.93566404294409e+02 &

8.03993643326886e+02 7.38727533005205e+02 3.95422628877349e+02 &

face id 2 &

8.05097224411228e+02 7.42957763671875e+02 3.93075069571725e+02 &

8.08851677477977e+02 7.42957763671875e+02 3.96344698776517e+02 &

8.03993643326886e+02 7.38727533005205e+02 3.95422628877349e+02

; block 252

poly group "10001" mat 1 con 1 &

face id 2 &

7.92078395752426e+02 7.40216819168812e+02 3.95906231480863e+02 &

7.93739440525348e+02 7.42957763671875e+02 3.96852948504114e+02 &

7.90621013779993e+02 7.42957763671875e+02 3.98748321284644e+02 &

face id 2 &

7.93739440525348e+02 7.42957763671875e+02 3.96852948504114e+02 &

7.92078395752426e+02 7.40216819168812e+02 3.95906231480863e+02 &

7.92609359140741e+02 7.39887378961859e+02 3.99568383832409e+02 &

face id 2 &

7.90621013779993e+02 7.42957763671875e+02 3.98748321284644e+02 &

7.93739440525348e+02 7.42957763671875e+02 3.96852948504114e+02 &

7.92609359140741e+02 7.39887378961859e+02 3.99568383832409e+02 &

face id 2 &

7.92078395752426e+02 7.40216819168812e+02 3.95906231480863e+02 &

7.90621013779993e+02 7.42957763671875e+02 3.98748321284644e+02 &

7.92609359140741e+02 7.39887378961859e+02 3.99568383832409e+02

; block 253

poly group "10001" mat 1 con 1 &

face id 2 &

8.05166630720558e+02 7.39260084743635e+02 4.09062297464921e+02 &

8.09796767008882e+02 7.42957763671875e+02 4.10290671888704e+02 &

8.05043104633530e+02 7.42957763671875e+02 4.11063531291342e+02 &

face id 2 &

8.09796767008882e+02 7.42957763671875e+02 4.10290671888704e+02 &

8.05166630720558e+02 7.39260084743635e+02 4.09062297464921e+02 &

8.06053783905492e+02 7.39353100736547e+02 4.13045444506629e+02 &

face id 2 &

8.05043104633530e+02 7.42957763671875e+02 4.11063531291342e+02 &

8.09796767008882e+02 7.42957763671875e+02 4.10290671888704e+02 &

8.06053783905492e+02 7.39353100736547e+02 4.13045444506629e+02 &

face id 2 &

8.05166630720558e+02 7.39260084743635e+02 4.09062297464921e+02 &

8.05043104633530e+02 7.42957763671875e+02 4.11063531291342e+02 &

8.06053783905492e+02 7.39353100736547e+02 4.13045444506629e+02

; block 254

poly group "10001" mat 1 con 1 &

face id 1 &

8.09796767008882e+02 7.42957763671875e+02 4.10290671888704e+02 &

8.11726863481470e+02 7.42957763671875e+02 4.05255074248258e+02 &

8.15234808544163e+02 7.42957763671875e+02 4.09308534149983e+02 &

face id 2 &

8.11726863481470e+02 7.42957763671875e+02 4.05255074248258e+02 &

8.09796767008882e+02 7.42957763671875e+02 4.10290671888704e+02 &

8.12071975737130e+02 7.36741810211853e+02 4.08693605631730e+02 &

face id 2 &

8.15234808544163e+02 7.42957763671875e+02 4.09308534149983e+02 &

8.11726863481470e+02 7.42957763671875e+02 4.05255074248258e+02 &

8.12071975737130e+02 7.36741810211853e+02 4.08693605631730e+02 &

face id 2 &

8.09796767008882e+02 7.42957763671875e+02 4.10290671888704e+02 &

8.15234808544163e+02 7.42957763671875e+02 4.09308534149983e+02 &

8.12071975737130e+02 7.36741810211853e+02 4.08693605631730e+02

; block 255

poly group "10001" mat 1 con 1 &

face id 2 &

8.00184267386772e+02 7.31153198242188e+02 4.08134781740007e+02 &

8.01673851444431e+02 7.33922995712247e+02 4.07708632066640e+02 &

7.99684650841628e+02 7.31153198242188e+02 4.05616577582522e+02 &

face id 2 &

8.01673851444431e+02 7.33922995712247e+02 4.07708632066640e+02 &

8.00184267386772e+02 7.31153198242188e+02 4.08134781740007e+02 &

7.99310419325134e+02 7.33763203029805e+02 4.07433170106430e+02 &

face id 2 &

7.99684650841628e+02 7.31153198242188e+02 4.05616577582522e+02 &

8.01673851444431e+02 7.33922995712247e+02 4.07708632066640e+02 &

7.99310419325134e+02 7.33763203029805e+02 4.07433170106430e+02 &

face id 2 &

8.00184267386772e+02 7.31153198242188e+02 4.08134781740007e+02 &

7.99684650841628e+02 7.31153198242188e+02 4.05616577582522e+02 &

7.99310419325134e+02 7.33763203029805e+02 4.07433170106430e+02

; block 256

poly group "10001" mat 1 con 1 &

face id 2 &

8.05202209472656e+02 7.40051391601562e+02 4.18037109375000e+02 &

8.06053783905492e+02 7.39353100736547e+02 4.13045444506629e+02 &

8.07792915601657e+02 7.42957763671875e+02 4.14815876761776e+02 &

face id 2 &

8.06053783905492e+02 7.39353100736547e+02 4.13045444506629e+02 &

8.05202209472656e+02 7.40051391601562e+02 4.18037109375000e+02 &

8.08606526611431e+02 7.39255690450706e+02 4.17166663969857e+02 &

face id 2 &

8.07792915601657e+02 7.42957763671875e+02 4.14815876761776e+02 &

8.06053783905492e+02 7.39353100736547e+02 4.13045444506629e+02 &

8.08606526611431e+02 7.39255690450706e+02 4.17166663969857e+02 &

face id 2 &

8.05202209472656e+02 7.40051391601562e+02 4.18037109375000e+02 &

8.07792915601657e+02 7.42957763671875e+02 4.14815876761776e+02 &

8.08606526611431e+02 7.39255690450706e+02 4.17166663969857e+02

; block 257

poly group "10001" mat 1 con 1 &

face id 1 &

8.05732608301790e+02 7.31153198242188e+02 4.10478336468474e+02 &

8.02747033303860e+02 7.31153198242188e+02 4.09084254148686e+02 &

8.02808956378546e+02 7.31153198242188e+02 4.12218040733640e+02 &

face id 2 &

8.02747033303860e+02 7.31153198242188e+02 4.09084254148686e+02 &

8.05732608301790e+02 7.31153198242188e+02 4.10478336468474e+02 &

8.04465957264557e+02 7.33969583985651e+02 4.12069612436356e+02 &

face id 2 &

8.02808956378546e+02 7.31153198242188e+02 4.12218040733640e+02 &

8.02747033303860e+02 7.31153198242188e+02 4.09084254148686e+02 &

8.04465957264557e+02 7.33969583985651e+02 4.12069612436356e+02 &

face id 2 &

8.05732608301790e+02 7.31153198242188e+02 4.10478336468474e+02 &

8.02808956378546e+02 7.31153198242188e+02 4.12218040733640e+02 &

8.04465957264557e+02 7.33969583985651e+02 4.12069612436356e+02

; block 258

poly group "10001" mat 1 con 1 &

face id 2 &

7.99389806333365e+02 7.38801050700467e+02 3.93943509497245e+02 &

8.02809345752658e+02 7.35127916281908e+02 3.91487446480074e+02 &

8.00446633691467e+02 7.34890585746234e+02 3.94456651409666e+02 &

face id 2 &

8.02809345752658e+02 7.35127916281908e+02 3.91487446480074e+02 &

7.99389806333365e+02 7.38801050700467e+02 3.93943509497245e+02 &

7.99028431323575e+02 7.35618187258196e+02 3.90154438356032e+02 &

face id 2 &

8.00446633691467e+02 7.34890585746234e+02 3.94456651409666e+02 &

8.02809345752658e+02 7.35127916281908e+02 3.91487446480074e+02 &

7.99028431323575e+02 7.35618187258196e+02 3.90154438356032e+02 &

face id 2 &

7.99389806333365e+02 7.38801050700467e+02 3.93943509497245e+02 &

8.00446633691467e+02 7.34890585746234e+02 3.94456651409666e+02 &

7.99028431323575e+02 7.35618187258196e+02 3.90154438356032e+02

; block 259

poly group "10001" mat 1 con 1 &

face id 1 &

8.22751929996934e+02 7.31153198242188e+02 3.94008913980799e+02 &

8.22991116415336e+02 7.31153198242188e+02 3.85627454268575e+02 &

8.16732385140107e+02 7.31153198242188e+02 3.89315202949226e+02 &

face id 2 &

8.22991116415336e+02 7.31153198242188e+02 3.85627454268575e+02 &

8.22751929996934e+02 7.31153198242188e+02 3.94008913980799e+02 &

8.18483672968706e+02 7.37593060216477e+02 3.91417863514613e+02 &

face id 2 &

8.16732385140107e+02 7.31153198242188e+02 3.89315202949226e+02 &

8.22991116415336e+02 7.31153198242188e+02 3.85627454268575e+02 &

8.18483672968706e+02 7.37593060216477e+02 3.91417863514613e+02 &

face id 2 &

8.22751929996934e+02 7.31153198242188e+02 3.94008913980799e+02 &

8.16732385140107e+02 7.31153198242188e+02 3.89315202949226e+02 &

8.18483672968706e+02 7.37593060216477e+02 3.91417863514613e+02

; block 260

poly group "10001" mat 1 con 1 &

face id 1 &

7.94113331391958e+02 7.31153198242188e+02 3.92954018796712e+02 &

7.97607558832901e+02 7.31153198242188e+02 3.95040058596455e+02 &

7.97111944069104e+02 7.31153198242188e+02 3.90546888385563e+02 &

face id 2 &

7.97607558832901e+02 7.31153198242188e+02 3.95040058596455e+02 &

7.94113331391958e+02 7.31153198242188e+02 3.92954018796712e+02 &

7.96478775035514e+02 7.34528043696007e+02 3.93001842890862e+02 &

face id 2 &

7.97111944069104e+02 7.31153198242188e+02 3.90546888385563e+02 &

7.97607558832901e+02 7.31153198242188e+02 3.95040058596455e+02 &

7.96478775035514e+02 7.34528043696007e+02 3.93001842890862e+02 &

face id 2 &

7.94113331391958e+02 7.31153198242188e+02 3.92954018796712e+02 &

7.97111944069104e+02 7.31153198242188e+02 3.90546888385563e+02 &

7.96478775035514e+02 7.34528043696007e+02 3.93001842890862e+02

; block 261

poly group "10001" mat 1 con 1 &

face id 1 &

8.16732385140107e+02 7.31153198242188e+02 3.89315202949226e+02 &

8.10009578774608e+02 7.31153198242188e+02 3.86599906858927e+02 &

8.10167029065789e+02 7.31153198242188e+02 3.92199997436435e+02 &

face id 2 &

8.10009578774608e+02 7.31153198242188e+02 3.86599906858927e+02 &

8.16732385140107e+02 7.31153198242188e+02 3.89315202949226e+02 &

8.12290681094227e+02 7.36396429920157e+02 3.89417525587450e+02 &

face id 2 &

8.10167029065789e+02 7.31153198242188e+02 3.92199997436435e+02 &

8.10009578774608e+02 7.31153198242188e+02 3.86599906858927e+02 &

8.12290681094227e+02 7.36396429920157e+02 3.89417525587450e+02 &

face id 2 &

8.16732385140107e+02 7.31153198242188e+02 3.89315202949226e+02 &

8.10167029065789e+02 7.31153198242188e+02 3.92199997436435e+02 &

8.12290681094227e+02 7.36396429920157e+02 3.89417525587450e+02

; block 262

poly group "10001" mat 1 con 1 &

face id 1 &

8.14017271454085e+02 7.42957763671875e+02 3.83339423702604e+02 &

8.14984905319849e+02 7.42957763671875e+02 3.89627546226137e+02 &

8.09805525478267e+02 7.42957763671875e+02 3.89445971649157e+02 &

face id 2 &

8.14984905319849e+02 7.42957763671875e+02 3.89627546226137e+02 &

8.14017271454085e+02 7.42957763671875e+02 3.83339423702604e+02 &

8.12290681094227e+02 7.36396429920157e+02 3.89417525587450e+02 &

face id 2 &

8.09805525478267e+02 7.42957763671875e+02 3.89445971649157e+02 &

8.14984905319849e+02 7.42957763671875e+02 3.89627546226137e+02 &

8.12290681094227e+02 7.36396429920157e+02 3.89417525587450e+02 &

face id 2 &

8.14017271454085e+02 7.42957763671875e+02 3.83339423702604e+02 &

8.09805525478267e+02 7.42957763671875e+02 3.89445971649157e+02 &

8.12290681094227e+02 7.36396429920157e+02 3.89417525587450e+02

; block 263

poly group "10001" mat 1 con 1 &

face id 2 &

8.08062608321876e+02 7.31153198242188e+02 3.81109923197277e+02 &

8.07581738970324e+02 7.36655126437911e+02 3.84328905480290e+02 &

8.15100275115170e+02 7.31153198242188e+02 3.82923343064734e+02 &

face id 2 &

8.07581738970324e+02 7.36655126437911e+02 3.84328905480290e+02 &

8.08062608321876e+02 7.31153198242188e+02 3.81109923197277e+02 &

8.16038173260348e+02 7.37794690779616e+02 3.79187736116119e+02 &

face id 2 &

8.15100275115170e+02 7.31153198242188e+02 3.82923343064734e+02 &

8.07581738970324e+02 7.36655126437911e+02 3.84328905480290e+02 &

8.16038173260348e+02 7.37794690779616e+02 3.79187736116119e+02 &

face id 2 &

8.08062608321876e+02 7.31153198242188e+02 3.81109923197277e+02 &

8.15100275115170e+02 7.31153198242188e+02 3.82923343064734e+02 &

8.16038173260348e+02 7.37794690779616e+02 3.79187736116119e+02

; block 264

poly group "10001" mat 1 con 1 &

face id 2 &

7.76938768959141e+02 7.39984334820939e+02 3.75213120298485e+02 &

7.74275596880056e+02 7.39458613389495e+02 3.71359700373594e+02 &

7.76268518704517e+02 7.42957763671875e+02 3.72447920290831e+02 &

face id 2 &

7.74275596880056e+02 7.39458613389495e+02 3.71359700373594e+02 &

7.76938768959141e+02 7.39984334820939e+02 3.75213120298485e+02 &

7.77182913303155e+02 7.38045780756561e+02 3.71850481726892e+02 &

face id 2 &

7.76268518704517e+02 7.42957763671875e+02 3.72447920290831e+02 &

7.74275596880056e+02 7.39458613389495e+02 3.71359700373594e+02 &

7.77182913303155e+02 7.38045780756561e+02 3.71850481726892e+02 &

face id 2 &

7.76938768959141e+02 7.39984334820939e+02 3.75213120298485e+02 &

7.76268518704517e+02 7.42957763671875e+02 3.72447920290831e+02 &

7.77182913303155e+02 7.38045780756561e+02 3.71850481726892e+02

; block 265

poly group "10001" mat 1 con 1 &

face id 2 &

7.77530581111218e+02 7.42957763671875e+02 3.77862977864846e+02 &

7.79220406464566e+02 7.42957763671875e+02 3.75205861997925e+02 &

7.78505842137311e+02 7.40529325582286e+02 3.79457485086928e+02 &

face id 2 &

7.79220406464566e+02 7.42957763671875e+02 3.75205861997925e+02 &

7.77530581111218e+02 7.42957763671875e+02 3.77862977864846e+02 &

7.76938768959141e+02 7.39984334820939e+02 3.75213120298485e+02 &

face id 2 &

7.78505842137311e+02 7.40529325582286e+02 3.79457485086928e+02 &

7.79220406464566e+02 7.42957763671875e+02 3.75205861997925e+02 &

7.76938768959141e+02 7.39984334820939e+02 3.75213120298485e+02 &

face id 2 &

7.77530581111218e+02 7.42957763671875e+02 3.77862977864846e+02 &

7.78505842137311e+02 7.40529325582286e+02 3.79457485086928e+02 &

7.76938768959141e+02 7.39984334820939e+02 3.75213120298485e+02

; block 266

poly group "10001" mat 1 con 1 &

face id 1 &

7.88069545474739e+02 7.31153198242188e+02 3.73773284411828e+02 &

7.85583521777277e+02 7.31153198242188e+02 3.77046735711661e+02 &

7.90039236128757e+02 7.31153198242188e+02 3.79462533789020e+02 &

face id 2 &

7.85583521777277e+02 7.31153198242188e+02 3.77046735711661e+02 &

7.88069545474739e+02 7.31153198242188e+02 3.73773284411828e+02 &

7.87897434460258e+02 7.35374415526222e+02 3.76760851304169e+02 &

face id 2 &

7.90039236128757e+02 7.31153198242188e+02 3.79462533789020e+02 &

7.85583521777277e+02 7.31153198242188e+02 3.77046735711661e+02 &

7.87897434460258e+02 7.35374415526222e+02 3.76760851304169e+02 &

face id 2 &

7.88069545474739e+02 7.31153198242188e+02 3.73773284411828e+02 &

7.90039236128757e+02 7.31153198242188e+02 3.79462533789020e+02 &

7.87897434460258e+02 7.35374415526222e+02 3.76760851304169e+02

; block 267

poly group "10001" mat 1 con 1 &

face id 2 &

7.90039236128757e+02 7.31153198242188e+02 3.79462533789020e+02 &

7.87897434460258e+02 7.35374415526222e+02 3.76760851304169e+02 &

7.89808816568164e+02 7.35078718869257e+02 3.82888823368539e+02 &

face id 2 &

7.87897434460258e+02 7.35374415526222e+02 3.76760851304169e+02 &

7.90039236128757e+02 7.31153198242188e+02 3.79462533789020e+02 &

7.92853233387824e+02 7.36309746732437e+02 3.77720728805979e+02 &

face id 2 &

7.89808816568164e+02 7.35078718869257e+02 3.82888823368539e+02 &

7.87897434460258e+02 7.35374415526222e+02 3.76760851304169e+02 &

7.92853233387824e+02 7.36309746732437e+02 3.77720728805979e+02 &

face id 2 &

7.90039236128757e+02 7.31153198242188e+02 3.79462533789020e+02 &

7.89808816568164e+02 7.35078718869257e+02 3.82888823368539e+02 &

7.92853233387824e+02 7.36309746732437e+02 3.77720728805979e+02

; block 268

poly group "10001" mat 1 con 1 &

face id 2 &

7.91329608055828e+02 7.31153198242188e+02 3.95119467848931e+02 &

7.90048611720629e+02 7.34003260059407e+02 3.93926493742337e+02 &

7.88322365919560e+02 7.31153198242188e+02 3.94575346324754e+02 &

face id 2 &

7.90048611720629e+02 7.34003260059407e+02 3.93926493742337e+02 &

7.91329608055828e+02 7.31153198242188e+02 3.95119467848931e+02 &

7.89909651462361e+02 7.33911936376853e+02 3.96791201524795e+02 &

face id 2 &

7.88322365919560e+02 7.31153198242188e+02 3.94575346324754e+02 &

7.90048611720629e+02 7.34003260059407e+02 3.93926493742337e+02 &

7.89909651462361e+02 7.33911936376853e+02 3.96791201524795e+02 &

face id 2 &

7.91329608055828e+02 7.31153198242188e+02 3.95119467848931e+02 &

7.88322365919560e+02 7.31153198242188e+02 3.94575346324754e+02 &

7.89909651462361e+02 7.33911936376853e+02 3.96791201524795e+02

; block 269

poly group "10001" mat 1 con 1 &

face id 1 &

7.90665610154540e+02 7.31153198242188e+02 3.92006573131752e+02 &

7.88322365919560e+02 7.31153198242188e+02 3.94575346324754e+02 &

7.91329608055828e+02 7.31153198242188e+02 3.95119467848931e+02 &

face id 2 &

7.88322365919560e+02 7.31153198242188e+02 3.94575346324754e+02 &

7.90665610154540e+02 7.31153198242188e+02 3.92006573131752e+02 &

7.90048611720629e+02 7.34003260059407e+02 3.93926493742337e+02 &

face id 2 &

7.91329608055828e+02 7.31153198242188e+02 3.95119467848931e+02 &

7.88322365919560e+02 7.31153198242188e+02 3.94575346324754e+02 &

7.90048611720629e+02 7.34003260059407e+02 3.93926493742337e+02 &

face id 2 &

7.90665610154540e+02 7.31153198242188e+02 3.92006573131752e+02 &

7.91329608055828e+02 7.31153198242188e+02 3.95119467848931e+02 &

7.90048611720629e+02 7.34003260059407e+02 3.93926493742337e+02

; block 270

poly group "10001" mat 1 con 1 &

face id 1 &

8.00965458695504e+02 7.31153198242188e+02 3.91758115901608e+02 &

7.97111944069104e+02 7.31153198242188e+02 3.90546888385563e+02 &

7.97607558832901e+02 7.31153198242188e+02 3.95040058596455e+02 &

face id 2 &

7.97111944069104e+02 7.31153198242188e+02 3.90546888385563e+02 &

8.00965458695504e+02 7.31153198242188e+02 3.91758115901608e+02 &

7.96478775035514e+02 7.34528043696007e+02 3.93001842890862e+02 &

face id 2 &

7.97607558832901e+02 7.31153198242188e+02 3.95040058596455e+02 &

7.97111944069104e+02 7.31153198242188e+02 3.90546888385563e+02 &

7.96478775035514e+02 7.34528043696007e+02 3.93001842890862e+02 &

face id 2 &

8.00965458695504e+02 7.31153198242188e+02 3.91758115901608e+02 &

7.97607558832901e+02 7.31153198242188e+02 3.95040058596455e+02 &

7.96478775035514e+02 7.34528043696007e+02 3.93001842890862e+02

; block 271

poly group "10001" mat 1 con 1 &

face id 1 &

7.93269602462016e+02 7.42957763671875e+02 3.93091351036508e+02 &

7.93739440525348e+02 7.42957763671875e+02 3.96852948504114e+02 &

7.90073406559598e+02 7.42957763671875e+02 3.95264116876513e+02 &

face id 2 &

7.93739440525348e+02 7.42957763671875e+02 3.96852948504114e+02 &

7.93269602462016e+02 7.42957763671875e+02 3.93091351036508e+02 &

7.92078395752426e+02 7.40216819168812e+02 3.95906231480863e+02 &

face id 2 &

7.90073406559598e+02 7.42957763671875e+02 3.95264116876513e+02 &

7.93739440525348e+02 7.42957763671875e+02 3.96852948504114e+02 &

7.92078395752426e+02 7.40216819168812e+02 3.95906231480863e+02 &

face id 2 &

7.93269602462016e+02 7.42957763671875e+02 3.93091351036508e+02 &

7.90073406559598e+02 7.42957763671875e+02 3.95264116876513e+02 &

7.92078395752426e+02 7.40216819168812e+02 3.95906231480863e+02

; block 272

poly group "10001" mat 1 con 1 &

face id 2 &

7.90073406559598e+02 7.42957763671875e+02 3.95264116876513e+02 &

7.88840026952497e+02 7.40307337647071e+02 3.93803242777784e+02 &

7.93269602462016e+02 7.42957763671875e+02 3.93091351036508e+02 &

face id 2 &

7.88840026952497e+02 7.40307337647071e+02 3.93803242777784e+02 &

7.90073406559598e+02 7.42957763671875e+02 3.95264116876513e+02 &

7.92078395752426e+02 7.40216819168812e+02 3.95906231480863e+02 &

face id 2 &

7.93269602462016e+02 7.42957763671875e+02 3.93091351036508e+02 &

7.88840026952497e+02 7.40307337647071e+02 3.93803242777784e+02 &

7.92078395752426e+02 7.40216819168812e+02 3.95906231480863e+02 &

face id 2 &

7.90073406559598e+02 7.42957763671875e+02 3.95264116876513e+02 &

7.93269602462016e+02 7.42957763671875e+02 3.93091351036508e+02 &

7.92078395752426e+02 7.40216819168812e+02 3.95906231480863e+02

; block 273

poly group "10001" mat 1 con 1 &

face id 1 &

7.89373224229090e+02 7.40320850286657e+02 3.97270117705758e+02 &

7.88840026952497e+02 7.40307337647071e+02 3.93803242777784e+02 &

7.90073406559598e+02 7.42957763671875e+02 3.95264116876513e+02 &

face id 2 &

7.88840026952497e+02 7.40307337647071e+02 3.93803242777784e+02 &

7.89373224229090e+02 7.40320850286657e+02 3.97270117705758e+02 &

7.92078395752426e+02 7.40216819168812e+02 3.95906231480863e+02 &

face id 2 &

7.90073406559598e+02 7.42957763671875e+02 3.95264116876513e+02 &

7.88840026952497e+02 7.40307337647071e+02 3.93803242777784e+02 &

7.92078395752426e+02 7.40216819168812e+02 3.95906231480863e+02 &

face id 2 &

7.89373224229090e+02 7.40320850286657e+02 3.97270117705758e+02 &

7.90073406559598e+02 7.42957763671875e+02 3.95264116876513e+02 &

7.92078395752426e+02 7.40216819168812e+02 3.95906231480863e+02

; block 274

poly group "10001" mat 1 con 1 &

face id 2 &

7.93454192356682e+02 7.31153198242188e+02 4.04941241608256e+02 &

7.92348838848174e+02 7.33658540667173e+02 4.04746717291045e+02 &

7.92932367677273e+02 7.31153198242188e+02 4.06808293598407e+02 &

face id 2 &

7.92348838848174e+02 7.33658540667173e+02 4.04746717291045e+02 &

7.93454192356682e+02 7.31153198242188e+02 4.04941241608256e+02 &

7.94470214819418e+02 7.32872355961335e+02 4.05574487462776e+02 &

face id 2 &

7.92932367677273e+02 7.31153198242188e+02 4.06808293598407e+02 &

7.92348838848174e+02 7.33658540667173e+02 4.04746717291045e+02 &

7.94470214819418e+02 7.32872355961335e+02 4.05574487462776e+02 &

face id 2 &

7.93454192356682e+02 7.31153198242188e+02 4.04941241608256e+02 &

7.92932367677273e+02 7.31153198242188e+02 4.06808293598407e+02 &

7.94470214819418e+02 7.32872355961335e+02 4.05574487462776e+02

; block 275

poly group "10001" mat 1 con 1 &

face id 1 &

8.25835327087865e+02 7.34466611642151e+02 4.46980813661767e+02 &

8.23015788477307e+02 7.36389502141782e+02 4.45123063667697e+02 &

8.25450073242188e+02 7.38673095703125e+02 4.48417846679688e+02 &

face id 2 &

8.23015788477307e+02 7.36389502141782e+02 4.45123063667697e+02 &

8.25835327087865e+02 7.34466611642151e+02 4.46980813661767e+02 &

8.25651820219652e+02 7.36989154779364e+02 4.43463021036467e+02 &

face id 2 &

8.25450073242188e+02 7.38673095703125e+02 4.48417846679688e+02 &

8.23015788477307e+02 7.36389502141782e+02 4.45123063667697e+02 &

8.25651820219652e+02 7.36989154779364e+02 4.43463021036467e+02 &

face id 2 &

8.25835327087865e+02 7.34466611642151e+02 4.46980813661767e+02 &

8.25450073242188e+02 7.38673095703125e+02 4.48417846679688e+02 &

8.25651820219652e+02 7.36989154779364e+02 4.43463021036467e+02

; block 276

poly group "10001" mat 1 con 1 &

face id 2 &

7.93739440525348e+02 7.42957763671875e+02 3.96852948504114e+02 &

7.96021030652622e+02 7.39527701437256e+02 3.96689394205240e+02 &

7.94070870845246e+02 7.42957763671875e+02 4.00551068462923e+02 &

face id 2 &

7.96021030652622e+02 7.39527701437256e+02 3.96689394205240e+02 &

7.93739440525348e+02 7.42957763671875e+02 3.96852948504114e+02 &

7.92609359140741e+02 7.39887378961859e+02 3.99568383832409e+02 &

face id 2 &

7.94070870845246e+02 7.42957763671875e+02 4.00551068462923e+02 &

7.96021030652622e+02 7.39527701437256e+02 3.96689394205240e+02 &

7.92609359140741e+02 7.39887378961859e+02 3.99568383832409e+02 &

face id 2 &

7.93739440525348e+02 7.42957763671875e+02 3.96852948504114e+02 &

7.94070870845246e+02 7.42957763671875e+02 4.00551068462923e+02 &

7.92609359140741e+02 7.39887378961859e+02 3.99568383832409e+02

; block 277

poly group "10001" mat 1 con 1 &

face id 1 &

8.13411519164205e+02 7.42957763671875e+02 4.34074692423553e+02 &

8.09588933421108e+02 7.42957763671875e+02 4.32548179801063e+02 &

8.12413884106901e+02 7.42957763671875e+02 4.30534299325918e+02 &

face id 2 &

8.09588933421108e+02 7.42957763671875e+02 4.32548179801063e+02 &

8.13411519164205e+02 7.42957763671875e+02 4.34074692423553e+02 &

8.11491610766812e+02 7.39835605296711e+02 4.32602759907100e+02 &

face id 2 &

8.12413884106901e+02 7.42957763671875e+02 4.30534299325918e+02 &

8.09588933421108e+02 7.42957763671875e+02 4.32548179801063e+02 &

8.11491610766812e+02 7.39835605296711e+02 4.32602759907100e+02 &

face id 2 &

8.13411519164205e+02 7.42957763671875e+02 4.34074692423553e+02 &

8.12413884106901e+02 7.42957763671875e+02 4.30534299325918e+02 &

8.11491610766812e+02 7.39835605296711e+02 4.32602759907100e+02

; block 278

poly group "10001" mat 1 con 1 &

face id 2 &

7.74035965173978e+02 7.40428059095133e+02 3.67725881874126e+02 &

7.74275596880056e+02 7.39458613389495e+02 3.71359700373594e+02 &

7.71135925292969e+02 7.40290741036923e+02 3.70036277725883e+02 &

face id 2 &

7.74275596880056e+02 7.39458613389495e+02 3.71359700373594e+02 &

7.74035965173978e+02 7.40428059095133e+02 3.67725881874126e+02 &

7.74603396653911e+02 7.37387876588618e+02 3.68598092656050e+02 &

face id 2 &

7.71135925292969e+02 7.40290741036923e+02 3.70036277725883e+02 &

7.74275596880056e+02 7.39458613389495e+02 3.71359700373594e+02 &

7.74603396653911e+02 7.37387876588618e+02 3.68598092656050e+02 &

face id 2 &

7.74035965173978e+02 7.40428059095133e+02 3.67725881874126e+02 &

7.71135925292969e+02 7.40290741036923e+02 3.70036277725883e+02 &

7.74603396653911e+02 7.37387876588618e+02 3.68598092656050e+02

; block 279

poly group "10001" mat 1 con 1 &

face id 1 &

8.08923346078766e+02 7.40786003671869e+02 4.31825248624020e+02 &

8.09588933421108e+02 7.42957763671875e+02 4.32548179801063e+02 &

8.10253018028565e+02 7.40840161728360e+02 4.35461772473196e+02 &

face id 2 &

8.09588933421108e+02 7.42957763671875e+02 4.32548179801063e+02 &

8.08923346078766e+02 7.40786003671869e+02 4.31825248624020e+02 &

8.11491610766812e+02 7.39835605296711e+02 4.32602759907100e+02 &

face id 2 &

8.10253018028565e+02 7.40840161728360e+02 4.35461772473196e+02 &

8.09588933421108e+02 7.42957763671875e+02 4.32548179801063e+02 &

8.11491610766812e+02 7.39835605296711e+02 4.32602759907100e+02 &

face id 2 &

8.08923346078766e+02 7.40786003671869e+02 4.31825248624020e+02 &

8.10253018028565e+02 7.40840161728360e+02 4.35461772473196e+02 &

8.11491610766812e+02 7.39835605296711e+02 4.32602759907100e+02

; block 280

poly group "10001" mat 1 con 1 &

face id 2 &

8.13221790344536e+02 7.40692716560294e+02 4.36922508985554e+02 &

8.12285854665448e+02 7.37661675314558e+02 4.34186351604388e+02 &

8.10253018028565e+02 7.40840161728360e+02 4.35461772473196e+02 &

face id 2 &

8.12285854665448e+02 7.37661675314558e+02 4.34186351604388e+02 &

8.13221790344536e+02 7.40692716560294e+02 4.36922508985554e+02 &

8.11491610766812e+02 7.39835605296711e+02 4.32602759907100e+02 &

face id 2 &

8.10253018028565e+02 7.40840161728360e+02 4.35461772473196e+02 &

8.12285854665448e+02 7.37661675314558e+02 4.34186351604388e+02 &

8.11491610766812e+02 7.39835605296711e+02 4.32602759907100e+02 &

face id 2 &

8.13221790344536e+02 7.40692716560294e+02 4.36922508985554e+02 &

8.10253018028565e+02 7.40840161728360e+02 4.35461772473196e+02 &

8.11491610766812e+02 7.39835605296711e+02 4.32602759907100e+02

; block 281

poly group "10001" mat 1 con 1 &

face id 2 &

7.74603396653911e+02 7.37387876588618e+02 3.68598092656050e+02 &

7.71135925292969e+02 7.37570331301490e+02 3.67890739425259e+02 &

7.71135925292969e+02 7.40290741036923e+02 3.70036277725883e+02 &

face id 2 &

7.71135925292969e+02 7.37570331301490e+02 3.67890739425259e+02 &

7.74603396653911e+02 7.37387876588618e+02 3.68598092656050e+02 &

7.74035965173978e+02 7.40428059095133e+02 3.67725881874126e+02 &

face id 2 &

7.71135925292969e+02 7.40290741036923e+02 3.70036277725883e+02 &

7.71135925292969e+02 7.37570331301490e+02 3.67890739425259e+02 &

7.74035965173978e+02 7.40428059095133e+02 3.67725881874126e+02 &

face id 2 &

7.74603396653911e+02 7.37387876588618e+02 3.68598092656050e+02 &

7.71135925292969e+02 7.40290741036923e+02 3.70036277725883e+02 &

7.74035965173978e+02 7.40428059095133e+02 3.67725881874126e+02

; block 282

poly group "10001" mat 1 con 1 &

face id 1 &

8.18564195407746e+02 7.31153198242188e+02 4.35743140235295e+02 &

8.18951035607923e+02 7.32012662020112e+02 4.37866528258961e+02 &

8.21044537549873e+02 7.31153198242188e+02 4.36848956154312e+02 &

face id 2 &

8.18951035607923e+02 7.32012662020112e+02 4.37866528258961e+02 &

8.18564195407746e+02 7.31153198242188e+02 4.35743140235295e+02 &

8.19270705387996e+02 7.33695343369261e+02 4.35937073693200e+02 &

face id 2 &

8.21044537549873e+02 7.31153198242188e+02 4.36848956154312e+02 &

8.18951035607923e+02 7.32012662020112e+02 4.37866528258961e+02 &

8.19270705387996e+02 7.33695343369261e+02 4.35937073693200e+02 &

face id 2 &

8.18564195407746e+02 7.31153198242188e+02 4.35743140235295e+02 &

8.21044537549873e+02 7.31153198242188e+02 4.36848956154312e+02 &

8.19270705387996e+02 7.33695343369261e+02 4.35937073693200e+02

; block 283

poly group "10001" mat 1 con 1 &

face id 2 &

7.79511950549799e+02 7.34457282336568e+02 3.78709527306644e+02 &

7.80279121453295e+02 7.34974032328978e+02 3.74087850984229e+02 &

7.79046047912539e+02 7.31153198242188e+02 3.75832298195535e+02 &

face id 2 &

7.80279121453295e+02 7.34974032328978e+02 3.74087850984229e+02 &

7.79511950549799e+02 7.34457282336568e+02 3.78709527306644e+02 &

7.76629831384941e+02 7.34030537730047e+02 3.76463041133043e+02 &

face id 2 &

7.79046047912539e+02 7.31153198242188e+02 3.75832298195535e+02 &

7.80279121453295e+02 7.34974032328978e+02 3.74087850984229e+02 &

7.76629831384941e+02 7.34030537730047e+02 3.76463041133043e+02 &

face id 2 &

7.79511950549799e+02 7.34457282336568e+02 3.78709527306644e+02 &

7.79046047912539e+02 7.31153198242188e+02 3.75832298195535e+02 &

7.76629831384941e+02 7.34030537730047e+02 3.76463041133043e+02

; block 284

poly group "10001" mat 1 con 1 &

face id 2 &

7.88322409056619e+02 7.33102641526606e+02 4.00964819164849e+02 &

7.89669829571038e+02 7.31153198242188e+02 3.99434746165080e+02 &

7.87436025160242e+02 7.32691984698342e+02 3.98548675430130e+02 &

face id 2 &

7.89669829571038e+02 7.31153198242188e+02 3.99434746165080e+02 &

7.88322409056619e+02 7.33102641526606e+02 4.00964819164849e+02 &

7.90269754468005e+02 7.34557900438457e+02 3.99262146441341e+02 &

face id 2 &

7.87436025160242e+02 7.32691984698342e+02 3.98548675430130e+02 &

7.89669829571038e+02 7.31153198242188e+02 3.99434746165080e+02 &

7.90269754468005e+02 7.34557900438457e+02 3.99262146441341e+02 &

face id 2 &

7.88322409056619e+02 7.33102641526606e+02 4.00964819164849e+02 &

7.87436025160242e+02 7.32691984698342e+02 3.98548675430130e+02 &

7.90269754468005e+02 7.34557900438457e+02 3.99262146441341e+02

; block 285

poly group "10001" mat 1 con 1 &

face id 2 &

7.78420725006757e+02 7.31153198242188e+02 3.72043049565402e+02 &

7.80279121453295e+02 7.34974032328978e+02 3.74087850984229e+02 &

7.82753568127254e+02 7.31153198242188e+02 3.73220620076838e+02 &

face id 2 &

7.80279121453295e+02 7.34974032328978e+02 3.74087850984229e+02 &

7.78420725006757e+02 7.31153198242188e+02 3.72043049565402e+02 &

7.79101118006749e+02 7.34898937065474e+02 3.69554192649387e+02 &

face id 2 &

7.82753568127254e+02 7.31153198242188e+02 3.73220620076838e+02 &

7.80279121453295e+02 7.34974032328978e+02 3.74087850984229e+02 &

7.79101118006749e+02 7.34898937065474e+02 3.69554192649387e+02 &

face id 2 &

7.78420725006757e+02 7.31153198242188e+02 3.72043049565402e+02 &

7.82753568127254e+02 7.31153198242188e+02 3.73220620076838e+02 &

7.79101118006749e+02 7.34898937065474e+02 3.69554192649387e+02

; block 286

poly group "10001" mat 1 con 1 &

face id 2 &

7.88217100716273e+02 7.39280839435586e+02 3.80044098564319e+02 &

7.84345668476437e+02 7.40135313357215e+02 3.78526374317212e+02 &

7.86150062467024e+02 7.42957763671875e+02 3.78920105584715e+02 &

face id 2 &

7.84345668476437e+02 7.40135313357215e+02 3.78526374317212e+02 &

7.88217100716273e+02 7.39280839435586e+02 3.80044098564319e+02 &

7.87873418313903e+02 7.38883924129284e+02 3.75421137459169e+02 &

face id 2 &

7.86150062467024e+02 7.42957763671875e+02 3.78920105584715e+02 &

7.84345668476437e+02 7.40135313357215e+02 3.78526374317212e+02 &

7.87873418313903e+02 7.38883924129284e+02 3.75421137459169e+02 &

face id 2 &

7.88217100716273e+02 7.39280839435586e+02 3.80044098564319e+02 &

7.86150062467024e+02 7.42957763671875e+02 3.78920105584715e+02 &

7.87873418313903e+02 7.38883924129284e+02 3.75421137459169e+02

; block 287

poly group "10001" mat 1 con 1 &

face id 1 &

7.87659568278308e+02 7.31153198242188e+02 3.84291136540166e+02 &

7.89451548023715e+02 7.31153198242188e+02 3.88362072609427e+02 &

7.91705731367781e+02 7.31153198242188e+02 3.84855508825140e+02 &

face id 2 &

7.89451548023715e+02 7.31153198242188e+02 3.88362072609427e+02 &

7.87659568278308e+02 7.31153198242188e+02 3.84291136540166e+02 &

7.87643654364816e+02 7.34661112155498e+02 3.86945147752232e+02 &

face id 2 &

7.91705731367781e+02 7.31153198242188e+02 3.84855508825140e+02 &

7.89451548023715e+02 7.31153198242188e+02 3.88362072609427e+02 &

7.87643654364816e+02 7.34661112155498e+02 3.86945147752232e+02 &

face id 2 &

7.87659568278308e+02 7.31153198242188e+02 3.84291136540166e+02 &

7.91705731367781e+02 7.31153198242188e+02 3.84855508825140e+02 &

7.87643654364816e+02 7.34661112155498e+02 3.86945147752232e+02

; block 288

poly group "10001" mat 1 con 1 &

face id 1 &

7.85839298561526e+02 7.31153198242188e+02 3.88082261200957e+02 &

7.89451548023715e+02 7.31153198242188e+02 3.88362072609427e+02 &

7.87659568278308e+02 7.31153198242188e+02 3.84291136540166e+02 &

face id 2 &

7.89451548023715e+02 7.31153198242188e+02 3.88362072609427e+02 &

7.85839298561526e+02 7.31153198242188e+02 3.88082261200957e+02 &

7.87643654364816e+02 7.34661112155498e+02 3.86945147752232e+02 &

face id 2 &

7.87659568278308e+02 7.31153198242188e+02 3.84291136540166e+02 &

7.89451548023715e+02 7.31153198242188e+02 3.88362072609427e+02 &

7.87643654364816e+02 7.34661112155498e+02 3.86945147752232e+02 &

face id 2 &

7.85839298561526e+02 7.31153198242188e+02 3.88082261200957e+02 &

7.87659568278308e+02 7.31153198242188e+02 3.84291136540166e+02 &

7.87643654364816e+02 7.34661112155498e+02 3.86945147752232e+02

; block 289

poly group "10001" mat 1 con 1 &

face id 2 &

7.92640570936094e+02 7.34389419631712e+02 3.91408058836435e+02 &

7.91723693011833e+02 7.34565359260124e+02 3.87421651270315e+02 &

7.93253124997322e+02 7.31153198242188e+02 3.89167589760091e+02 &

face id 2 &

7.91723693011833e+02 7.34565359260124e+02 3.87421651270315e+02 &

7.92640570936094e+02 7.34389419631712e+02 3.91408058836435e+02 &

7.89197276621344e+02 7.34576411507420e+02 3.90327463427344e+02 &

face id 2 &

7.93253124997322e+02 7.31153198242188e+02 3.89167589760091e+02 &

7.91723693011833e+02 7.34565359260124e+02 3.87421651270315e+02 &

7.89197276621344e+02 7.34576411507420e+02 3.90327463427344e+02 &

face id 2 &

7.92640570936094e+02 7.34389419631712e+02 3.91408058836435e+02 &

7.93253124997322e+02 7.31153198242188e+02 3.89167589760091e+02 &

7.89197276621344e+02 7.34576411507420e+02 3.90327463427344e+02

; block 290

poly group "10001" mat 1 con 1 &

face id 2 &

7.93253124997322e+02 7.31153198242188e+02 3.89167589760091e+02 &

7.91723693011833e+02 7.34565359260124e+02 3.87421651270315e+02 &

7.89451548023715e+02 7.31153198242188e+02 3.88362072609427e+02 &

face id 2 &

7.91723693011833e+02 7.34565359260124e+02 3.87421651270315e+02 &

7.93253124997322e+02 7.31153198242188e+02 3.89167589760091e+02 &

7.89197276621344e+02 7.34576411507420e+02 3.90327463427344e+02 &

face id 2 &

7.89451548023715e+02 7.31153198242188e+02 3.88362072609427e+02 &

7.91723693011833e+02 7.34565359260124e+02 3.87421651270315e+02 &

7.89197276621344e+02 7.34576411507420e+02 3.90327463427344e+02 &

face id 2 &

7.93253124997322e+02 7.31153198242188e+02 3.89167589760091e+02 &

7.89451548023715e+02 7.31153198242188e+02 3.88362072609427e+02 &

7.89197276621344e+02 7.34576411507420e+02 3.90327463427344e+02

; block 291

poly group "10001" mat 1 con 1 &

face id 2 &

7.96012403601172e+02 7.34733593543699e+02 3.88834189030604e+02 &

7.91723693011833e+02 7.34565359260124e+02 3.87421651270315e+02 &

7.92640570936094e+02 7.34389419631712e+02 3.91408058836435e+02 &

face id 2 &

7.91723693011833e+02 7.34565359260124e+02 3.87421651270315e+02 &

7.96012403601172e+02 7.34733593543699e+02 3.88834189030604e+02 &

7.93075306390299e+02 7.37259553714495e+02 3.89912466417267e+02 &

face id 2 &

7.92640570936094e+02 7.34389419631712e+02 3.91408058836435e+02 &

7.91723693011833e+02 7.34565359260124e+02 3.87421651270315e+02 &

7.93075306390299e+02 7.37259553714495e+02 3.89912466417267e+02 &

face id 2 &

7.96012403601172e+02 7.34733593543699e+02 3.88834189030604e+02 &

7.92640570936094e+02 7.34389419631712e+02 3.91408058836435e+02 &

7.93075306390299e+02 7.37259553714495e+02 3.89912466417267e+02

; block 292

poly group "10001" mat 1 con 1 &

face id 1 &

8.26217746986868e+02 7.42957763671875e+02 4.35505283716008e+02 &

8.26215102552087e+02 7.42957763671875e+02 4.40024896299389e+02 &

8.22935017305640e+02 7.42957763671875e+02 4.38190950294301e+02 &

face id 2 &

8.26215102552087e+02 7.42957763671875e+02 4.40024896299389e+02 &

8.26217746986868e+02 7.42957763671875e+02 4.35505283716008e+02 &

8.23877178682653e+02 7.39381023327622e+02 4.36180865465919e+02 &

face id 2 &

8.22935017305640e+02 7.42957763671875e+02 4.38190950294301e+02 &

8.26215102552087e+02 7.42957763671875e+02 4.40024896299389e+02 &

8.23877178682653e+02 7.39381023327622e+02 4.36180865465919e+02 &

face id 2 &

8.26217746986868e+02 7.42957763671875e+02 4.35505283716008e+02 &

8.22935017305640e+02 7.42957763671875e+02 4.38190950294301e+02 &

8.23877178682653e+02 7.39381023327622e+02 4.36180865465919e+02

; block 293

poly group "10001" mat 1 con 1 &

face id 2 &

7.89197276621344e+02 7.34576411507420e+02 3.90327463427344e+02 &

7.92640570936094e+02 7.34389419631712e+02 3.91408058836435e+02 &

7.91723693011833e+02 7.34565359260124e+02 3.87421651270315e+02 &

face id 2 &

7.92640570936094e+02 7.34389419631712e+02 3.91408058836435e+02 &

7.89197276621344e+02 7.34576411507420e+02 3.90327463427344e+02 &

7.93075306390299e+02 7.37259553714495e+02 3.89912466417267e+02 &

face id 2 &

7.91723693011833e+02 7.34565359260124e+02 3.87421651270315e+02 &

7.92640570936094e+02 7.34389419631712e+02 3.91408058836435e+02 &

7.93075306390299e+02 7.37259553714495e+02 3.89912466417267e+02 &

face id 2 &

7.89197276621344e+02 7.34576411507420e+02 3.90327463427344e+02 &

7.91723693011833e+02 7.34565359260124e+02 3.87421651270315e+02 &

7.93075306390299e+02 7.37259553714495e+02 3.89912466417267e+02

; block 294

poly group "10001" mat 1 con 1 &

face id 2 &

8.18920370898699e+02 7.37329641873752e+02 3.85333219479087e+02 &

8.12290681094227e+02 7.36396429920157e+02 3.89417525587450e+02 &

8.14017271454085e+02 7.42957763671875e+02 3.83339423702604e+02 &

face id 2 &

8.12290681094227e+02 7.36396429920157e+02 3.89417525587450e+02 &

8.18920370898699e+02 7.37329641873752e+02 3.85333219479087e+02 &

8.16038173260348e+02 7.37794690779616e+02 3.79187736116119e+02 &

face id 2 &

8.14017271454085e+02 7.42957763671875e+02 3.83339423702604e+02 &

8.12290681094227e+02 7.36396429920157e+02 3.89417525587450e+02 &

8.16038173260348e+02 7.37794690779616e+02 3.79187736116119e+02 &

face id 2 &

8.18920370898699e+02 7.37329641873752e+02 3.85333219479087e+02 &

8.14017271454085e+02 7.42957763671875e+02 3.83339423702604e+02 &

8.16038173260348e+02 7.37794690779616e+02 3.79187736116119e+02

; block 295

poly group "10001" mat 1 con 1 &

face id 2 &

7.97607558832901e+02 7.31153198242188e+02 3.95040058596455e+02 &

8.00446633691467e+02 7.34890585746234e+02 3.94456651409666e+02 &

8.00965458695504e+02 7.31153198242188e+02 3.91758115901608e+02 &

face id 2 &

8.00446633691467e+02 7.34890585746234e+02 3.94456651409666e+02 &

7.97607558832901e+02 7.31153198242188e+02 3.95040058596455e+02 &

7.96478775035514e+02 7.34528043696007e+02 3.93001842890862e+02 &

face id 2 &

8.00965458695504e+02 7.31153198242188e+02 3.91758115901608e+02 &

8.00446633691467e+02 7.34890585746234e+02 3.94456651409666e+02 &

7.96478775035514e+02 7.34528043696007e+02 3.93001842890862e+02 &

face id 2 &

7.97607558832901e+02 7.31153198242188e+02 3.95040058596455e+02 &

8.00965458695504e+02 7.31153198242188e+02 3.91758115901608e+02 &

7.96478775035514e+02 7.34528043696007e+02 3.93001842890862e+02

; block 296

poly group "10001" mat 1 con 1 &

face id 1 &

8.05097224411228e+02 7.42957763671875e+02 3.93075069571725e+02 &

8.04424438044407e+02 7.42957763671875e+02 3.86522059563540e+02 &

8.09805525478267e+02 7.42957763671875e+02 3.89445971649157e+02 &

face id 2 &

8.04424438044407e+02 7.42957763671875e+02 3.86522059563540e+02 &

8.05097224411228e+02 7.42957763671875e+02 3.93075069571725e+02 &

8.06109708892715e+02 7.37858790834568e+02 3.89837284033218e+02 &

face id 2 &

8.09805525478267e+02 7.42957763671875e+02 3.89445971649157e+02 &

8.04424438044407e+02 7.42957763671875e+02 3.86522059563540e+02 &

8.06109708892715e+02 7.37858790834568e+02 3.89837284033218e+02 &

face id 2 &

8.05097224411228e+02 7.42957763671875e+02 3.93075069571725e+02 &

8.09805525478267e+02 7.42957763671875e+02 3.89445971649157e+02 &

8.06109708892715e+02 7.37858790834568e+02 3.89837284033218e+02

; block 297

poly group "10001" mat 1 con 1 &

face id 2 &

7.96012403601172e+02 7.34733593543699e+02 3.88834189030604e+02 &

7.96478775035514e+02 7.34528043696007e+02 3.93001842890862e+02 &

7.97111944069104e+02 7.31153198242188e+02 3.90546888385563e+02 &

face id 2 &

7.96478775035514e+02 7.34528043696007e+02 3.93001842890862e+02 &

7.96012403601172e+02 7.34733593543699e+02 3.88834189030604e+02 &

7.99028431323575e+02 7.35618187258196e+02 3.90154438356032e+02 &

face id 2 &

7.97111944069104e+02 7.31153198242188e+02 3.90546888385563e+02 &

7.96478775035514e+02 7.34528043696007e+02 3.93001842890862e+02 &

7.99028431323575e+02 7.35618187258196e+02 3.90154438356032e+02 &

face id 2 &

7.96012403601172e+02 7.34733593543699e+02 3.88834189030604e+02 &

7.97111944069104e+02 7.31153198242188e+02 3.90546888385563e+02 &

7.99028431323575e+02 7.35618187258196e+02 3.90154438356032e+02

; block 298

poly group "10001" mat 1 con 1 &

face id 1 &

7.71135925292969e+02 7.33726108156615e+02 3.76246415882706e+02 &

7.72905778333058e+02 7.35543709744398e+02 3.77622196844083e+02 &

7.72417671557342e+02 7.33094075356536e+02 3.77831986423786e+02 &

face id 2 &

7.72905778333058e+02 7.35543709744398e+02 3.77622196844083e+02 &

7.71135925292969e+02 7.33726108156615e+02 3.76246415882706e+02 &

7.74716057886906e+02 7.34040302468977e+02 3.74164033232456e+02 &

face id 2 &

7.72417671557342e+02 7.33094075356536e+02 3.77831986423786e+02 &

7.72905778333058e+02 7.35543709744398e+02 3.77622196844083e+02 &

7.74716057886906e+02 7.34040302468977e+02 3.74164033232456e+02 &

face id 2 &

7.71135925292969e+02 7.33726108156615e+02 3.76246415882706e+02 &

7.72417671557342e+02 7.33094075356536e+02 3.77831986423786e+02 &

7.74716057886906e+02 7.34040302468977e+02 3.74164033232456e+02

; block 299

poly group "10001" mat 1 con 1 &

face id 2 &

7.89669829571038e+02 7.31153198242188e+02 3.99434746165080e+02 &

7.91099717380957e+02 7.31153198242188e+02 4.01060586132354e+02 &

7.92440189486717e+02 7.33520249849107e+02 4.00528860955446e+02 &

face id 2 &

7.91099717380957e+02 7.31153198242188e+02 4.01060586132354e+02 &

7.89669829571038e+02 7.31153198242188e+02 3.99434746165080e+02 &

7.90671467575070e+02 7.33496249726850e+02 4.02031030029439e+02 &

face id 2 &

7.92440189486717e+02 7.33520249849107e+02 4.00528860955446e+02 &

7.91099717380957e+02 7.31153198242188e+02 4.01060586132354e+02 &

7.90671467575070e+02 7.33496249726850e+02 4.02031030029439e+02 &

face id 2 &

7.89669829571038e+02 7.31153198242188e+02 3.99434746165080e+02 &

7.92440189486717e+02 7.33520249849107e+02 4.00528860955446e+02 &

7.90671467575070e+02 7.33496249726850e+02 4.02031030029439e+02

; block 300

poly group "10001" mat 1 con 1 &

face id 1 &

8.18246011230260e+02 7.42957763671875e+02 3.71155657593316e+02 &

8.21241190665237e+02 7.42957763671875e+02 3.64848876953125e+02 &

8.28903935786781e+02 7.42957763671875e+02 3.71492197084357e+02 &

face id 2 &

8.21241190665237e+02 7.42957763671875e+02 3.64848876953125e+02 &

8.18246011230260e+02 7.42957763671875e+02 3.71155657593316e+02 &

8.20842798872635e+02 7.37189646407413e+02 3.71833994279653e+02 &

face id 2 &

8.28903935786781e+02 7.42957763671875e+02 3.71492197084357e+02 &

8.21241190665237e+02 7.42957763671875e+02 3.64848876953125e+02 &

8.20842798872635e+02 7.37189646407413e+02 3.71833994279653e+02 &

face id 2 &

8.18246011230260e+02 7.42957763671875e+02 3.71155657593316e+02 &

8.28903935786781e+02 7.42957763671875e+02 3.71492197084357e+02 &

8.20842798872635e+02 7.37189646407413e+02 3.71833994279653e+02

; block 301

poly group "10001" mat 1 con 1 &

face id 1 &

7.93898632039194e+02 7.42957763671875e+02 3.64848876953125e+02 &

7.96608477764343e+02 7.37040518562079e+02 3.64848876953125e+02 &

7.99831206538623e+02 7.42957763671875e+02 3.64848876953125e+02 &

face id 2 &

7.96608477764343e+02 7.37040518562079e+02 3.64848876953125e+02 &

7.93898632039194e+02 7.42957763671875e+02 3.64848876953125e+02 &

7.98072850148110e+02 7.38921841899001e+02 3.69042179430648e+02 &

face id 2 &

7.99831206538623e+02 7.42957763671875e+02 3.64848876953125e+02 &

7.96608477764343e+02 7.37040518562079e+02 3.64848876953125e+02 &

7.98072850148110e+02 7.38921841899001e+02 3.69042179430648e+02 &

face id 2 &

7.93898632039194e+02 7.42957763671875e+02 3.64848876953125e+02 &

7.99831206538623e+02 7.42957763671875e+02 3.64848876953125e+02 &

7.98072850148110e+02 7.38921841899001e+02 3.69042179430648e+02

; block 302

poly group "10001" mat 1 con 1 &

face id 2 &

7.99156871754180e+02 7.42957763671875e+02 3.74726900763980e+02 &

8.02547639341709e+02 7.36329323377224e+02 3.73878279205792e+02 &

7.96250595319160e+02 7.36616698982022e+02 3.74074088190253e+02 &

face id 2 &

8.02547639341709e+02 7.36329323377224e+02 3.73878279205792e+02 &

7.99156871754180e+02 7.42957763671875e+02 3.74726900763980e+02 &

7.98072850148110e+02 7.38921841899001e+02 3.69042179430648e+02 &

face id 2 &

7.96250595319160e+02 7.36616698982022e+02 3.74074088190253e+02 &

8.02547639341709e+02 7.36329323377224e+02 3.73878279205792e+02 &

7.98072850148110e+02 7.38921841899001e+02 3.69042179430648e+02 &

face id 2 &

7.99156871754180e+02 7.42957763671875e+02 3.74726900763980e+02 &

7.96250595319160e+02 7.36616698982022e+02 3.74074088190253e+02 &

7.98072850148110e+02 7.38921841899001e+02 3.69042179430648e+02

; block 303

poly group "10001" mat 1 con 1 &

face id 2 &

8.39084289550781e+02 7.37015214063697e+02 4.30185524731766e+02 &

8.39084289550781e+02 7.38099320041941e+02 4.35153393151707e+02 &

8.33190865039410e+02 7.35305529052256e+02 4.34142847499269e+02 &

face id 1 &

8.39084289550781e+02 7.38099320041941e+02 4.35153393151707e+02 &

8.39084289550781e+02 7.37015214063697e+02 4.30185524731766e+02 &

8.39084289550781e+02 7.31153198242188e+02 4.35338289521477e+02 &

face id 2 &

8.33190865039410e+02 7.35305529052256e+02 4.34142847499269e+02 &

8.39084289550781e+02 7.38099320041941e+02 4.35153393151707e+02 &

8.39084289550781e+02 7.31153198242188e+02 4.35338289521477e+02 &

face id 2 &

8.39084289550781e+02 7.37015214063697e+02 4.30185524731766e+02 &

8.33190865039410e+02 7.35305529052256e+02 4.34142847499269e+02 &

8.39084289550781e+02 7.31153198242188e+02 4.35338289521477e+02

; block 304

poly group "10001" mat 1 con 1 &

face id 2 &

7.98072850148110e+02 7.38921841899001e+02 3.69042179430648e+02 &

7.93658752091436e+02 7.34748621680107e+02 3.69706280758364e+02 &

7.96250595319160e+02 7.36616698982022e+02 3.74074088190253e+02 &

face id 2 &

7.93658752091436e+02 7.34748621680107e+02 3.69706280758364e+02 &

7.98072850148110e+02 7.38921841899001e+02 3.69042179430648e+02 &

7.94048121019450e+02 7.39434762152529e+02 3.69924562422854e+02 &

face id 2 &

7.96250595319160e+02 7.36616698982022e+02 3.74074088190253e+02 &

7.93658752091436e+02 7.34748621680107e+02 3.69706280758364e+02 &

7.94048121019450e+02 7.39434762152529e+02 3.69924562422854e+02 &

face id 2 &

7.98072850148110e+02 7.38921841899001e+02 3.69042179430648e+02 &

7.96250595319160e+02 7.36616698982022e+02 3.74074088190253e+02 &

7.94048121019450e+02 7.39434762152529e+02 3.69924562422854e+02

; block 305

poly group "10001" mat 1 con 1 &

face id 2 &

7.93658752091436e+02 7.34748621680107e+02 3.69706280758364e+02 &

7.98072850148110e+02 7.38921841899001e+02 3.69042179430648e+02 &

7.96608477764343e+02 7.37040518562079e+02 3.64848876953125e+02 &

face id 2 &

7.98072850148110e+02 7.38921841899001e+02 3.69042179430648e+02 &

7.93658752091436e+02 7.34748621680107e+02 3.69706280758364e+02 &

7.94048121019450e+02 7.39434762152529e+02 3.69924562422854e+02 &

face id 2 &

7.96608477764343e+02 7.37040518562079e+02 3.64848876953125e+02 &

7.98072850148110e+02 7.38921841899001e+02 3.69042179430648e+02 &

7.94048121019450e+02 7.39434762152529e+02 3.69924562422854e+02 &

face id 2 &

7.93658752091436e+02 7.34748621680107e+02 3.69706280758364e+02 &

7.96608477764343e+02 7.37040518562079e+02 3.64848876953125e+02 &

7.94048121019450e+02 7.39434762152529e+02 3.69924562422854e+02

; block 306

poly group "10001" mat 1 con 1 &

face id 1 &

8.20704762342298e+02 7.42957763671875e+02 3.77856341162262e+02 &

8.24572165467485e+02 7.42957763671875e+02 3.83668001515414e+02 &

8.19289966892218e+02 7.42957763671875e+02 3.85948308253048e+02 &

face id 2 &

8.24572165467485e+02 7.42957763671875e+02 3.83668001515414e+02 &

8.20704762342298e+02 7.42957763671875e+02 3.77856341162262e+02 &

8.18920370898699e+02 7.37329641873752e+02 3.85333219479087e+02 &

face id 2 &

8.19289966892218e+02 7.42957763671875e+02 3.85948308253048e+02 &

8.24572165467485e+02 7.42957763671875e+02 3.83668001515414e+02 &

8.18920370898699e+02 7.37329641873752e+02 3.85333219479087e+02 &

face id 2 &

8.20704762342298e+02 7.42957763671875e+02 3.77856341162262e+02 &

8.19289966892218e+02 7.42957763671875e+02 3.85948308253048e+02 &

8.18920370898699e+02 7.37329641873752e+02 3.85333219479087e+02

; block 307

poly group "10001" mat 1 con 1 &

face id 1 &

7.94535054290126e+02 7.33127082593229e+02 4.09095159059601e+02 &

7.96585601940516e+02 7.33011023940279e+02 4.10127244628184e+02 &

7.96071150094496e+02 7.31153198242188e+02 4.08799949593773e+02 &

face id 2 &

7.96585601940516e+02 7.33011023940279e+02 4.10127244628184e+02 &

7.94535054290126e+02 7.33127082593229e+02 4.09095159059601e+02 &

7.95446009423280e+02 7.33370401603083e+02 4.07686153733035e+02 &

face id 2 &

7.96071150094496e+02 7.31153198242188e+02 4.08799949593773e+02 &

7.96585601940516e+02 7.33011023940279e+02 4.10127244628184e+02 &

7.95446009423280e+02 7.33370401603083e+02 4.07686153733035e+02 &

face id 2 &

7.94535054290126e+02 7.33127082593229e+02 4.09095159059601e+02 &

7.96071150094496e+02 7.31153198242188e+02 4.08799949593773e+02 &

7.95446009423280e+02 7.33370401603083e+02 4.07686153733035e+02

; block 308

poly group "10001" mat 1 con 1 &

face id 2 &

8.15130661800511e+02 7.42957763671875e+02 4.37033689477478e+02 &

8.14838076915517e+02 7.39071150525117e+02 4.39175800078397e+02 &

8.16975670140343e+02 7.39892790962574e+02 4.39960247842898e+02 &

face id 2 &

8.14838076915517e+02 7.39071150525117e+02 4.39175800078397e+02 &

8.15130661800511e+02 7.42957763671875e+02 4.37033689477478e+02 &

8.14343169924012e+02 7.42957763671875e+02 4.40026044980605e+02 &

face id 2 &

8.16975670140343e+02 7.39892790962574e+02 4.39960247842898e+02 &

8.14838076915517e+02 7.39071150525117e+02 4.39175800078397e+02 &

8.14343169924012e+02 7.42957763671875e+02 4.40026044980605e+02 &

face id 2 &

8.15130661800511e+02 7.42957763671875e+02 4.37033689477478e+02 &

8.16975670140343e+02 7.39892790962574e+02 4.39960247842898e+02 &

8.14343169924012e+02 7.42957763671875e+02 4.40026044980605e+02

; block 309

poly group "10001" mat 1 con 1 &

face id 1 &

7.97293155916416e+02 7.35149565914216e+02 4.10938424197682e+02 &

7.96585601940516e+02 7.33011023940279e+02 4.10127244628184e+02 &

7.95275524466244e+02 7.35319480824925e+02 4.09720652974944e+02 &

face id 2 &

7.96585601940516e+02 7.33011023940279e+02 4.10127244628184e+02 &

7.97293155916416e+02 7.35149565914216e+02 4.10938424197682e+02 &

7.97589752627985e+02 7.34292202745755e+02 4.08481026952103e+02 &

face id 2 &

7.95275524466244e+02 7.35319480824925e+02 4.09720652974944e+02 &

7.96585601940516e+02 7.33011023940279e+02 4.10127244628184e+02 &

7.97589752627985e+02 7.34292202745755e+02 4.08481026952103e+02 &

face id 2 &

7.97293155916416e+02 7.35149565914216e+02 4.10938424197682e+02 &

7.95275524466244e+02 7.35319480824925e+02 4.09720652974944e+02 &

7.97589752627985e+02 7.34292202745755e+02 4.08481026952103e+02

; block 310

poly group "10001" mat 1 con 1 &

face id 2 &

8.04234314501538e+02 7.34635014532937e+02 4.05451928779116e+02 &

8.04590244899594e+02 7.31153198242188e+02 4.03296348788896e+02 &

8.02264872604041e+02 7.31153198242188e+02 4.05891525265379e+02 &

face id 2 &

8.04590244899594e+02 7.31153198242188e+02 4.03296348788896e+02 &

8.04234314501538e+02 7.34635014532937e+02 4.05451928779116e+02 &

8.00896162902359e+02 7.34934397043815e+02 4.04514414125901e+02 &

face id 2 &

8.02264872604041e+02 7.31153198242188e+02 4.05891525265379e+02 &

8.04590244899594e+02 7.31153198242188e+02 4.03296348788896e+02 &

8.00896162902359e+02 7.34934397043815e+02 4.04514414125901e+02 &

face id 2 &

8.04234314501538e+02 7.34635014532937e+02 4.05451928779116e+02 &

8.02264872604041e+02 7.31153198242188e+02 4.05891525265379e+02 &

8.00896162902359e+02 7.34934397043815e+02 4.04514414125901e+02

; block 311

poly group "10001" mat 1 con 1 &

face id 1 &

7.91099717380957e+02 7.31153198242188e+02 4.01060586132354e+02 &

7.92196673750300e+02 7.31153198242188e+02 3.98319581504036e+02 &

7.89669829571038e+02 7.31153198242188e+02 3.99434746165080e+02 &

face id 2 &

7.92196673750300e+02 7.31153198242188e+02 3.98319581504036e+02 &

7.91099717380957e+02 7.31153198242188e+02 4.01060586132354e+02 &

7.92440189486717e+02 7.33520249849107e+02 4.00528860955446e+02 &

face id 2 &

7.89669829571038e+02 7.31153198242188e+02 3.99434746165080e+02 &

7.92196673750300e+02 7.31153198242188e+02 3.98319581504036e+02 &

7.92440189486717e+02 7.33520249849107e+02 4.00528860955446e+02 &

face id 2 &

7.91099717380957e+02 7.31153198242188e+02 4.01060586132354e+02 &

7.89669829571038e+02 7.31153198242188e+02 3.99434746165080e+02 &

7.92440189486717e+02 7.33520249849107e+02 4.00528860955446e+02

; block 312

poly group "10001" mat 1 con 1 &

face id 1 &

7.92196673750300e+02 7.31153198242188e+02 3.98319581504036e+02 &

7.91099717380957e+02 7.31153198242188e+02 4.01060586132354e+02 &

7.93917417136445e+02 7.31153198242188e+02 4.00869604015908e+02 &

face id 2 &

7.91099717380957e+02 7.31153198242188e+02 4.01060586132354e+02 &

7.92196673750300e+02 7.31153198242188e+02 3.98319581504036e+02 &

7.92440189486717e+02 7.33520249849107e+02 4.00528860955446e+02 &

face id 2 &

7.93917417136445e+02 7.31153198242188e+02 4.00869604015908e+02 &

7.91099717380957e+02 7.31153198242188e+02 4.01060586132354e+02 &

7.92440189486717e+02 7.33520249849107e+02 4.00528860955446e+02 &

face id 2 &

7.92196673750300e+02 7.31153198242188e+02 3.98319581504036e+02 &

7.93917417136445e+02 7.31153198242188e+02 4.00869604015908e+02 &

7.92440189486717e+02 7.33520249849107e+02 4.00528860955446e+02

; block 313

poly group "10001" mat 1 con 1 &

face id 2 &

7.89220069374438e+02 7.39019972726502e+02 3.69753861949652e+02 &

7.82786128969092e+02 7.37151441981042e+02 3.68867086099698e+02 &

7.85546529336975e+02 7.37083937814405e+02 3.64848876953125e+02 &

face id 2 &

7.82786128969092e+02 7.37151441981042e+02 3.68867086099698e+02 &

7.89220069374438e+02 7.39019972726502e+02 3.69753861949652e+02 &

7.88153724348244e+02 7.34661142676503e+02 3.69505537208423e+02 &

face id 2 &

7.85546529336975e+02 7.37083937814405e+02 3.64848876953125e+02 &

7.82786128969092e+02 7.37151441981042e+02 3.68867086099698e+02 &

7.88153724348244e+02 7.34661142676503e+02 3.69505537208423e+02 &

face id 2 &

7.89220069374438e+02 7.39019972726502e+02 3.69753861949652e+02 &

7.85546529336975e+02 7.37083937814405e+02 3.64848876953125e+02 &

7.88153724348244e+02 7.34661142676503e+02 3.69505537208423e+02

; block 314

poly group "10001" mat 1 con 1 &

face id 2 &

7.97111944069104e+02 7.31153198242188e+02 3.90546888385563e+02 &

7.96478775035514e+02 7.34528043696007e+02 3.93001842890862e+02 &

8.00965458695504e+02 7.31153198242188e+02 3.91758115901608e+02 &

face id 2 &

7.96478775035514e+02 7.34528043696007e+02 3.93001842890862e+02 &

7.97111944069104e+02 7.31153198242188e+02 3.90546888385563e+02 &

7.99028431323575e+02 7.35618187258196e+02 3.90154438356032e+02 &

face id 2 &

8.00965458695504e+02 7.31153198242188e+02 3.91758115901608e+02 &

7.96478775035514e+02 7.34528043696007e+02 3.93001842890862e+02 &

7.99028431323575e+02 7.35618187258196e+02 3.90154438356032e+02 &

face id 2 &

7.97111944069104e+02 7.31153198242188e+02 3.90546888385563e+02 &

8.00965458695504e+02 7.31153198242188e+02 3.91758115901608e+02 &

7.99028431323575e+02 7.35618187258196e+02 3.90154438356032e+02

; block 315

poly group "10001" mat 1 con 1 &

face id 2 &

7.99724447295145e+02 7.42957763671875e+02 3.87022301448822e+02 &

7.97168838062648e+02 7.39187632994892e+02 3.86818051061183e+02 &

8.00154378440319e+02 7.42957763671875e+02 3.81345085869093e+02 &

face id 2 &

7.97168838062648e+02 7.39187632994892e+02 3.86818051061183e+02 &

7.99724447295145e+02 7.42957763671875e+02 3.87022301448822e+02 &

8.01551593806463e+02 7.37121430531446e+02 3.83226223248936e+02 &

face id 2 &

8.00154378440319e+02 7.42957763671875e+02 3.81345085869093e+02 &

7.97168838062648e+02 7.39187632994892e+02 3.86818051061183e+02 &

8.01551593806463e+02 7.37121430531446e+02 3.83226223248936e+02 &

face id 2 &

7.99724447295145e+02 7.42957763671875e+02 3.87022301448822e+02 &

8.00154378440319e+02 7.42957763671875e+02 3.81345085869093e+02 &

8.01551593806463e+02 7.37121430531446e+02 3.83226223248936e+02

; block 316

poly group "10001" mat 1 con 1 &

face id 2 &

7.96007174242569e+02 7.31153198242188e+02 3.86339457867366e+02 &

7.94608063469192e+02 7.35413562350436e+02 3.84880272566777e+02 &

7.96012403601172e+02 7.34733593543699e+02 3.88834189030604e+02 &

face id 2 &

7.94608063469192e+02 7.35413562350436e+02 3.84880272566777e+02 &

7.96007174242569e+02 7.31153198242188e+02 3.86339457867366e+02 &

7.98654543635136e+02 7.35451075167486e+02 3.85750035024265e+02 &

face id 2 &

7.96012403601172e+02 7.34733593543699e+02 3.88834189030604e+02 &

7.94608063469192e+02 7.35413562350436e+02 3.84880272566777e+02 &

7.98654543635136e+02 7.35451075167486e+02 3.85750035024265e+02 &

face id 2 &

7.96007174242569e+02 7.31153198242188e+02 3.86339457867366e+02 &

7.96012403601172e+02 7.34733593543699e+02 3.88834189030604e+02 &

7.98654543635136e+02 7.35451075167486e+02 3.85750035024265e+02

; block 317

poly group "10001" mat 1 con 1 &

face id 2 &

8.18920370898699e+02 7.37329641873752e+02 3.85333219479087e+02 &

8.14984905319849e+02 7.42957763671875e+02 3.89627546226137e+02 &

8.14017271454085e+02 7.42957763671875e+02 3.83339423702604e+02 &

face id 2 &

8.14984905319849e+02 7.42957763671875e+02 3.89627546226137e+02 &

8.18920370898699e+02 7.37329641873752e+02 3.85333219479087e+02 &

8.12290681094227e+02 7.36396429920157e+02 3.89417525587450e+02 &

face id 2 &

8.14017271454085e+02 7.42957763671875e+02 3.83339423702604e+02 &

8.14984905319849e+02 7.42957763671875e+02 3.89627546226137e+02 &

8.12290681094227e+02 7.36396429920157e+02 3.89417525587450e+02 &

face id 2 &

8.18920370898699e+02 7.37329641873752e+02 3.85333219479087e+02 &

8.14017271454085e+02 7.42957763671875e+02 3.83339423702604e+02 &

8.12290681094227e+02 7.36396429920157e+02 3.89417525587450e+02

; block 318

poly group "10001" mat 1 con 1 &

face id 2 &

8.22991116415336e+02 7.31153198242188e+02 3.85627454268575e+02 &

8.18920370898699e+02 7.37329641873752e+02 3.85333219479087e+02 &

8.16732385140107e+02 7.31153198242188e+02 3.89315202949226e+02 &

face id 2 &

8.18920370898699e+02 7.37329641873752e+02 3.85333219479087e+02 &

8.22991116415336e+02 7.31153198242188e+02 3.85627454268575e+02 &

8.18483672968706e+02 7.37593060216477e+02 3.91417863514613e+02 &

face id 2 &

8.16732385140107e+02 7.31153198242188e+02 3.89315202949226e+02 &

8.18920370898699e+02 7.37329641873752e+02 3.85333219479087e+02 &

8.18483672968706e+02 7.37593060216477e+02 3.91417863514613e+02 &

face id 2 &

8.22991116415336e+02 7.31153198242188e+02 3.85627454268575e+02 &

8.16732385140107e+02 7.31153198242188e+02 3.89315202949226e+02 &

8.18483672968706e+02 7.37593060216477e+02 3.91417863514613e+02

; block 319

poly group "10001" mat 1 con 1 &

face id 2 &

8.03297268032056e+02 7.35208989333878e+02 3.86866573172394e+02 &

8.00423884947867e+02 7.31153198242188e+02 3.87599039819564e+02 &

8.02809345752658e+02 7.35127916281908e+02 3.91487446480074e+02 &

face id 2 &

8.00423884947867e+02 7.31153198242188e+02 3.87599039819564e+02 &

8.03297268032056e+02 7.35208989333878e+02 3.86866573172394e+02 &

7.99028431323575e+02 7.35618187258196e+02 3.90154438356032e+02 &

face id 2 &

8.02809345752658e+02 7.35127916281908e+02 3.91487446480074e+02 &

8.00423884947867e+02 7.31153198242188e+02 3.87599039819564e+02 &

7.99028431323575e+02 7.35618187258196e+02 3.90154438356032e+02 &

face id 2 &

8.03297268032056e+02 7.35208989333878e+02 3.86866573172394e+02 &

8.02809345752658e+02 7.35127916281908e+02 3.91487446480074e+02 &

7.99028431323575e+02 7.35618187258196e+02 3.90154438356032e+02

; block 320

poly group "10001" mat 1 con 1 &

face id 2 &

7.97168838062648e+02 7.39187632994892e+02 3.86818051061183e+02 &

7.99724447295145e+02 7.42957763671875e+02 3.87022301448822e+02 &

7.96671058174266e+02 7.42957763671875e+02 3.90227925573037e+02 &

face id 2 &

7.99724447295145e+02 7.42957763671875e+02 3.87022301448822e+02 &

7.97168838062648e+02 7.39187632994892e+02 3.86818051061183e+02 &

8.01076203387915e+02 7.39174910359122e+02 3.89319688925391e+02 &

face id 2 &

7.96671058174266e+02 7.42957763671875e+02 3.90227925573037e+02 &

7.99724447295145e+02 7.42957763671875e+02 3.87022301448822e+02 &

8.01076203387915e+02 7.39174910359122e+02 3.89319688925391e+02 &

face id 2 &

7.97168838062648e+02 7.39187632994892e+02 3.86818051061183e+02 &

7.96671058174266e+02 7.42957763671875e+02 3.90227925573037e+02 &

8.01076203387915e+02 7.39174910359122e+02 3.89319688925391e+02

; block 321

poly group "10001" mat 1 con 1 &

face id 2 &

7.99156871754180e+02 7.42957763671875e+02 3.74726900763980e+02 &

7.98831708908744e+02 7.36125522704012e+02 3.77926771882722e+02 &

7.96250595319160e+02 7.36616698982022e+02 3.74074088190253e+02 &

face id 2 &

7.98831708908744e+02 7.36125522704012e+02 3.77926771882722e+02 &

7.99156871754180e+02 7.42957763671875e+02 3.74726900763980e+02 &

8.02547639341709e+02 7.36329323377224e+02 3.73878279205792e+02 &

face id 2 &

7.96250595319160e+02 7.36616698982022e+02 3.74074088190253e+02 &

7.98831708908744e+02 7.36125522704012e+02 3.77926771882722e+02 &

8.02547639341709e+02 7.36329323377224e+02 3.73878279205792e+02 &

face id 2 &

7.99156871754180e+02 7.42957763671875e+02 3.74726900763980e+02 &

7.96250595319160e+02 7.36616698982022e+02 3.74074088190253e+02 &

8.02547639341709e+02 7.36329323377224e+02 3.73878279205792e+02

; block 322

poly group "10001" mat 1 con 1 &

face id 2 &

8.14017271454085e+02 7.42957763671875e+02 3.83339423702604e+02 &

8.12290681094227e+02 7.36396429920157e+02 3.89417525587450e+02 &

8.07581738970324e+02 7.36655126437911e+02 3.84328905480290e+02 &

face id 2 &

8.12290681094227e+02 7.36396429920157e+02 3.89417525587450e+02 &

8.14017271454085e+02 7.42957763671875e+02 3.83339423702604e+02 &

8.16038173260348e+02 7.37794690779616e+02 3.79187736116119e+02 &

face id 2 &

8.07581738970324e+02 7.36655126437911e+02 3.84328905480290e+02 &

8.12290681094227e+02 7.36396429920157e+02 3.89417525587450e+02 &

8.16038173260348e+02 7.37794690779616e+02 3.79187736116119e+02 &

face id 2 &

8.14017271454085e+02 7.42957763671875e+02 3.83339423702604e+02 &

8.07581738970324e+02 7.36655126437911e+02 3.84328905480290e+02 &

8.16038173260348e+02 7.37794690779616e+02 3.79187736116119e+02

; block 323

poly group "10001" mat 1 con 1 &

face id 2 &

8.14984905319849e+02 7.42957763671875e+02 3.89627546226137e+02 &

8.10641773231557e+02 7.37725836349123e+02 3.93566404294409e+02 &

8.09805525478267e+02 7.42957763671875e+02 3.89445971649157e+02 &

face id 2 &

8.10641773231557e+02 7.37725836349123e+02 3.93566404294409e+02 &

8.14984905319849e+02 7.42957763671875e+02 3.89627546226137e+02 &

8.12290681094227e+02 7.36396429920157e+02 3.89417525587450e+02 &

face id 2 &

8.09805525478267e+02 7.42957763671875e+02 3.89445971649157e+02 &

8.10641773231557e+02 7.37725836349123e+02 3.93566404294409e+02 &

8.12290681094227e+02 7.36396429920157e+02 3.89417525587450e+02 &

face id 2 &

8.14984905319849e+02 7.42957763671875e+02 3.89627546226137e+02 &

8.09805525478267e+02 7.42957763671875e+02 3.89445971649157e+02 &

8.12290681094227e+02 7.36396429920157e+02 3.89417525587450e+02

; block 324

poly group "10001" mat 1 con 1 &

face id 2 &

7.94608063469192e+02 7.35413562350436e+02 3.84880272566777e+02 &

7.97168838062648e+02 7.39187632994892e+02 3.86818051061183e+02 &

7.96012403601172e+02 7.34733593543699e+02 3.88834189030604e+02 &

face id 2 &

7.97168838062648e+02 7.39187632994892e+02 3.86818051061183e+02 &

7.94608063469192e+02 7.35413562350436e+02 3.84880272566777e+02 &

7.98654543635136e+02 7.35451075167486e+02 3.85750035024265e+02 &

face id 2 &

7.96012403601172e+02 7.34733593543699e+02 3.88834189030604e+02 &

7.97168838062648e+02 7.39187632994892e+02 3.86818051061183e+02 &

7.98654543635136e+02 7.35451075167486e+02 3.85750035024265e+02 &

face id 2 &

7.94608063469192e+02 7.35413562350436e+02 3.84880272566777e+02 &

7.96012403601172e+02 7.34733593543699e+02 3.88834189030604e+02 &

7.98654543635136e+02 7.35451075167486e+02 3.85750035024265e+02

; block 325

poly group "10001" mat 1 con 1 &

face id 1 &

8.21044537549873e+02 7.31153198242188e+02 4.36848956154312e+02 &

8.23611633300781e+02 7.31153198242188e+02 4.38513122558594e+02 &

8.24135792527692e+02 7.31153198242188e+02 4.34857938641253e+02 &

face id 2 &

8.23611633300781e+02 7.31153198242188e+02 4.38513122558594e+02 &

8.21044537549873e+02 7.31153198242188e+02 4.36848956154312e+02 &

8.23363932724725e+02 7.33802420140527e+02 4.37276399935772e+02 &

face id 2 &

8.24135792527692e+02 7.31153198242188e+02 4.34857938641253e+02 &

8.23611633300781e+02 7.31153198242188e+02 4.38513122558594e+02 &

8.23363932724725e+02 7.33802420140527e+02 4.37276399935772e+02 &

face id 2 &

8.21044537549873e+02 7.31153198242188e+02 4.36848956154312e+02 &

8.24135792527692e+02 7.31153198242188e+02 4.34857938641253e+02 &

8.23363932724725e+02 7.33802420140527e+02 4.37276399935772e+02

; block 326

poly group "10001" mat 1 con 1 &

face id 2 &

8.26011586687483e+02 7.37674958461435e+02 4.37842021451426e+02 &

8.23877178682653e+02 7.39381023327622e+02 4.36180865465919e+02 &

8.26215102552087e+02 7.42957763671875e+02 4.40024896299389e+02 &

face id 2 &

8.23877178682653e+02 7.39381023327622e+02 4.36180865465919e+02 &

8.26011586687483e+02 7.37674958461435e+02 4.37842021451426e+02 &

8.26217746986868e+02 7.42957763671875e+02 4.35505283716008e+02 &

face id 2 &

8.26215102552087e+02 7.42957763671875e+02 4.40024896299389e+02 &

8.23877178682653e+02 7.39381023327622e+02 4.36180865465919e+02 &

8.26217746986868e+02 7.42957763671875e+02 4.35505283716008e+02 &

face id 2 &

8.26011586687483e+02 7.37674958461435e+02 4.37842021451426e+02 &

8.26215102552087e+02 7.42957763671875e+02 4.40024896299389e+02 &

8.26217746986868e+02 7.42957763671875e+02 4.35505283716008e+02

; block 327

poly group "10001" mat 1 con 1 &

face id 1 &

8.24066589355469e+02 7.33591491699219e+02 4.44044647216797e+02 &

8.21872915596893e+02 7.35455555334649e+02 4.44418293051948e+02 &

8.23015788477307e+02 7.36389502141782e+02 4.45123063667697e+02 &

face id 2 &

8.21872915596893e+02 7.35455555334649e+02 4.44418293051948e+02 &

8.24066589355469e+02 7.33591491699219e+02 4.44044647216797e+02 &

8.22822252675870e+02 7.36637749364954e+02 4.41131123199765e+02 &

face id 2 &

8.23015788477307e+02 7.36389502141782e+02 4.45123063667697e+02 &

8.21872915596893e+02 7.35455555334649e+02 4.44418293051948e+02 &

8.22822252675870e+02 7.36637749364954e+02 4.41131123199765e+02 &

face id 2 &

8.24066589355469e+02 7.33591491699219e+02 4.44044647216797e+02 &

8.23015788477307e+02 7.36389502141782e+02 4.45123063667697e+02 &

8.22822252675870e+02 7.36637749364954e+02 4.41131123199765e+02

; block 328

poly group "10001" mat 1 con 1 &

face id 2 &

7.73620174311311e+02 7.31153198242188e+02 3.75562823635389e+02 &

7.71135925292969e+02 7.33726108156615e+02 3.76246415882706e+02 &

7.72417671557342e+02 7.33094075356536e+02 3.77831986423786e+02 &

face id 2 &

7.71135925292969e+02 7.33726108156615e+02 3.76246415882706e+02 &

7.73620174311311e+02 7.31153198242188e+02 3.75562823635389e+02 &

7.74716057886906e+02 7.34040302468977e+02 3.74164033232456e+02 &

face id 2 &

7.72417671557342e+02 7.33094075356536e+02 3.77831986423786e+02 &

7.71135925292969e+02 7.33726108156615e+02 3.76246415882706e+02 &

7.74716057886906e+02 7.34040302468977e+02 3.74164033232456e+02 &

face id 2 &

7.73620174311311e+02 7.31153198242188e+02 3.75562823635389e+02 &

7.72417671557342e+02 7.33094075356536e+02 3.77831986423786e+02 &

7.74716057886906e+02 7.34040302468977e+02 3.74164033232456e+02

; block 329

poly group "10001" mat 1 con 1 &

face id 2 &

8.30279431984152e+02 7.42957763671875e+02 4.42330783680423e+02 &

8.27910339647761e+02 7.39308812863053e+02 4.44974237053554e+02 &

8.26215102552087e+02 7.42957763671875e+02 4.40024896299389e+02 &

face id 2 &

8.27910339647761e+02 7.39308812863053e+02 4.44974237053554e+02 &

8.30279431984152e+02 7.42957763671875e+02 4.42330783680423e+02 &

8.28516744472485e+02 7.39206897844698e+02 4.40026719289599e+02 &

face id 2 &

8.26215102552087e+02 7.42957763671875e+02 4.40024896299389e+02 &

8.27910339647761e+02 7.39308812863053e+02 4.44974237053554e+02 &

8.28516744472485e+02 7.39206897844698e+02 4.40026719289599e+02 &

face id 2 &

8.30279431984152e+02 7.42957763671875e+02 4.42330783680423e+02 &

8.26215102552087e+02 7.42957763671875e+02 4.40024896299389e+02 &

8.28516744472485e+02 7.39206897844698e+02 4.40026719289599e+02

; block 330

poly group "10001" mat 1 con 1 &

face id 2 &

8.22685164003876e+02 7.42957763671875e+02 4.34593170451076e+02 &

8.20813556445955e+02 7.40175884004747e+02 4.38506718959274e+02 &

8.19969183963055e+02 7.42957763671875e+02 4.36434014796289e+02 &

face id 2 &

8.20813556445955e+02 7.40175884004747e+02 4.38506718959274e+02 &

8.22685164003876e+02 7.42957763671875e+02 4.34593170451076e+02 &

8.19582997168244e+02 7.40188540403784e+02 4.34477675949180e+02 &

face id 2 &

8.19969183963055e+02 7.42957763671875e+02 4.36434014796289e+02 &

8.20813556445955e+02 7.40175884004747e+02 4.38506718959274e+02 &

8.19582997168244e+02 7.40188540403784e+02 4.34477675949180e+02 &

face id 2 &

8.22685164003876e+02 7.42957763671875e+02 4.34593170451076e+02 &

8.19969183963055e+02 7.42957763671875e+02 4.36434014796289e+02 &

8.19582997168244e+02 7.40188540403784e+02 4.34477675949180e+02

; block 331

poly group "10001" mat 1 con 1 &

face id 2 &

8.29507144346306e+02 7.31153198242188e+02 4.41289893556219e+02 &

8.26114508878027e+02 7.34878405664679e+02 4.40774789501596e+02 &

8.27040491723330e+02 7.31153198242188e+02 4.44660601575022e+02 &

face id 2 &

8.26114508878027e+02 7.34878405664679e+02 4.40774789501596e+02 &

8.29507144346306e+02 7.31153198242188e+02 4.41289893556219e+02 &

8.29251774542374e+02 7.34873958371039e+02 4.43856971841759e+02 &

face id 2 &

8.27040491723330e+02 7.31153198242188e+02 4.44660601575022e+02 &

8.26114508878027e+02 7.34878405664679e+02 4.40774789501596e+02 &

8.29251774542374e+02 7.34873958371039e+02 4.43856971841759e+02 &

face id 2 &

8.29507144346306e+02 7.31153198242188e+02 4.41289893556219e+02 &

8.27040491723330e+02 7.31153198242188e+02 4.44660601575022e+02 &

8.29251774542374e+02 7.34873958371039e+02 4.43856971841759e+02

; block 332

poly group "10001" mat 1 con 1 &

face id 1 &

8.21872915596893e+02 7.35455555334649e+02 4.44418293051948e+02 &

8.24066589355469e+02 7.33591491699219e+02 4.44044647216797e+02 &

8.23125000000000e+02 7.32601562500000e+02 4.41433044433594e+02 &

face id 2 &

8.24066589355469e+02 7.33591491699219e+02 4.44044647216797e+02 &

8.21872915596893e+02 7.35455555334649e+02 4.44418293051948e+02 &

8.22822252675870e+02 7.36637749364954e+02 4.41131123199765e+02 &

face id 2 &

8.23125000000000e+02 7.32601562500000e+02 4.41433044433594e+02 &

8.24066589355469e+02 7.33591491699219e+02 4.44044647216797e+02 &

8.22822252675870e+02 7.36637749364954e+02 4.41131123199765e+02 &

face id 2 &

8.21872915596893e+02 7.35455555334649e+02 4.44418293051948e+02 &

8.23125000000000e+02 7.32601562500000e+02 4.41433044433594e+02 &

8.22822252675870e+02 7.36637749364954e+02 4.41131123199765e+02

; block 333

poly group "10001" mat 1 con 1 &

face id 2 &

8.26210126105588e+02 7.35272944122540e+02 4.33492780179440e+02 &

8.23363932724725e+02 7.33802420140527e+02 4.37276399935772e+02 &

8.27899766625565e+02 7.31153198242188e+02 4.37953027515864e+02 &

face id 2 &

8.23363932724725e+02 7.33802420140527e+02 4.37276399935772e+02 &

8.26210126105588e+02 7.35272944122540e+02 4.33492780179440e+02 &

8.26011586687483e+02 7.37674958461435e+02 4.37842021451426e+02 &

face id 2 &

8.27899766625565e+02 7.31153198242188e+02 4.37953027515864e+02 &

8.23363932724725e+02 7.33802420140527e+02 4.37276399935772e+02 &

8.26011586687483e+02 7.37674958461435e+02 4.37842021451426e+02 &

face id 2 &

8.26210126105588e+02 7.35272944122540e+02 4.33492780179440e+02 &

8.27899766625565e+02 7.31153198242188e+02 4.37953027515864e+02 &

8.26011586687483e+02 7.37674958461435e+02 4.37842021451426e+02

; block 334

poly group "10001" mat 1 con 1 &

face id 1 &

8.26217746986868e+02 7.42957763671875e+02 4.35505283716008e+02 &

8.30336570924782e+02 7.42957763671875e+02 4.37949645698195e+02 &

8.26215102552087e+02 7.42957763671875e+02 4.40024896299389e+02 &

face id 2 &

8.30336570924782e+02 7.42957763671875e+02 4.37949645698195e+02 &

8.26217746986868e+02 7.42957763671875e+02 4.35505283716008e+02 &

8.28516744472485e+02 7.39206897844698e+02 4.40026719289599e+02 &

face id 2 &

8.26215102552087e+02 7.42957763671875e+02 4.40024896299389e+02 &

8.30336570924782e+02 7.42957763671875e+02 4.37949645698195e+02 &

8.28516744472485e+02 7.39206897844698e+02 4.40026719289599e+02 &

face id 2 &

8.26217746986868e+02 7.42957763671875e+02 4.35505283716008e+02 &

8.26215102552087e+02 7.42957763671875e+02 4.40024896299389e+02 &

8.28516744472485e+02 7.39206897844698e+02 4.40026719289599e+02

; block 335

poly group "10001" mat 1 con 1 &

face id 1 &

7.94535054290126e+02 7.33127082593229e+02 4.09095159059601e+02 &

7.93214784798896e+02 7.32908579213720e+02 4.07883259000688e+02 &

7.92794982910156e+02 7.35121520996094e+02 4.08285064697266e+02 &

face id 2 &

7.93214784798896e+02 7.32908579213720e+02 4.07883259000688e+02 &

7.94535054290126e+02 7.33127082593229e+02 4.09095159059601e+02 &

7.95446009423280e+02 7.33370401603083e+02 4.07686153733035e+02 &

face id 2 &

7.92794982910156e+02 7.35121520996094e+02 4.08285064697266e+02 &

7.93214784798896e+02 7.32908579213720e+02 4.07883259000688e+02 &

7.95446009423280e+02 7.33370401603083e+02 4.07686153733035e+02 &

face id 2 &

7.94535054290126e+02 7.33127082593229e+02 4.09095159059601e+02 &

7.92794982910156e+02 7.35121520996094e+02 4.08285064697266e+02 &

7.95446009423280e+02 7.33370401603083e+02 4.07686153733035e+02

; block 336

poly group "10001" mat 1 con 1 &

face id 1 &

7.71135925292969e+02 7.33726108156615e+02 3.76246415882706e+02 &

7.71135925292969e+02 7.35708841438567e+02 3.75363883785495e+02 &

7.72905778333058e+02 7.35543709744398e+02 3.77622196844083e+02 &

face id 2 &

7.71135925292969e+02 7.35708841438567e+02 3.75363883785495e+02 &

7.71135925292969e+02 7.33726108156615e+02 3.76246415882706e+02 &

7.74716057886906e+02 7.34040302468977e+02 3.74164033232456e+02 &

face id 2 &

7.72905778333058e+02 7.35543709744398e+02 3.77622196844083e+02 &

7.71135925292969e+02 7.35708841438567e+02 3.75363883785495e+02 &

7.74716057886906e+02 7.34040302468977e+02 3.74164033232456e+02 &

face id 2 &

7.71135925292969e+02 7.33726108156615e+02 3.76246415882706e+02 &

7.72905778333058e+02 7.35543709744398e+02 3.77622196844083e+02 &

7.74716057886906e+02 7.34040302468977e+02 3.74164033232456e+02

; block 337

poly group "10001" mat 1 con 1 &

face id 1 &

8.00586258626220e+02 7.31153198242188e+02 4.02680939650079e+02 &

8.04590244899594e+02 7.31153198242188e+02 4.03296348788896e+02 &

8.03279798752908e+02 7.31153198242188e+02 3.99668089001987e+02 &

face id 2 &

8.04590244899594e+02 7.31153198242188e+02 4.03296348788896e+02 &

8.00586258626220e+02 7.31153198242188e+02 4.02680939650079e+02 &

8.02767614436435e+02 7.34864934584089e+02 4.02023317503959e+02 &

face id 2 &

8.03279798752908e+02 7.31153198242188e+02 3.99668089001987e+02 &

8.04590244899594e+02 7.31153198242188e+02 4.03296348788896e+02 &

8.02767614436435e+02 7.34864934584089e+02 4.02023317503959e+02 &

face id 2 &

8.00586258626220e+02 7.31153198242188e+02 4.02680939650079e+02 &

8.03279798752908e+02 7.31153198242188e+02 3.99668089001987e+02 &

8.02767614436435e+02 7.34864934584089e+02 4.02023317503959e+02

; block 338

poly group "10001" mat 1 con 1 &

face id 2 &

8.00896162902359e+02 7.34934397043815e+02 4.04514414125901e+02 &

8.04590244899594e+02 7.31153198242188e+02 4.03296348788896e+02 &

8.00586258626220e+02 7.31153198242188e+02 4.02680939650079e+02 &

face id 2 &

8.04590244899594e+02 7.31153198242188e+02 4.03296348788896e+02 &

8.00896162902359e+02 7.34934397043815e+02 4.04514414125901e+02 &

8.02767614436435e+02 7.34864934584089e+02 4.02023317503959e+02 &

face id 2 &

8.00586258626220e+02 7.31153198242188e+02 4.02680939650079e+02 &

8.04590244899594e+02 7.31153198242188e+02 4.03296348788896e+02 &

8.02767614436435e+02 7.34864934584089e+02 4.02023317503959e+02 &

face id 2 &

8.00896162902359e+02 7.34934397043815e+02 4.04514414125901e+02 &

8.00586258626220e+02 7.31153198242188e+02 4.02680939650079e+02 &

8.02767614436435e+02 7.34864934584089e+02 4.02023317503959e+02

; block 339

poly group "10001" mat 1 con 1 &

face id 1 &

8.13323409378183e+02 7.31153198242188e+02 4.11395970865662e+02 &

8.19109440993736e+02 7.31153198242188e+02 4.10310739103682e+02 &

8.13668525515790e+02 7.31153198242188e+02 4.06737925704045e+02 &

face id 2 &

8.19109440993736e+02 7.31153198242188e+02 4.10310739103682e+02 &

8.13323409378183e+02 7.31153198242188e+02 4.11395970865662e+02 &

8.18044212102941e+02 7.37294444794664e+02 4.06947605911675e+02 &

face id 2 &

8.13668525515790e+02 7.31153198242188e+02 4.06737925704045e+02 &

8.19109440993736e+02 7.31153198242188e+02 4.10310739103682e+02 &

8.18044212102941e+02 7.37294444794664e+02 4.06947605911675e+02 &

face id 2 &

8.13323409378183e+02 7.31153198242188e+02 4.11395970865662e+02 &

8.13668525515790e+02 7.31153198242188e+02 4.06737925704045e+02 &

8.18044212102941e+02 7.37294444794664e+02 4.06947605911675e+02

; block 340

poly group "10001" mat 1 con 1 &

face id 2 &

8.20600098315628e+02 7.37493952138853e+02 3.97440050588930e+02 &

8.13648731082524e+02 7.37198409103348e+02 3.98003234961222e+02 &

8.13870124921521e+02 7.42957763671875e+02 3.95224545195568e+02 &

face id 2 &

8.13648731082524e+02 7.37198409103348e+02 3.98003234961222e+02 &

8.20600098315628e+02 7.37493952138853e+02 3.97440050588930e+02 &

8.18483672968706e+02 7.37593060216477e+02 3.91417863514613e+02 &

face id 2 &

8.13870124921521e+02 7.42957763671875e+02 3.95224545195568e+02 &

8.13648731082524e+02 7.37198409103348e+02 3.98003234961222e+02 &

8.18483672968706e+02 7.37593060216477e+02 3.91417863514613e+02 &

face id 2 &

8.20600098315628e+02 7.37493952138853e+02 3.97440050588930e+02 &

8.13870124921521e+02 7.42957763671875e+02 3.95224545195568e+02 &

8.18483672968706e+02 7.37593060216477e+02 3.91417863514613e+02

; block 341

poly group "10001" mat 1 con 1 &

face id 2 &

8.00896600562332e+02 7.42957763671875e+02 3.91322005726277e+02 &

7.96671058174266e+02 7.42957763671875e+02 3.90227925573037e+02 &

8.01076203387915e+02 7.39174910359122e+02 3.89319688925391e+02 &

face id 2 &

7.96671058174266e+02 7.42957763671875e+02 3.90227925573037e+02 &

8.00896600562332e+02 7.42957763671875e+02 3.91322005726277e+02 &

7.95765195392309e+02 7.39396095350468e+02 3.92331740066270e+02 &

face id 2 &

8.01076203387915e+02 7.39174910359122e+02 3.89319688925391e+02 &

7.96671058174266e+02 7.42957763671875e+02 3.90227925573037e+02 &

7.95765195392309e+02 7.39396095350468e+02 3.92331740066270e+02 &

face id 2 &

8.00896600562332e+02 7.42957763671875e+02 3.91322005726277e+02 &

8.01076203387915e+02 7.39174910359122e+02 3.89319688925391e+02 &

7.95765195392309e+02 7.39396095350468e+02 3.92331740066270e+02

; block 342

poly group "10001" mat 1 con 1 &

face id 2 &

8.05166630720558e+02 7.39260084743635e+02 4.09062297464921e+02 &

8.04748042289060e+02 7.34593085936688e+02 4.08934836753061e+02 &

8.03509241519356e+02 7.36685442706800e+02 4.11505637635243e+02 &

face id 2 &

8.04748042289060e+02 7.34593085936688e+02 4.08934836753061e+02 &

8.05166630720558e+02 7.39260084743635e+02 4.09062297464921e+02 &

8.03287461742746e+02 7.37038107997743e+02 4.07568950280680e+02 &

face id 2 &

8.03509241519356e+02 7.36685442706800e+02 4.11505637635243e+02 &

8.04748042289060e+02 7.34593085936688e+02 4.08934836753061e+02 &

8.03287461742746e+02 7.37038107997743e+02 4.07568950280680e+02 &

face id 2 &

8.05166630720558e+02 7.39260084743635e+02 4.09062297464921e+02 &

8.03509241519356e+02 7.36685442706800e+02 4.11505637635243e+02 &

8.03287461742746e+02 7.37038107997743e+02 4.07568950280680e+02

; block 343

poly group "10001" mat 1 con 1 &

face id 2 &

8.15108642578125e+02 7.32791687011719e+02 4.38261230468750e+02 &

8.17662933994469e+02 7.34542125234693e+02 4.38288677337318e+02 &

8.15793233645232e+02 7.34747728264843e+02 4.35883786023879e+02 &

face id 2 &

8.17662933994469e+02 7.34542125234693e+02 4.38288677337318e+02 &

8.15108642578125e+02 7.32791687011719e+02 4.38261230468750e+02 &

8.16269421594957e+02 7.36054575659189e+02 4.39716662360012e+02 &

face id 2 &

8.15793233645232e+02 7.34747728264843e+02 4.35883786023879e+02 &

8.17662933994469e+02 7.34542125234693e+02 4.38288677337318e+02 &

8.16269421594957e+02 7.36054575659189e+02 4.39716662360012e+02 &

face id 2 &

8.15108642578125e+02 7.32791687011719e+02 4.38261230468750e+02 &

8.15793233645232e+02 7.34747728264843e+02 4.35883786023879e+02 &

8.16269421594957e+02 7.36054575659189e+02 4.39716662360012e+02

; block 344

poly group "10001" mat 1 con 1 &

face id 2 &

8.02747033303860e+02 7.31153198242188e+02 4.09084254148686e+02 &

8.01038761361681e+02 7.33990064792097e+02 4.10041158352416e+02 &

8.02808956378546e+02 7.31153198242188e+02 4.12218040733640e+02 &

face id 2 &

8.01038761361681e+02 7.33990064792097e+02 4.10041158352416e+02 &

8.02747033303860e+02 7.31153198242188e+02 4.09084254148686e+02 &

8.04465957264557e+02 7.33969583985651e+02 4.12069612436356e+02 &

face id 2 &

8.02808956378546e+02 7.31153198242188e+02 4.12218040733640e+02 &

8.01038761361681e+02 7.33990064792097e+02 4.10041158352416e+02 &

8.04465957264557e+02 7.33969583985651e+02 4.12069612436356e+02 &

face id 2 &

8.02747033303860e+02 7.31153198242188e+02 4.09084254148686e+02 &

8.02808956378546e+02 7.31153198242188e+02 4.12218040733640e+02 &

8.04465957264557e+02 7.33969583985651e+02 4.12069612436356e+02

; block 345

poly group "10001" mat 1 con 1 &

face id 2 &

7.91329608055828e+02 7.31153198242188e+02 3.95119467848931e+02 &

7.93143690467033e+02 7.34153895053626e+02 3.94736213202014e+02 &

7.90665610154540e+02 7.31153198242188e+02 3.92006573131752e+02 &

face id 2 &

7.93143690467033e+02 7.34153895053626e+02 3.94736213202014e+02 &

7.91329608055828e+02 7.31153198242188e+02 3.95119467848931e+02 &

7.90048611720629e+02 7.34003260059407e+02 3.93926493742337e+02 &

face id 2 &

7.90665610154540e+02 7.31153198242188e+02 3.92006573131752e+02 &

7.93143690467033e+02 7.34153895053626e+02 3.94736213202014e+02 &

7.90048611720629e+02 7.34003260059407e+02 3.93926493742337e+02 &

face id 2 &

7.91329608055828e+02 7.31153198242188e+02 3.95119467848931e+02 &

7.90665610154540e+02 7.31153198242188e+02 3.92006573131752e+02 &

7.90048611720629e+02 7.34003260059407e+02 3.93926493742337e+02

; block 346

poly group "10001" mat 1 con 1 &

face id 2 &

7.93269602462016e+02 7.42957763671875e+02 3.93091351036508e+02 &

7.96021030652622e+02 7.39527701437256e+02 3.96689394205240e+02 &

7.93739440525348e+02 7.42957763671875e+02 3.96852948504114e+02 &

face id 2 &

7.96021030652622e+02 7.39527701437256e+02 3.96689394205240e+02 &

7.93269602462016e+02 7.42957763671875e+02 3.93091351036508e+02 &

7.92078395752426e+02 7.40216819168812e+02 3.95906231480863e+02 &

face id 2 &

7.93739440525348e+02 7.42957763671875e+02 3.96852948504114e+02 &

7.96021030652622e+02 7.39527701437256e+02 3.96689394205240e+02 &

7.92078395752426e+02 7.40216819168812e+02 3.95906231480863e+02 &

face id 2 &

7.93269602462016e+02 7.42957763671875e+02 3.93091351036508e+02 &

7.93739440525348e+02 7.42957763671875e+02 3.96852948504114e+02 &

7.92078395752426e+02 7.40216819168812e+02 3.95906231480863e+02

; block 347

poly group "10001" mat 1 con 1 &

face id 1 &

7.88118627782222e+02 7.40092607595057e+02 3.90321821153071e+02 &

7.89040717114038e+02 7.42957763671875e+02 3.87735495760401e+02 &

7.89677653079890e+02 7.42957763671875e+02 3.91613324171349e+02 &

face id 2 &

7.89040717114038e+02 7.42957763671875e+02 3.87735495760401e+02 &

7.88118627782222e+02 7.40092607595057e+02 3.90321821153071e+02 &

7.91117097900182e+02 7.39426485780028e+02 3.91447399439320e+02 &

face id 2 &

7.89677653079890e+02 7.42957763671875e+02 3.91613324171349e+02 &

7.89040717114038e+02 7.42957763671875e+02 3.87735495760401e+02 &

7.91117097900182e+02 7.39426485780028e+02 3.91447399439320e+02 &

face id 2 &

7.88118627782222e+02 7.40092607595057e+02 3.90321821153071e+02 &

7.89677653079890e+02 7.42957763671875e+02 3.91613324171349e+02 &

7.91117097900182e+02 7.39426485780028e+02 3.91447399439320e+02

; block 348

poly group "10001" mat 1 con 1 &

face id 1 &

8.12305419921875e+02 7.39440551757812e+02 4.40937561035156e+02 &

8.13033386230469e+02 7.37296997070312e+02 4.41984466552734e+02 &

8.12198316771942e+02 7.36419651568513e+02 4.39675799850027e+02 &

face id 2 &

8.13033386230469e+02 7.37296997070312e+02 4.41984466552734e+02 &

8.12305419921875e+02 7.39440551757812e+02 4.40937561035156e+02 &

8.14838076915517e+02 7.39071150525117e+02 4.39175800078397e+02 &

face id 2 &

8.12198316771942e+02 7.36419651568513e+02 4.39675799850027e+02 &

8.13033386230469e+02 7.37296997070312e+02 4.41984466552734e+02 &

8.14838076915517e+02 7.39071150525117e+02 4.39175800078397e+02 &

face id 2 &

8.12305419921875e+02 7.39440551757812e+02 4.40937561035156e+02 &

8.12198316771942e+02 7.36419651568513e+02 4.39675799850027e+02 &

8.14838076915517e+02 7.39071150525117e+02 4.39175800078397e+02

; block 349

poly group "10001" mat 1 con 1 &

face id 2 &

7.95765195392309e+02 7.39396095350468e+02 3.92331740066270e+02 &

7.96021030652622e+02 7.39527701437256e+02 3.96689394205240e+02 &

7.93269602462016e+02 7.42957763671875e+02 3.93091351036508e+02 &

face id 2 &

7.96021030652622e+02 7.39527701437256e+02 3.96689394205240e+02 &

7.95765195392309e+02 7.39396095350468e+02 3.92331740066270e+02 &

7.92078395752426e+02 7.40216819168812e+02 3.95906231480863e+02 &

face id 2 &

7.93269602462016e+02 7.42957763671875e+02 3.93091351036508e+02 &

7.96021030652622e+02 7.39527701437256e+02 3.96689394205240e+02 &

7.92078395752426e+02 7.40216819168812e+02 3.95906231480863e+02 &

face id 2 &

7.95765195392309e+02 7.39396095350468e+02 3.92331740066270e+02 &

7.93269602462016e+02 7.42957763671875e+02 3.93091351036508e+02 &

7.92078395752426e+02 7.40216819168812e+02 3.95906231480863e+02

; block 350

poly group "10001" mat 1 con 1 &

face id 1 &

7.88840026952497e+02 7.40307337647071e+02 3.93803242777784e+02 &

7.89373224229090e+02 7.40320850286657e+02 3.97270117705758e+02 &

7.88326407845862e+02 7.38079130393022e+02 3.96095971328112e+02 &

face id 2 &

7.89373224229090e+02 7.40320850286657e+02 3.97270117705758e+02 &

7.88840026952497e+02 7.40307337647071e+02 3.93803242777784e+02 &

7.92078395752426e+02 7.40216819168812e+02 3.95906231480863e+02 &

face id 2 &

7.88326407845862e+02 7.38079130393022e+02 3.96095971328112e+02 &

7.89373224229090e+02 7.40320850286657e+02 3.97270117705758e+02 &

7.92078395752426e+02 7.40216819168812e+02 3.95906231480863e+02 &

face id 2 &

7.88840026952497e+02 7.40307337647071e+02 3.93803242777784e+02 &

7.88326407845862e+02 7.38079130393022e+02 3.96095971328112e+02 &

7.92078395752426e+02 7.40216819168812e+02 3.95906231480863e+02

; block 351

poly group "10001" mat 1 con 1 &

face id 1 &

7.89373224229090e+02 7.40320850286657e+02 3.97270117705758e+02 &

7.88920547739239e+02 7.37839599356893e+02 3.99365105432018e+02 &

7.88326407845862e+02 7.38079130393022e+02 3.96095971328112e+02 &

face id 2 &

7.88920547739239e+02 7.37839599356893e+02 3.99365105432018e+02 &

7.89373224229090e+02 7.40320850286657e+02 3.97270117705758e+02 &

7.91408350694546e+02 7.37046149302478e+02 3.97065808643254e+02 &

face id 2 &

7.88326407845862e+02 7.38079130393022e+02 3.96095971328112e+02 &

7.88920547739239e+02 7.37839599356893e+02 3.99365105432018e+02 &

7.91408350694546e+02 7.37046149302478e+02 3.97065808643254e+02 &

face id 2 &

7.89373224229090e+02 7.40320850286657e+02 3.97270117705758e+02 &

7.88326407845862e+02 7.38079130393022e+02 3.96095971328112e+02 &

7.91408350694546e+02 7.37046149302478e+02 3.97065808643254e+02

; block 352

poly group "10001" mat 1 con 1 &

face id 1 &

7.99522611055049e+02 7.42957763671875e+02 4.05500640780260e+02 &

7.98543325683381e+02 7.42957763671875e+02 4.09357360801871e+02 &

7.95969494748092e+02 7.42957763671875e+02 4.07148050498129e+02 &

face id 2 &

7.98543325683381e+02 7.42957763671875e+02 4.09357360801871e+02 &

7.99522611055049e+02 7.42957763671875e+02 4.05500640780260e+02 &

7.99326131256449e+02 7.40590042422744e+02 4.07820655966596e+02 &

face id 2 &

7.95969494748092e+02 7.42957763671875e+02 4.07148050498129e+02 &

7.98543325683381e+02 7.42957763671875e+02 4.09357360801871e+02 &

7.99326131256449e+02 7.40590042422744e+02 4.07820655966596e+02 &

face id 2 &

7.99522611055049e+02 7.42957763671875e+02 4.05500640780260e+02 &

7.95969494748092e+02 7.42957763671875e+02 4.07148050498129e+02 &

7.99326131256449e+02 7.40590042422744e+02 4.07820655966596e+02

; block 353

poly group "10001" mat 1 con 1 &

face id 2 &

7.95765195392309e+02 7.39396095350468e+02 3.92331740066270e+02 &

7.92078395752426e+02 7.40216819168812e+02 3.95906231480863e+02 &

7.93269602462016e+02 7.42957763671875e+02 3.93091351036508e+02 &

face id 2 &

7.92078395752426e+02 7.40216819168812e+02 3.95906231480863e+02 &

7.95765195392309e+02 7.39396095350468e+02 3.92331740066270e+02 &

7.91117097900182e+02 7.39426485780028e+02 3.91447399439320e+02 &

face id 2 &

7.93269602462016e+02 7.42957763671875e+02 3.93091351036508e+02 &

7.92078395752426e+02 7.40216819168812e+02 3.95906231480863e+02 &

7.91117097900182e+02 7.39426485780028e+02 3.91447399439320e+02 &

face id 2 &

7.95765195392309e+02 7.39396095350468e+02 3.92331740066270e+02 &

7.93269602462016e+02 7.42957763671875e+02 3.93091351036508e+02 &

7.91117097900182e+02 7.39426485780028e+02 3.91447399439320e+02

; block 354

poly group "10001" mat 1 con 1 &

face id 2 &

7.93269602462016e+02 7.42957763671875e+02 3.93091351036508e+02 &

7.92078395752426e+02 7.40216819168812e+02 3.95906231480863e+02 &

7.88840026952497e+02 7.40307337647071e+02 3.93803242777784e+02 &

face id 2 &

7.92078395752426e+02 7.40216819168812e+02 3.95906231480863e+02 &

7.93269602462016e+02 7.42957763671875e+02 3.93091351036508e+02 &

7.91117097900182e+02 7.39426485780028e+02 3.91447399439320e+02 &

face id 2 &

7.88840026952497e+02 7.40307337647071e+02 3.93803242777784e+02 &

7.92078395752426e+02 7.40216819168812e+02 3.95906231480863e+02 &

7.91117097900182e+02 7.39426485780028e+02 3.91447399439320e+02 &

face id 2 &

7.93269602462016e+02 7.42957763671875e+02 3.93091351036508e+02 &

7.88840026952497e+02 7.40307337647071e+02 3.93803242777784e+02 &

7.91117097900182e+02 7.39426485780028e+02 3.91447399439320e+02

; block 355

poly group "10001" mat 1 con 1 &

face id 2 &

7.88326407845862e+02 7.38079130393022e+02 3.96095971328112e+02 &

7.92078395752426e+02 7.40216819168812e+02 3.95906231480863e+02 &

7.89373224229090e+02 7.40320850286657e+02 3.97270117705758e+02 &

face id 2 &

7.92078395752426e+02 7.40216819168812e+02 3.95906231480863e+02 &

7.88326407845862e+02 7.38079130393022e+02 3.96095971328112e+02 &

7.91408350694546e+02 7.37046149302478e+02 3.97065808643254e+02 &

face id 2 &

7.89373224229090e+02 7.40320850286657e+02 3.97270117705758e+02 &

7.92078395752426e+02 7.40216819168812e+02 3.95906231480863e+02 &

7.91408350694546e+02 7.37046149302478e+02 3.97065808643254e+02 &

face id 2 &

7.88326407845862e+02 7.38079130393022e+02 3.96095971328112e+02 &

7.89373224229090e+02 7.40320850286657e+02 3.97270117705758e+02 &

7.91408350694546e+02 7.37046149302478e+02 3.97065808643254e+02

; block 356

poly group "10001" mat 1 con 1 &

face id 2 &

8.39084289550781e+02 7.31153198242188e+02 4.35338289521477e+02 &

8.33841753250661e+02 7.31153198242188e+02 4.32006745170201e+02 &

8.33190865039410e+02 7.35305529052256e+02 4.34142847499269e+02 &

face id 2 &

8.33841753250661e+02 7.31153198242188e+02 4.32006745170201e+02 &

8.39084289550781e+02 7.31153198242188e+02 4.35338289521477e+02 &

8.39084289550781e+02 7.37015214063697e+02 4.30185524731766e+02 &

face id 2 &

8.33190865039410e+02 7.35305529052256e+02 4.34142847499269e+02 &

8.33841753250661e+02 7.31153198242188e+02 4.32006745170201e+02 &

8.39084289550781e+02 7.37015214063697e+02 4.30185524731766e+02 &

face id 2 &

8.39084289550781e+02 7.31153198242188e+02 4.35338289521477e+02 &

8.33190865039410e+02 7.35305529052256e+02 4.34142847499269e+02 &

8.39084289550781e+02 7.37015214063697e+02 4.30185524731766e+02

; block 357

poly group "10001" mat 1 con 1 &

face id 2 &

7.95632045078570e+02 7.39829233879320e+02 4.01197287506287e+02 &

7.92491426636271e+02 7.36717278666031e+02 4.00346859894982e+02 &

7.92609359140741e+02 7.39887378961859e+02 3.99568383832409e+02 &

face id 2 &

7.92491426636271e+02 7.36717278666031e+02 4.00346859894982e+02 &

7.95632045078570e+02 7.39829233879320e+02 4.01197287506287e+02 &

7.94943182185095e+02 7.36813761564512e+02 3.98851536379301e+02 &

face id 2 &

7.92609359140741e+02 7.39887378961859e+02 3.99568383832409e+02 &

7.92491426636271e+02 7.36717278666031e+02 4.00346859894982e+02 &

7.94943182185095e+02 7.36813761564512e+02 3.98851536379301e+02 &

face id 2 &

7.95632045078570e+02 7.39829233879320e+02 4.01197287506287e+02 &

7.92609359140741e+02 7.39887378961859e+02 3.99568383832409e+02 &

7.94943182185095e+02 7.36813761564512e+02 3.98851536379301e+02

; block 358

poly group "10001" mat 1 con 1 &

face id 2 &

7.92609359140741e+02 7.39887378961859e+02 3.99568383832409e+02 &

7.92078395752426e+02 7.40216819168812e+02 3.95906231480863e+02 &

7.96021030652622e+02 7.39527701437256e+02 3.96689394205240e+02 &

face id 2 &

7.92078395752426e+02 7.40216819168812e+02 3.95906231480863e+02 &

7.92609359140741e+02 7.39887378961859e+02 3.99568383832409e+02 &

7.91408350694546e+02 7.37046149302478e+02 3.97065808643254e+02 &

face id 2 &

7.96021030652622e+02 7.39527701437256e+02 3.96689394205240e+02 &

7.92078395752426e+02 7.40216819168812e+02 3.95906231480863e+02 &

7.91408350694546e+02 7.37046149302478e+02 3.97065808643254e+02 &

face id 2 &

7.92609359140741e+02 7.39887378961859e+02 3.99568383832409e+02 &

7.96021030652622e+02 7.39527701437256e+02 3.96689394205240e+02 &

7.91408350694546e+02 7.37046149302478e+02 3.97065808643254e+02

; block 359

poly group "10001" mat 1 con 1 &

face id 1 &

8.02959053430883e+02 7.42957763671875e+02 3.70313836990870e+02 &

8.05448465309216e+02 7.42957763671875e+02 3.76149659904077e+02 &

7.99156871754180e+02 7.42957763671875e+02 3.74726900763980e+02 &

face id 2 &

8.05448465309216e+02 7.42957763671875e+02 3.76149659904077e+02 &

8.02959053430883e+02 7.42957763671875e+02 3.70313836990870e+02 &

8.02547639341709e+02 7.36329323377224e+02 3.73878279205792e+02 &

face id 2 &

7.99156871754180e+02 7.42957763671875e+02 3.74726900763980e+02 &

8.05448465309216e+02 7.42957763671875e+02 3.76149659904077e+02 &

8.02547639341709e+02 7.36329323377224e+02 3.73878279205792e+02 &

face id 2 &

8.02959053430883e+02 7.42957763671875e+02 3.70313836990870e+02 &

7.99156871754180e+02 7.42957763671875e+02 3.74726900763980e+02 &

8.02547639341709e+02 7.36329323377224e+02 3.73878279205792e+02

; block 360

poly group "10001" mat 1 con 1 &

face id 2 &

7.71135925292969e+02 7.42957763671875e+02 3.67974465136804e+02 &

7.73641497426250e+02 7.42957763671875e+02 3.67323224537366e+02 &

7.74035965173978e+02 7.40428059095133e+02 3.67725881874126e+02 &

face id 1 &

7.73641497426250e+02 7.42957763671875e+02 3.67323224537366e+02 &

7.71135925292969e+02 7.42957763671875e+02 3.67974465136804e+02 &

7.71135925292969e+02 7.42957763671875e+02 3.64848876953125e+02 &

face id 2 &

7.74035965173978e+02 7.40428059095133e+02 3.67725881874126e+02 &

7.73641497426250e+02 7.42957763671875e+02 3.67323224537366e+02 &

7.71135925292969e+02 7.42957763671875e+02 3.64848876953125e+02 &

face id 2 &

7.71135925292969e+02 7.42957763671875e+02 3.67974465136804e+02 &

7.74035965173978e+02 7.40428059095133e+02 3.67725881874126e+02 &

7.71135925292969e+02 7.42957763671875e+02 3.64848876953125e+02

; block 361

poly group "10001" mat 1 con 1 &

face id 1 &

7.95969494748092e+02 7.42957763671875e+02 4.07148050498129e+02 &

7.93905372512047e+02 7.40465181717938e+02 4.06970757485805e+02 &

7.93561308374798e+02 7.42957763671875e+02 4.04816605543573e+02 &

face id 2 &

7.93905372512047e+02 7.40465181717938e+02 4.06970757485805e+02 &

7.95969494748092e+02 7.42957763671875e+02 4.07148050498129e+02 &

7.95504903662561e+02 7.39874254256717e+02 4.05138948699233e+02 &

face id 2 &

7.93561308374798e+02 7.42957763671875e+02 4.04816605543573e+02 &

7.93905372512047e+02 7.40465181717938e+02 4.06970757485805e+02 &

7.95504903662561e+02 7.39874254256717e+02 4.05138948699233e+02 &

face id 2 &

7.95969494748092e+02 7.42957763671875e+02 4.07148050498129e+02 &

7.93561308374798e+02 7.42957763671875e+02 4.04816605543573e+02 &

7.95504903662561e+02 7.39874254256717e+02 4.05138948699233e+02

; block 362

poly group "10001" mat 1 con 1 &

face id 1 &

7.81919545568227e+02 7.31153198242188e+02 3.77708803974256e+02 &

7.79046047912539e+02 7.31153198242188e+02 3.75832298195535e+02 &

7.78389795522681e+02 7.31153198242188e+02 3.79403540956464e+02 &

face id 2 &

7.79046047912539e+02 7.31153198242188e+02 3.75832298195535e+02 &

7.81919545568227e+02 7.31153198242188e+02 3.77708803974256e+02 &

7.79511950549799e+02 7.34457282336568e+02 3.78709527306644e+02 &

face id 2 &

7.78389795522681e+02 7.31153198242188e+02 3.79403540956464e+02 &

7.79046047912539e+02 7.31153198242188e+02 3.75832298195535e+02 &

7.79511950549799e+02 7.34457282336568e+02 3.78709527306644e+02 &

face id 2 &

7.81919545568227e+02 7.31153198242188e+02 3.77708803974256e+02 &

7.78389795522681e+02 7.31153198242188e+02 3.79403540956464e+02 &

7.79511950549799e+02 7.34457282336568e+02 3.78709527306644e+02

; block 363

poly group "10001" mat 1 con 1 &

face id 1 &

7.93905372512047e+02 7.40465181717938e+02 4.06970757485805e+02 &

7.94434081357035e+02 7.37853681186436e+02 4.08663893359867e+02 &

7.92588269424653e+02 7.38082674352109e+02 4.07029758855016e+02 &

face id 2 &

7.94434081357035e+02 7.37853681186436e+02 4.08663893359867e+02 &

7.93905372512047e+02 7.40465181717938e+02 4.06970757485805e+02 &

7.95504903662561e+02 7.39874254256717e+02 4.05138948699233e+02 &

face id 2 &

7.92588269424653e+02 7.38082674352109e+02 4.07029758855016e+02 &

7.94434081357035e+02 7.37853681186436e+02 4.08663893359867e+02 &

7.95504903662561e+02 7.39874254256717e+02 4.05138948699233e+02 &

face id 2 &

7.93905372512047e+02 7.40465181717938e+02 4.06970757485805e+02 &

7.92588269424653e+02 7.38082674352109e+02 4.07029758855016e+02 &

7.95504903662561e+02 7.39874254256717e+02 4.05138948699233e+02

; block 364

poly group "10001" mat 1 con 1 &

face id 2 &

7.97602694134558e+02 7.31153198242188e+02 4.04311629734455e+02 &

8.00896162902359e+02 7.34934397043815e+02 4.04514414125901e+02 &

7.96518989330910e+02 7.34092206745266e+02 4.03418560921382e+02 &

face id 2 &

8.00896162902359e+02 7.34934397043815e+02 4.04514414125901e+02 &

7.97602694134558e+02 7.31153198242188e+02 4.04311629734455e+02 &

7.97326795645079e+02 7.33484291964610e+02 4.05886842936342e+02 &

face id 2 &

7.96518989330910e+02 7.34092206745266e+02 4.03418560921382e+02 &

8.00896162902359e+02 7.34934397043815e+02 4.04514414125901e+02 &

7.97326795645079e+02 7.33484291964610e+02 4.05886842936342e+02 &

face id 2 &

7.97602694134558e+02 7.31153198242188e+02 4.04311629734455e+02 &

7.96518989330910e+02 7.34092206745266e+02 4.03418560921382e+02 &

7.97326795645079e+02 7.33484291964610e+02 4.05886842936342e+02

; block 365

poly group "10001" mat 1 con 1 &

face id 2 &

8.00586258626220e+02 7.31153198242188e+02 4.02680939650079e+02 &

8.02767614436435e+02 7.34864934584089e+02 4.02023317503959e+02 &

8.03279798752908e+02 7.31153198242188e+02 3.99668089001987e+02 &

face id 2 &

8.02767614436435e+02 7.34864934584089e+02 4.02023317503959e+02 &

8.00586258626220e+02 7.31153198242188e+02 4.02680939650079e+02 &

7.99432805907969e+02 7.34735523444890e+02 4.01271303355683e+02 &

face id 2 &

8.03279798752908e+02 7.31153198242188e+02 3.99668089001987e+02 &

8.02767614436435e+02 7.34864934584089e+02 4.02023317503959e+02 &

7.99432805907969e+02 7.34735523444890e+02 4.01271303355683e+02 &

face id 2 &

8.00586258626220e+02 7.31153198242188e+02 4.02680939650079e+02 &

8.03279798752908e+02 7.31153198242188e+02 3.99668089001987e+02 &

7.99432805907969e+02 7.34735523444890e+02 4.01271303355683e+02

; block 366

poly group "10001" mat 1 con 1 &

face id 2 &

7.93177635869142e+02 7.36964441637668e+02 4.03028389020813e+02 &

7.95504903662561e+02 7.39874254256717e+02 4.05138948699233e+02 &

7.92588269424653e+02 7.38082674352109e+02 4.07029758855016e+02 &

face id 2 &

7.95504903662561e+02 7.39874254256717e+02 4.05138948699233e+02 &

7.93177635869142e+02 7.36964441637668e+02 4.03028389020813e+02 &

7.94502659315746e+02 7.35572496129421e+02 4.06057284817908e+02 &

face id 2 &

7.92588269424653e+02 7.38082674352109e+02 4.07029758855016e+02 &

7.95504903662561e+02 7.39874254256717e+02 4.05138948699233e+02 &

7.94502659315746e+02 7.35572496129421e+02 4.06057284817908e+02 &

face id 2 &

7.93177635869142e+02 7.36964441637668e+02 4.03028389020813e+02 &

7.92588269424653e+02 7.38082674352109e+02 4.07029758855016e+02 &

7.94502659315746e+02 7.35572496129421e+02 4.06057284817908e+02

; block 367

poly group "10001" mat 1 con 1 &

face id 1 &

7.99360324142697e+02 7.31153198242188e+02 3.98847639366796e+02 &

7.97607558832901e+02 7.31153198242188e+02 3.95040058596455e+02 &

7.95760540850189e+02 7.31153198242187e+02 3.98481504684038e+02 &

face id 2 &

7.97607558832901e+02 7.31153198242188e+02 3.95040058596455e+02 &

7.99360324142697e+02 7.31153198242188e+02 3.98847639366796e+02 &

7.97516497216007e+02 7.34563943186781e+02 3.97383221855559e+02 &

face id 2 &

7.95760540850189e+02 7.31153198242187e+02 3.98481504684038e+02 &

7.97607558832901e+02 7.31153198242188e+02 3.95040058596455e+02 &

7.97516497216007e+02 7.34563943186781e+02 3.97383221855559e+02 &

face id 2 &

7.99360324142697e+02 7.31153198242188e+02 3.98847639366796e+02 &

7.95760540850189e+02 7.31153198242187e+02 3.98481504684038e+02 &

7.97516497216007e+02 7.34563943186781e+02 3.97383221855559e+02

; block 368

poly group "10001" mat 1 con 1 &

face id 1 &

7.99360324142697e+02 7.31153198242188e+02 3.98847639366796e+02 &

8.01878943722119e+02 7.31153198242188e+02 3.95977524084350e+02 &

7.97607558832901e+02 7.31153198242188e+02 3.95040058596455e+02 &

face id 2 &

8.01878943722119e+02 7.31153198242188e+02 3.95977524084350e+02 &

7.99360324142697e+02 7.31153198242188e+02 3.98847639366796e+02 &

7.97516497216007e+02 7.34563943186781e+02 3.97383221855559e+02 &

face id 2 &

7.97607558832901e+02 7.31153198242188e+02 3.95040058596455e+02 &

8.01878943722119e+02 7.31153198242188e+02 3.95977524084350e+02 &

7.97516497216007e+02 7.34563943186781e+02 3.97383221855559e+02 &

face id 2 &

7.99360324142697e+02 7.31153198242188e+02 3.98847639366796e+02 &

7.97607558832901e+02 7.31153198242188e+02 3.95040058596455e+02 &

7.97516497216007e+02 7.34563943186781e+02 3.97383221855559e+02

; block 369

poly group "10001" mat 1 con 1 &

face id 2 &

8.01878943722119e+02 7.31153198242188e+02 3.95977524084350e+02 &

8.05722853410866e+02 7.35087646434574e+02 3.98900933743773e+02 &

8.06591027208975e+02 7.31153198242188e+02 3.96486365333883e+02 &

face id 2 &

8.05722853410866e+02 7.35087646434574e+02 3.98900933743773e+02 &

8.01878943722119e+02 7.31153198242188e+02 3.95977524084350e+02 &

8.06533727168147e+02 7.34687160194815e+02 3.94660458496592e+02 &

face id 2 &

8.06591027208975e+02 7.31153198242188e+02 3.96486365333883e+02 &

8.05722853410866e+02 7.35087646434574e+02 3.98900933743773e+02 &

8.06533727168147e+02 7.34687160194815e+02 3.94660458496592e+02 &

face id 2 &

8.01878943722119e+02 7.31153198242188e+02 3.95977524084350e+02 &

8.06591027208975e+02 7.31153198242188e+02 3.96486365333883e+02 &

8.06533727168147e+02 7.34687160194815e+02 3.94660458496592e+02

; block 370

poly group "10001" mat 1 con 1 &

face id 2 &

7.93561308374798e+02 7.42957763671875e+02 4.04816605543573e+02 &

7.94070870845246e+02 7.42957763671875e+02 4.00551068462923e+02 &

7.95632045078570e+02 7.39829233879320e+02 4.01197287506287e+02 &

face id 1 &

7.94070870845246e+02 7.42957763671875e+02 4.00551068462923e+02 &

7.93561308374798e+02 7.42957763671875e+02 4.04816605543573e+02 &

7.91673311097484e+02 7.42957763671875e+02 4.02025301271298e+02 &

face id 2 &

7.95632045078570e+02 7.39829233879320e+02 4.01197287506287e+02 &

7.94070870845246e+02 7.42957763671875e+02 4.00551068462923e+02 &

7.91673311097484e+02 7.42957763671875e+02 4.02025301271298e+02 &

face id 2 &

7.93561308374798e+02 7.42957763671875e+02 4.04816605543573e+02 &

7.95632045078570e+02 7.39829233879320e+02 4.01197287506287e+02 &

7.91673311097484e+02 7.42957763671875e+02 4.02025301271298e+02

; block 371

poly group "10001" mat 1 con 1 &

face id 1 &

8.10167029065789e+02 7.31153198242188e+02 3.92199997436435e+02 &

8.11593521502643e+02 7.31153198242188e+02 3.97113810821721e+02 &

8.15808542739627e+02 7.31153198242188e+02 3.94330660359524e+02 &

face id 2 &

8.11593521502643e+02 7.31153198242188e+02 3.97113810821721e+02 &

8.10167029065789e+02 7.31153198242188e+02 3.92199997436435e+02 &

8.10641773231557e+02 7.37725836349123e+02 3.93566404294409e+02 &

face id 2 &

8.15808542739627e+02 7.31153198242188e+02 3.94330660359524e+02 &

8.11593521502643e+02 7.31153198242188e+02 3.97113810821721e+02 &

8.10641773231557e+02 7.37725836349123e+02 3.93566404294409e+02 &

face id 2 &

8.10167029065789e+02 7.31153198242188e+02 3.92199997436435e+02 &

8.15808542739627e+02 7.31153198242188e+02 3.94330660359524e+02 &

8.10641773231557e+02 7.37725836349123e+02 3.93566404294409e+02

; block 372

poly group "10001" mat 1 con 1 &

face id 2 &

7.89278770650053e+02 7.31153198242188e+02 3.97182633830160e+02 &

7.88322365919560e+02 7.31153198242188e+02 3.94575346324754e+02 &

7.86915466308594e+02 7.33644042968750e+02 3.97024963378906e+02 &

face id 2 &

7.88322365919560e+02 7.31153198242188e+02 3.94575346324754e+02 &

7.89278770650053e+02 7.31153198242188e+02 3.97182633830160e+02 &

7.89909651462361e+02 7.33911936376853e+02 3.96791201524795e+02 &

face id 2 &

7.86915466308594e+02 7.33644042968750e+02 3.97024963378906e+02 &

7.88322365919560e+02 7.31153198242188e+02 3.94575346324754e+02 &

7.89909651462361e+02 7.33911936376853e+02 3.96791201524795e+02 &

face id 2 &

7.89278770650053e+02 7.31153198242188e+02 3.97182633830160e+02 &

7.86915466308594e+02 7.33644042968750e+02 3.97024963378906e+02 &

7.89909651462361e+02 7.33911936376853e+02 3.96791201524795e+02

; block 373

poly group "10001" mat 1 con 1 &

face id 2 &

7.80799502317009e+02 7.38595032412240e+02 3.78079266885418e+02 &

7.77051870553251e+02 7.37085809011748e+02 3.76695713437529e+02 &

7.76623604261048e+02 7.38637497875149e+02 3.79313058357629e+02 &

face id 2 &

7.77051870553251e+02 7.37085809011748e+02 3.76695713437529e+02 &

7.80799502317009e+02 7.38595032412240e+02 3.78079266885418e+02 &

7.76938768959141e+02 7.39984334820939e+02 3.75213120298485e+02 &

face id 2 &

7.76623604261048e+02 7.38637497875149e+02 3.79313058357629e+02 &

7.77051870553251e+02 7.37085809011748e+02 3.76695713437529e+02 &

7.76938768959141e+02 7.39984334820939e+02 3.75213120298485e+02 &

face id 2 &

7.80799502317009e+02 7.38595032412240e+02 3.78079266885418e+02 &

7.76623604261048e+02 7.38637497875149e+02 3.79313058357629e+02 &

7.76938768959141e+02 7.39984334820939e+02 3.75213120298485e+02

; block 374

poly group "10001" mat 1 con 1 &

face id 2 &

7.92588269424653e+02 7.38082674352109e+02 4.07029758855016e+02 &

7.95504903662561e+02 7.39874254256717e+02 4.05138948699233e+02 &

7.94434081357035e+02 7.37853681186436e+02 4.08663893359867e+02 &

face id 2 &

7.95504903662561e+02 7.39874254256717e+02 4.05138948699233e+02 &

7.92588269424653e+02 7.38082674352109e+02 4.07029758855016e+02 &

7.94502659315746e+02 7.35572496129421e+02 4.06057284817908e+02 &

face id 2 &

7.94434081357035e+02 7.37853681186436e+02 4.08663893359867e+02 &

7.95504903662561e+02 7.39874254256717e+02 4.05138948699233e+02 &

7.94502659315746e+02 7.35572496129421e+02 4.06057284817908e+02 &

face id 2 &

7.92588269424653e+02 7.38082674352109e+02 4.07029758855016e+02 &

7.94434081357035e+02 7.37853681186436e+02 4.08663893359867e+02 &

7.94502659315746e+02 7.35572496129421e+02 4.06057284817908e+02

; block 375

poly group "10001" mat 1 con 1 &

face id 2 &

8.14343169924012e+02 7.42957763671875e+02 4.40026044980605e+02 &

8.17372340770323e+02 7.42957763671875e+02 4.38463365216075e+02 &

8.16975670140343e+02 7.39892790962574e+02 4.39960247842898e+02 &

face id 1 &

8.17372340770323e+02 7.42957763671875e+02 4.38463365216075e+02 &

8.14343169924012e+02 7.42957763671875e+02 4.40026044980605e+02 &

8.15130661800511e+02 7.42957763671875e+02 4.37033689477478e+02 &

face id 2 &

8.16975670140343e+02 7.39892790962574e+02 4.39960247842898e+02 &

8.17372340770323e+02 7.42957763671875e+02 4.38463365216075e+02 &

8.15130661800511e+02 7.42957763671875e+02 4.37033689477478e+02 &

face id 2 &

8.14343169924012e+02 7.42957763671875e+02 4.40026044980605e+02 &

8.16975670140343e+02 7.39892790962574e+02 4.39960247842898e+02 &

8.15130661800511e+02 7.42957763671875e+02 4.37033689477478e+02

; block 376

poly group "10001" mat 1 con 1 &

face id 1 &

7.96874613892984e+02 7.37657177803636e+02 4.10536208064038e+02 &

7.95275524466244e+02 7.35319480824925e+02 4.09720652974944e+02 &

7.94434081357035e+02 7.37853681186436e+02 4.08663893359867e+02 &

face id 2 &

7.95275524466244e+02 7.35319480824925e+02 4.09720652974944e+02 &

7.96874613892984e+02 7.37657177803636e+02 4.10536208064038e+02 &

7.97444340220374e+02 7.37746999378060e+02 4.07257002540444e+02 &

face id 2 &

7.94434081357035e+02 7.37853681186436e+02 4.08663893359867e+02 &

7.95275524466244e+02 7.35319480824925e+02 4.09720652974944e+02 &

7.97444340220374e+02 7.37746999378060e+02 4.07257002540444e+02 &

face id 2 &

7.96874613892984e+02 7.37657177803636e+02 4.10536208064038e+02 &

7.94434081357035e+02 7.37853681186436e+02 4.08663893359867e+02 &

7.97444340220374e+02 7.37746999378060e+02 4.07257002540444e+02

; block 377

poly group "10001" mat 1 con 1 &

face id 1 &

7.92932367677273e+02 7.31153198242188e+02 4.06808293598407e+02 &

7.91202811018456e+02 7.31153198242188e+02 4.05282018205460e+02 &

7.91396847271049e+02 7.33840276826808e+02 4.06896489211776e+02 &

face id 2 &

7.91202811018456e+02 7.31153198242188e+02 4.05282018205460e+02 &

7.92932367677273e+02 7.31153198242188e+02 4.06808293598407e+02 &

7.92348838848174e+02 7.33658540667173e+02 4.04746717291045e+02 &

face id 2 &

7.91396847271049e+02 7.33840276826808e+02 4.06896489211776e+02 &

7.91202811018456e+02 7.31153198242188e+02 4.05282018205460e+02 &

7.92348838848174e+02 7.33658540667173e+02 4.04746717291045e+02 &

face id 2 &

7.92932367677273e+02 7.31153198242188e+02 4.06808293598407e+02 &

7.91396847271049e+02 7.33840276826808e+02 4.06896489211776e+02 &

7.92348838848174e+02 7.33658540667173e+02 4.04746717291045e+02

; block 378

poly group "10001" mat 1 con 1 &

face id 1 &

7.96585601940516e+02 7.33011023940279e+02 4.10127244628184e+02 &

7.94535054290126e+02 7.33127082593229e+02 4.09095159059601e+02 &

7.95275524466244e+02 7.35319480824925e+02 4.09720652974944e+02 &

face id 2 &

7.94535054290126e+02 7.33127082593229e+02 4.09095159059601e+02 &

7.96585601940516e+02 7.33011023940279e+02 4.10127244628184e+02 &

7.95446009423280e+02 7.33370401603083e+02 4.07686153733035e+02 &

face id 2 &

7.95275524466244e+02 7.35319480824925e+02 4.09720652974944e+02 &

7.94535054290126e+02 7.33127082593229e+02 4.09095159059601e+02 &

7.95446009423280e+02 7.33370401603083e+02 4.07686153733035e+02 &

face id 2 &

7.96585601940516e+02 7.33011023940279e+02 4.10127244628184e+02 &

7.95275524466244e+02 7.35319480824925e+02 4.09720652974944e+02 &

7.95446009423280e+02 7.33370401603083e+02 4.07686153733035e+02

; block 379

poly group "10001" mat 1 con 1 &

face id 2 &

8.05267509908869e+02 7.37189947377813e+02 4.17093434190900e+02 &

8.06053783905492e+02 7.39353100736547e+02 4.13045444506629e+02 &

8.05202209472656e+02 7.40051391601562e+02 4.18037109375000e+02 &

face id 2 &

8.06053783905492e+02 7.39353100736547e+02 4.13045444506629e+02 &

8.05267509908869e+02 7.37189947377813e+02 4.17093434190900e+02 &

8.08606526611431e+02 7.39255690450706e+02 4.17166663969857e+02 &

face id 2 &

8.05202209472656e+02 7.40051391601562e+02 4.18037109375000e+02 &

8.06053783905492e+02 7.39353100736547e+02 4.13045444506629e+02 &

8.08606526611431e+02 7.39255690450706e+02 4.17166663969857e+02 &

face id 2 &

8.05267509908869e+02 7.37189947377813e+02 4.17093434190900e+02 &

8.05202209472656e+02 7.40051391601562e+02 4.18037109375000e+02 &

8.08606526611431e+02 7.39255690450706e+02 4.17166663969857e+02

; block 380

poly group "10001" mat 1 con 1 &

face id 2 &

7.98831708908744e+02 7.36125522704012e+02 3.77926771882722e+02 &

8.02547639341709e+02 7.36329323377224e+02 3.73878279205792e+02 &

7.99156871754180e+02 7.42957763671875e+02 3.74726900763980e+02 &

face id 2 &

8.02547639341709e+02 7.36329323377224e+02 3.73878279205792e+02 &

7.98831708908744e+02 7.36125522704012e+02 3.77926771882722e+02 &

8.04135590675662e+02 7.37036903814702e+02 3.79060658468265e+02 &

face id 2 &

7.99156871754180e+02 7.42957763671875e+02 3.74726900763980e+02 &

8.02547639341709e+02 7.36329323377224e+02 3.73878279205792e+02 &

8.04135590675662e+02 7.37036903814702e+02 3.79060658468265e+02 &

face id 2 &

7.98831708908744e+02 7.36125522704012e+02 3.77926771882722e+02 &

7.99156871754180e+02 7.42957763671875e+02 3.74726900763980e+02 &

8.04135590675662e+02 7.37036903814702e+02 3.79060658468265e+02

; block 381

poly group "10001" mat 1 con 1 &

face id 1 &

8.10605613244387e+02 7.31153198242188e+02 4.18975774697481e+02 &

8.06917813374775e+02 7.34261517050595e+02 4.18928731139724e+02 &

8.08622778697454e+02 7.33969474930182e+02 4.21836602431366e+02 &

face id 2 &

8.06917813374775e+02 7.34261517050595e+02 4.18928731139724e+02 &

8.10605613244387e+02 7.31153198242188e+02 4.18975774697481e+02 &

8.09490919631596e+02 7.36167724749901e+02 4.19676489619966e+02 &

face id 2 &

8.08622778697454e+02 7.33969474930182e+02 4.21836602431366e+02 &

8.06917813374775e+02 7.34261517050595e+02 4.18928731139724e+02 &

8.09490919631596e+02 7.36167724749901e+02 4.19676489619966e+02 &

face id 2 &

8.10605613244387e+02 7.31153198242188e+02 4.18975774697481e+02 &

8.08622778697454e+02 7.33969474930182e+02 4.21836602431366e+02 &

8.09490919631596e+02 7.36167724749901e+02 4.19676489619966e+02

; block 382

poly group "10001" mat 1 con 1 &

face id 2 &

8.09722830709629e+02 7.35099693077143e+02 4.16724782696196e+02 &

8.06917813374775e+02 7.34261517050595e+02 4.18928731139724e+02 &

8.10605613244387e+02 7.31153198242188e+02 4.18975774697481e+02 &

face id 2 &

8.06917813374775e+02 7.34261517050595e+02 4.18928731139724e+02 &

8.09722830709629e+02 7.35099693077143e+02 4.16724782696196e+02 &

8.09490919631596e+02 7.36167724749901e+02 4.19676489619966e+02 &

face id 2 &

8.10605613244387e+02 7.31153198242188e+02 4.18975774697481e+02 &

8.06917813374775e+02 7.34261517050595e+02 4.18928731139724e+02 &

8.09490919631596e+02 7.36167724749901e+02 4.19676489619966e+02 &

face id 2 &

8.09722830709629e+02 7.35099693077143e+02 4.16724782696196e+02 &

8.10605613244387e+02 7.31153198242188e+02 4.18975774697481e+02 &

8.09490919631596e+02 7.36167724749901e+02 4.19676489619966e+02

; block 383

poly group "10001" mat 1 con 1 &

face id 2 &

8.22847947705952e+02 7.31153198242188e+02 4.22600298353562e+02 &

8.22003939543759e+02 7.37603465338854e+02 4.20982666462121e+02 &

8.18663640401986e+02 7.31153198242188e+02 4.20323564658305e+02 &

face id 2 &

8.22003939543759e+02 7.37603465338854e+02 4.20982666462121e+02 &

8.22847947705952e+02 7.31153198242188e+02 4.22600298353562e+02 &

8.21662862454680e+02 7.34770629444877e+02 4.24506602520138e+02 &

face id 2 &

8.18663640401986e+02 7.31153198242188e+02 4.20323564658305e+02 &

8.22003939543759e+02 7.37603465338854e+02 4.20982666462121e+02 &

8.21662862454680e+02 7.34770629444877e+02 4.24506602520138e+02 &

face id 2 &

8.22847947705952e+02 7.31153198242188e+02 4.22600298353562e+02 &

8.18663640401986e+02 7.31153198242188e+02 4.20323564658305e+02 &

8.21662862454680e+02 7.34770629444877e+02 4.24506602520138e+02

; block 384

poly group "10001" mat 1 con 1 &

face id 2 &

7.82786128969092e+02 7.37151441981042e+02 3.68867086099698e+02 &

7.80785664505661e+02 7.39236281877246e+02 3.73124042480188e+02 &

7.80279121453295e+02 7.34974032328978e+02 3.74087850984229e+02 &

face id 2 &

7.80785664505661e+02 7.39236281877246e+02 3.73124042480188e+02 &

7.82786128969092e+02 7.37151441981042e+02 3.68867086099698e+02 &

7.85027598845248e+02 7.38476661150185e+02 3.72345633894984e+02 &

face id 2 &

7.80279121453295e+02 7.34974032328978e+02 3.74087850984229e+02 &

7.80785664505661e+02 7.39236281877246e+02 3.73124042480188e+02 &

7.85027598845248e+02 7.38476661150185e+02 3.72345633894984e+02 &

face id 2 &

7.82786128969092e+02 7.37151441981042e+02 3.68867086099698e+02 &

7.80279121453295e+02 7.34974032328978e+02 3.74087850984229e+02 &

7.85027598845248e+02 7.38476661150185e+02 3.72345633894984e+02

; block 385

poly group "10001" mat 1 con 1 &

face id 1 &

8.13058615733111e+02 7.31153198242188e+02 4.26040031870464e+02 &

8.16626759213409e+02 7.31153198242188e+02 4.27014493977602e+02 &

8.15880858022893e+02 7.31153198242188e+02 4.23505248338825e+02 &

face id 2 &

8.16626759213409e+02 7.31153198242188e+02 4.27014493977602e+02 &

8.13058615733111e+02 7.31153198242188e+02 4.26040031870464e+02 &

8.15135590016714e+02 7.34048471724572e+02 4.27166808243313e+02 &

face id 2 &

8.15880858022893e+02 7.31153198242188e+02 4.23505248338825e+02 &

8.16626759213409e+02 7.31153198242188e+02 4.27014493977602e+02 &

8.15135590016714e+02 7.34048471724572e+02 4.27166808243313e+02 &

face id 2 &

8.13058615733111e+02 7.31153198242188e+02 4.26040031870464e+02 &

8.15880858022893e+02 7.31153198242188e+02 4.23505248338825e+02 &

8.15135590016714e+02 7.34048471724572e+02 4.27166808243313e+02

; block 386

poly group "10001" mat 1 con 1 &

face id 2 &

7.89669829571038e+02 7.31153198242188e+02 3.99434746165080e+02 &

7.91099717380957e+02 7.31153198242188e+02 4.01060586132354e+02 &

7.90671467575070e+02 7.33496249726850e+02 4.02031030029439e+02 &

face id 1 &

7.91099717380957e+02 7.31153198242188e+02 4.01060586132354e+02 &

7.89669829571038e+02 7.31153198242188e+02 3.99434746165080e+02 &

7.88470336914062e+02 7.31153198242188e+02 4.01435272216797e+02 &

face id 2 &

7.90671467575070e+02 7.33496249726850e+02 4.02031030029439e+02 &

7.91099717380957e+02 7.31153198242188e+02 4.01060586132354e+02 &

7.88470336914062e+02 7.31153198242188e+02 4.01435272216797e+02 &

face id 2 &

7.89669829571038e+02 7.31153198242188e+02 3.99434746165080e+02 &

7.90671467575070e+02 7.33496249726850e+02 4.02031030029439e+02 &

7.88470336914062e+02 7.31153198242188e+02 4.01435272216797e+02

; block 387

poly group "10001" mat 1 con 1 &

face id 2 &

7.78389795522681e+02 7.31153198242188e+02 3.79403540956464e+02 &

7.75901329829113e+02 7.31153198242188e+02 3.77553750069347e+02 &

7.76690916896458e+02 7.33984982361572e+02 3.81358736446098e+02 &

face id 2 &

7.75901329829113e+02 7.31153198242188e+02 3.77553750069347e+02 &

7.78389795522681e+02 7.31153198242188e+02 3.79403540956464e+02 &

7.76629831384941e+02 7.34030537730047e+02 3.76463041133043e+02 &

face id 2 &

7.76690916896458e+02 7.33984982361572e+02 3.81358736446098e+02 &

7.75901329829113e+02 7.31153198242188e+02 3.77553750069347e+02 &

7.76629831384941e+02 7.34030537730047e+02 3.76463041133043e+02 &

face id 2 &

7.78389795522681e+02 7.31153198242188e+02 3.79403540956464e+02 &

7.76690916896458e+02 7.33984982361572e+02 3.81358736446098e+02 &

7.76629831384941e+02 7.34030537730047e+02 3.76463041133043e+02

; block 388

poly group "10001" mat 1 con 1 &

face id 2 &

7.76690916896458e+02 7.33984982361572e+02 3.81358736446098e+02 &

7.79511950549799e+02 7.34457282336568e+02 3.78709527306644e+02 &

7.78389795522681e+02 7.31153198242188e+02 3.79403540956464e+02 &

face id 2 &

7.79511950549799e+02 7.34457282336568e+02 3.78709527306644e+02 &

7.76690916896458e+02 7.33984982361572e+02 3.81358736446098e+02 &

7.76629831384941e+02 7.34030537730047e+02 3.76463041133043e+02 &

face id 2 &

7.78389795522681e+02 7.31153198242188e+02 3.79403540956464e+02 &

7.79511950549799e+02 7.34457282336568e+02 3.78709527306644e+02 &

7.76629831384941e+02 7.34030537730047e+02 3.76463041133043e+02 &

face id 2 &

7.76690916896458e+02 7.33984982361572e+02 3.81358736446098e+02 &

7.78389795522681e+02 7.31153198242188e+02 3.79403540956464e+02 &

7.76629831384941e+02 7.34030537730047e+02 3.76463041133043e+02

; block 389

poly group "10001" mat 1 con 1 &

face id 2 &

8.19582997168244e+02 7.40188540403784e+02 4.34477675949180e+02 &

8.16320558032978e+02 7.42957763671875e+02 4.31298908485349e+02 &

8.20009998744681e+02 7.42957763671875e+02 4.32354041528251e+02 &

face id 2 &

8.16320558032978e+02 7.42957763671875e+02 4.31298908485349e+02 &

8.19582997168244e+02 7.40188540403784e+02 4.34477675949180e+02 &

8.18073172819596e+02 7.39472123297221e+02 4.29992682558518e+02 &

face id 2 &

8.20009998744681e+02 7.42957763671875e+02 4.32354041528251e+02 &

8.16320558032978e+02 7.42957763671875e+02 4.31298908485349e+02 &

8.18073172819596e+02 7.39472123297221e+02 4.29992682558518e+02 &

face id 2 &

8.19582997168244e+02 7.40188540403784e+02 4.34477675949180e+02 &

8.20009998744681e+02 7.42957763671875e+02 4.32354041528251e+02 &

8.18073172819596e+02 7.39472123297221e+02 4.29992682558518e+02

; block 390

poly group "10001" mat 1 con 1 &

face id 2 &

7.87436025160242e+02 7.32691984698342e+02 3.98548675430130e+02 &

7.89669829571038e+02 7.31153198242188e+02 3.99434746165080e+02 &

7.87470807425448e+02 7.31153198242188e+02 3.99267743862157e+02 &

face id 2 &

7.89669829571038e+02 7.31153198242188e+02 3.99434746165080e+02 &

7.87436025160242e+02 7.32691984698342e+02 3.98548675430130e+02 &

7.89278770650053e+02 7.31153198242188e+02 3.97182633830160e+02 &

face id 1 &

7.87470807425448e+02 7.31153198242188e+02 3.99267743862157e+02 &

7.89669829571038e+02 7.31153198242188e+02 3.99434746165080e+02 &

7.89278770650053e+02 7.31153198242188e+02 3.97182633830160e+02 &

face id 2 &

7.87436025160242e+02 7.32691984698342e+02 3.98548675430130e+02 &

7.87470807425448e+02 7.31153198242188e+02 3.99267743862157e+02 &

7.89278770650053e+02 7.31153198242188e+02 3.97182633830160e+02

; block 391

poly group "10001" mat 1 con 1 &

face id 2 &

7.85839298561526e+02 7.31153198242188e+02 3.88082261200957e+02 &

7.84076891175360e+02 7.31153198242188e+02 3.91169905157991e+02 &

7.84182101630836e+02 7.33952564315048e+02 3.89093205382393e+02 &

face id 1 &

7.84076891175360e+02 7.31153198242188e+02 3.91169905157991e+02 &

7.85839298561526e+02 7.31153198242188e+02 3.88082261200957e+02 &

7.82307835025844e+02 7.31153198242188e+02 3.88140687950919e+02 &

face id 1 &

7.84182101630836e+02 7.33952564315048e+02 3.89093205382393e+02 &

7.84076891175360e+02 7.31153198242188e+02 3.91169905157991e+02 &

7.82307835025844e+02 7.31153198242188e+02 3.88140687950919e+02 &

face id 2 &

7.85839298561526e+02 7.31153198242188e+02 3.88082261200957e+02 &

7.84182101630836e+02 7.33952564315048e+02 3.89093205382393e+02 &

7.82307835025844e+02 7.31153198242188e+02 3.88140687950919e+02

; block 392

poly group "10001" mat 1 con 1 &

face id 1 &

8.29507144346306e+02 7.31153198242188e+02 4.41289893556219e+02 &

8.33038277772156e+02 7.31153198242188e+02 4.44424962119596e+02 &

8.33328695733478e+02 7.31153198242188e+02 4.38258345423521e+02 &

face id 2 &

8.33038277772156e+02 7.31153198242188e+02 4.44424962119596e+02 &

8.29507144346306e+02 7.31153198242188e+02 4.41289893556219e+02 &

8.34729885421035e+02 7.36525928807045e+02 4.41869552698341e+02 &

face id 2 &

8.33328695733478e+02 7.31153198242188e+02 4.38258345423521e+02 &

8.33038277772156e+02 7.31153198242188e+02 4.44424962119596e+02 &

8.34729885421035e+02 7.36525928807045e+02 4.41869552698341e+02 &

face id 2 &

8.29507144346306e+02 7.31153198242188e+02 4.41289893556219e+02 &

8.33328695733478e+02 7.31153198242188e+02 4.38258345423521e+02 &

8.34729885421035e+02 7.36525928807045e+02 4.41869552698341e+02

; block 393

poly group "10001" mat 1 con 1 &

face id 1 &

8.29507144346306e+02 7.31153198242188e+02 4.41289893556219e+02 &

8.25415422619949e+02 7.31153198242188e+02 4.41338127497763e+02 &

8.27040491723330e+02 7.31153198242188e+02 4.44660601575022e+02 &

face id 2 &

8.25415422619949e+02 7.31153198242188e+02 4.41338127497763e+02 &

8.29507144346306e+02 7.31153198242188e+02 4.41289893556219e+02 &

8.26114508878027e+02 7.34878405664679e+02 4.40774789501596e+02 &

face id 2 &

8.27040491723330e+02 7.31153198242188e+02 4.44660601575022e+02 &

8.25415422619949e+02 7.31153198242188e+02 4.41338127497763e+02 &

8.26114508878027e+02 7.34878405664679e+02 4.40774789501596e+02 &

face id 2 &

8.29507144346306e+02 7.31153198242188e+02 4.41289893556219e+02 &

8.27040491723330e+02 7.31153198242188e+02 4.44660601575022e+02 &

8.26114508878027e+02 7.34878405664679e+02 4.40774789501596e+02

; block 394

poly group "10001" mat 1 con 1 &

face id 1 &

7.84401767048789e+02 7.37072043945781e+02 3.86376068513752e+02 &

7.86133875572391e+02 7.37145664379186e+02 3.89509305279638e+02 &

7.84182101630836e+02 7.33952564315048e+02 3.89093205382393e+02 &

face id 2 &

7.86133875572391e+02 7.37145664379186e+02 3.89509305279638e+02 &

7.84401767048789e+02 7.37072043945781e+02 3.86376068513752e+02 &

7.87643654364816e+02 7.34661112155498e+02 3.86945147752232e+02 &

face id 2 &

7.84182101630836e+02 7.33952564315048e+02 3.89093205382393e+02 &

7.86133875572391e+02 7.37145664379186e+02 3.89509305279638e+02 &

7.87643654364816e+02 7.34661112155498e+02 3.86945147752232e+02 &

face id 2 &

7.84401767048789e+02 7.37072043945781e+02 3.86376068513752e+02 &

7.84182101630836e+02 7.33952564315048e+02 3.89093205382393e+02 &

7.87643654364816e+02 7.34661112155498e+02 3.86945147752232e+02

; block 395

poly group "10001" mat 1 con 1 &

face id 1 &

7.79046047912539e+02 7.31153198242188e+02 3.75832298195535e+02 &

7.75901329829113e+02 7.31153198242188e+02 3.77553750069347e+02 &

7.78389795522681e+02 7.31153198242188e+02 3.79403540956464e+02 &

face id 2 &

7.75901329829113e+02 7.31153198242188e+02 3.77553750069347e+02 &

7.79046047912539e+02 7.31153198242188e+02 3.75832298195535e+02 &

7.76629831384941e+02 7.34030537730047e+02 3.76463041133043e+02 &

face id 2 &

7.78389795522681e+02 7.31153198242188e+02 3.79403540956464e+02 &

7.75901329829113e+02 7.31153198242188e+02 3.77553750069347e+02 &

7.76629831384941e+02 7.34030537730047e+02 3.76463041133043e+02 &

face id 2 &

7.79046047912539e+02 7.31153198242188e+02 3.75832298195535e+02 &

7.78389795522681e+02 7.31153198242188e+02 3.79403540956464e+02 &

7.76629831384941e+02 7.34030537730047e+02 3.76463041133043e+02

; block 396

poly group "10001" mat 1 con 1 &

face id 1 &

8.27040491723330e+02 7.31153198242188e+02 4.44660601575022e+02 &

8.25415422619949e+02 7.31153198242188e+02 4.41338127497763e+02 &

8.24066589355469e+02 7.33591491699219e+02 4.44044647216797e+02 &

face id 2 &

8.25415422619949e+02 7.31153198242188e+02 4.41338127497763e+02 &

8.27040491723330e+02 7.31153198242188e+02 4.44660601575022e+02 &

8.26114508878027e+02 7.34878405664679e+02 4.40774789501596e+02 &

face id 2 &

8.24066589355469e+02 7.33591491699219e+02 4.44044647216797e+02 &

8.25415422619949e+02 7.31153198242188e+02 4.41338127497763e+02 &

8.26114508878027e+02 7.34878405664679e+02 4.40774789501596e+02 &

face id 2 &

8.27040491723330e+02 7.31153198242188e+02 4.44660601575022e+02 &

8.24066589355469e+02 7.33591491699219e+02 4.44044647216797e+02 &

8.26114508878027e+02 7.34878405664679e+02 4.40774789501596e+02

; block 397

poly group "10001" mat 1 con 1 &

face id 2 &

7.79552550043064e+02 7.34262768372563e+02 3.83255082743573e+02 &

7.78389795522681e+02 7.31153198242188e+02 3.79403540956464e+02 &

7.81100846896327e+02 7.31153198242188e+02 3.81735958194611e+02 &

face id 2 &

7.78389795522681e+02 7.31153198242188e+02 3.79403540956464e+02 &

7.79552550043064e+02 7.34262768372563e+02 3.83255082743573e+02 &

7.77537120707883e+02 7.31153198242188e+02 3.82763768064916e+02 &

face id 1 &

7.81100846896327e+02 7.31153198242188e+02 3.81735958194611e+02 &

7.78389795522681e+02 7.31153198242188e+02 3.79403540956464e+02 &

7.77537120707883e+02 7.31153198242188e+02 3.82763768064916e+02 &

face id 2 &

7.79552550043064e+02 7.34262768372563e+02 3.83255082743573e+02 &

7.81100846896327e+02 7.31153198242188e+02 3.81735958194611e+02 &

7.77537120707883e+02 7.31153198242188e+02 3.82763768064916e+02

; block 398

poly group "10001" mat 1 con 1 &

face id 2 &

7.78389795522681e+02 7.31153198242188e+02 3.79403540956464e+02 &

7.79511950549799e+02 7.34457282336568e+02 3.78709527306644e+02 &

7.79046047912539e+02 7.31153198242188e+02 3.75832298195535e+02 &

face id 2 &

7.79511950549799e+02 7.34457282336568e+02 3.78709527306644e+02 &

7.78389795522681e+02 7.31153198242188e+02 3.79403540956464e+02 &

7.76629831384941e+02 7.34030537730047e+02 3.76463041133043e+02 &

face id 2 &

7.79046047912539e+02 7.31153198242188e+02 3.75832298195535e+02 &

7.79511950549799e+02 7.34457282336568e+02 3.78709527306644e+02 &

7.76629831384941e+02 7.34030537730047e+02 3.76463041133043e+02 &

face id 2 &

7.78389795522681e+02 7.31153198242188e+02 3.79403540956464e+02 &

7.79046047912539e+02 7.31153198242188e+02 3.75832298195535e+02 &

7.76629831384941e+02 7.34030537730047e+02 3.76463041133043e+02

; block 399

poly group "10001" mat 1 con 1 &

face id 1 &

8.27040491723330e+02 7.31153198242188e+02 4.44660601575022e+02 &

8.24066589355469e+02 7.33591491699219e+02 4.44044647216797e+02 &

8.25835327087865e+02 7.34466611642151e+02 4.46980813661767e+02 &

face id 2 &

8.24066589355469e+02 7.33591491699219e+02 4.44044647216797e+02 &

8.27040491723330e+02 7.31153198242188e+02 4.44660601575022e+02 &

8.29251774542374e+02 7.34873958371039e+02 4.43856971841759e+02 &

face id 2 &

8.25835327087865e+02 7.34466611642151e+02 4.46980813661767e+02 &

8.24066589355469e+02 7.33591491699219e+02 4.44044647216797e+02 &

8.29251774542374e+02 7.34873958371039e+02 4.43856971841759e+02 &

face id 2 &

8.27040491723330e+02 7.31153198242188e+02 4.44660601575022e+02 &

8.25835327087865e+02 7.34466611642151e+02 4.46980813661767e+02 &

8.29251774542374e+02 7.34873958371039e+02 4.43856971841759e+02

; block 400

poly group "10001" mat 1 con 1 &

face id 1 &

8.24135792527692e+02 7.31153198242188e+02 4.34857938641253e+02 &

8.23611633300781e+02 7.31153198242188e+02 4.38513122558594e+02 &

8.27899766625565e+02 7.31153198242188e+02 4.37953027515864e+02 &

face id 2 &

8.23611633300781e+02 7.31153198242188e+02 4.38513122558594e+02 &

8.24135792527692e+02 7.31153198242188e+02 4.34857938641253e+02 &

8.23363932724725e+02 7.33802420140527e+02 4.37276399935772e+02 &

face id 2 &

8.27899766625565e+02 7.31153198242188e+02 4.37953027515864e+02 &

8.23611633300781e+02 7.31153198242188e+02 4.38513122558594e+02 &

8.23363932724725e+02 7.33802420140527e+02 4.37276399935772e+02 &

face id 2 &

8.24135792527692e+02 7.31153198242188e+02 4.34857938641253e+02 &

8.27899766625565e+02 7.31153198242188e+02 4.37953027515864e+02 &

8.23363932724725e+02 7.33802420140527e+02 4.37276399935772e+02

; block 401

poly group "10001" mat 1 con 1 &

face id 2 &

8.22048825758541e+02 7.38705365341897e+02 4.27519172119006e+02 &

8.18073172819596e+02 7.39472123297221e+02 4.29992682558518e+02 &

8.18126133140210e+02 7.39128284797895e+02 4.24521969903783e+02 &

face id 2 &

8.18073172819596e+02 7.39472123297221e+02 4.29992682558518e+02 &

8.22048825758541e+02 7.38705365341897e+02 4.27519172119006e+02 &

8.16363471593303e+02 7.36867764055710e+02 4.27997707946941e+02 &

face id 2 &

8.18126133140210e+02 7.39128284797895e+02 4.24521969903783e+02 &

8.18073172819596e+02 7.39472123297221e+02 4.29992682558518e+02 &

8.16363471593303e+02 7.36867764055710e+02 4.27997707946941e+02 &

face id 2 &

8.22048825758541e+02 7.38705365341897e+02 4.27519172119006e+02 &

8.18126133140210e+02 7.39128284797895e+02 4.24521969903783e+02 &

8.16363471593303e+02 7.36867764055710e+02 4.27997707946941e+02

; block 402

poly group "10001" mat 1 con 1 &

face id 1 &

8.39084289550781e+02 7.31153198242188e+02 4.28616635958402e+02 &

8.39084289550781e+02 7.37015214063697e+02 4.30185524731766e+02 &

8.39084289550781e+02 7.37102521277678e+02 4.23975409053137e+02 &

face id 2 &

8.39084289550781e+02 7.37015214063697e+02 4.30185524731766e+02 &

8.39084289550781e+02 7.31153198242188e+02 4.28616635958402e+02 &

8.33405021547574e+02 7.37173781106677e+02 4.27268238018421e+02 &

face id 2 &

8.39084289550781e+02 7.37102521277678e+02 4.23975409053137e+02 &

8.39084289550781e+02 7.37015214063697e+02 4.30185524731766e+02 &

8.33405021547574e+02 7.37173781106677e+02 4.27268238018421e+02 &

face id 2 &

8.39084289550781e+02 7.31153198242188e+02 4.28616635958402e+02 &

8.39084289550781e+02 7.37102521277678e+02 4.23975409053137e+02 &

8.33405021547574e+02 7.37173781106677e+02 4.27268238018421e+02

; block 403

poly group "10001" mat 1 con 1 &

face id 1 &

7.89654758545250e+02 7.37121377960737e+02 4.03007864821918e+02 &

7.91616421383069e+02 7.39816178005118e+02 4.04519104605588e+02 &

7.91230906089945e+02 7.36766305600082e+02 4.05813632054086e+02 &

face id 2 &

7.91616421383069e+02 7.39816178005118e+02 4.04519104605588e+02 &

7.89654758545250e+02 7.37121377960737e+02 4.03007864821918e+02 &

7.93177635869142e+02 7.36964441637668e+02 4.03028389020813e+02 &

face id 2 &

7.91230906089945e+02 7.36766305600082e+02 4.05813632054086e+02 &

7.91616421383069e+02 7.39816178005118e+02 4.04519104605588e+02 &

7.93177635869142e+02 7.36964441637668e+02 4.03028389020813e+02 &

face id 2 &

7.89654758545250e+02 7.37121377960737e+02 4.03007864821918e+02 &

7.91230906089945e+02 7.36766305600082e+02 4.05813632054086e+02 &

7.93177635869142e+02 7.36964441637668e+02 4.03028389020813e+02

; block 404

poly group "10001" mat 1 con 1 &

face id 1 &

8.09526154163339e+02 7.35873050296643e+02 4.28779842855120e+02 &

8.11157068079853e+02 7.33246730421718e+02 4.28032237505750e+02 &

8.09332280963208e+02 7.35603617621337e+02 4.26448846175998e+02 &

face id 2 &

8.11157068079853e+02 7.33246730421718e+02 4.28032237505750e+02 &

8.09526154163339e+02 7.35873050296643e+02 4.28779842855120e+02 &

8.12106141232222e+02 7.36413785110797e+02 4.27506689060193e+02 &

face id 2 &

8.09332280963208e+02 7.35603617621337e+02 4.26448846175998e+02 &

8.11157068079853e+02 7.33246730421718e+02 4.28032237505750e+02 &

8.12106141232222e+02 7.36413785110797e+02 4.27506689060193e+02 &

face id 2 &

8.09526154163339e+02 7.35873050296643e+02 4.28779842855120e+02 &

8.09332280963208e+02 7.35603617621337e+02 4.26448846175998e+02 &

8.12106141232222e+02 7.36413785110797e+02 4.27506689060193e+02

; block 405

poly group "10001" mat 1 con 1 &

face id 2 &

8.09332280963208e+02 7.35603617621337e+02 4.26448846175998e+02 &

8.10984080669042e+02 7.39211793104898e+02 4.26585347528298e+02 &

8.09526154163339e+02 7.35873050296643e+02 4.28779842855120e+02 &

face id 2 &

8.10984080669042e+02 7.39211793104898e+02 4.26585347528298e+02 &

8.09332280963208e+02 7.35603617621337e+02 4.26448846175998e+02 &

8.12106141232222e+02 7.36413785110797e+02 4.27506689060193e+02 &

face id 2 &

8.09526154163339e+02 7.35873050296643e+02 4.28779842855120e+02 &

8.10984080669042e+02 7.39211793104898e+02 4.26585347528298e+02 &

8.12106141232222e+02 7.36413785110797e+02 4.27506689060193e+02 &

face id 2 &

8.09332280963208e+02 7.35603617621337e+02 4.26448846175998e+02 &

8.09526154163339e+02 7.35873050296643e+02 4.28779842855120e+02 &

8.12106141232222e+02 7.36413785110797e+02 4.27506689060193e+02

; block 406

poly group "10001" mat 1 con 1 &

face id 1 &

8.20017509411527e+02 7.31153198242188e+02 4.28097071064497e+02 &

8.16626759213409e+02 7.31153198242188e+02 4.27014493977602e+02 &

8.17436891461985e+02 7.31153198242188e+02 4.30682311023628e+02 &

face id 2 &

8.16626759213409e+02 7.31153198242188e+02 4.27014493977602e+02 &

8.20017509411527e+02 7.31153198242188e+02 4.28097071064497e+02 &

8.19506582324828e+02 7.34677513556790e+02 4.29945141932819e+02 &

face id 2 &

8.17436891461985e+02 7.31153198242188e+02 4.30682311023628e+02 &

8.16626759213409e+02 7.31153198242188e+02 4.27014493977602e+02 &

8.19506582324828e+02 7.34677513556790e+02 4.29945141932819e+02 &

face id 2 &

8.20017509411527e+02 7.31153198242188e+02 4.28097071064497e+02 &

8.17436891461985e+02 7.31153198242188e+02 4.30682311023628e+02 &

8.19506582324828e+02 7.34677513556790e+02 4.29945141932819e+02

; block 407

poly group "10001" mat 1 con 1 &

face id 2 &

8.18073172819596e+02 7.39472123297221e+02 4.29992682558518e+02 &

8.22048825758541e+02 7.38705365341897e+02 4.27519172119006e+02 &

8.19506582324828e+02 7.34677513556790e+02 4.29945141932819e+02 &

face id 2 &

8.22048825758541e+02 7.38705365341897e+02 4.27519172119006e+02 &

8.18073172819596e+02 7.39472123297221e+02 4.29992682558518e+02 &

8.16363471593303e+02 7.36867764055710e+02 4.27997707946941e+02 &

face id 2 &

8.19506582324828e+02 7.34677513556790e+02 4.29945141932819e+02 &

8.22048825758541e+02 7.38705365341897e+02 4.27519172119006e+02 &

8.16363471593303e+02 7.36867764055710e+02 4.27997707946941e+02 &

face id 2 &

8.18073172819596e+02 7.39472123297221e+02 4.29992682558518e+02 &

8.19506582324828e+02 7.34677513556790e+02 4.29945141932819e+02 &

8.16363471593303e+02 7.36867764055710e+02 4.27997707946941e+02

; block 408

poly group "10001" mat 1 con 1 &

face id 2 &

8.18663640401986e+02 7.31153198242188e+02 4.20323564658305e+02 &

8.22003939543759e+02 7.37603465338854e+02 4.20982666462121e+02 &

8.16747344865554e+02 7.34789113348238e+02 4.20781967480880e+02 &

face id 2 &

8.22003939543759e+02 7.37603465338854e+02 4.20982666462121e+02 &

8.18663640401986e+02 7.31153198242188e+02 4.20323564658305e+02 &

8.21662862454680e+02 7.34770629444877e+02 4.24506602520138e+02 &

face id 2 &

8.16747344865554e+02 7.34789113348238e+02 4.20781967480880e+02 &

8.22003939543759e+02 7.37603465338854e+02 4.20982666462121e+02 &

8.21662862454680e+02 7.34770629444877e+02 4.24506602520138e+02 &

face id 2 &

8.18663640401986e+02 7.31153198242188e+02 4.20323564658305e+02 &

8.16747344865554e+02 7.34789113348238e+02 4.20781967480880e+02 &

8.21662862454680e+02 7.34770629444877e+02 4.24506602520138e+02

; block 409

poly group "10001" mat 1 con 1 &

face id 1 &

7.90619961402637e+02 7.31153198242188e+02 3.69409004990024e+02 &

7.93276514590484e+02 7.31153198242188e+02 3.74383781487787e+02 &

7.96234950413729e+02 7.31153198242188e+02 3.69842370472253e+02 &

face id 2 &

7.93276514590484e+02 7.31153198242188e+02 3.74383781487787e+02 &

7.90619961402637e+02 7.31153198242188e+02 3.69409004990024e+02 &

7.93658752091436e+02 7.34748621680107e+02 3.69706280758364e+02 &

face id 2 &

7.96234950413729e+02 7.31153198242188e+02 3.69842370472253e+02 &

7.93276514590484e+02 7.31153198242188e+02 3.74383781487787e+02 &

7.93658752091436e+02 7.34748621680107e+02 3.69706280758364e+02 &

face id 2 &

7.90619961402637e+02 7.31153198242188e+02 3.69409004990024e+02 &

7.96234950413729e+02 7.31153198242188e+02 3.69842370472253e+02 &

7.93658752091436e+02 7.34748621680107e+02 3.69706280758364e+02

; block 410

poly group "10001" mat 1 con 1 &

face id 1 &

8.13058615733111e+02 7.31153198242188e+02 4.26040031870464e+02 &

8.10294982910156e+02 7.33683044433594e+02 4.24688598632812e+02 &

8.11157068079853e+02 7.33246730421718e+02 4.28032237505750e+02 &

face id 2 &

8.10294982910156e+02 7.33683044433594e+02 4.24688598632812e+02 &

8.13058615733111e+02 7.31153198242188e+02 4.26040031870464e+02 &

8.13694588926832e+02 7.34627007610324e+02 4.24114636773096e+02 &

face id 2 &

8.11157068079853e+02 7.33246730421718e+02 4.28032237505750e+02 &

8.10294982910156e+02 7.33683044433594e+02 4.24688598632812e+02 &

8.13694588926832e+02 7.34627007610324e+02 4.24114636773096e+02 &

face id 2 &

8.13058615733111e+02 7.31153198242188e+02 4.26040031870464e+02 &

8.11157068079853e+02 7.33246730421718e+02 4.28032237505750e+02 &

8.13694588926832e+02 7.34627007610324e+02 4.24114636773096e+02

; block 411

poly group "10001" mat 1 con 1 &

face id 1 &

7.86133875572391e+02 7.37145664379186e+02 3.89509305279638e+02 &

7.84401767048789e+02 7.37072043945781e+02 3.86376068513752e+02 &

7.86847560388448e+02 7.40049255151163e+02 3.86796454113899e+02 &

face id 2 &

7.84401767048789e+02 7.37072043945781e+02 3.86376068513752e+02 &

7.86133875572391e+02 7.37145664379186e+02 3.89509305279638e+02 &

7.87643654364816e+02 7.34661112155498e+02 3.86945147752232e+02 &

face id 2 &

7.86847560388448e+02 7.40049255151163e+02 3.86796454113899e+02 &

7.84401767048789e+02 7.37072043945781e+02 3.86376068513752e+02 &

7.87643654364816e+02 7.34661112155498e+02 3.86945147752232e+02 &

face id 2 &

7.86133875572391e+02 7.37145664379186e+02 3.89509305279638e+02 &

7.86847560388448e+02 7.40049255151163e+02 3.86796454113899e+02 &

7.87643654364816e+02 7.34661112155498e+02 3.86945147752232e+02

; block 412

poly group "10001" mat 1 con 1 &

face id 2 &

7.86847560388448e+02 7.40049255151163e+02 3.86796454113899e+02 &

7.89863937233331e+02 7.38539747715945e+02 3.87566254647709e+02 &

7.87643654364816e+02 7.34661112155498e+02 3.86945147752232e+02 &

face id 2 &

7.89863937233331e+02 7.38539747715945e+02 3.87566254647709e+02 &

7.86847560388448e+02 7.40049255151163e+02 3.86796454113899e+02 &

7.87710389350481e+02 7.38310198672720e+02 3.83817696746716e+02 &

face id 2 &

7.87643654364816e+02 7.34661112155498e+02 3.86945147752232e+02 &

7.89863937233331e+02 7.38539747715945e+02 3.87566254647709e+02 &

7.87710389350481e+02 7.38310198672720e+02 3.83817696746716e+02 &

face id 2 &

7.86847560388448e+02 7.40049255151163e+02 3.86796454113899e+02 &

7.87643654364816e+02 7.34661112155498e+02 3.86945147752232e+02 &

7.87710389350481e+02 7.38310198672720e+02 3.83817696746716e+02

; block 413

poly group "10001" mat 1 con 1 &

face id 2 &

7.89863937233331e+02 7.38539747715945e+02 3.87566254647709e+02 &

7.89808816568164e+02 7.35078718869257e+02 3.82888823368539e+02 &

7.87643654364816e+02 7.34661112155498e+02 3.86945147752232e+02 &

face id 2 &

7.89808816568164e+02 7.35078718869257e+02 3.82888823368539e+02 &

7.89863937233331e+02 7.38539747715945e+02 3.87566254647709e+02 &

7.87710389350481e+02 7.38310198672720e+02 3.83817696746716e+02 &

face id 2 &

7.87643654364816e+02 7.34661112155498e+02 3.86945147752232e+02 &

7.89808816568164e+02 7.35078718869257e+02 3.82888823368539e+02 &

7.87710389350481e+02 7.38310198672720e+02 3.83817696746716e+02 &

face id 2 &

7.89863937233331e+02 7.38539747715945e+02 3.87566254647709e+02 &

7.87643654364816e+02 7.34661112155498e+02 3.86945147752232e+02 &

7.87710389350481e+02 7.38310198672720e+02 3.83817696746716e+02

; block 414

poly group "10001" mat 1 con 1 &

face id 2 &

8.15880858022893e+02 7.31153198242188e+02 4.23505248338825e+02 &

8.13694588926832e+02 7.34627007610324e+02 4.24114636773096e+02 &

8.13058615733111e+02 7.31153198242188e+02 4.26040031870464e+02 &

face id 2 &

8.13694588926832e+02 7.34627007610324e+02 4.24114636773096e+02 &

8.15880858022893e+02 7.31153198242188e+02 4.23505248338825e+02 &

8.15135590016714e+02 7.34048471724572e+02 4.27166808243313e+02 &

face id 2 &

8.13058615733111e+02 7.31153198242188e+02 4.26040031870464e+02 &

8.13694588926832e+02 7.34627007610324e+02 4.24114636773096e+02 &

8.15135590016714e+02 7.34048471724572e+02 4.27166808243313e+02 &

face id 2 &

8.15880858022893e+02 7.31153198242188e+02 4.23505248338825e+02 &

8.13058615733111e+02 7.31153198242188e+02 4.26040031870464e+02 &

8.15135590016714e+02 7.34048471724572e+02 4.27166808243313e+02

; block 415

poly group "10001" mat 1 con 1 &

face id 1 &

7.95249005168754e+02 7.42957763671875e+02 3.77395501535433e+02 &

7.93502350955747e+02 7.42957763671875e+02 3.73386897874445e+02 &

7.99156871754180e+02 7.42957763671875e+02 3.74726900763980e+02 &

face id 2 &

7.93502350955747e+02 7.42957763671875e+02 3.73386897874445e+02 &

7.95249005168754e+02 7.42957763671875e+02 3.77395501535433e+02 &

7.96250595319160e+02 7.36616698982022e+02 3.74074088190253e+02 &

face id 2 &

7.99156871754180e+02 7.42957763671875e+02 3.74726900763980e+02 &

7.93502350955747e+02 7.42957763671875e+02 3.73386897874445e+02 &

7.96250595319160e+02 7.36616698982022e+02 3.74074088190253e+02 &

face id 2 &

7.95249005168754e+02 7.42957763671875e+02 3.77395501535433e+02 &

7.99156871754180e+02 7.42957763671875e+02 3.74726900763980e+02 &

7.96250595319160e+02 7.36616698982022e+02 3.74074088190253e+02

; block 416

poly group "10001" mat 1 con 1 &

face id 1 &

8.29833687142197e+02 7.42957763671875e+02 3.79453943973010e+02 &

8.20704762342298e+02 7.42957763671875e+02 3.77856341162262e+02 &

8.28903935786781e+02 7.42957763671875e+02 3.71492197084357e+02 &

face id 2 &

8.20704762342298e+02 7.42957763671875e+02 3.77856341162262e+02 &

8.29833687142197e+02 7.42957763671875e+02 3.79453943973010e+02 &

8.24640127572402e+02 7.37081027998942e+02 3.77991472841031e+02 &

face id 2 &

8.28903935786781e+02 7.42957763671875e+02 3.71492197084357e+02 &

8.20704762342298e+02 7.42957763671875e+02 3.77856341162262e+02 &

8.24640127572402e+02 7.37081027998942e+02 3.77991472841031e+02 &

face id 2 &

8.29833687142197e+02 7.42957763671875e+02 3.79453943973010e+02 &

8.28903935786781e+02 7.42957763671875e+02 3.71492197084357e+02 &

8.24640127572402e+02 7.37081027998942e+02 3.77991472841031e+02

; block 417

poly group "10001" mat 1 con 1 &

face id 2 &

8.18126133140210e+02 7.39128284797895e+02 4.24521969903783e+02 &

8.13756389123963e+02 7.39589893800934e+02 4.25916239240713e+02 &

8.13694588926832e+02 7.34627007610324e+02 4.24114636773096e+02 &

face id 2 &

8.13756389123963e+02 7.39589893800934e+02 4.25916239240713e+02 &

8.18126133140210e+02 7.39128284797895e+02 4.24521969903783e+02 &

8.16363471593303e+02 7.36867764055710e+02 4.27997707946941e+02 &

face id 2 &

8.13694588926832e+02 7.34627007610324e+02 4.24114636773096e+02 &

8.13756389123963e+02 7.39589893800934e+02 4.25916239240713e+02 &

8.16363471593303e+02 7.36867764055710e+02 4.27997707946941e+02 &

face id 2 &

8.18126133140210e+02 7.39128284797895e+02 4.24521969903783e+02 &

8.13694588926832e+02 7.34627007610324e+02 4.24114636773096e+02 &

8.16363471593303e+02 7.36867764055710e+02 4.27997707946941e+02

; block 418

poly group "10001" mat 1 con 1 &

face id 1 &

8.27899766625565e+02 7.31153198242188e+02 4.37953027515864e+02 &

8.25415422619949e+02 7.31153198242188e+02 4.41338127497763e+02 &

8.29507144346306e+02 7.31153198242188e+02 4.41289893556219e+02 &

face id 2 &

8.25415422619949e+02 7.31153198242188e+02 4.41338127497763e+02 &

8.27899766625565e+02 7.31153198242188e+02 4.37953027515864e+02 &

8.26114508878027e+02 7.34878405664679e+02 4.40774789501596e+02 &

face id 2 &

8.29507144346306e+02 7.31153198242188e+02 4.41289893556219e+02 &

8.25415422619949e+02 7.31153198242188e+02 4.41338127497763e+02 &

8.26114508878027e+02 7.34878405664679e+02 4.40774789501596e+02 &

face id 2 &

8.27899766625565e+02 7.31153198242188e+02 4.37953027515864e+02 &

8.29507144346306e+02 7.31153198242188e+02 4.41289893556219e+02 &

8.26114508878027e+02 7.34878405664679e+02 4.40774789501596e+02

; block 419

poly group "10001" mat 1 con 1 &

face id 2 &

8.29507144346306e+02 7.31153198242188e+02 4.41289893556219e+02 &

8.30150338633184e+02 7.35459866734055e+02 4.38544963342445e+02 &

8.27899766625565e+02 7.31153198242188e+02 4.37953027515864e+02 &

face id 2 &

8.30150338633184e+02 7.35459866734055e+02 4.38544963342445e+02 &

8.29507144346306e+02 7.31153198242188e+02 4.41289893556219e+02 &

8.26114508878027e+02 7.34878405664679e+02 4.40774789501596e+02 &

face id 2 &

8.27899766625565e+02 7.31153198242188e+02 4.37953027515864e+02 &

8.30150338633184e+02 7.35459866734055e+02 4.38544963342445e+02 &

8.26114508878027e+02 7.34878405664679e+02 4.40774789501596e+02 &

face id 2 &

8.29507144346306e+02 7.31153198242188e+02 4.41289893556219e+02 &

8.27899766625565e+02 7.31153198242188e+02 4.37953027515864e+02 &

8.26114508878027e+02 7.34878405664679e+02 4.40774789501596e+02

; block 420

poly group "10001" mat 1 con 1 &

face id 2 &

8.14181028467167e+02 7.32132772428309e+02 4.34887560622655e+02 &

8.15793233645232e+02 7.34747728264843e+02 4.35883786023879e+02 &

8.13616534804187e+02 7.34858466472432e+02 4.33854840236454e+02 &

face id 2 &

8.15793233645232e+02 7.34747728264843e+02 4.35883786023879e+02 &

8.14181028467167e+02 7.32132772428309e+02 4.34887560622655e+02 &

8.12824694618945e+02 7.33817339552651e+02 4.36694878313933e+02 &

face id 2 &

8.13616534804187e+02 7.34858466472432e+02 4.33854840236454e+02 &

8.15793233645232e+02 7.34747728264843e+02 4.35883786023879e+02 &

8.12824694618945e+02 7.33817339552651e+02 4.36694878313933e+02 &

face id 2 &

8.14181028467167e+02 7.32132772428309e+02 4.34887560622655e+02 &

8.13616534804187e+02 7.34858466472432e+02 4.33854840236454e+02 &

8.12824694618945e+02 7.33817339552651e+02 4.36694878313933e+02

; block 421

poly group "10001" mat 1 con 1 &

face id 2 &

8.26215102552087e+02 7.42957763671875e+02 4.40024896299389e+02 &

8.27910339647761e+02 7.39308812863053e+02 4.44974237053554e+02 &

8.24231497582248e+02 7.39555545391830e+02 4.40655328606252e+02 &

face id 2 &

8.27910339647761e+02 7.39308812863053e+02 4.44974237053554e+02 &

8.26215102552087e+02 7.42957763671875e+02 4.40024896299389e+02 &

8.28516744472485e+02 7.39206897844698e+02 4.40026719289599e+02 &

face id 2 &

8.24231497582248e+02 7.39555545391830e+02 4.40655328606252e+02 &

8.27910339647761e+02 7.39308812863053e+02 4.44974237053554e+02 &

8.28516744472485e+02 7.39206897844698e+02 4.40026719289599e+02 &

face id 2 &

8.26215102552087e+02 7.42957763671875e+02 4.40024896299389e+02 &

8.24231497582248e+02 7.39555545391830e+02 4.40655328606252e+02 &

8.28516744472485e+02 7.39206897844698e+02 4.40026719289599e+02

; block 422

poly group "10001" mat 1 con 1 &

face id 2 &

8.25193073523086e+02 7.42957763671875e+02 4.47593921454763e+02 &

8.27910339647761e+02 7.39308812863053e+02 4.44974237053554e+02 &

8.26644054329157e+02 7.40940525295196e+02 4.49188869423419e+02 &

face id 2 &

8.27910339647761e+02 7.39308812863053e+02 4.44974237053554e+02 &

8.25193073523086e+02 7.42957763671875e+02 4.47593921454763e+02 &

8.24345516769850e+02 7.40101480767813e+02 4.44800655912060e+02 &

face id 2 &

8.26644054329157e+02 7.40940525295196e+02 4.49188869423419e+02 &

8.27910339647761e+02 7.39308812863053e+02 4.44974237053554e+02 &

8.24345516769850e+02 7.40101480767813e+02 4.44800655912060e+02 &

face id 2 &

8.25193073523086e+02 7.42957763671875e+02 4.47593921454763e+02 &

8.26644054329157e+02 7.40940525295196e+02 4.49188869423419e+02 &

8.24345516769850e+02 7.40101480767813e+02 4.44800655912060e+02

; block 423

poly group "10001" mat 1 con 1 &

face id 2 &

8.14343169924012e+02 7.42957763671875e+02 4.40026044980605e+02 &

8.13909788550154e+02 7.41293423121517e+02 4.40993346381461e+02 &

8.14838076915517e+02 7.39071150525117e+02 4.39175800078397e+02 &

face id 2 &

8.13909788550154e+02 7.41293423121517e+02 4.40993346381461e+02 &

8.14343169924012e+02 7.42957763671875e+02 4.40026044980605e+02 &

8.16975670140343e+02 7.39892790962574e+02 4.39960247842898e+02 &

face id 2 &

8.14838076915517e+02 7.39071150525117e+02 4.39175800078397e+02 &

8.13909788550154e+02 7.41293423121517e+02 4.40993346381461e+02 &

8.16975670140343e+02 7.39892790962574e+02 4.39960247842898e+02 &

face id 2 &

8.14343169924012e+02 7.42957763671875e+02 4.40026044980605e+02 &

8.14838076915517e+02 7.39071150525117e+02 4.39175800078397e+02 &

8.16975670140343e+02 7.39892790962574e+02 4.39960247842898e+02

; block 424

poly group "10001" mat 1 con 1 &

face id 1 &

8.14984905319849e+02 7.42957763671875e+02 3.89627546226137e+02 &

8.19171832620069e+02 7.42957763671875e+02 3.93190604054630e+02 &

8.13870124921521e+02 7.42957763671875e+02 3.95224545195568e+02 &

face id 2 &

8.19171832620069e+02 7.42957763671875e+02 3.93190604054630e+02 &

8.14984905319849e+02 7.42957763671875e+02 3.89627546226137e+02 &

8.18483672968706e+02 7.37593060216477e+02 3.91417863514613e+02 &

face id 2 &

8.13870124921521e+02 7.42957763671875e+02 3.95224545195568e+02 &

8.19171832620069e+02 7.42957763671875e+02 3.93190604054630e+02 &

8.18483672968706e+02 7.37593060216477e+02 3.91417863514613e+02 &

face id 2 &

8.14984905319849e+02 7.42957763671875e+02 3.89627546226137e+02 &

8.13870124921521e+02 7.42957763671875e+02 3.95224545195568e+02 &

8.18483672968706e+02 7.37593060216477e+02 3.91417863514613e+02

; block 425

poly group "10001" mat 1 con 1 &

face id 2 &

8.13870124921521e+02 7.42957763671875e+02 3.95224545195568e+02 &

8.10641773231557e+02 7.37725836349123e+02 3.93566404294409e+02 &

8.14984905319849e+02 7.42957763671875e+02 3.89627546226137e+02 &

face id 2 &

8.10641773231557e+02 7.37725836349123e+02 3.93566404294409e+02 &

8.13870124921521e+02 7.42957763671875e+02 3.95224545195568e+02 &

8.18483672968706e+02 7.37593060216477e+02 3.91417863514613e+02 &

face id 2 &

8.14984905319849e+02 7.42957763671875e+02 3.89627546226137e+02 &

8.10641773231557e+02 7.37725836349123e+02 3.93566404294409e+02 &

8.18483672968706e+02 7.37593060216477e+02 3.91417863514613e+02 &

face id 2 &

8.13870124921521e+02 7.42957763671875e+02 3.95224545195568e+02 &

8.14984905319849e+02 7.42957763671875e+02 3.89627546226137e+02 &

8.18483672968706e+02 7.37593060216477e+02 3.91417863514613e+02

; block 426

poly group "10001" mat 1 con 1 &

face id 1 &

8.10009578774608e+02 7.31153198242188e+02 3.86599906858927e+02 &

8.16732385140107e+02 7.31153198242188e+02 3.89315202949226e+02 &

8.15100275115170e+02 7.31153198242188e+02 3.82923343064734e+02 &

face id 2 &

8.16732385140107e+02 7.31153198242188e+02 3.89315202949226e+02 &

8.10009578774608e+02 7.31153198242188e+02 3.86599906858927e+02 &

8.12290681094227e+02 7.36396429920157e+02 3.89417525587450e+02 &

face id 2 &

8.15100275115170e+02 7.31153198242188e+02 3.82923343064734e+02 &

8.16732385140107e+02 7.31153198242188e+02 3.89315202949226e+02 &

8.12290681094227e+02 7.36396429920157e+02 3.89417525587450e+02 &

face id 2 &

8.10009578774608e+02 7.31153198242188e+02 3.86599906858927e+02 &

8.15100275115170e+02 7.31153198242188e+02 3.82923343064734e+02 &

8.12290681094227e+02 7.36396429920157e+02 3.89417525587450e+02

; block 427

poly group "10001" mat 1 con 1 &

face id 2 &

8.16732385140107e+02 7.31153198242188e+02 3.89315202949226e+02 &

8.18920370898699e+02 7.37329641873752e+02 3.85333219479087e+02 &

8.15100275115170e+02 7.31153198242188e+02 3.82923343064734e+02 &

face id 2 &

8.18920370898699e+02 7.37329641873752e+02 3.85333219479087e+02 &

8.16732385140107e+02 7.31153198242188e+02 3.89315202949226e+02 &

8.12290681094227e+02 7.36396429920157e+02 3.89417525587450e+02 &

face id 2 &

8.15100275115170e+02 7.31153198242188e+02 3.82923343064734e+02 &

8.18920370898699e+02 7.37329641873752e+02 3.85333219479087e+02 &

8.12290681094227e+02 7.36396429920157e+02 3.89417525587450e+02 &

face id 2 &

8.16732385140107e+02 7.31153198242188e+02 3.89315202949226e+02 &

8.15100275115170e+02 7.31153198242188e+02 3.82923343064734e+02 &

8.12290681094227e+02 7.36396429920157e+02 3.89417525587450e+02

; block 428

poly group "10001" mat 1 con 1 &

face id 1 &

8.39084289550781e+02 7.31153198242188e+02 4.04197801118395e+02 &

8.30305606368492e+02 7.31153198242188e+02 3.99328987610237e+02 &

8.32171457884459e+02 7.31153198242188e+02 4.08821396931575e+02 &

face id 2 &

8.30305606368492e+02 7.31153198242188e+02 3.99328987610237e+02 &

8.39084289550781e+02 7.31153198242188e+02 4.04197801118395e+02 &

8.33068678521100e+02 7.37328863609821e+02 4.05702979015694e+02 &

face id 2 &

8.32171457884459e+02 7.31153198242188e+02 4.08821396931575e+02 &

8.30305606368492e+02 7.31153198242188e+02 3.99328987610237e+02 &

8.33068678521100e+02 7.37328863609821e+02 4.05702979015694e+02 &

face id 2 &

8.39084289550781e+02 7.31153198242188e+02 4.04197801118395e+02 &

8.32171457884459e+02 7.31153198242188e+02 4.08821396931575e+02 &

8.33068678521100e+02 7.37328863609821e+02 4.05702979015694e+02

; block 429

poly group "10001" mat 1 con 1 &

face id 2 &

8.30534430583977e+02 7.37647109839753e+02 4.13260662990091e+02 &

8.39084289550781e+02 7.37086515635440e+02 4.08040178768829e+02 &

8.32171457884459e+02 7.31153198242188e+02 4.08821396931575e+02 &

face id 2 &

8.39084289550781e+02 7.37086515635440e+02 4.08040178768829e+02 &

8.30534430583977e+02 7.37647109839753e+02 4.13260662990091e+02 &

8.33068678521100e+02 7.37328863609821e+02 4.05702979015694e+02 &

face id 2 &

8.32171457884459e+02 7.31153198242188e+02 4.08821396931575e+02 &

8.39084289550781e+02 7.37086515635440e+02 4.08040178768829e+02 &

8.33068678521100e+02 7.37328863609821e+02 4.05702979015694e+02 &

face id 2 &

8.30534430583977e+02 7.37647109839753e+02 4.13260662990091e+02 &

8.32171457884459e+02 7.31153198242188e+02 4.08821396931575e+02 &

8.33068678521100e+02 7.37328863609821e+02 4.05702979015694e+02

; block 430

poly group "10001" mat 1 con 1 &

face id 1 &

8.14143386380350e+02 7.42957763671875e+02 4.19814593480705e+02 &

8.17389222175387e+02 7.42957763671875e+02 4.15634944560790e+02 &

8.18827984754551e+02 7.42957763671875e+02 4.21773857031163e+02 &

face id 2 &

8.17389222175387e+02 7.42957763671875e+02 4.15634944560790e+02 &

8.14143386380350e+02 7.42957763671875e+02 4.19814593480705e+02 &

8.16786864436763e+02 7.38348293032234e+02 4.19074465024219e+02 &

face id 2 &

8.18827984754551e+02 7.42957763671875e+02 4.21773857031163e+02 &

8.17389222175387e+02 7.42957763671875e+02 4.15634944560790e+02 &

8.16786864436763e+02 7.38348293032234e+02 4.19074465024219e+02 &

face id 2 &

8.14143386380350e+02 7.42957763671875e+02 4.19814593480705e+02 &

8.18827984754551e+02 7.42957763671875e+02 4.21773857031163e+02 &

8.16786864436763e+02 7.38348293032234e+02 4.19074465024219e+02

; block 431

poly group "10001" mat 1 con 1 &

face id 2 &

8.18663640401986e+02 7.31153198242188e+02 4.20323564658305e+02 &

8.22003939543759e+02 7.37603465338854e+02 4.20982666462121e+02 &

8.22198614973049e+02 7.31153198242188e+02 4.17110132334116e+02 &

face id 2 &

8.22003939543759e+02 7.37603465338854e+02 4.20982666462121e+02 &

8.18663640401986e+02 7.31153198242188e+02 4.20323564658305e+02 &

8.20437476796949e+02 7.36584150205391e+02 4.14823811708292e+02 &

face id 2 &

8.22198614973049e+02 7.31153198242188e+02 4.17110132334116e+02 &

8.22003939543759e+02 7.37603465338854e+02 4.20982666462121e+02 &

8.20437476796949e+02 7.36584150205391e+02 4.14823811708292e+02 &

face id 2 &

8.18663640401986e+02 7.31153198242188e+02 4.20323564658305e+02 &

8.22198614973049e+02 7.31153198242188e+02 4.17110132334116e+02 &

8.20437476796949e+02 7.36584150205391e+02 4.14823811708292e+02

; block 432

poly group "10001" mat 1 con 1 &

face id 2 &

8.22722716594357e+02 7.42957763671875e+02 4.17247040728472e+02 &

8.16786864436763e+02 7.38348293032234e+02 4.19074465024219e+02 &

8.17389222175387e+02 7.42957763671875e+02 4.15634944560790e+02 &

face id 2 &

8.16786864436763e+02 7.38348293032234e+02 4.19074465024219e+02 &

8.22722716594357e+02 7.42957763671875e+02 4.17247040728472e+02 &

8.20437476796949e+02 7.36584150205391e+02 4.14823811708292e+02 &

face id 2 &

8.17389222175387e+02 7.42957763671875e+02 4.15634944560790e+02 &

8.16786864436763e+02 7.38348293032234e+02 4.19074465024219e+02 &

8.20437476796949e+02 7.36584150205391e+02 4.14823811708292e+02 &

face id 2 &

8.22722716594357e+02 7.42957763671875e+02 4.17247040728472e+02 &

8.17389222175387e+02 7.42957763671875e+02 4.15634944560790e+02 &

8.20437476796949e+02 7.36584150205391e+02 4.14823811708292e+02

; block 433

poly group "10001" mat 1 con 1 &

face id 2 &

8.20842798872635e+02 7.37189646407413e+02 3.71833994279653e+02 &

8.25214620888485e+02 7.37064024906030e+02 3.64848876953125e+02 &

8.28903935786781e+02 7.42957763671875e+02 3.71492197084357e+02 &

face id 2 &

8.25214620888485e+02 7.37064024906030e+02 3.64848876953125e+02 &

8.20842798872635e+02 7.37189646407413e+02 3.71833994279653e+02 &

8.24925363977043e+02 7.31153198242188e+02 3.71989177722232e+02 &

face id 2 &

8.28903935786781e+02 7.42957763671875e+02 3.71492197084357e+02 &

8.25214620888485e+02 7.37064024906030e+02 3.64848876953125e+02 &

8.24925363977043e+02 7.31153198242188e+02 3.71989177722232e+02 &

face id 2 &

8.20842798872635e+02 7.37189646407413e+02 3.71833994279653e+02 &

8.28903935786781e+02 7.42957763671875e+02 3.71492197084357e+02 &

8.24925363977043e+02 7.31153198242188e+02 3.71989177722232e+02

; block 434

poly group "10001" mat 1 con 1 &

face id 2 &

8.12647078267193e+02 7.38844593444619e+02 4.17279149585504e+02 &

8.17389222175387e+02 7.42957763671875e+02 4.15634944560790e+02 &

8.14143386380350e+02 7.42957763671875e+02 4.19814593480705e+02 &

face id 2 &

8.17389222175387e+02 7.42957763671875e+02 4.15634944560790e+02 &

8.12647078267193e+02 7.38844593444619e+02 4.17279149585504e+02 &

8.16786864436763e+02 7.38348293032234e+02 4.19074465024219e+02 &

face id 2 &

8.14143386380350e+02 7.42957763671875e+02 4.19814593480705e+02 &

8.17389222175387e+02 7.42957763671875e+02 4.15634944560790e+02 &

8.16786864436763e+02 7.38348293032234e+02 4.19074465024219e+02 &

face id 2 &

8.12647078267193e+02 7.38844593444619e+02 4.17279149585504e+02 &

8.14143386380350e+02 7.42957763671875e+02 4.19814593480705e+02 &

8.16786864436763e+02 7.38348293032234e+02 4.19074465024219e+02

; block 435

poly group "10001" mat 1 con 1 &

face id 2 &

8.08851677477977e+02 7.42957763671875e+02 3.96344698776517e+02 &

8.10641773231557e+02 7.37725836349123e+02 3.93566404294409e+02 &

8.13870124921521e+02 7.42957763671875e+02 3.95224545195568e+02 &

face id 2 &

8.10641773231557e+02 7.37725836349123e+02 3.93566404294409e+02 &

8.08851677477977e+02 7.42957763671875e+02 3.96344698776517e+02 &

8.13648731082524e+02 7.37198409103348e+02 3.98003234961222e+02 &

face id 2 &

8.13870124921521e+02 7.42957763671875e+02 3.95224545195568e+02 &

8.10641773231557e+02 7.37725836349123e+02 3.93566404294409e+02 &

8.13648731082524e+02 7.37198409103348e+02 3.98003234961222e+02 &

face id 2 &

8.08851677477977e+02 7.42957763671875e+02 3.96344698776517e+02 &

8.13870124921521e+02 7.42957763671875e+02 3.95224545195568e+02 &

8.13648731082524e+02 7.37198409103348e+02 3.98003234961222e+02

; block 436

poly group "10001" mat 1 con 1 &

face id 1 &

8.12675986932833e+02 7.31153198242188e+02 4.01815573567969e+02 &

8.13668525515790e+02 7.31153198242188e+02 4.06737925704045e+02 &

8.18183731612104e+02 7.31153198242188e+02 4.04154982986739e+02 &

face id 2 &

8.13668525515790e+02 7.31153198242188e+02 4.06737925704045e+02 &

8.12675986932833e+02 7.31153198242188e+02 4.01815573567969e+02 &

8.14194519614525e+02 7.36222240308663e+02 4.03223835774641e+02 &

face id 2 &

8.18183731612104e+02 7.31153198242188e+02 4.04154982986739e+02 &

8.13668525515790e+02 7.31153198242188e+02 4.06737925704045e+02 &

8.14194519614525e+02 7.36222240308663e+02 4.03223835774641e+02 &

face id 2 &

8.12675986932833e+02 7.31153198242188e+02 4.01815573567969e+02 &

8.18183731612104e+02 7.31153198242188e+02 4.04154982986739e+02 &

8.14194519614525e+02 7.36222240308663e+02 4.03223835774641e+02

; block 437

poly group "10001" mat 1 con 1 &

face id 1 &

7.99205186443479e+02 7.33448512231380e+02 4.11444913745973e+02 &

7.97293155916416e+02 7.35149565914216e+02 4.10938424197682e+02 &

7.99102856185158e+02 7.36471630851171e+02 4.11961130736426e+02 &

face id 2 &

7.97293155916416e+02 7.35149565914216e+02 4.10938424197682e+02 &

7.99205186443479e+02 7.33448512231380e+02 4.11444913745973e+02 &

8.01038761361681e+02 7.33990064792097e+02 4.10041158352416e+02 &

face id 2 &

7.99102856185158e+02 7.36471630851171e+02 4.11961130736426e+02 &

7.97293155916416e+02 7.35149565914216e+02 4.10938424197682e+02 &

8.01038761361681e+02 7.33990064792097e+02 4.10041158352416e+02 &

face id 2 &

7.99205186443479e+02 7.33448512231380e+02 4.11444913745973e+02 &

7.99102856185158e+02 7.36471630851171e+02 4.11961130736426e+02 &

8.01038761361681e+02 7.33990064792097e+02 4.10041158352416e+02

; block 438

poly group "10001" mat 1 con 1 &

face id 2 &

7.74432015880905e+02 7.31153198242188e+02 3.72068947519307e+02 &

7.73884979376549e+02 7.35280033613756e+02 3.71475020333047e+02 &

7.71135925292969e+02 7.34076365039034e+02 3.68803624990762e+02 &

face id 2 &

7.73884979376549e+02 7.35280033613756e+02 3.71475020333047e+02 &

7.74432015880905e+02 7.31153198242188e+02 3.72068947519307e+02 &

7.71135925292969e+02 7.33451152287357e+02 3.72826619886730e+02 &

face id 2 &

7.71135925292969e+02 7.34076365039034e+02 3.68803624990762e+02 &

7.73884979376549e+02 7.35280033613756e+02 3.71475020333047e+02 &

7.71135925292969e+02 7.33451152287357e+02 3.72826619886730e+02 &

face id 2 &

7.74432015880905e+02 7.31153198242188e+02 3.72068947519307e+02 &

7.71135925292969e+02 7.34076365039034e+02 3.68803624990762e+02 &

7.71135925292969e+02 7.33451152287357e+02 3.72826619886730e+02

; block 439

poly group "10001" mat 1 con 1 &

face id 2 &

8.00965458695504e+02 7.31153198242188e+02 3.91758115901608e+02 &

7.96478775035514e+02 7.34528043696007e+02 3.93001842890862e+02 &

8.00446633691467e+02 7.34890585746234e+02 3.94456651409666e+02 &

face id 2 &

7.96478775035514e+02 7.34528043696007e+02 3.93001842890862e+02 &

8.00965458695504e+02 7.31153198242188e+02 3.91758115901608e+02 &

7.99028431323575e+02 7.35618187258196e+02 3.90154438356032e+02 &

face id 2 &

8.00446633691467e+02 7.34890585746234e+02 3.94456651409666e+02 &

7.96478775035514e+02 7.34528043696007e+02 3.93001842890862e+02 &

7.99028431323575e+02 7.35618187258196e+02 3.90154438356032e+02 &

face id 2 &

8.00965458695504e+02 7.31153198242188e+02 3.91758115901608e+02 &

8.00446633691467e+02 7.34890585746234e+02 3.94456651409666e+02 &

7.99028431323575e+02 7.35618187258196e+02 3.90154438356032e+02

; block 440

poly group "10001" mat 1 con 1 &

face id 1 &

8.20992676355960e+02 7.42957763671875e+02 4.09981770246072e+02 &

8.15234808544163e+02 7.42957763671875e+02 4.09308534149983e+02 &

8.18069174339446e+02 7.42957763671875e+02 4.01829283575629e+02 &

face id 2 &

8.15234808544163e+02 7.42957763671875e+02 4.09308534149983e+02 &

8.20992676355960e+02 7.42957763671875e+02 4.09981770246072e+02 &

8.18044212102941e+02 7.37294444794664e+02 4.06947605911675e+02 &

face id 2 &

8.18069174339446e+02 7.42957763671875e+02 4.01829283575629e+02 &

8.15234808544163e+02 7.42957763671875e+02 4.09308534149983e+02 &

8.18044212102941e+02 7.37294444794664e+02 4.06947605911675e+02 &

face id 2 &

8.20992676355960e+02 7.42957763671875e+02 4.09981770246072e+02 &

8.18069174339446e+02 7.42957763671875e+02 4.01829283575629e+02 &

8.18044212102941e+02 7.37294444794664e+02 4.06947605911675e+02

; block 441

poly group "10001" mat 1 con 1 &

face id 2 &

7.91673311097484e+02 7.42957763671875e+02 4.02025301271298e+02 &

7.91616421383069e+02 7.39816178005118e+02 4.04519104605588e+02 &

7.95632045078570e+02 7.39829233879320e+02 4.01197287506287e+02 &

face id 1 &

7.91616421383069e+02 7.39816178005118e+02 4.04519104605588e+02 &

7.91673311097484e+02 7.42957763671875e+02 4.02025301271298e+02 &

7.93561308374798e+02 7.42957763671875e+02 4.04816605543573e+02 &

face id 2 &

7.95632045078570e+02 7.39829233879320e+02 4.01197287506287e+02 &

7.91616421383069e+02 7.39816178005118e+02 4.04519104605588e+02 &

7.93561308374798e+02 7.42957763671875e+02 4.04816605543573e+02 &

face id 2 &

7.91673311097484e+02 7.42957763671875e+02 4.02025301271298e+02 &

7.95632045078570e+02 7.39829233879320e+02 4.01197287506287e+02 &

7.93561308374798e+02 7.42957763671875e+02 4.04816605543573e+02

; block 442

poly group "10001" mat 1 con 1 &

face id 2 &

7.96157367511355e+02 7.35031216448138e+02 3.81369527881585e+02 &

7.89808816568164e+02 7.35078718869257e+02 3.82888823368539e+02 &

7.91306819168008e+02 7.39396483628046e+02 3.82381074655449e+02 &

face id 2 &

7.89808816568164e+02 7.35078718869257e+02 3.82888823368539e+02 &

7.96157367511355e+02 7.35031216448138e+02 3.81369527881585e+02 &

7.92853233387824e+02 7.36309746732437e+02 3.77720728805979e+02 &

face id 2 &

7.91306819168008e+02 7.39396483628046e+02 3.82381074655449e+02 &

7.89808816568164e+02 7.35078718869257e+02 3.82888823368539e+02 &

7.92853233387824e+02 7.36309746732437e+02 3.77720728805979e+02 &

face id 2 &

7.96157367511355e+02 7.35031216448138e+02 3.81369527881585e+02 &

7.91306819168008e+02 7.39396483628046e+02 3.82381074655449e+02 &

7.92853233387824e+02 7.36309746732437e+02 3.77720728805979e+02

; block 443

poly group "10001" mat 1 con 1 &

face id 2 &

8.01551593806463e+02 7.37121430531446e+02 3.83226223248936e+02 &

7.98831708908744e+02 7.36125522704012e+02 3.77926771882722e+02 &

8.01714828031126e+02 7.31153198242188e+02 3.79707377436264e+02 &

face id 2 &

7.98831708908744e+02 7.36125522704012e+02 3.77926771882722e+02 &

8.01551593806463e+02 7.37121430531446e+02 3.83226223248936e+02 &

7.96157367511355e+02 7.35031216448138e+02 3.81369527881585e+02 &

face id 2 &

8.01714828031126e+02 7.31153198242188e+02 3.79707377436264e+02 &

7.98831708908744e+02 7.36125522704012e+02 3.77926771882722e+02 &

7.96157367511355e+02 7.35031216448138e+02 3.81369527881585e+02 &

face id 2 &

8.01551593806463e+02 7.37121430531446e+02 3.83226223248936e+02 &

8.01714828031126e+02 7.31153198242188e+02 3.79707377436264e+02 &

7.96157367511355e+02 7.35031216448138e+02 3.81369527881585e+02

; block 444

poly group "10001" mat 1 con 1 &

face id 1 &

7.96007174242569e+02 7.31153198242188e+02 3.86339457867366e+02 &

8.00423884947867e+02 7.31153198242188e+02 3.87599039819564e+02 &

7.98907867856302e+02 7.31153198242188e+02 3.83142656318648e+02 &

face id 2 &

8.00423884947867e+02 7.31153198242188e+02 3.87599039819564e+02 &

7.96007174242569e+02 7.31153198242188e+02 3.86339457867366e+02 &

7.98654543635136e+02 7.35451075167486e+02 3.85750035024265e+02 &

face id 2 &

7.98907867856302e+02 7.31153198242188e+02 3.83142656318648e+02 &

8.00423884947867e+02 7.31153198242188e+02 3.87599039819564e+02 &

7.98654543635136e+02 7.35451075167486e+02 3.85750035024265e+02 &

face id 2 &
[truncated: 2,166,807 more chars]
